# Identifying Dysregulated lncRNA-Associated ceRNA Network Biomarkers in CML Based on Dynamical Network Biomarkers

**DOI:** 10.1155/2020/5189549

**Published:** 2020-02-18

**Authors:** Junhua Xu, Min Wu, Yichen Sun, Hongqian Zhao, Yujie Wang, Jie Gao

**Affiliations:** School of Science, Jiangnan University, Wuxi 214122, China

## Abstract

The incidence of chronic myeloid leukemia (CML) is increasing year by year, which is a serious threat to human health. Early diagnosis can reduce mortality and improve prognosis. LncRNAs have been shown to be effective biomarkers for a variety of diseases and can act as competitive endogenous RNA (ceRNA). In this study, the dysregulated lncRNA-associated ceRNA networks (DLCN) of the chronic phase (CP), accelerated phase (AP), and blastic crisis (BC) for CML are constructed. Then, based on dynamic network biomarkers (DNB), some dysregulated lncRNA-associated ceRNA network biomarkers of CP, AP, and BC for CML are identified according to DNB criteria. Thus, a lncRNA (SNHG5) is identified from DLCN_CP, a lncRNA (DLEU2) is identified from DLCN_AP, and two lncRNAs (SNHG3, SNHG5) are identified from DLCN_BC. In addition, the critical index (CI) used to detect disease outbreaks shows that CML occurs in CP, which is consistent with clinical medicine. By analyzing the functions of the identified ceRNA network biomarkers, it has been found that they are effective lncRNA biomarkers directly or indirectly related to CML. The result of this study will help deepen the understanding of CML pathology from the perspective of ceRNA and help discover the effective biomarkers of CP, AP, and BC for CML in the future, so as to help patients get timely treatment and reduce the mortality of CML.

## 1. Introduction

Chronic myeloid leukemia (CML) is a clonal malignant proliferative disease of hematopoietic stem cells (HSCs). The main hallmark is the formation of BCR-ABL fusion gene at the molecular level [1]. BCR-ABL is a chimeric oncogene arising from t (9; 22) (q34; q11) chromosomal translocation. The resultant protein-tyrosine kinase (PTK) drives signaling events and transforms HSCs. BCR-ABL activity in HSC causes CML [2]. Generally, CML is divided into three phases. Initially, CML presents in the chronic phase (CP), and it will invariably transform through the acceleration phase (AP) without curative intervention, progression then proceeds to blast crisis (BC). BC is highly resistant to treatment. Patients generally die of infection and bleeding complications due to a lack of normal granulocytes and platelets [2]. Although the BCR-ABL fusion gene has been determined as a pathogenic gene of CML, it is only used for the diagnosis of CML and does not reflect the molecular mechanism and three stages of CML.

Noncoding RNAs constitute 80–90% of the human transcriptome. They are involved in numerous gene regulation processes in cells that lead to complex diseases, especially cancer [3]. Studies have shown that coding and noncoding RNAs can regulate expression by targeting their common miRNAs [4]. This mechanism is called competitive endogenous RNA (ceRNA) [5]. In detail, multiple RNA transcripts containing miRNA binding sites can compete for the same miRNA to achieve mutual regulation, thereby affecting gene expression in cells.

Recent studies have shown that lncRNA is related to CML closely. Xiao et al. demonstrated that UCA1 functions as a ceRNA of MDR1 through completely binding to the common miR-16. UCA1-MDR1 might be a novel target for enhancing the therapeutic efficacy of CML patients with IM resistance [6]. He et al. found that SNHG5 promoted imatinib resistance in CML via acting as a ceRNA against miR-205-5p [7]. Sen et al. constructed a ceRNA network for the CML cell line, and the top ranked significant functional modules in the ceRNA network displayed cancer associated attributes and revealed putative regulators in CML pathogenesis [8].

In this study, the dysregulated lncRNA-associated ceRNA networks (DLCN) of CP, AP, and BC for CML are constructed by integrating regulatory interactions among lncRNAs, miRNAs, and mRNAs and expression profile data. Next, lncRNA-associated ceRNA network biomarkers in the three DLCNs based on dynamic network biomarkers (DNB) proposed by Chen et al. [9] are identified. Three potential lncRNAs (SNHG5, SNHG3, DLEU2) are uncovered as functional ceRNAs with key roles in the pathogenesis of CML. In addition, the critical index (CI) constructed to detect disease outbreaks shows that CML occurs in CP, which is consistent with clinical medicine. Then, functional enrichment analysis is performed on the identified ceRNA network biomarkers, and the role of the 3 lncRNAs in CML is validated by KEGG enrichment analysis and literature mining. These novel lncRNAs acting as ceRNAs at the posttranscriptional level may become promising diagnostic biomarkers and therapeutic targets.

## 2. Materials and Methods

### 2.1. Microarray Data

The gene expression profile of GSE47927 based on Affymetrix Human Gene 1.0 ST Array is downloaded from the National Center for Biotechnology Information's Gene Expression Omnibus (GEO) database (http://www.ncbi.nlm.nih.gov/geo/). CML is a clonal malignant proliferative disease of HSCs. BCR-ABL activity in HSC causes CML. 3 normal samples, 6 CP patient samples, 4 AP patient samples, and 2 BC patient samples of HSCs are chosen.

The probe set in the HuGene-1_0-st array is reannotated to obtain the corresponding lncRNA expression profile data. First, the probe set of the Affymetrix HuGene-1_0-st array is remapped to the human genome (GRCh38) using SeqMap. If the probe set maps to the human genome uniquely without a mismatch, the probe set is retained. Second, the chromosomal location of the above probe set matches the chromosomal location of the lncRNA from the GENCODE project [5]. Finally, 848 lncRNA expression profiles are obtained. For the mRNA expression profile, if multiple probes are mapped to the same gene, the average is taken, and finally 18288 mRNA expression profiles are obtained.

### 2.2. Gene Expression Profile Analysis

The significantly differentially expressed (SDE) genes are detected by *t*-test and multiple test corrections. Set the *p* value of 0.05 and the fold change of 1.5 to obtain SDE mRNAs and SDE lncRNAs between CP, AP, and BC in CML and normal samples.

By integrating the experimentally validated miRNA-mRNA interactions downloaded from TarBase v8.0 database and starBase v2.0 databases, 769,832 nonredundant miRNA-mRNA interactions are obtained. The experimentally validated miRNA-lncRNA interactions are downloaded from starBase v2.0 database and 10,213 miRNA-lncRNA relationships are obtained.

In order to identify dysregulated lncRNA-mRNA competitive interaction in CP, AP, and BC for CML. Based on the “ceRNA hypothesis,” a hypergeometric test is used to screen lncRNA-mRNA competition pairs with *p* value <0.05, which are called candidate lncRNA-mRNA competitive interactions and 12071 candidate lncRNA-mRNA competitive interactions are obtained as follows:

(1) $$\begin{array}{c} p=\sum_{i=N_{c}}^{\min\left(N_{m},N_{\ln\;c}\right)}\frac{\left(\begin{array}{c} N_{m}\\ i \end{array}\right)\left(\begin{array}{c} N_{\text{Total}}-N_{m}\\ N_{\ln\;c}-i \end{array}\right)}{\left(\begin{array}{c} N_{\text{Total}}\\ N_{\ln\;c} \end{array}\right)}, \end{array}$$

where *N*_Total_ is the number of common miRNAs between all human miRNAs targeted human mRNAs and all human miRNAs regulated all human lncRNAs. *N*_*m*_ is the number of miRNAs targeted a given mRNA. *N*_ln *c*_ is the number of miRNAs which regulated a given lncRNA. *N*_*c*_ is the number of common miRNAs shared by mRNA and lncRNA.

Next, the Pearson correlation coefficient (PCC) is used to identify mRNA-lncRNA competitive interactions. The PCC between mRNA and lncRNA is correlated positively, defining the mRNA-lncRNA competitive interaction. In order to improve the reliability of the results, lncRNA-mRNA pairs with PCC >0.5 are selected as follows:

(2) $$\begin{array}{c} \text{PCC}\left(x,y\right)=\frac{\sum_{i=1}^{n}\left(x_{i}-\bar{x}\right)\left(y_{i}-\bar{y}\right)}{\sqrt{\sum_{i=1}^{n}{\left(x_{i}-\bar{x}\right)}^{2}\sum_{i=1}^{n}{\left(y_{i}-\bar{y}\right)}^{2}}}, \end{array}$$

where *x*_*i*_ and *y*_*i*_ represent the expressions of gene *x* and gene *y* of the *i*-th sample in the reference sample, respectively. $\bar{x}$ and $\bar{y}$ represent the average gene expression of gene *x* and gene *y* in the reference sample, respectively.

### 2.3. Identifying lncRNA Biomarkers Based on DNB

Generally, each person or sample has multiple modules. To further identify lncRNA biomarkers, DNB is used to detect potential modules. Dysregulated mRNA-lncRNA competitive interactions obtained previously are clustered by hierarchical cluster analysis. According to the criteria for identification of DNB proposed by Chen et al. [9], the optimal group of genes or proteins is selected as DNB. Thus, a CI can be constructed to evaluate the DNB module and detect the critical state in multiple samples as follows:

(3) $$\begin{array}{c} {\text{CI}}_{t}=\frac{{\text{SD}}_{t}\times {\text{PCC}}_{t}}{{\text{OPCC}}_{t}}, \end{array}$$

where PCC_*t*_ is the average PCC of the DNB group at time *t* in absolute value. OPCC_*t*_ is the average PCC between the DNB group and the outside of the DNB group at time *t* in absolute value. SD_*t*_ is the standard deviation of the DNB group at time *t*.

## 3. Results and Discussion

### 3.1. Differentially Expressed mRNAs and lncRNAs

In this study, the dataset is divided into normal samples and CML patient samples in CP, AP, and BC, and the SDE genes are selected by *t*-test. 1920 SDE mRNAs in CP, 1786 SDE mRNAs in AP, and 2682 SDE mRNAs in BC are identified, respectively. 41 SDE lncRNAs in CP, 31 SDE lncRNAs in AP, and 80 SDE lncRNAs in BC are identified, respectively. Finally, in order to consider fully, 4399 SDE mRNAs and 107 SDE lncRNAs are obtained from the union set.

By integrating the regulatory relationship between mRNA and lncRNA in SDE genes, DLCN_CP, DLCN_AP, and DLCN_BC are constructed based on ceRNA theory. The dysregulated mRNA-lncRNA pairs of CP, AP, and BC for CML are identified as described in Materials and Methods. The result is shown in Table 1 (see Supplementary [Supplementary-material supplementary-material-1]). To further investigate the dysregulated mRNA-lncRNA pairs, the same mRNA and lncRNA are taken from three stages, and 14 lncRNAs and 858 mRNAs are obtained finally. That is, 1548, 1864, and 2386 mRNA-lncRNA pairs in CP, AP, and BC compete for interaction.

**Figure 1:**
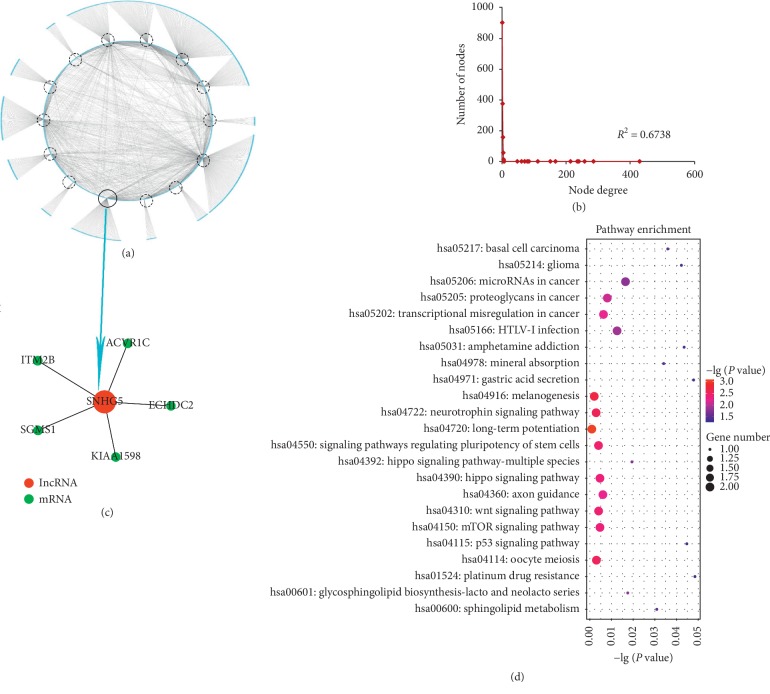
Network distribution and functional analysis of the DLCN_CP. (a) Global view of the DLCN_CP for CML. The DLCN_CP consists of 2436 edges between 1510 mRNAs and 14 lncRNAs. LncRNAs are circled in circles. (b) Degree distribution of nodes in the DLCN_CP. (c) DNB of the DLCN_CP. (d) Pathway enrichment analysis of DNB in the DLCN_CP.

**Figure 2:**
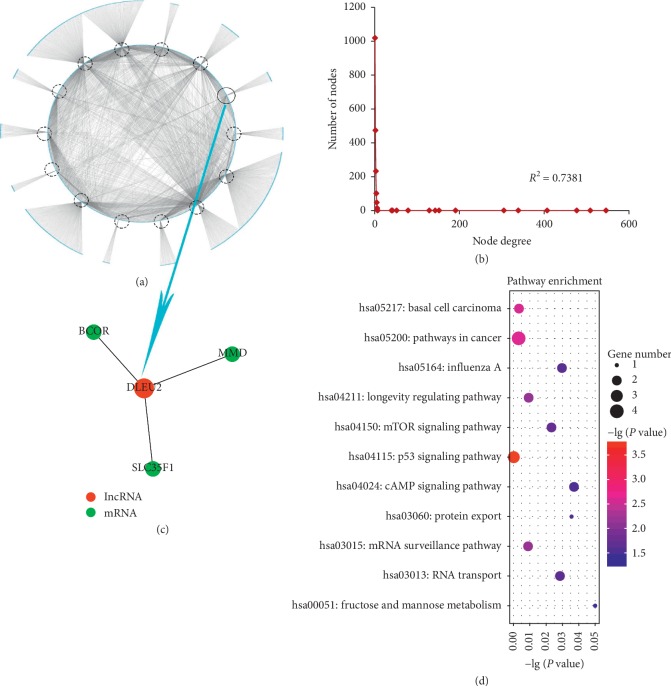
Network distribution and functional analysis of the DLCN_AP. (a) Global view of the DLCN_AP for CML. The DLCN_AP consists of 2436 edges between 1510 mRNAs and 14 lncRNAs. LncRNAs are circled in circles. (b) Degree distribution of nodes in the DLCN_AP. (c) DNB of the DLCN_AP. (d) Pathway enrichment analysis of DNB in the DLCN_AP.

**Figure 3:**
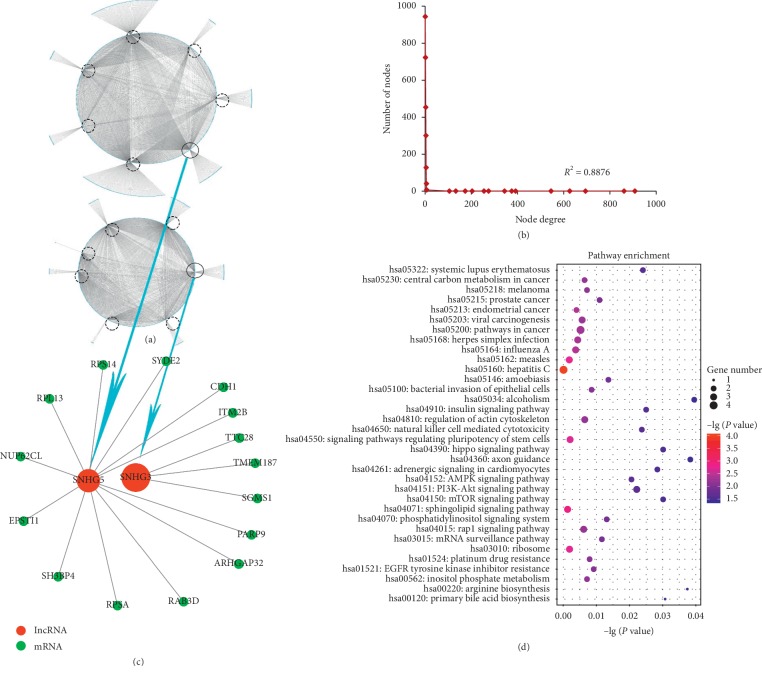
Network distribution and functional analysis of the DLCN_BC. (a) Global view of the DLCN_BC for CML. The DLCN_BC consists of 2436 edges between 1510 mRNAs and 14 lncRNAs. LncRNAs are circled in circles. (b) Degree distribution of nodes in the DLCN_BC. (c) DNB of the DLCN_BC. (d) Pathway enrichment analysis of DNB in the DLCN_BC.

In order to study the basic topological characteristics of biological networks, degree and degree distribution of the three networks are calculated, as shown in Figures 1(a), 2(a), 3(a) ,and Supplementary [Supplementary-material supplementary-material-1]. In DLCN_CP, the power law distribution of *R*^2^ = 0.6738 is observed (Figure 1(b)). In DLCN_AP, the power law distribution of *R*^2^ = 0.7381 is observed (Figure 2(b)). In DLCN_BC, the power law distribution of *R*^2^ = 0.8878 is observed (Figure 3(b)), indicating that only a few nodes of the three networks are connected to other nodes highly, and the correlation between nodes is relatively high. These nodes show typical scale-free features of biological networks, called hub genes.

### 3.2. Identifying Dysregulated lncRNA-Associated ceRNA Network Biomarkers Based on DNB in DLCN_CP, DLCN_AP, and DLCN_BC

**Figure 4:**
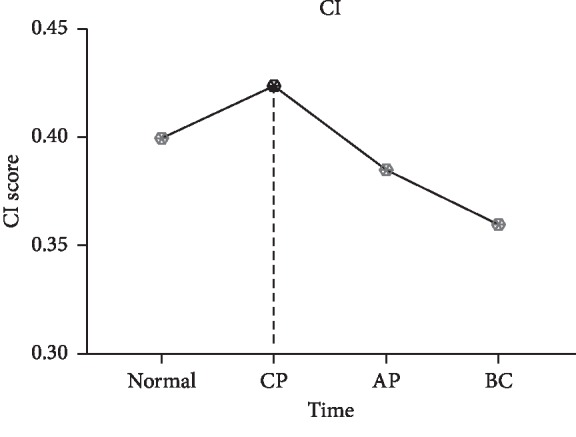
The CI of CML. Trends in CI used to detect disease outbreaks indicate that CML occurs in CP, which is consistent with clinical medicine.

To further investigate the competition between mRNA and lncRNA in CP, AP, and BC of CML, the dysregulated lncRNA-associated ceRNA network biomarkers are identified (see Table 2) based on DNB. First, hierarchical clustering is performed on DLCN_CP, DLCN_AP, and DLCN_BC, respectively. To make the classification of each period more obvious, the relative distance of each category at each period is set to 0.7. The three periods are divided into 9 categories, 8 categories, and 8 categories, respectively. After analyzing all categories by DNB, a group of 28 genes is identified as DNB in CP, which include 27 mRNAs and 1 lncRNA. A group of 62 genes is identified as DNB in AP, which include 61 mRNAs and 1 lncRNA. A group of 71 genes is identified as DNB in BC, which include 69 mRNAs and 2 lncRNAs. The dysregulated lncRNA-associated ceRNA network biomarkers in DLCN_CP are shown in Figure 1(c) and Supplementary [Supplementary-material supplementary-material-1], and lncRNA SNHG5 competes with five mRNAs. The dysregulated lncRNA-associated ceRNA network biomarkers in DLCN_AP are shown in Figure 2(c) and Supplementary [Supplementary-material supplementary-material-1], and lncRNA DLEU2 competes with three mRNAs. The dysregulated lncRNA-associated ceRNA network biomarkers in DLCN_BC are shown in Figure 3(c) and Supplementary [Supplementary-material supplementary-material-1], and two lncRNAs (SNHG3 and SNHG5) compete with 15 mRNAs.  Figure 4 shows the changes of CI in detail. In the process of CML, the CI value of CP is the largest, indicating that CML occurs in CP. And in clinical medicine, CML is found in the CP and begins to receive treatment. It has been reported that overexpression of SNHG5 inhibits the expression of mir-205-5p in CML patients and is related to the expression of mir-205-5p inversely, which has been shown to be a ceRNA against miR-205-5p to promote imatinib resistance in CML [7]. B-cell-specific activator protein (BSAP) interacts with the DLEU2 promoter directly. Derepression of the DLEU2 promoter via inhibition of histone deacetylation combined with BSAP knockdown increases miR-15a/16–1 expression and also increases malignant B-cell death. In summary, therapy targeting enhanced host gene DLEU2 transcription may augment CLL therapy [10].

To further investigate the biological function of each DNB, a significantly enriched KEGG pathway using the mRNA in each DNB is identified (Supplementary [Supplementary-material supplementary-material-1]). In DLCN_CP, several pathways well known in CML are enriched significantly, such as Wnt signaling pathway and p53 signaling pathway. In the canonical Wnt pathway, the primary role of Wnt ligands in binding to their receptors is to stabilize the cytoplasmic *β*-catenin by inhibiting the *β*-catenin degradation complex. Then *β*-catenin frees to enter the nucleus and activates Wnt-regulated genes through its interaction with TCF (T-cell factor) family transcription factors and concomitant recruitment of coactivator [11]. It has been reported that Wnt1 and *β*-catenin depletion and overexpression of nuclear *β*-catenin, together with TCF binding sites activation, demonstrates that ABCB1 in CML is positively regulated by the canonical pathway of Wnt signaling [12]. p53 is considered to be a universal sensor of genotoxic stress and is involved in different DNA repair mechanisms and in cell cycle checkpoint regulation through various signaling pathways. The resistance of CML to the tyrosine kinase inhibitor (imatinib) may be associated with persistent STAT5-mediated ROS production and the abnormality of the p53 pathway [13]. In addition, some other pathways are also related to CML closely, including the mTOR signaling pathway and the Hippo signaling pathway. In CML cells, autophagy and cytotoxicity induced by diosgenin are accompanied by the production of reactive oxygen species (ROS) and inhibition of the mTOR signaling pathway [14]. Developing drugs and/or identifying tumor markers of the Hippo signaling pathway and the Aurora kinase family, alone or in combination, can optimize CML treatment by enhancing the susceptibility of leukemia cells to apoptosis and leading to better disease prognosis [15]. In DLCN_AP, the p53 signaling pathway is well known in CML, and pathways in cancer and mTOR signaling pathway are related to CML. In DLCN_BC, the PI3K-Akt signaling pathway is related to CML. The phosphatidylinositol-3′-kinase (PI3K)-Akt signaling pathway is activated by many types of cellular stimuli or toxic insults and regulates fundamental cellular functions such as transcription, translation, proliferation, growth, and survival [16]. Further studies have shown that phosphatidylinositol-3 kinase (PI3K)/Akt/nuclear factor (NF)-*к*B signaling pathway is involved in CML pathogenesis [17]. Also, pathways in cancer, Phosphatidylinositol signaling system, Natural killer cell mediated cytotoxicity, Hippo signaling pathway, and mTOR signaling pathway are related to CML closely. Natural killer (NK) cells are lymphocytes of the innate immune system that are involved in the early defense of allogeneic (nonself) cells and autologous cells that undergo various forms of stress, such as infection with viruses, bacteria, or parasites or malignant transformation [18]. The presence of suppressor cells and perhaps an intrinsic inability of HNK-1+ cells may together contribute to the impaired NK cytotoxicity of low NK responder CML patients [19].

## 4. Conclusions

Studies have shown that lncRNA can be used as an effective biomarker for various diseases. In ceRNA theory, mRNA and lncRNA can regulate each other's expression by targeting their common miRNAs, which is important for understanding the progression of the disease. But little is known about CML. In this study, DLCN_CP, DLCN_AP, and DLCN_BC are constructed by integrating whole-genome lncRNA and mRNA expression profile data. To further investigate the competition between mRNA and lncRNA in CP, AP, and BC of CML, DNB is used to screen a module in the dysregulated lncRNA-associated ceRNA networks, and thus identify the lncRNA-associated ceRNA network biomarkers for CML. A lncRNA SNHG5 competes with five mRNAs in the DNB of DLCN_CP. A lncRNA DLEU2 competes with three mRNAs in the DNB of DLCN_AP. Two lncRNAs (SNHG3 and SNHG5) compete with 15 mRNAs in the DNB of DLCN_BC. Furthermore, the CI constructed to detect disease outbreaks shows that CML occurs in CP, which is consistent with clinical medicine. This also indicates that lncRNA biomarkers are effective. According to reports, SNHG5 is associated with CML. Although DLEU2 and SNHG3 are not related to CML directly, related materials show that DLEU2 is closely related to CLL, which provides us with new insights into ceRNA-mediated CML therapeutic regulatory mechanisms and facilitates the diagnosis of biomarkers and CML therapeutic targets.

Functional enrichment analysis of mRNAs coexpressed with lncRNAs reveals biological pathways of CP, AP, and BC in CML. Wnt signaling pathway is enriched significantly in DLCN_CP. It has been reported Wnt1 and *β*-catenin depletion and overexpression of nuclear *β*-catenin, together with TCF binding sites activation demonstrates that ABCB1 in CML is positively regulated by the canonical pathway of Wnt signaling. p53 signaling pathway is significantly enriched in DLCN_CP and DLCN_AP. The resistance of CML to the tyrosine kinase inhibitor (imatinib) may be associated with persistent STAT5-mediated ROS production, and the abnormality of the p53 pathway. PI3K-Akt signaling pathway is significantly enriched in DLCN_BC. Further studies have shown that phosphatidylinositol-3 kinase (PI3K)/Akt/nuclear factor (NF)-*к*B signaling pathway is involved in CML pathogenesis. Besides, pathways in cancer are significantly enriched in DLCN_CP, DLCN_AP, and DLCN_BC.

In summary, this study identifies three candidate sets of biomarkers in the ceRNA network based on DNB. The result will enhance our understanding of CML pathology from the ceRNA perspective and help us discover the effective biomarkers of CP, AP, and BC in CML, which help patients get timely treatment and thereby reduce the mortality rate of CML. This study only focuses on three periods of CML. In the future, it is hoped that the network can be built for the same patient at different time points, which will be more accurate in identifying biomarkers.

## Figures and Tables

**Table 1:** The dysregulated mRNA-lncRNA pairs of CP, AP, and BC for CML.

	DLCN_CP	DLCN_AP	DLCN_BC
mRNA-lncRNA interaction	2436	3411	5891
Node_lncRNA	14	14	14
Node_mRNA	1510	1892	2598

**Table 2:** The detailed information of the identified lncRNA biomarkers for CML CP, AP, and BC.

Ensemble ID	Gene name	Genomic location	Known research
ENSG00000203875	SNHG5	Chr6: 85677007–85678733 (−)	(1) Lymphoma [20] (2) Gastric cancer [21]
ENSG00000231607	DLEU2	Chr13: 50082169–50528643 (+)	(1) Chronic lymphocytic leukemia [22]
ENSG00000242125	SNHG3	Chr1: 28505943–285108929 (+)	(1) Hepatocellular carcinoma [23]

## Data Availability

The dataset used to analyze in the study can be downloaded from https://www.ncbi.nlm.nih.gov/geo/query/acc.cgi?acc=GSE47927.

## Supplementary Materials

Supplementary Materials Supplementary Table 1: dysregulated lncRNA-mRNA competing interactions of CP, AP, BC for CML. Supplementary Table 2: dysregulated lncRNA-associated ceRNA networks of CP, AP, and BC for CML (DLCN_CP, DLCN_AP, and DLCN_BC). Supplementary Table 3: CeRNA network biomarkers in DLCN_CP, DLCN_AP, and DLCN_BC. Supplementary Table 4: significantly enriched pathways in DLCN_CP, DLCN_AP, and DLCN_BC. .
