# Supplementary material for: Identifying Dysregulated lncRNA-Associated ceRNA Network Biomarkers in CML Based on Dynamical Network Biomarkers
Source: Biomed Res Int. 2020 Feb 18;2020:5189549. doi: 10.1155/2020/5189549 (PMC7049421; doi:10.1155/2020/5189549)
Supplement: Supplementary Materials — Supplementary Table 1: dysregulated lncRNA-mRNA competing interactions of CP, AP, BC for CML. Supplementary Table 2: dysregulated lncRNA-associated ceRNA networks of CP, AP, and BC for CML (DLCN_CP, DLCN_AP, and DLCN_BC). Supplementary Table 3: CeRNA network biomarkers in DLCN_CP, DLCN_AP, and DLCN_BC. Supplementary Table 4: significantly enriched pathways in DLCN_CP, DLCN_AP, and DLCN_BC. . [file 5189549.f1.zip › 5189549.f1/Supplementary Table S1.pdf]

Supplementary Table 1: Dysregulated lncRNA-mRNA competing interactions of CP, AP, BC for CML.

Dysregulated lncRNA-mRNA competing interactions of CP for CML			Dysregulated lncRNA-mRNA competing interactions of AP for CML			Dysregulated lncRNA-mRNA competing interactions of BC for CML		
mRNA	lncRNA	PCC	mRNA	lncRNA	PCC	mRNA	lncRNA	PCC
NDFIP1	SNHG3	0.996741781	AADAT	MCM3AP-AS1	0.95498742	AADAT	MCM3AP-AS1	1
CDKN1B	SNHG3	0.989205209	AADAT	ZNRD1-AS1	0.87761468	AAGAB	KTN1-AS1	1
FAM50B	KTN1-AS1	0.985996525	AAGAB	SNHG3	0.776647477	AAGAB	MCM3AP-AS1	1
ICA1L	ZNRD1-AS1	0.983257119	AATF	MCM3AP-AS1	0.861550311	AAGAB	SNHG3	1
GPAM	KTN1-AS1	0.980033424	ABCA5	DLEU2	0.669434074	AAK1	H19	1
PAFAH1B3	KTN1-AS1	0.978276221	ABCA5	MCM3AP-AS1	0.584602452	AAK1	SNHG5	1
CTNNAL1	MCM3AP-AS1	0.977956857	ABCA5	ZNRD1-AS1	0.736350547	AAK1	ZNRD1-AS1	1
TCF7L2	HCP5	0.977083723	ABCB10	HCP5	0.676051883	AATF	MCM3AP-AS1	1
SPIRE1	HCP5	0.976831295	ABCB6	H19	0.838829253	AATF	SNHG3	1
BEND5	SNHG3	0.97644022	ABCD1	KTN1-AS1	0.632491911	ABCA5	MCM3AP-AS1	1
FAM126A	SNHG3	0.976182574	ABHD14B	H19	0.635322579	ABCB10	HCP5	1
TMC7	SNHG5	0.975949161	ABHD14B	ZNRD1-AS1	0.650578923	ABCB10	SCARNA9	1
SERP1	RHPN1-AS1	0.975133637	ABHD15	H19	0.983534541	ABCB10	SNHG3	1
ZNF516	MCM3AP-AS1	0.974092116	ABHD4	H19	0.608524046	ABCB6	HCP5	1
STK38L	RUSC1-AS1	0.972629213	ABHD4	LINC00467	0.919994244	ABCC4	KTN1-AS1	1
EEPD1	HCP5	0.972618078	ABHD5	SNHG5	0.815293288	ABCD1	KTN1-AS1	1
FUCA1	KTN1-AS1	0.972516657	ABHD6	TP53TG1	0.862432713	ABCD3	DLEU2	1
ERO1L	HCP5	0.970465487	ABL1	TPT1-AS1	0.976875981	ABCD3	H19	1
CENPO	H19	0.970199354	ABL1	ZNRD1-AS1	0.975833329	ABCD3	TPT1-AS1	1
PDAP1	DLEU2	0.968575635	ABTB1	ZNRD1-AS1	0.885113758	ABCD3	ZNRD1-AS1	1
SYPL1	SCARNA9	0.9682777	ACACB	ZNRD1-AS1	0.856178033	ABHD15	HCP5	1
ASPH	RUSC1-AS1	0.967783596	ACADM	H19	0.696457961	ABHD5	HCP5	1
ZXDB	HCP5	0.967229367	ACADM	HCP5	0.626273888	ABHD5	MCM3AP-AS1	1
POLQ	H19	0.966717572	ACER3	HCP5	0.643395151	ABHD6	KTN1-AS1	1
SSBP3	HCP5	0.966053875	ACOT7	LINC00467	0.837352842	ABHD6	TP53TG1	1
ETS1	SNHG3	0.965990837	ACSL1	SNHG3	0.971944729	ABL1	DLEU2	1
PINK1	HCP5	0.964495596	ACSL1	SNHG5	0.826981256	ABL1	H19	1
MRPL16	SNHG3	0.96144649	ACSL4	SNHG3	0.950437607	ABL1	TPT1-AS1	1
CEP68	HCP5	0.961037848	ACSL4	SNHG5	0.750601647	ABL1	ZNRD1-AS1	1
C3orf58	SNHG5	0.958922902	ACSS1	ZNRD1-AS1	0.738116713	ABTB1	ZNRD1-AS1	1
ARID5B	SNHG5	0.958829012	ACTL6A	HCP5	0.574568799	ABTB2	H19	1
NSMCE2	TP53TG1	0.958392443	ACTL6A	LINC00467	0.531232779	ABTB2	ZNRD1-AS1	1
RPL23	SNHG5	0.958039802	ACTL6A	TP53TG1	0.766564086	ACAA2	SNHG3	1
CLIC4	SCARNA9	0.957416206	ACVR1B	SNHG3	0.870988852	ACACB	ZNRD1-AS1	1
GINS4	H19	0.956083626	ACVR1C	SNHG5	0.982294248	ACAD9	SNHG3	1
TXNL4A	SCARNA9	0.955600727	ADA	H19	0.78012998	ACADM	HCP5	1
GATM	SNHG3	0.955525655	ADA	HCP5	0.73942109	ACADM	KTN1-AS1	1
ATP2B4	SNHG3	0.95377592	ADAM10	SNHG3	0.957598007	ACADM	MCM3AP-AS1	1
ZNF681	HCP5	0.9526605	ADAM10	SNHG5	0.848285191	ACADM	SNHG3	1
FOSB	HCP5	0.95139388	ADAM19	SNHG5	0.81300676	ACAT2	KTN1-AS1	1
GOLPH3	SCARNA9	0.951140529	ADAT2	ZNRD1-AS1	0.719848636	ACAT2	TP53TG1	1
MLKL	ZNRD1-AS1	0.951029154	ADCY9	TP53TG1	0.670963417	ACER3	HCP5	1
ATXN1	RUSC1-AS1	0.950895677	ADD3	H19	0.712529942	ACER3	KTN1-AS1	1
PMM2	ZNRD1-AS1	0.950835978	ADD3	KTN1-AS1	0.890051075	ACER3	MCM3AP-AS1	1

FOS	HCP5	0.950155238	ADD3	LINC00467	0.992443986	ACER3	SNHG3	1
DDX21	H19	0.949986513	ADD3	MCM3AP-AS1	0.80439698	ACOT7	LINC00467	1
BTG1	HCP5	0.949232801	ADD3	ZNRD1-AS1	0.672336063	ACOT7	ZNRD1-AS1	1
MED10	SNHG3	0.948703217	ADK	H19	0.862289392	ACOT9	HCP5	1
KDM6B	DLEU2	0.948633996	ADK	HCP5	0.593255102	ACP2	MCM3AP-AS1	1
ADSS	SNHG3	0.948278577	ADK	KTN1-AS1	0.571532796	ACP2	SCARNA9	1
FADD	KTN1-AS1	0.948205617	ADPRH	H19	0.599355633	ACSL1	H19	1
L3MBTL4	SNHG3	0.947400379	ADRA2A	HCP5	0.999456777	ACSL1	RUSC1-AS1	1
LACTB	DLEU2	0.946184926	ADRA2A	KTN1-AS1	0.550403227	ACSL1	SNHG5	1
ABCA5	DLEU2	0.945403782	ADRA2A	TP53TG1	0.602659822	ACSL1	ZNRD1-AS1	1
TSR1	H19	0.944918614	ADRBK1	KTN1-AS1	0.997631902	ACSL4	DLEU2	1
GDE1	SNHG3	0.944520399	ADRBK1	MCM3AP-AS1	0.971601728	ACSL4	H19	1
HIST2H2BF	SNHG5	0.943810337	ADRBK2	MCM3AP-AS1	0.936461511	ACSL4	SNHG5	1
FAM117B	SNHG3	0.943118835	ADRBK2	ZNRD1-AS1	0.854549947	ACSL4	TPT1-AS1	1
C20orf194	ZNRD1-AS1	0.942997885	ADSS	H19	0.74210186	ACSL4	ZNRD1-AS1	1
SLC30A9	HCP5	0.942573382	ADSS	HCP5	0.60083369	ACTA2	HCP5	1
RABGAP1	ZNRD1-AS1	0.942138977	ADSS	TP53TG1	0.950220936	ACTL6A	HCP5	1
ADAT2	ZNRD1-AS1	0.941836077	AEN	H19	0.855691222	ACTL6A	TP53TG1	1
GABARAPL	LINC00467	0.941373994	AEN	HCP5	0.561326739	ACTN1	DLEU2	1
PNPLA4	HCP5	0.940727119	AEN	ZNRD1-AS1	0.650656528	ACTN1	H19	1
TMEM254	SNHG5	0.940548516	AFF3	ZNRD1-AS1	0.677003846	ACTN1	TPT1-AS1	1
AKT3	HCP5	0.940365199	AGAP4	KTN1-AS1	0.526790343	ACTN1	ZNRD1-AS1	1
KLHL2	H19	0.94031876	AGAP6	KTN1-AS1	0.647433923	ACVR1B	H19	1
PTPN4	HCP5	0.940280787	AGFG2	HCP5	0.849845512	ACVR1B	ZNRD1-AS1	1
SEPN1	LINC00467	0.939304371	AGFG2	MCM3AP-AS1	0.840053843	ACVR1C	KTN1-AS1	1
LSM5	HCP5	0.939082326	AGFG2	ZNRD1-AS1	0.801331892	ADA	H19	1
TBC1D4	SNHG3	0.938681602	AGMAT	HCP5	0.889121783	ADAM10	HCP5	1
GCHFR	SNHG5	0.937200577	AGTRAP	SNHG3	0.546843032	ADAM10	MCM3AP-AS1	1
RPS6KA4	HCP5	0.936674657	AHDC1	MCM3AP-AS1	0.612015971	ADAM10	SNHG3	1
PRKACB	SNHG3	0.936230256	AHDC1	ZNRD1-AS1	0.722565789	ADAM12	HCP5	1
SH2D4A	SNHG5	0.935326834	AHI1	DLEU2	0.619062819	ADAM12	MCM3AP-AS1	1
TIMP2	SNHG3	0.934638989	AIFM2	SNHG5	0.803798023	ADAM12	TP53TG1	1
HNRNPR	MCM3AP-AS1	0.934430717	AIM1	H19	0.926329536	ADAM15	SCARNA9	1
BCL11A	SNHG3	0.934149162	AKAP1	H19	0.780928313	ADAM17	RUSC1-AS1	1
DSC2	KTN1-AS1	0.933815246	AKAP1	HCP5	0.605428747	ADAM17	SNHG5	1
SLC25A15	H19	0.932038833	AKAP1	TPT1-AS1	0.52966601	ADAM19	DLEU2	1
HOXA7	LINC00467	0.931881398	AKAP1	ZNRD1-AS1	0.740783006	ADAM19	H19	1
MXI1	SNHG3	0.931832442	AKAP12	SNHG3	0.745428537	ADAM19	SNHG5	1
PCMTD2	SNHG3	0.930917989	AKAP12	SNHG5	0.865060792	ADAM19	TPT1-AS1	1
RBPMS	RUSC1-AS1	0.930333472	AKIRIN1	SNHG5	0.578552991	ADAM19	ZNRD1-AS1	1
UBE2G2	ZNRD1-AS1	0.929446474	AKR7A2	H19	0.906750002	ADAM8	HCP5	1
ZNF395	RHPN1-AS1	0.928985112	AKR7A2	HCP5	0.557667258	ADAMTS3	MCM3AP-AS1	1
TANK	DLEU2	0.928746966	AKT3	H19	0.652765392	ADAMTS7	HCP5	1
WASF1	RUSC1-AS1	0.928021635	AKT3	HCP5	0.746251995	ADAT2	HCP5	1
C11orf49	KTN1-AS1	0.927516817	AKT3	MCM3AP-AS1	0.865276849	ADCY1	KTN1-AS1	1
ITGA6	SNHG3	0.927116878	AKT3	TPT1-AS1	0.502758708	ADCY1	MCM3AP-AS1	1
GLTP	SNHG3	0.927007526	AKT3	ZNRD1-AS1	0.753400774	ADCY9	TP53TG1	1
ANXA6	SNHG3	0.92603783	AKTIP	SNHG3	0.900176546	ADD3	H19	1
NFAT5	DLEU2	0.925469239	ALCAM	SNHG3	0.780280917	ADD3	LINC00467	1
ATP9A	SNHG5	0.925136386	ALDH1A1	H19	0.612361692	ADD3	SNHG5	1
GPRIN3	HCP5	0.924821885	ALDH1A2	SNHG3	0.799669874	ADD3	ZNRD1-AS1	1

PDK4	KTN1-AS1	0.924617643	ALDH3B1	MCM3AP-AS1	0.908062843	ADK	H19	1
CAMK2G	MCM3AP-AS1	0.924591314	ALDH3B1	ZNRD1-AS1	0.839815011	ADK	RUSC1-AS1	1
GPD1L	SNHG3	0.923940554	ALDH7A1	HCP5	0.661220618	ADK	SNHG5	1
SLC20A2	HCP5	0.923442263	ALPK1	HCP5	0.612362804	ADK	ZNRD1-AS1	1
SP1	HCP5	0.922786543	ALPK1	KTN1-AS1	0.967388754	ADORA2B	HCP5	1
PIBF1	RUSC1-AS1	0.922703699	AMACR	H19	0.506049645	ADORA2B	TP53TG1	1
ZFP36	HCP5	0.922585269	AMOT	H19	0.620296219	ADPRH	H19	1
PNRC1	HCP5	0.922453722	AMOT	HCP5	0.870191576	ADPRH	ZNRD1-AS1	1
MARCKS	SNHG3	0.92209465	AMOT	MCM3AP-AS1	0.560437486	ADRA2A	HCP5	1
ADK	HCP5	0.921575862	AMPD2	H19	0.782432849	ADRA2A	KTN1-AS1	1
HEBP1	KTN1-AS1	0.921540276	AMPD2	ZNRD1-AS1	0.827346125	ADRA2A	MCM3AP-AS1	1
PTEN	MCM3AP-AS1	0.921392439	ANGPT1	H19	0.579032583	ADRA2A	SNHG3	1
APP	SNHG3	0.921268278	ANGPT1	MCM3AP-AS1	0.816432971	ADRA2A	TP53TG1	1
EPCAM	MCM3AP-AS1	0.920761792	ANGPT1	ZNRD1-AS1	0.702713326	ADRBK1	SNHG5	1
ARL13B	DLEU2	0.920258026	ANKAR	RUSC1-AS1	0.821232875	ADRBK2	MCM3AP-AS1	1
SLC5A3	SNHG3	0.919999371	ANKH	H19	0.771160043	ADSS	HCP5	1
C5	ZNRD1-AS1	0.918041295	ANKH	HCP5	0.521206417	ADSS	MCM3AP-AS1	1
C16orf87	RUSC1-AS1	0.917867204	ANKH	MCM3AP-AS1	0.905695453	ADSS	SNHG3	1
GLCE	SNHG5	0.917478101	ANKH	ZNRD1-AS1	0.80247019	ADSS	TP53TG1	1
ITGB8	RUSC1-AS1	0.917374643	ANKRA2	TPT1-AS1	0.968602782	AEN	HCP5	1
MRPL37	TP53TG1	0.917291857	ANKRA2	ZNRD1-AS1	0.943841799	AFF3	DLEU2	1
BRE	HCP5	0.916100063	ANKRD26	H19	0.560653945	AFF3	H19	1
TET3	DLEU2	0.91585218	ANKRD26	KTN1-AS1	0.755349771	AFF3	ZNRD1-AS1	1
TMEM19	SNHG3	0.91577631	ANKRD26	TPT1-AS1	0.881744841	AGAP6	SNHG5	1
TAB2	HCP5	0.915625385	ANKRD26	ZNRD1-AS1	0.794001049	AGFG2	HCP5	1
OTUD1	SNHG3	0.915421858	ANKRD28	MCM3AP-AS1	0.840246521	AGFG2	MCM3AP-AS1	1
GAB1	SNHG3	0.914860541	ANKRD28	ZNRD1-AS1	0.913085522	AGMAT	HCP5	1
P4HA1	DLEU2	0.914760991	ANKRD46	H19	0.72108944	AGPAT6	ZNRD1-AS1	1
FGGY	SNHG5	0.914740485	ANKRD46	HCP5	0.796775387	AGTRAP	SNHG3	1
SORL1	SNHG3	0.914230668	ANKRD46	MCM3AP-AS1	0.602158119	AHDC1	H19	1
PRRG1	KTN1-AS1	0.914227817	ANKRD6	MCM3AP-AS1	0.863573129	AHDC1	ZNRD1-AS1	1
PRDM2	ZNRD1-AS1	0.913661882	ANKRD6	ZNRD1-AS1	0.947508454	AHI1	KTN1-AS1	1
SENP5	DLEU2	0.912322008	ANLN	LINC00467	0.752802088	AHI1	MCM3AP-AS1	1
AADAT	ZNRD1-AS1	0.912240897	ANPEP	MCM3AP-AS1	0.993933183	AHNAK	DLEU2	1
FAM58A	SNHG3	0.911879293	ANPEP	ZNRD1-AS1	0.970427238	AHNAK	H19	1
CRTAP	KTN1-AS1	0.911343578	ANTXR1	H19	0.583801183	AHNAK	RUSC1-AS1	1
NUP88	ZNRD1-AS1	0.911053013	ANTXR1	TP53TG1	0.775565313	AHNAK	TPT1-AS1	1
YEATS4	SCARNA9	0.910713063	ANXA1	H19	0.800104465	AHNAK	ZNRD1-AS1	1
AKTIP	HCP5	0.910369553	AP1G2	MCM3AP-AS1	0.866220254	AIF1L	RHPN1-AS1	1
DENND4A	RUSC1-AS1	0.910250482	AP1G2	ZNRD1-AS1	0.905594905	AIM1	H19	1
ANKRD26	ZNRD1-AS1	0.91006912	AP1S3	HCP5	0.921789715	AKAP1	HCP5	1
SEC24A	DLEU2	0.909952729	AP1S3	MCM3AP-AS1	0.767739265	AKAP1	RHPN1-AS1	1
LPAR1	SNHG5	0.908181017	AP1S3	ZNRD1-AS1	0.681395749	AKAP1	SCARNA9	1
RALA	HCP5	0.908176203	AP3D1	MCM3AP-AS1	0.542222521	AKAP1	SNHG3	1
EPB41L3	SNHG5	0.908165603	AP3D1	TPT1-AS1	0.851851984	AKAP12	HCP5	1
CNPY4	SNHG3	0.90816366	AP3D1	ZNRD1-AS1	0.631934563	AKAP12	KTN1-AS1	1
ADAM15	SNHG5	0.90754379	AP3S1	HCP5	0.848646131	AKAP12	MCM3AP-AS1	1
C4orf19	TPT1-AS1	0.907165604	AP3S1	LINC00467	0.922384566	AKAP12	SNHG3	1
CENPK	H19	0.906976297	APBA2	RHPN1-AS1	0.588378699	AKAP12	TP53TG1	1
PRUNE	LINC00467	0.90657966	APBA2	SNHG3	0.642396834	AKAP13	H19	1
IQSEC1	HCP5	0.905791842	APC	SNHG3	0.80474888	AKAP13	ZNRD1-AS1	1

FAM46C	SNHG5	0.905701822	APC	SNHG5	0.588940291	AKAP7	MCM3AP-AS1	1
CTNNBIP1	HCP5	0.9048928	APOO	HCP5	0.733732712	AKIRIN1	HCP5	1
PHKA1	MCM3AP-AS1	0.90473217	APP	LINC00467	0.512058503	AKIRIN1	MCM3AP-AS1	1
CRIM1	HCP5	0.904622112	APP	MCM3AP-AS1	0.916012687	AKR7A2	HCP5	1
ZDHHC2	SNHG3	0.904562413	APP	ZNRD1-AS1	0.938401873	AKT3	DLEU2	1
CHAF1A	H19	0.904334125	APPL1	H19	0.842377364	AKT3	H19	1
SSBP3	RHPN1-AS1	0.903846461	APPL1	HCP5	0.58985282	AKT3	TPT1-AS1	1
GLI1	TPT1-AS1	0.903717356	APPL1	MCM3AP-AS1	0.789580446	AKT3	ZNRD1-AS1	1
CDK13	H19	0.90342458	APPL1	ZNRD1-AS1	0.648476382	AKTIP	HCP5	1
DHX33	ZNRD1-AS1	0.903400353	ARAP2	DLEU2	0.785574191	AKTIP	SNHG3	1
TMEM65	SNHG3	0.903390384	ARF5	H19	0.739821275	ALAS1	KTN1-AS1	1
KCTD12	TP53TG1	0.903386789	ARF5	RHPN1-AS1	0.600599792	ALAS1	MCM3AP-AS1	1
TFPI	HCP5	0.903354223	ARFGAP3	SNHG3	0.887220976	ALAS1	SNHG3	1
COL24A1	ZNRD1-AS1	0.903142253	ARHGAP10	HCP5	0.620714417	ALCAM	H19	1
CCSER1	SNHG3	0.902277191	ARHGAP10	ZNRD1-AS1	0.822266032	ALCAM	LINC00467	1
ERG	HCP5	0.902236381	ARHGAP12	H19	0.908703566	ALCAM	SNHG5	1
FOXO3	SNHG3	0.902205679	ARHGAP18	SNHG3	0.936211673	ALCAM	ZNRD1-AS1	1
ATPAF1	SNHG3	0.9015459	ARHGAP20	MCM3AP-AS1	0.819006473	ALDH1A1	SNHG3	1
TOB1	SNHG3	0.901510494	ARHGAP32	DLEU2	0.662818803	ALDH1A2	KTN1-AS1	1
LYRM1	RHPN1-AS1	0.901236032	ARHGAP32	SNHG5	0.901999851	ALDH1A2	MCM3AP-AS1	1
ANXA1	SNHG3	0.900296382	ARHGDIB	H19	0.681624704	ALDH1A2	SNHG3	1
EBF1	HCP5	0.899934763	ARHGDIB	HCP5	0.760222822	ALDH2	DLEU2	1
SLC25A25	DLEU2	0.899424602	ARHGEF10	HCP5	0.942975471	ALDH2	TPT1-AS1	1
ZNF217	SNHG3	0.898689078	ARHGEF2	TPT1-AS1	0.760453407	ALDH3B1	ZNRD1-AS1	1
TMEM109	KTN1-AS1	0.898576518	ARHGEF3	H19	0.767737567	ALDH5A1	KTN1-AS1	1
CCDC71L	HCP5	0.89856038	ARHGEF3	HCP5	0.741159683	ALDH6A1	SNHG5	1
GPAM	SNHG5	0.898442874	ARHGEF3	KTN1-AS1	0.809219668	ALG1	MCM3AP-AS1	1
WASL	HCP5	0.898293849	ARHGEF3	LINC00467	0.996713143	ALG13	H19	1
ZNF823	HCP5	0.898111527	ARHGEF3	ZNRD1-AS1	0.543755922	ALG13	ZNRD1-AS1	1
SASS6	H19	0.898018377	ARID4B	TPT1-AS1	0.684446566	ALG2	DLEU2	1
TFDP1	SNHG3	0.89789536	ARID5B	DLEU2	0.738640988	ALG2	H19	1
CAST	DLEU2	0.897050228	ARID5B	HCP5	0.687114275	ALG2	TPT1-AS1	1
BACE1	HCP5	0.896196755	ARID5B	RUSC1-AS1	0.675870527	ALG2	ZNRD1-AS1	1
EXOC4	SNHG3	0.896196567	ARL2	HCP5	0.73995576	ALPK1	HCP5	1
ZNF155	MCM3AP-AS1	0.896022768	ARL2	MCM3AP-AS1	0.863571731	ALPK1	KTN1-AS1	1
ANKRD28	SCARNA9	0.895767002	ARL2	ZNRD1-AS1	0.750155977	AMACR	SNHG3	1
ZMAT3	MCM3AP-AS1	0.895754877	ARL5A	H19	0.755440356	AMIGO2	KTN1-AS1	1
KIAA0430	RUSC1-AS1	0.895736674	ARL5B	RHPN1-AS1	0.724201988	AMIGO2	MCM3AP-AS1	1
ZFC3H1	ZNRD1-AS1	0.895318011	ARL5B	SNHG3	0.67483382	AMOT	H19	1
SLC5A3	KTN1-AS1	0.895287384	ARL5B	SNHG5	0.63666154	AMOT	RUSC1-AS1	1
PIK3C2B	HCP5	0.895198454	ARL6IP5	MCM3AP-AS1	0.612046326	AMOT	SNHG5	1
PUS7	ZNRD1-AS1	0.894766595	ARL6IP5	ZNRD1-AS1	0.501924343	AMOT	ZNRD1-AS1	1
REPS2	KTN1-AS1	0.894699626	ARL6IP6	SNHG3	0.786721476	AMPD2	HCP5	1
ALCAM	HCP5	0.894678699	ARMCX2	SNHG5	0.65504386	AMPD2	SNHG3	1
SAMD12	SNHG5	0.894209793	ARPC5L	RHPN1-AS1	0.944178782	AMPD3	H19	1
ZFP36L2	RUSC1-AS1	0.893234879	ARPC5L	SNHG3	0.974137981	AMPD3	LINC00467	1
RAB12	DLEU2	0.892044035	ARRDC4	H19	0.662526423	AMPD3	ZNRD1-AS1	1
EFNA3	HCP5	0.891919373	ARRDC4	KTN1-AS1	0.881708211	ANAPC1	SNHG5	1
CHD2	ZNRD1-AS1	0.891763557	ARRDC4	MCM3AP-AS1	0.886205146	ANGPT1	H19	1
EIF5	ZNRD1-AS1	0.89170082	ARRDC4	TPT1-AS1	0.860467814	ANGPT1	ZNRD1-AS1	1
HHLA3	LINC00467	0.891395653	ARRDC4	ZNRD1-AS1	0.857545691	ANKAR	RUSC1-AS1	1

KLHL24	RUSC1-AS1	0.891204107	ARSK	TP53TG1	0.863028086	ANKH	H19	1
SPIN4	KTN1-AS1	0.89094738	ASAH1	H19	0.559020626	ANKH	ZNRD1-AS1	1
TMEM106B	RHPN1-AS1	0.890666342	ASAH1	HCP5	0.906173159	ANKRA2	HCP5	1
RALA	SNHG3	0.890585521	ASAH1	KTN1-AS1	0.691313437	ANKRD12	HCP5	1
JAG2	TPT1-AS1	0.890302399	ASB13	H19	0.631582819	ANKRD12	MCM3AP-AS1	1
APC	H19	0.889968031	ASB9	H19	0.719108624	ANKRD26	DLEU2	1
ECHDC2	SNHG5	0.889953455	ASB9	HCP5	0.788673377	ANKRD26	H19	1
ANKRD28	HCP5	0.889761417	ASB9	ZNRD1-AS1	0.543814227	ANKRD26	TPT1-AS1	1
CHEK2	H19	0.889485625	ASCC3	SNHG3	0.716282225	ANKRD26	ZNRD1-AS1	1
ITGA2	SNHG3	0.889210567	ASF1B	H19	0.533556171	ANKRD28	H19	1
HNRNPR	ZNRD1-AS1	0.889118149	ASIC1	H19	0.628436115	ANKRD28	LINC00467	1
PSD3	SNHG5	0.888985678	ASIC1	HCP5	0.86443788	ANKRD28	SNHG5	1
HOXA3	MCM3AP-AS1	0.887798937	ASPH	H19	0.870264184	ANKRD28	ZNRD1-AS1	1
IVNS1ABP	ZNRD1-AS1	0.887281552	ASPH	MCM3AP-AS1	0.838055383	ANKRD46	HCP5	1
DIRC2	HCP5	0.886913376	ASPH	TPT1-AS1	0.595922554	ANKRD46	MCM3AP-AS1	1
6-Sep	HCP5	0.885617417	ASPH	ZNRD1-AS1	0.725914605	ANKRD6	HCP5	1
TRIB1	KTN1-AS1	0.884963447	ASPHD1	HCP5	0.685183804	ANKRD6	MCM3AP-AS1	1
HLA-DQB1	SNHG3	0.88467257	ATAD2	H19	0.5174067	ANLN	MCM3AP-AS1	1
SCAI	ZNRD1-AS1	0.884541247	ATAD2	HCP5	0.546662448	ANLN	SNHG3	1
SSU72	RHPN1-AS1	0.884222867	ATAD2B	MCM3AP-AS1	0.71496057	ANO3	MCM3AP-AS1	1
CHD7	H19	0.884197891	ATAD2B	TPT1-AS1	0.967813955	ANPEP	ZNRD1-AS1	1
CRIM1	SNHG3	0.884085809	ATAD5	MCM3AP-AS1	0.752993044	ANTXR1	MCM3AP-AS1	1
MLLT3	HCP5	0.883336508	ATAD5	TPT1-AS1	0.592498192	ANTXR1	SNHG3	1
CTTNBP2NI	DLEU2	0.883213139	ATAD5	ZNRD1-AS1	0.650664117	ANTXR1	TP53TG1	1
GMDS	SNHG5	0.883172907	ATF3	SNHG5	0.722821291	ANXA1	DLEU2	1
EPB41L2	H19	0.882753941	ATG4D	ZNRD1-AS1	0.953767688	ANXA1	H19	1
ALCAM	SNHG3	0.882211062	ATG5	SNHG3	0.924418376	ANXA6	RUSC1-AS1	1
PIK3R1	HCP5	0.881641847	ATL2	HCP5	0.596858257	AP1G2	MCM3AP-AS1	1
NUDCD3	SNHG5	0.881538362	ATL2	SCARNA9	0.68154281	AP1S3	HCP5	1
C11orf49	LINC00467	0.881365919	ATP13A3	SNHG3	0.736730232	AP1S3	MCM3AP-AS1	1
BACH2	HCP5	0.881341464	ATP1B1	SNHG3	0.680451119	AP3D1	DLEU2	1
JUNB	DLEU2	0.881184644	ATP1B1	SNHG5	0.804294393	AP3D1	H19	1
PLAA	ZNRD1-AS1	0.881004491	ATP2A2	SNHG3	0.842082855	AP3D1	TPT1-AS1	1
FPGT	SNHG5	0.880840836	ATP2A2	SNHG5	0.935841246	AP3D1	ZNRD1-AS1	1
TP53	HCP5	0.880350537	ATP2B1	H19	0.890401764	AP3S1	DLEU2	1
SEMA6D	SNHG3	0.880248631	ATP2B1	LINC00467	0.953741325	AP3S1	LINC00467	1
WEE1	H19	0.879928838	ATP2B1	MCM3AP-AS1	0.788658501	AP3S1	TPT1-AS1	1
GGCT	SNHG5	0.879914757	ATP2B1	ZNRD1-AS1	0.652582675	AP4E1	HCP5	1
PPAP2B	KTN1-AS1	0.879868409	ATP2B4	HCP5	0.861955341	AP4E1	TP53TG1	1
EMP1	SNHG3	0.879609297	ATP2B4	KTN1-AS1	0.7979222	APBA2	RHPN1-AS1	1
TBX19	ZNRD1-AS1	0.879537531	ATP2B4	MCM3AP-AS1	0.800564952	APBA2	SNHG3	1
FAIM3	LINC00467	0.878878312	ATP2B4	ZNRD1-AS1	0.772439765	APC	HCP5	1
KLF4	RHPN1-AS1	0.878774915	ATP6V0E2	KTN1-AS1	0.753006332	APC	MCM3AP-AS1	1
GATA3	HCP5	0.87815653	ATP6V0E2	MCM3AP-AS1	0.645885464	APC	SCARNA9	1
GGCT	KTN1-AS1	0.878105998	ATP7B	SNHG5	0.925966225	APC	SNHG3	1
FANCC	H19	0.877420614	ATP8A1	MCM3AP-AS1	0.890828797	APIP	SNHG3	1
AMOT	RUSC1-AS1	0.877127688	ATP8A1	TP53TG1	0.645902829	APOBEC3B	MCM3AP-AS1	1
SMO	SNHG3	0.876963091	ATP8A1	TPT1-AS1	0.564131081	APOL2	MCM3AP-AS1	1
DCTN4	SNHG3	0.876853708	ATP9A	LINC00467	0.960500142	APOO	HCP5	1
LMBR1L	DLEU2	0.87680568	ATPAF1	HCP5	0.684394296	APOO	MCM3AP-AS1	1
DUSP18	HCP5	0.876540454	ATPAF1	LINC00467	0.789240058	APOO	SNHG3	1

NVL	ZNRD1-AS1	0.87650058	ATXN1	DLEU2	0.552499063	APP	H19	1
PSMD11	H19	0.876370975	ATXN1	KTN1-AS1	0.611604026	APP	LINC00467	1
XPOT	ZNRD1-AS1	0.876288879	ATXN1	MCM3AP-AS1	0.737505738	APP	SNHG5	1
HOXA3	ZNRD1-AS1	0.876191998	ATXN1	RUSC1-AS1	0.837002829	APP	ZNRD1-AS1	1
CEP41	SNHG3	0.875893523	ATXN1	TPT1-AS1	0.897741511	APPL1	H19	1
COBLL1	HCP5	0.875398926	ATXN1	ZNRD1-AS1	0.857287856	APPL1	RUSC1-AS1	1
HAUS1	SNHG3	0.87532753	ATXN7L1	MCM3AP-AS1	0.929413068	APPL1	SNHG5	1
GCNT1	KTN1-AS1	0.875148859	ATXN7L1	TPT1-AS1	0.921134282	APPL1	ZNRD1-AS1	1
MGST1	SNHG3	0.875085002	ATXN7L1	ZNRD1-AS1	0.979766292	AQP3	KTN1-AS1	1
WDR35	H19	0.875066926	AURKB	MCM3AP-AS1	0.743537345	AQP3	MCM3AP-AS1	1
FCHO1	ZNRD1-AS1	0.874404639	AURKB	ZNRD1-AS1	0.591975791	ARAP2	HCP5	1
DPYSL2	KTN1-AS1	0.873119323	B3GNT5	SNHG5	0.890108645	ARAP2	MCM3AP-AS1	1
MSRB2	SNHG5	0.873024782	B4GALT5	RHPN1-AS1	0.828570729	ARF5	HCP5	1
CD47	SNHG3	0.872369755	B4GALT5	SNHG3	0.929412863	ARF5	RHPN1-AS1	1
IMPA2	TP53TG1	0.87217309	B4GALT6	HCP5	0.971501693	ARFGAP3	HCP5	1
PRKACB	HCP5	0.872072529	B4GALT6	MCM3AP-AS1	0.659656924	ARFGAP3	KTN1-AS1	1
FRMD6	SCARNA9	0.871810244	B4GALT6	ZNRD1-AS1	0.57867294	ARFGAP3	MCM3AP-AS1	1
H2AFJ	HCP5	0.871734178	BACE1	H19	0.963836444	ARFGAP3	SNHG3	1
CLEC2D	ZNRD1-AS1	0.871628286	BACE1	MCM3AP-AS1	0.595008892	ARFGEF2	DLEU2	1
CUTC	MCM3AP-AS1	0.871276659	BAIAP2	MCM3AP-AS1	0.623493519	ARFGEF2	H19	1
TBC1D12	KTN1-AS1	0.870935565	BAIAP2	ZNRD1-AS1	0.766597468	ARFGEF2	LINC00467	1
ERP29	SNHG3	0.87081247	BARD1	H19	0.566368696	ARFGEF2	SNHG5	1
CCDC28A	SCARNA9	0.870223883	BAX	H19	0.781705885	ARFGEF2	ZNRD1-AS1	1
FYN	ZNRD1-AS1	0.870173351	BAX	ZNRD1-AS1	0.822871439	ARG2	H19	1
DCTN4	SCARNA9	0.869459103	BAZ1A	RUSC1-AS1	0.632778995	ARG2	ZNRD1-AS1	1
SGK1	HCP5	0.869033837	BAZ1A	TPT1-AS1	0.73646484	ARHGAP10	ZNRD1-AS1	1
PBX3	LINC00467	0.868739315	BAZ2B	KTN1-AS1	0.530582601	ARHGAP11A	SCARNA9	1
PRDM2	MCM3AP-AS1	0.86859476	BAZ2B	MCM3AP-AS1	0.645516424	ARHGAP11A	SNHG3	1
TPP1	LINC00467	0.868199531	BAZ2B	RUSC1-AS1	0.600051771	ARHGAP12	H19	1
ARHGDIB	HCP5	0.868013898	BAZ2B	TPT1-AS1	0.93687667	ARHGAP12	SNHG5	1
HECA	SNHG3	0.867854311	BAZ2B	ZNRD1-AS1	0.756250482	ARHGAP12	ZNRD1-AS1	1
DCUN1D5	RUSC1-AS1	0.867836948	BBS2	KTN1-AS1	0.668262677	ARHGAP18	MCM3AP-AS1	1
VANGL1	HCP5	0.867827649	BBS2	MCM3AP-AS1	0.785056495	ARHGAP18	SNHG3	1
ITGA2	HCP5	0.867614829	BBS2	TPT1-AS1	0.972749275	ARHGAP20	DLEU2	1
OTUD1	HCP5	0.867055112	BBS2	ZNRD1-AS1	0.89240438	ARHGAP26	DLEU2	1
ZNF395	HCP5	0.866733147	BCCIP	H19	0.886038179	ARHGAP26	H19	1
LIPT2	KTN1-AS1	0.866707372	BCL11A	H19	0.667683044	ARHGAP26	SNHG5	1
FSD1L	KTN1-AS1	0.866344919	BCL11A	HCP5	0.769455112	ARHGAP26	TPT1-AS1	1
TNFRSF10D	TPT1-AS1	0.865875754	BCL11A	TP53TG1	0.832307073	ARHGAP26	ZNRD1-AS1	1
PKP4	MCM3AP-AS1	0.865213362	BCL11A	ZNRD1-AS1	0.703811603	ARHGAP32	DLEU2	1
ELOVL6	SNHG3	0.865130117	BCL2	HCP5	0.842665824	ARHGAP32	SNHG5	1
BNIP3L	SNHG3	0.864818398	BCL2L1	DLEU2	0.555663872	ARHGAP32	TPT1-AS1	1
TSKU	TP53TG1	0.864477962	BCL2L11	H19	0.693331787	ARHGAP32	ZNRD1-AS1	1
HGF	SNHG3	0.864171421	BCL9L	KTN1-AS1	0.781705525	ARHGAP6	MCM3AP-AS1	1
AADAT	MCM3AP-AS1	0.863972452	BCL9L	MCM3AP-AS1	0.877243931	ARHGDIB	HCP5	1
KIAA1161	SNHG3	0.863506111	BCL9L	ZNRD1-AS1	0.955957198	ARHGEF10	HCP5	1
CRTAP	RHPN1-AS1	0.863442841	BCOR	DLEU2	0.587967621	ARHGEF10	SNHG3	1
RBM10	H19	0.863087186	BCOR	RUSC1-AS1	0.737534782	ARHGEF2	DLEU2	1
DIXDC1	SNHG3	0.862868088	BDH1	H19	0.507777149	ARHGEF2	TPT1-AS1	1
KLF4	HCP5	0.862781971	BECN1	SNHG3	0.856000092	ARHGEF3	HCP5	1
PPAT	SNHG5	0.862531727	BEND6	H19	0.783334769	ARHGEF3	KTN1-AS1	1

FANCA	H19	0.862383692	BEND7	DLEU2	0.957516453	ARHGEF3	SNHG3	1
PATZ1	HCP5	0.862040202	BEND7	SNHG5	0.542445313	ARID4B	DLEU2	1
MAP6D1	RHPN1-AS1	0.861866141	BIN1	MCM3AP-AS1	0.816136337	ARID4B	H19	1
BNIP3L	HCP5	0.861692327	BLM	HCP5	0.769946473	ARID4B	LINC00467	1
TMEM38B	SCARNA9	0.861608705	BLM	TP53TG1	0.905192084	ARID4B	SNHG5	1
DBP	SNHG3	0.861598334	BLOC1S1	SNHG3	0.560616642	ARID4B	TPT1-AS1	1
FAM160B1	HCP5	0.861293929	BMP4	SNHG5	0.527437386	ARID4B	ZNRD1-AS1	1
CAB39	H19	0.861061273	BMP6	SNHG3	0.868628222	ARID5B	DLEU2	1
HMGA1	SNHG5	0.860450606	BMP8B	HCP5	0.852222073	ARID5B	H19	1
L2HGDH	H19	0.860371912	BMPR1B	H19	0.89575305	ARID5B	RUSC1-AS1	1
KIAA0040	HCP5	0.860322516	BNIP1	RHPN1-AS1	0.770787988	ARID5B	SNHG5	1
PDCD1LG2	H19	0.859960803	BNIP3L	HCP5	0.746315481	ARID5B	TPT1-AS1	1
BEND7	HCP5	0.859876953	BNIP3L	KTN1-AS1	0.878088712	ARID5B	ZNRD1-AS1	1
BUB1B	H19	0.859747195	BNIP3L	MCM3AP-AS1	0.897725514	ARL13B	HCP5	1
FAM46A	SNHG5	0.859411347	BNIP3L	TPT1-AS1	0.685596157	ARL2	HCP5	1
SFXN2	ZNRD1-AS1	0.859048287	BNIP3L	ZNRD1-AS1	0.885956999	ARL2	MCM3AP-AS1	1
STRADB	SNHG5	0.858768416	BRCA1	MCM3AP-AS1	0.664216354	ARL5A	H19	1
SCARB2	HCP5	0.858332585	BRCA1	ZNRD1-AS1	0.500423216	ARL5B	DLEU2	1
CUL4B	HCP5	0.85820996	BRCA2	H19	0.762405243	ARL5B	RUSC1-AS1	1
SPAG9	DLEU2	0.858207021	BRCA2	ZNRD1-AS1	0.78497312	ARL5B	SNHG5	1
PTPN12	ZNRD1-AS1	0.857903936	BRD2	MCM3AP-AS1	0.668924555	ARL5B	TPT1-AS1	1
HOMER1	SNHG5	0.857244595	BRD2	ZNRD1-AS1	0.765786351	ARL5B	ZNRD1-AS1	1
AKIRIN1	HCP5	0.856728521	BRE	HCP5	0.881838309	ARL6IP5	H19	1
SUSD3	SNHG5	0.856646019	BRI3BP	H19	0.983313679	ARL6IP5	RUSC1-AS1	1
LPCAT4	SNHG3	0.856162917	BRIP1	TP53TG1	0.934151419	ARL6IP5	SNHG5	1
CPNE8	RUSC1-AS1	0.855902376	BRPF3	RUSC1-AS1	0.590192188	ARL6IP5	ZNRD1-AS1	1
TFPI	RHPN1-AS1	0.855898685	BRPF3	ZNRD1-AS1	0.504421018	ARL6IP6	HCP5	1
DRAM1	SNHG3	0.855616203	BSDC1	SNHG3	0.599102185	ARL6IP6	MCM3AP-AS1	1
NUDCD3	SNHG3	0.855582847	BTAF1	RUSC1-AS1	0.847165934	ARL6IP6	SCARNA9	1
HOXA4	ZNRD1-AS1	0.855519682	BTBD2	TP53TG1	0.624348451	ARL6IP6	SNHG3	1
FCHO2	RUSC1-AS1	0.855334011	BTBD8	H19	0.59355386	ARMCX2	MCM3AP-AS1	1
FOXN2	SNHG5	0.854842014	BTBD8	HCP5	0.726225065	ARMCX6	RHPN1-AS1	1
USP51	SNHG5	0.854282886	BTBD8	ZNRD1-AS1	0.822710982	ARPC5L	HCP5	1
GRK5	RUSC1-AS1	0.854167241	BTG2	SNHG5	0.762608018	ARPC5L	RHPN1-AS1	1
INTS6	HCP5	0.854090016	BTG3	SNHG5	0.778541193	ARPC5L	SNHG3	1
TCF7L2	RUSC1-AS1	0.853910943	BTN3A1	HCP5	0.838052537	ARRDC4	HCP5	1
SEMA6D	HCP5	0.85340917	BTN3A1	KTN1-AS1	0.861800615	ARRDC4	KTN1-AS1	1
GPR126	KTN1-AS1	0.85337092	BTN3A1	LINC00467	0.769040579	ARRDC4	MCM3AP-AS1	1
GALK1	KTN1-AS1	0.853304146	BTN3A1	MCM3AP-AS1	0.85606299	ARRDC4	TP53TG1	1
MLF1	HCP5	0.85306036	BTN3A1	ZNRD1-AS1	0.815973345	ARSK	TP53TG1	1
NOG	SNHG3	0.852914764	BTN3A2	H19	0.690495385	ASAH1	HCP5	1
ARHGEF2	DLEU2	0.852482884	BTN3A2	HCP5	0.696822664	ASAH1	KTN1-AS1	1
SAP30L	SNHG5	0.852454428	BTN3A2	KTN1-AS1	0.945558561	ASAP2	MCM3AP-AS1	1
ARMCX6	RHPN1-AS1	0.852379739	BTN3A2	LINC00467	0.972068342	ASB1	HCP5	1
ZKSCAN1	MCM3AP-AS1	0.85213511	BTN3A2	ZNRD1-AS1	0.768032227	ASB1	MCM3AP-AS1	1
RBM47	KTN1-AS1	0.851534686	BTN3A3	H19	0.53029043	ASB13	SCARNA9	1
GPAM	TP53TG1	0.851473238	BTN3A3	HCP5	0.825650426	ASB9	H19	1
LTV1	ZNRD1-AS1	0.851302691	BTN3A3	KTN1-AS1	0.908631345	ASB9	ZNRD1-AS1	1
KLF3	HCP5	0.851177483	BTN3A3	MCM3AP-AS1	0.853384797	ASCC3	DLEU2	1
NPEPL1	ZNRD1-AS1	0.850837797	BTN3A3	ZNRD1-AS1	0.755165347	ASCC3	SNHG5	1
RASGEF1A	HCP5	0.850674905	BUB1	H19	0.818851965	ASCC3	TPT1-AS1	1

PLK4	H19	0.85062845	BUB1	HCP5	0.581842954	ASCC3	ZNRD1-AS1	1
ATXN1	HCP5	0.849821046	BUB1B	H19	0.893317073	ASF1B	HCP5	1
TRIT1	MCM3AP-AS1	0.849535854	BUB3	SNHG3	0.928607096	ASIC1	HCP5	1
SLC5A3	SNHG5	0.848434011	BUB3	SNHG5	0.538639576	ASPH	DLEU2	1
TGFBR2	SNHG3	0.847532324	C10orf10	LINC00467	0.506328387	ASPH	H19	1
TSEN15	SNHG3	0.847179926	C11orf49	LINC00467	0.612061307	ASPH	RUSC1-AS1	1
CHMP4C	DLEU2	0.846689894	C12orf5	H19	0.858564981	ASPH	SNHG5	1
CPNE8	MCM3AP-AS1	0.845898778	C12orf5	HCP5	0.514153302	ASPH	TPT1-AS1	1
B4GALT6	SCARNA9	0.84585817	C12orf5	ZNRD1-AS1	0.685504256	ASPH	ZNRD1-AS1	1
PLS1	SNHG5	0.845609193	C14orf159	H19	0.872949942	ASPHD1	HCP5	1
KIAA1211	H19	0.845540545	C16orf45	H19	0.686429539	ATAD2	HCP5	1
ZNF652	SNHG3	0.845402174	C16orf70	H19	0.766259357	ATAD2	SCARNA9	1
FKBP11	ZNRD1-AS1	0.84539685	C16orf70	HCP5	0.752399923	ATAD2	SNHG3	1
ATP13A3	H19	0.844808361	C16orf70	LINC00467	0.988280928	ATAD2B	DLEU2	1
CCDC82	ZNRD1-AS1	0.84442973	C16orf70	MCM3AP-AS1	0.645147339	ATAD2B	H19	1
CBX5	SNHG3	0.843736998	C16orf72	H19	0.718297667	ATAD2B	SNHG5	1
MRPS23	MCM3AP-AS1	0.843274141	C16orf72	KTN1-AS1	0.983917461	ATAD2B	TPT1-AS1	1
ARHGAP32	MCM3AP-AS1	0.843022898	C16orf72	LINC00467	0.889501843	ATAD5	MCM3AP-AS1	1
SCARB2	SNHG3	0.842518296	C16orf72	MCM3AP-AS1	0.94642733	ATF5	RHPN1-AS1	1
FASTKD3	MCM3AP-AS1	0.841726907	C16orf72	ZNRD1-AS1	0.865790795	ATG14	H19	1
WDR81	HCP5	0.841312457	C16orf87	LINC00467	0.712046896	ATG14	SNHG5	1
AMOT	SNHG3	0.841259465	C16orf87	MCM3AP-AS1	0.998627022	ATG14	ZNRD1-AS1	1
CLEC2D	MCM3AP-AS1	0.841208981	C16orf87	ZNRD1-AS1	0.988345022	ATG5	DLEU2	1
ASPH	HCP5	0.841110244	C18orf54	H19	0.816455374	ATG5	H19	1
DRAM1	RUSC1-AS1	0.840551743	C18orf54	HCP5	0.693014045	ATG5	TPT1-AS1	1
RCOR1	MCM3AP-AS1	0.840297413	C18orf54	KTN1-AS1	0.679980377	ATG5	ZNRD1-AS1	1
CENPA	TPT1-AS1	0.840266364	C1S	SNHG5	0.788574781	ATL2	HCP5	1
GPSM2	HCP5	0.840001301	C1orf112	H19	0.634807521	ATL2	MCM3AP-AS1	1
ANKRA2	TPT1-AS1	0.839782995	C1orf21	H19	0.627536226	ATL2	SCARNA9	1
RNF150	RUSC1-AS1	0.839403621	C1orf21	HCP5	0.862035625	ATL2	SNHG3	1
ZNF22	HCP5	0.839189239	C1orf21	LINC00467	0.965939856	ATL2	TP53TG1	1
OSBPL10	HCP5	0.837971424	C1orf21	MCM3AP-AS1	0.665628927	ATM	DLEU2	1
TOB1	HCP5	0.83797035	C1orf21	ZNRD1-AS1	0.518913189	ATM	H19	1
STK17B	SCARNA9	0.837939698	C1orf74	H19	0.767830947	ATM	LINC00467	1
ERLIN2	SNHG3	0.837681398	C1orf74	HCP5	0.72502941	ATM	SNHG5	1
HSPD1	SNHG5	0.837154018	C1orf74	TP53TG1	0.980495386	ATM	TPT1-AS1	1
ZNF217	SCARNA9	0.837147088	C20orf194	MCM3AP-AS1	0.559587432	ATP13A3	H19	1
CCDC71L	RUSC1-AS1	0.83702073	C20orf194	ZNRD1-AS1	0.648321177	ATP13A3	ZNRD1-AS1	1
XPO7	SNHG3	0.836561066	C2CD2	ZNRD1-AS1	0.883332595	ATP1B1	HCP5	1
MAP7	MCM3AP-AS1	0.836112887	C5	H19	0.984262456	ATP1B1	MCM3AP-AS1	1
SEH1L	SNHG3	0.835850815	C5orf15	SNHG5	0.764248209	ATP1B1	SNHG3	1
HRSP12	KTN1-AS1	0.835550316	C5orf30	HCP5	0.954628909	ATP1B1	TP53TG1	1
FOSL2	SNHG3	0.835542085	C5orf30	KTN1-AS1	0.719665263	ATP2A2	HCP5	1
KIAA0895	RUSC1-AS1	0.835246859	C5orf30	LINC00467	0.884074454	ATP2A2	MCM3AP-AS1	1
MEF2A	MCM3AP-AS1	0.834844487	C5orf30	MCM3AP-AS1	0.639319264	ATP2A2	SCARNA9	1
GLCCI1	KTN1-AS1	0.834717149	C5orf30	ZNRD1-AS1	0.519670587	ATP2A2	SNHG3	1
TBC1D12	TP53TG1	0.83469895	C6orf211	SNHG3	0.649666623	ATP2B1	DLEU2	1
HS6ST2	SCARNA9	0.834687014	C9orf40	H19	0.636811724	ATP2B1	H19	1
NEFH	H19	0.8344833	C9orf40	HCP5	0.811570802	ATP2B1	LINC00467	1
EBF1	SNHG3	0.834440866	CA2	DLEU2	0.581232833	ATP2B1	RUSC1-AS1	1
MRPL37	KTN1-AS1	0.834189286	CA2	SNHG5	0.970217631	ATP2B1	SNHG5	1

ITPR1	ZNRD1-AS1	0.833965878	CABLES2	HCP5	0.770143509	ATP2B1	TPT1-AS1	1
ADAM19	SNHG5	0.833569501	CAD	LINC00467	0.990526067	ATP2B1	ZNRD1-AS1	1
DFFB	SNHG5	0.833232386	CADM1	SNHG3	0.79467466	ATP2B4	H19	1
PTDSS1	SNHG5	0.832909166	CADM1	SNHG5	0.699017656	ATP2B4	SNHG5	1
MELK	H19	0.832367537	CALD1	HCP5	0.985622187	ATP2B4	ZNRD1-AS1	1
CDYL2	SNHG3	0.831722268	CALD1	LINC00467	0.696494331	ATP5A1	KTN1-AS1	1
SLC4A4	SNHG5	0.831577317	CALU	SNHG3	0.960495951	ATP6V0E2	KTN1-AS1	1
ATG14	ZNRD1-AS1	0.830938004	CALU	SNHG5	0.75791971	ATP6V0E2	MCM3AP-AS1	1
MYBL2	H19	0.829955982	CAMK1D	SCARNA9	0.514010335	ATP6V0E2	RHPN1-AS1	1
PARP16	RUSC1-AS1	0.829815541	CAMK1D	SNHG5	0.669289319	ATP7B	HCP5	1
C6orf211	SNHG3	0.829773977	CAMK2D	RHPN1-AS1	0.90274919	ATP8A1	MCM3AP-AS1	1
SIDT1	SNHG5	0.829577012	CAMK2D	SNHG3	0.914553516	ATP8A1	TP53TG1	1
SRSF7	TPT1-AS1	0.829558681	CAMK2G	SNHG3	0.794852837	ATPAF1	LINC00467	1
ZNF652	HCP5	0.829255596	CAMSAP2	SNHG5	0.685505199	ATPAF1	RUSC1-AS1	1
USP46	KTN1-AS1	0.829146083	CARHSP1	H19	0.767126267	ATPAF1	SNHG5	1
GLCCI1	SNHG3	0.829092251	CARS	TPT1-AS1	0.863417407	ATXN1	HCP5	1
DENND4A	HCP5	0.829004882	CASK	H19	0.50932458	ATXN1	KTN1-AS1	1
HAUS8	H19	0.828946173	CASK	SNHG3	0.705692187	ATXN1	MCM3AP-AS1	1
TRUB1	LINC00467	0.828530402	CASP2	H19	0.576932944	ATXN1	SNHG3	1
ANKRA2	ZNRD1-AS1	0.828382769	CASP2	HCP5	0.873516997	ATXN7L1	DLEU2	1
STOM	HCP5	0.828334543	CASP2	MCM3AP-AS1	0.735127565	ATXN7L1	H19	1
ARHGAP12	MCM3AP-AS1	0.828017209	CASP2	ZNRD1-AS1	0.606284653	ATXN7L1	LINC00467	1
UBXN2B	MCM3AP-AS1	0.827423177	CAST	H19	0.589627253	ATXN7L1	TPT1-AS1	1
FAM65B	HCP5	0.826397573	CAST	MCM3AP-AS1	0.669438387	ATXN7L1	ZNRD1-AS1	1
MBOAT2	LINC00467	0.826363551	CAST	TPT1-AS1	0.800061731	AURKA	SCARNA9	1
PAICS	ZNRD1-AS1	0.826111348	CAST	ZNRD1-AS1	0.673291175	AURKB	MCM3AP-AS1	1
IVNS1ABP	MCM3AP-AS1	0.825700744	CASZ1	ZNRD1-AS1	0.687383053	AVL9	MCM3AP-AS1	1
PAM	SNHG5	0.825464801	CAT	TP53TG1	0.936893386	B3GNT5	HCP5	1
MYD88	LINC00467	0.82538817	CBX2	H19	0.70881862	B4GALT4	HCP5	1
THAP11	SCARNA9	0.825271982	CBX2	HCP5	0.703403755	B4GALT4	KTN1-AS1	1
TRIO	ZNRD1-AS1	0.824909796	CBX2	LINC00467	0.981231122	B4GALT4	MCM3AP-AS1	1
KBTBD6	KTN1-AS1	0.824863622	CBX2	MCM3AP-AS1	0.858915105	B4GALT5	HCP5	1
S1PR1	HCP5	0.824749895	CBX2	ZNRD1-AS1	0.740056822	B4GALT5	MCM3AP-AS1	1
CD79A	RHPN1-AS1	0.824694665	CBX5	H19	0.740019665	B4GALT5	RHPN1-AS1	1
HOXA10	SNHG3	0.824693299	CBX5	HCP5	0.75238094	B4GALT5	SNHG3	1
NAA16	ZNRD1-AS1	0.824530361	CBX5	KTN1-AS1	0.847364625	B4GALT6	HCP5	1
ZHX2	HCP5	0.824138181	CBX5	LINC00467	0.997664336	B4GALT6	MCM3AP-AS1	1
PTK2B	KTN1-AS1	0.824055491	CBX5	MCM3AP-AS1	0.74850206	B4GALT6	SCARNA9	1
STK17B	DLEU2	0.823875348	CBX5	ZNRD1-AS1	0.603037746	B4GALT6	SNHG3	1
PLAG1	H19	0.823398579	CBX7	ZNRD1-AS1	0.574118323	BACE1	HCP5	1
UQCR10	KTN1-AS1	0.823251722	CCDC138	H19	0.652218685	BACE1	MCM3AP-AS1	1
CD44	LINC00467	0.823034901	CCDC138	HCP5	0.849407517	BACE1	SNHG3	1
TMEM39B	KTN1-AS1	0.822916745	CCDC15	H19	0.80670221	BACH2	HCP5	1
SBF2	MCM3AP-AS1	0.822730188	CCDC28A	KTN1-AS1	0.618270921	BACH2	MCM3AP-AS1	1
COBLL1	SNHG3	0.822710618	CCDC34	KTN1-AS1	0.632485252	BACH2	TP53TG1	1
TTC30B	HCP5	0.822333978	CCDC34	MCM3AP-AS1	0.506567906	BAG2	RUSC1-AS1	1
AP4E1	DLEU2	0.822309869	CCDC43	H19	0.939315019	BAG2	ZNRD1-AS1	1
BTN3A1	KTN1-AS1	0.822270176	CCDC43	LINC00467	0.665494963	BAG4	MCM3AP-AS1	1
PREX1	HCP5	0.821981165	CCDC53	TP53TG1	0.99324669	BAG4	SNHG3	1
TNFRSF10B	MCM3AP-AS1	0.821827772	CCDC71L	HCP5	0.998596841	BAIAP2	HCP5	1
DSC2	SNHG5	0.821683194	CCDC71L	TP53TG1	0.601836384	BAIAP2	MCM3AP-AS1	1

SMAD5	KTN1-AS1	0.82145515	CCDC8	H19	0.760244465	BARD1	MCM3AP-AS1	1
KIAA0101	H19	0.821370417	CCDC8	MCM3AP-AS1	0.601409709	BARD1	SCARNA9	1
SLC46A3	MCM3AP-AS1	0.821067519	CCDC85C	SNHG3	0.613789357	BARD1	SNHG3	1
YPEL2	HCP5	0.820915078	CCDC85C	SNHG5	0.759805398	BAX	HCP5	1
UBE2E2	SNHG5	0.820714003	CCM2	KTN1-AS1	0.863410398	BAZ1A	DLEU2	1
HS2ST1	KTN1-AS1	0.820540354	CCM2	MCM3AP-AS1	0.772870012	BAZ1A	H19	1
CYB5D2	SNHG5	0.820112588	CCM2	ZNRD1-AS1	0.636242575	BAZ1A	RUSC1-AS1	1
PAICS	H19	0.81998162	CCNA2	H19	0.752860704	BAZ1A	TPT1-AS1	1
ARAP2	HCP5	0.81991806	CCNA2	HCP5	0.560448002	BAZ1A	ZNRD1-AS1	1
ZBTB18	HCP5	0.819796975	CCNJL	SNHG3	0.930998224	BAZ2B	DLEU2	1
SMAD5	SNHG5	0.81958794	CCNJL	SNHG5	0.668153748	BAZ2B	H19	1
HIVEP1	DLEU2	0.819577763	CCP110	H19	0.590735614	BAZ2B	RUSC1-AS1	1
GPR126	SNHG3	0.819326208	CCP110	HCP5	0.841143953	BAZ2B	SNHG5	1
URB2	SCARNA9	0.819234578	CCP110	ZNRD1-AS1	0.671606603	BAZ2B	TPT1-AS1	1
FAM134A	KTN1-AS1	0.819219562	CCPG1	TPT1-AS1	0.741520105	BAZ2B	ZNRD1-AS1	1
C16orf87	HCP5	0.818870392	CCT2	SNHG3	0.928069297	BBS2	KTN1-AS1	1
ASAH1	KTN1-AS1	0.818868281	CCT2	SNHG5	0.593881347	BBS2	MCM3AP-AS1	1
RAB12	HCP5	0.81852389	CCT6A	SNHG5	0.690730667	BBS9	MCM3AP-AS1	1
ATP6V0E2	KTN1-AS1	0.818508861	CCT8	SNHG3	0.949030424	BCAT2	MCM3AP-AS1	1
MCM7	H19	0.818291124	CD164	HCP5	0.601905766	BCCIP	MCM3AP-AS1	1
MECOM	SNHG3	0.818154653	CD164	SCARNA9	0.816836226	BCL11A	DLEU2	1
NR4A1	DLEU2	0.818105146	CD24	SNHG3	0.93542664	BCL11A	H19	1
FAM117B	HCP5	0.817359773	CD276	H19	0.849900659	BCL11A	SNHG5	1
FANCM	H19	0.817334582	CD276	ZNRD1-AS1	0.537308694	BCL11A	TPT1-AS1	1
AKAP1	H19	0.817094216	CD302	H19	0.56100496	BCL11A	ZNRD1-AS1	1
BRCA2	H19	0.817063088	CD302	KTN1-AS1	0.837080435	BCL2	HCP5	1
RUNDC3B	SNHG3	0.81696804	CD302	LINC00467	0.953971357	BCL2	KTN1-AS1	1
LSM5	RUSC1-AS1	0.816862167	CD302	MCM3AP-AS1	0.758137852	BCL2	MCM3AP-AS1	1
ACOT9	H19	0.816668107	CD44	SNHG3	0.970429799	BCL2	RHPN1-AS1	1
GPR157	H19	0.816630434	CD47	SNHG3	0.885704181	BCL2	SNHG3	1
EPS8	HCP5	0.815934976	CD59	SCARNA9	0.636660137	BCL2L1	DLEU2	1
ZDHHC17	RUSC1-AS1	0.815668058	CD69	SNHG5	0.789504981	BCL2L1	TPT1-AS1	1
NAA15	SNHG3	0.81552909	CD80	SNHG3	0.858313204	BCL2L11	HCP5	1
BRE	SNHG3	0.815268188	CD83	DLEU2	0.732303376	BCL2L11	MCM3AP-AS1	1
GRK5	HCP5	0.815222902	CD83	SNHG3	0.566708986	BCL2L12	MCM3AP-AS1	1
NRM	SNHG5	0.815078289	CD99L2	RHPN1-AS1	0.925873762	BCL6	DLEU2	1
GLCCI1	HCP5	0.814976186	CDADC1	KTN1-AS1	0.942281138	BCL6	H19	1
KANK1	SNHG3	0.814327554	CDADC1	MCM3AP-AS1	0.907572782	BCL6	SNHG5	1
ZNF607	SNHG5	0.814098494	CDADC1	ZNRD1-AS1	0.831472256	BCL6	TPT1-AS1	1
SRRT	H19	0.81405798	CDC20	LINC00467	0.924875114	BCL6	ZNRD1-AS1	1
CREBRF	HCP5	0.813989921	CDC23	H19	0.570786889	BCL9L	ZNRD1-AS1	1
RNF24	SNHG3	0.813891228	CDC42EP2	SNHG5	0.723882631	BCOR	HCP5	1
NABP1	HCP5	0.813866367	CDC7	HCP5	0.929317	BCOR	SCARNA9	1
PRDM1	SNHG5	0.813691589	CDCA2	TP53TG1	0.970518478	BCOR	SNHG3	1
PRKRA	RUSC1-AS1	0.813541085	CDCA3	TP53TG1	0.82672043	BCOR	TP53TG1	1
3-Sep	SNHG5	0.81214828	CDCA5	H19	0.650895313	BDH1	HCP5	1
DAPP1	DLEU2	0.811586356	CDCA5	LINC00467	0.594292403	BECN1	HCP5	1
BCL11A	HCP5	0.811155153	CDCA7	H19	0.697924199	BECN1	KTN1-AS1	1
TRIM59	SNHG3	0.810941678	CDCA7	HCP5	0.789244711	BECN1	MCM3AP-AS1	1
RABGAP1	MCM3AP-AS1	0.810696164	CDCA7	ZNRD1-AS1	0.60928709	BECN1	SCARNA9	1
FMNL2	MCM3AP-AS1	0.810685766	CDH1	SNHG3	0.789596717	BECN1	SNHG3	1

SLC35D2	SNHG3	0.810527455	CDH1	SNHG5	0.707559936	BEND6	MCM3AP-AS1	1
FBXW7	MCM3AP-AS1	0.810489544	CDH2	HCP5	0.798605473	BEND7	HCP5	1
EML4	ZNRD1-AS1	0.810427847	CDIPT	H19	0.838435484	BEND7	MCM3AP-AS1	1
ATP1B1	HCP5	0.81041265	CDIPT	HCP5	0.642402806	BEND7	TP53TG1	1
MRPS35	HCP5	0.810205642	CDIPT	MCM3AP-AS1	0.726288674	BEST1	DLEU2	1
ZFP90	SNHG3	0.809926438	CDIPT	ZNRD1-AS1	0.571380352	BHLHE41	HCP5	1
NME4	SNHG3	0.809882759	CDK1	H19	0.692887594	BIN1	MCM3AP-AS1	1
FAT4	KTN1-AS1	0.808280525	CDK13	H19	0.566750749	BIRC2	H19	1
FAM134B	KTN1-AS1	0.808061113	CDK13	LINC00467	0.728492622	BIRC5	MCM3AP-AS1	1
PLEKHB1	RHPN1-AS1	0.807678535	CDK13	MCM3AP-AS1	0.979463255	BIRC5	SNHG3	1
CD164	SNHG3	0.807338954	CDK13	ZNRD1-AS1	0.953133237	BLM	HCP5	1
RBL1	H19	0.807024207	CDK14	H19	0.530098376	BLM	TP53TG1	1
ARHGEF3	SNHG3	0.806901609	CDK14	TP53TG1	0.779251156	BLOC1S1	SNHG3	1
LHX6	HCP5	0.806534722	CDK14	ZNRD1-AS1	0.660954377	BMF	HCP5	1
CSNK1G1	SNHG3	0.80561275	CDK18	H19	0.64286789	BMF	KTN1-AS1	1
ATG4C	SCARNA9	0.805464411	CDK18	HCP5	0.640042043	BMF	MCM3AP-AS1	1
MIS18BP1	DLEU2	0.804850689	CDK18	LINC00467	0.929661199	BMP1	H19	1
COX11	RHPN1-AS1	0.804164558	CDK4	H19	0.610647213	BMP1	SNHG5	1
SAMD4A	HCP5	0.802772928	CDK6	H19	0.530855773	BMP4	MCM3AP-AS1	1
CRBN	HCP5	0.802616125	CDK6	HCP5	0.869448355	BMP6	H19	1
DPYSL3	SNHG3	0.80261511	CDK6	MCM3AP-AS1	0.790607831	BMP6	RUSC1-AS1	1
BCCIP	ZNRD1-AS1	0.802550371	CDK6	TP53TG1	0.772193236	BMP6	ZNRD1-AS1	1
BTN3A1	SNHG3	0.802533541	CDK6	ZNRD1-AS1	0.678803558	BMP8B	HCP5	1
ZNF652	KTN1-AS1	0.802243635	CDKN1B	H19	0.902034909	BMPR1B	SCARNA9	1
KLF3	KTN1-AS1	0.802231242	CDKN1B	KTN1-AS1	0.869665122	BNIP1	SNHG5	1
TBC1D12	SNHG3	0.802209491	CDKN1B	MCM3AP-AS1	0.779309953	BNIP3L	HCP5	1
CCP110	ZNRD1-AS1	0.802202512	CDKN3	H19	0.684903987	BNIP3L	KTN1-AS1	1
CBX5	HCP5	0.802163695	CDR2	SNHG3	0.930007952	BNIP3L	MCM3AP-AS1	1
PRRG1	SNHG5	0.802149657	CEBPG	H19	0.72840155	BNIP3L	SNHG3	1
MSRB3	SNHG3	0.802083374	CEBPG	ZNRD1-AS1	0.870625512	BOLA3	MCM3AP-AS1	1
LYRM1	HCP5	0.802073943	CENPF	H19	0.55077999	BORA	KTN1-AS1	1
ASCC3	KTN1-AS1	0.801929402	CENPF	MCM3AP-AS1	0.659619919	BORA	MCM3AP-AS1	1
WDR47	ZNRD1-AS1	0.801713453	CENPF	ZNRD1-AS1	0.673187098	BRCA1	MCM3AP-AS1	1
ZHX3	ZNRD1-AS1	0.80170257	CENPK	H19	0.780850321	BRD2	MCM3AP-AS1	1
RBM34	DLEU2	0.801514848	CENPM	KTN1-AS1	0.681098186	BRD2	SNHG3	1
WDR43	MCM3AP-AS1	0.801252017	CENPO	H19	0.88234214	BRD2	TP53TG1	1
ABHD4	LINC00467	0.800659937	CEP128	H19	0.994998187	BRE	HCP5	1
PRDM1	KTN1-AS1	0.800543037	CEP135	SNHG3	0.69762605	BRE	MCM3AP-AS1	1
ZXDB	RUSC1-AS1	0.800497235	CEP55	H19	0.925647762	BRE	SNHG3	1
NAP1L2	HCP5	0.800262533	CEP68	HCP5	0.631646183	BRI3BP	HCP5	1
KLHL23	SNHG3	0.800252409	CEP68	LINC00467	0.834768318	BRI3BP	SCARNA9	1
GLCCI1	TP53TG1	0.800231545	CEP68	ZNRD1-AS1	0.93202153	BRIP1	TP53TG1	1
ALDH3B1	MCM3AP-AS1	0.800174224	CERCAM	H19	0.59839888	BRPF3	RUSC1-AS1	1
KLF3	SNHG3	0.800027657	CERCAM	HCP5	0.857986114	BRPF3	SNHG5	1
SNTB1	SNHG5	0.799752128	CERCAM	ZNRD1-AS1	0.616984392	BRPF3	ZNRD1-AS1	1
RNF19A	ZNRD1-AS1	0.799688115	CERK	HCP5	0.536033278	BSCL2	HCP5	1
AMOT	HCP5	0.799282597	CHAF1A	ZNRD1-AS1	0.979709391	BSDC1	H19	1
MRTO4	H19	0.799188907	CHCHD7	SNHG3	0.689634687	BSDC1	RUSC1-AS1	1
TIMM50	LINC00467	0.799070179	CHD2	MCM3AP-AS1	0.79450678	BSDC1	SNHG5	1
GCNT2	TPT1-AS1	0.798988623	CHD2	TPT1-AS1	0.984295707	BSDC1	ZNRD1-AS1	1
SSBP2	RUSC1-AS1	0.798981382	CHD2	ZNRD1-AS1	0.869690823	BTAF1	H19	1

DIS3L	ZNRD1-AS1	0.798911431	CHD5	H19	0.775012112	BTAF1	RUSC1-AS1	1
ITM2B	SNHG3	0.79861472	CHD5	MCM3AP-AS1	0.914503602	BTAF1	ZNRD1-AS1	1
E2F7	H19	0.798353209	CHD5	ZNRD1-AS1	0.83413875	BTBD2	SNHG3	1
B4GALT4	HCP5	0.798031212	CHD7	SNHG3	0.919701916	BTBD2	TP53TG1	1
FAM198B	SNHG5	0.797975368	CHM	SNHG3	0.919277872	BTBD8	HCP5	1
MIS18BP1	TPT1-AS1	0.797654716	CHM	SNHG5	0.733485384	BTG1	H19	1
TUBGCP4	SNHG3	0.797566688	CHMP4A	RHPN1-AS1	0.814855145	BTG1	SNHG5	1
ISCU	SCARNA9	0.79754757	CHMP4C	MCM3AP-AS1	0.978784728	BTG1	ZNRD1-AS1	1
MYOZ3	SNHG5	0.797020405	CHMP4C	ZNRD1-AS1	0.931175392	BTG2	HCP5	1
SPRED1	SNHG3	0.79649412	CHRNA5	SNHG5	0.780294221	BTG3	DLEU2	1
ANAPC10	DLEU2	0.796200057	CHUK	SNHG3	0.978578073	BTG3	H19	1
ITGA2	RUSC1-AS1	0.796022985	CHUK	SNHG5	0.793542095	BTG3	LINC00467	1
CREB5	RUSC1-AS1	0.795893727	CIDEB	MCM3AP-AS1	0.920736516	BTG3	SNHG5	1
KCTD15	SNHG3	0.795594774	CIDEB	ZNRD1-AS1	0.871188775	BTG3	TPT1-AS1	1
IVNS1ABP	RUSC1-AS1	0.795309149	CISH	DLEU2	0.90159119	BTG3	ZNRD1-AS1	1
MECOM	RUSC1-AS1	0.795299748	CIT	H19	0.874964912	BTN3A1	H19	1
COPB1	DLEU2	0.794845364	CIT	MCM3AP-AS1	0.832773101	BTN3A1	LINC00467	1
SDCCAG8	ZNRD1-AS1	0.794364643	CIT	TPT1-AS1	0.589854703	BTN3A1	ZNRD1-AS1	1
AP3S1	HCP5	0.794318494	CIT	ZNRD1-AS1	0.719458437	BTN3A2	HCP5	1
ADRA2A	H19	0.794072793	CKAP2	H19	0.711495149	BTN3A2	KTN1-AS1	1
SSPN	RHPN1-AS1	0.79398059	CKAP2	HCP5	0.629657583	BTN3A2	SNHG3	1
ESYT2	SCARNA9	0.793327718	CKB	MCM3AP-AS1	0.963158236	BTN3A3	H19	1
FLI1	SNHG5	0.793275173	CKB	ZNRD1-AS1	0.895031471	BTN3A3	ZNRD1-AS1	1
MECOM	HCP5	0.792908864	CKS2	TP53TG1	0.711036413	BUB1	HCP5	1
FCHO2	HCP5	0.792380374	CLDND1	SNHG3	0.997034504	BUB1	SNHG3	1
FAM133A	KTN1-AS1	0.792334761	CLDND1	SNHG5	0.711305231	BUB1B	SCARNA9	1
RPL32	TP53TG1	0.792265493	CLEC2D	MCM3AP-AS1	0.792755126	BUB3	DLEU2	1
WBP4	ZNRD1-AS1	0.792218234	CLEC2D	ZNRD1-AS1	0.873270778	BUB3	H19	1
ANGPT1	MCM3AP-AS1	0.792097194	CLGN	TP53TG1	0.983578297	BUB3	LINC00467	1
SLC11A2	H19	0.792059182	CLIC4	SCARNA9	0.581388938	BUB3	RUSC1-AS1	1
LRBA	SNHG3	0.792059153	CLIC4	SNHG3	0.790187719	BUB3	SNHG5	1
PPA1	HCP5	0.791989914	CLIC4	SNHG5	0.986122299	BUB3	TPT1-AS1	1
GM2A	TP53TG1	0.791817987	CLIP1	MCM3AP-AS1	0.637036801	C10orf10	LINC00467	1
STX7	RUSC1-AS1	0.791577696	CLIP1	ZNRD1-AS1	0.737960612	C11orf49	LINC00467	1
HMGA1	SNHG3	0.791213381	CLIP4	DLEU2	0.902146391	C11orf73	H19	1
ZBTB38	HCP5	0.79108453	CLIP4	SNHG5	0.88009835	C11orf73	ZNRD1-AS1	1
STK17A	RUSC1-AS1	0.79066385	CLN6	H19	0.795393268	C11orf96	H19	1
RAVER2	SNHG3	0.790445106	CLN6	MCM3AP-AS1	0.619095623	C11orf96	ZNRD1-AS1	1
APPL1	SNHG3	0.790407196	CLSPN	MCM3AP-AS1	0.816655421	C12orf4	HCP5	1
CCT5	H19	0.790405929	CLSPN	TP53TG1	0.8751915	C12orf4	MCM3AP-AS1	1
CEBPG	HCP5	0.789898883	CLSPN	ZNRD1-AS1	0.683243281	C12orf5	HCP5	1
CYBRD1	KTN1-AS1	0.789838399	CLU	MCM3AP-AS1	0.618313012	C12orf5	SCARNA9	1
RAD51AP1	H19	0.789805963	CNKSR1	ZNRD1-AS1	0.834363325	C14orf159	H19	1
BCL9L	HCP5	0.789500718	CNKSR3	HCP5	0.664076516	C14orf2	H19	1
SMARCA1	DLEU2	0.789361575	CNKSR3	MCM3AP-AS1	0.846638845	C14orf2	ZNRD1-AS1	1
ZNF326	TPT1-AS1	0.78935051	CNKSR3	ZNRD1-AS1	0.721559918	C15orf39	MCM3AP-AS1	1
STK24	LINC00467	0.78902972	CNNM3	H19	0.668907626	C16orf59	HCP5	1
KIF3B	H19	0.788893715	CNNM3	HCP5	0.835861958	C16orf70	HCP5	1
EMP1	HCP5	0.788878103	CNOT6L	HCP5	0.921522736	C16orf70	MCM3AP-AS1	1
UBE2K	HCP5	0.788410877	COBLL1	HCP5	0.916618584	C16orf70	SNHG3	1
KIF3B	ZNRD1-AS1	0.788403963	COBLL1	KTN1-AS1	0.806923643	C16orf72	H19	1

RALGPS2	SNHG3	0.788166071	COBLL1	MCM3AP-AS1	0.736978372	C16orf72	LINC00467	1
TFPI2	KTN1-AS1	0.788112868	COL15A1	SNHG3	0.850090861	C16orf72	SNHG5	1
EIF2B2	SCARNA9	0.788069102	COL24A1	ZNRD1-AS1	0.921225769	C16orf72	ZNRD1-AS1	1
CROT	RUSC1-AS1	0.787965772	COTL1	SNHG3	0.757771371	C16orf87	HCP5	1
MRPS23	ZNRD1-AS1	0.787903652	CPA3	H19	0.543330431	C16orf87	MCM3AP-AS1	1
MAMDC2	MCM3AP-AS1	0.78749513	CPA3	HCP5	0.913165578	C16orf87	SNHG3	1
LLGL1	SNHG3	0.787387948	CPA3	TP53TG1	0.858920909	C17orf49	HCP5	1
CLIC4	SNHG3	0.787290704	CPEB2	DLEU2	0.589855979	C17orf53	SNHG3	1
KIF5C	SNHG5	0.786564712	CPEB2	RHPN1-AS1	0.5709295	C17orf75	HCP5	1
CARD16	SNHG3	0.786441651	CPEB2	SNHG5	0.787975221	C18orf54	H19	1
ORC1	H19	0.786412475	CPEB4	DLEU2	0.514223747	C18orf54	ZNRD1-AS1	1
PRMT2	KTN1-AS1	0.786361588	CPNE3	H19	0.646958643	C1orf112	MCM3AP-AS1	1
TSGA10	SNHG5	0.786318631	CPNE3	HCP5	0.788160045	C1orf112	SNHG3	1
ALDH6A1	SNHG5	0.785876794	CPNE3	KTN1-AS1	0.897862782	C1orf21	H19	1
RNF44	DLEU2	0.785488107	CPNE3	ZNRD1-AS1	0.700399778	C1orf21	LINC00467	1
SFMBT1	ZNRD1-AS1	0.784962806	CPNE8	H19	0.506901371	C1orf21	ZNRD1-AS1	1
HECA	HCP5	0.784699253	CPNE8	HCP5	0.821932083	C1orf74	HCP5	1
E2F5	HCP5	0.784612556	CPNE8	KTN1-AS1	0.915363713	C1orf74	TP53TG1	1
PLXNA2	MCM3AP-AS1	0.784319176	CPNE8	MCM3AP-AS1	0.866395693	C20orf194	RUSC1-AS1	1
TAB3	HCP5	0.783883429	CPNE8	TPT1-AS1	0.51074962	C20orf194	SNHG5	1
SHCBP1	H19	0.783823848	CPNE8	ZNRD1-AS1	0.77506708	C20orf194	ZNRD1-AS1	1
CREM	DLEU2	0.783788425	CPS1	H19	0.712157497	C21orf33	HCP5	1
SFXN2	MCM3AP-AS1	0.783592051	CPS1	HCP5	0.66910538	C2CD2	H19	1
GLTP	HCP5	0.783415965	CPVL	H19	0.557552263	C2CD2	SNHG5	1
MBOAT2	TP53TG1	0.783235589	CRAMP1L	H19	0.572407504	C2CD2	ZNRD1-AS1	1
RAD51C	LINC00467	0.782822907	CRAMP1L	KTN1-AS1	0.991545537	C3	SNHG5	1
PIGM	KTN1-AS1	0.782581614	CRAMP1L	MCM3AP-AS1	0.990693284	C3orf58	HCP5	1
TOMM34	RUSC1-AS1	0.782500053	CRAMP1L	ZNRD1-AS1	0.951498522	C3orf58	MCM3AP-AS1	1
NPRL2	SNHG5	0.782330454	CRBN	KTN1-AS1	0.938554748	C4orf19	SCARNA9	1
PTPDC1	KTN1-AS1	0.782113565	CRBN	MCM3AP-AS1	0.98266693	C5orf15	DLEU2	1
PIK3IP1	RUSC1-AS1	0.782031582	CREB5	RUSC1-AS1	0.957983179	C5orf15	H19	1
TCERG1	ZNRD1-AS1	0.78186222	CREBBP	KTN1-AS1	0.805296424	C5orf15	SNHG5	1
SGMS1	TP53TG1	0.781858206	CREBBP	TPT1-AS1	0.992232221	C5orf15	ZNRD1-AS1	1
RAI14	DLEU2	0.781420999	CREBRF	MCM3AP-AS1	0.776290613	C5orf30	HCP5	1
ATG5	DLEU2	0.781361891	CREBRF	ZNRD1-AS1	0.881300639	C5orf30	KTN1-AS1	1
PIK3CG	SCARNA9	0.781261246	CREG1	H19	0.559047708	C5orf30	MCM3AP-AS1	1
ECHDC2	TP53TG1	0.78123321	CREG1	HCP5	0.888277791	C5orf30	SCARNA9	1
SMYD2	SNHG5	0.780842911	CREG1	KTN1-AS1	0.8024529	C5orf30	SNHG3	1
RPAP3	DLEU2	0.780729743	CREG1	MCM3AP-AS1	0.716231868	C6orf120	HCP5	1
JAG2	ZNRD1-AS1	0.780536076	CREG1	ZNRD1-AS1	0.586776872	C6orf120	MCM3AP-AS1	1
MARCKS	HCP5	0.780484307	CRELD1	SNHG5	0.634577563	C6orf211	HCP5	1
CHM	SNHG5	0.780463437	CREM	SNHG3	0.877805703	C6orf211	KTN1-AS1	1
PSMC3IP	H19	0.780083592	CREM	SNHG5	0.743436744	C6orf211	MCM3AP-AS1	1
FAM46A	KTN1-AS1	0.780056845	CRIM1	RHPN1-AS1	0.868702201	C6orf211	SNHG3	1
RANBP17	MCM3AP-AS1	0.779631761	CRIM1	SNHG3	0.678093033	C8orf33	MCM3AP-AS1	1
MEIS1	MCM3AP-AS1	0.779436689	CROT	H19	0.948125984	C9orf40	HCP5	1
KIAA0922	SNHG3	0.779428806	CROT	TP53TG1	0.88707817	C9orf40	SNHG3	1
TBC1D9	SNHG5	0.779335886	CRTAP	KTN1-AS1	0.878869579	C9orf9	ZNRD1-AS1	1
SLC20A2	SNHG3	0.779281331	CRTAP	MCM3AP-AS1	0.819049098	CA13	MCM3AP-AS1	1
ZFP36L2	SNHG3	0.779202432	CSF1	SNHG3	0.620462468	CA2	HCP5	1
BBS2	KTN1-AS1	0.779194502	CSNK1G1	SNHG3	0.992918464	CA2	KTN1-AS1	1

RFXAP	HCP5	0.779144618	CSRP2BP	H19	0.581646453	CA2	MCM3AP-AS1	1
SNRNP40	KTN1-AS1	0.778244684	CSTF3	H19	0.679385865	CA8	ZNRD1-AS1	1
ATAD2	H19	0.778072565	CSTF3	HCP5	0.69145579	CAB39	H19	1
UFD1L	H19	0.77787547	CTDSPL	LINC00467	0.975385697	CAB39	LINC00467	1
ADK	RUSC1-AS1	0.77784395	CTDSPL	MCM3AP-AS1	0.818668833	CAB39	RUSC1-AS1	1
SEC61A2	ZNRD1-AS1	0.777622914	CTDSPL	ZNRD1-AS1	0.698017221	CAB39	ZNRD1-AS1	1
ANXA2	KTN1-AS1	0.777407782	CTNNAL1	H19	0.610683576	CABLES2	HCP5	1
ERICH1	ZNRD1-AS1	0.777340451	CTNNBIP1	H19	0.633054775	CACNB2	SNHG3	1
ALG1	SNHG5	0.777328945	CTNNBIP1	HCP5	0.790050858	CADM1	HCP5	1
AEN	HCP5	0.777309036	CTNNBIP1	KTN1-AS1	0.904339972	CADM1	MCM3AP-AS1	1
RBM34	TPT1-AS1	0.777158019	CTNNBIP1	MCM3AP-AS1	0.831960876	CADM1	SCARNA9	1
SLC35D2	LINC00467	0.776983462	CTNNBIP1	ZNRD1-AS1	0.714431863	CADM1	SNHG3	1
ATP7B	HCP5	0.776873257	CTPS2	H19	0.657071452	CALD1	HCP5	1
B4GALT6	SNHG3	0.77683544	CTPS2	LINC00467	0.903977385	CALD1	MCM3AP-AS1	1
CCDC15	H19	0.77666611	CTSD	H19	0.507407689	CALD1	SNHG3	1
FRMD6	HCP5	0.776663079	CTSD	MCM3AP-AS1	0.922271859	CALU	HCP5	1
HPS3	SCARNA9	0.776639873	CTSF	LINC00467	0.786177908	CALU	MCM3AP-AS1	1
RALA	RUSC1-AS1	0.776494864	CTSO	HCP5	0.96259896	CALU	SNHG3	1
CDC7	MCM3AP-AS1	0.776436253	CTTNBP2NL	DLEU2	0.677663904	CALU	TP53TG1	1
RPSA	SNHG5	0.776186528	CTTNBP2NL	SNHG3	0.580883951	CAMK1	KTN1-AS1	1
GLMN	RUSC1-AS1	0.775903363	CTTNBP2NL	SNHG5	0.868574948	CAMK1	MCM3AP-AS1	1
SRSF7	ZNRD1-AS1	0.775574698	CUEDC2	SNHG3	0.75436217	CAMK1D	SNHG5	1
KIF5C	MCM3AP-AS1	0.774720582	CUL4B	HCP5	0.954390701	CAMK2D	HCP5	1
IARS	MCM3AP-AS1	0.774564976	CUL4B	MCM3AP-AS1	0.665600355	CAMK2D	MCM3AP-AS1	1
CNKSR3	MCM3AP-AS1	0.774493966	CUL4B	ZNRD1-AS1	0.553564421	CAMK2D	RHPN1-AS1	1
SFMBT1	H19	0.774465134	CUTC	H19	0.91625717	CAMK2D	SNHG3	1
CTNNAL1	ZNRD1-AS1	0.77429272	CUTC	HCP5	0.536506299	CAMK2G	MCM3AP-AS1	1
PHYH	HCP5	0.77406503	CUTC	KTN1-AS1	0.683490405	CAMK2G	SNHG3	1
VAMP8	RHPN1-AS1	0.774063575	CUTC	MCM3AP-AS1	0.547875641	CAMLG	H19	1
UQCRB	LINC00467	0.773986406	CUX1	MCM3AP-AS1	0.920769386	CAMLG	LINC00467	1
DCUN1D5	MCM3AP-AS1	0.773383744	CUX1	ZNRD1-AS1	0.961525986	CAMLG	ZNRD1-AS1	1
STEAP3	MCM3AP-AS1	0.77332992	CXCL12	SCARNA9	0.959388255	CAMSAP2	DLEU2	1
H2AFY	HCP5	0.773164997	CYB5D1	H19	0.670974753	CAMSAP2	H19	1
SGMS1	SNHG5	0.773027547	CYBRD1	H19	0.535798334	CAMSAP2	LINC00467	1
TMEM242	ZNRD1-AS1	0.772997008	CYBRD1	HCP5	0.917376917	CAMSAP2	SNHG5	1
ME1	SNHG5	0.772746365	CYBRD1	KTN1-AS1	0.67407077	CAMSAP2	ZNRD1-AS1	1
CNOT6L	HCP5	0.772612662	CYBRD1	MCM3AP-AS1	0.567699745	CARD16	ZNRD1-AS1	1
KCTD12	SNHG5	0.77235317	CYP2S1	MCM3AP-AS1	0.91978602	CARD6	SNHG5	1
FOXRED1	SNHG3	0.772224393	CYP2S1	ZNRD1-AS1	0.820831918	CASK	H19	1
ATP8A1	MCM3AP-AS1	0.7720613	CYP2U1	H19	0.600719314	CASK	SNHG5	1
JUP	SCARNA9	0.771995137	CYP2U1	HCP5	0.838442493	CASK	ZNRD1-AS1	1
FBXW7	ZNRD1-AS1	0.771335148	CYP4V2	HCP5	0.866436581	CASP10	MCM3AP-AS1	1
STK38L	HCP5	0.771237666	CYSLTR2	H19	0.738657392	CASP2	HCP5	1
8-Sep	SNHG5	0.770837	CYTH3	SNHG3	0.587138281	CASP2	MCM3AP-AS1	1
CSRNP1	HCP5	0.770692703	CYTH4	ZNRD1-AS1	0.974192088	CASP4	H19	1
FLNB	HCP5	0.770382975	DAB2	H19	0.578843938	CASP4	LINC00467	1
ZDHHC1	HCP5	0.770256377	DACH1	MCM3AP-AS1	0.85125386	CASP4	ZNRD1-AS1	1
EGR2	HCP5	0.770219356	DACH1	TP53TG1	0.80060626	CASP7	DLEU2	1
IGFBP7	SNHG3	0.769956213	DACH1	ZNRD1-AS1	0.737682955	CASP7	H19	1
TMEM194A	H19	0.769954982	DACT1	TPT1-AS1	0.603069324	CASP7	LINC00467	1
MRPL35	TPT1-AS1	0.769637808	DAG1	HCP5	0.962031819	CASP7	TPT1-AS1	1

NME4	HCP5	0.769594331	DAGLA	ZNRD1-AS1	0.660890764	CASP7	ZNRD1-AS1	1
SBF2	RUSC1-AS1	0.769498279	DAGLB	H19	0.709209629	CASP8AP2	SCARNA9	1
WDR43	ZNRD1-AS1	0.769418086	DAP3	SNHG3	0.965267247	CASP8AP2	SNHG3	1
FSD1L	SNHG5	0.769141926	DBI	SNHG3	0.972180256	CAST	DLEU2	1
DENND5A	DLEU2	0.768942588	DBP	MCM3AP-AS1	0.8944448	CAST	H19	1
TBC1D12	HCP5	0.768556849	DCAF17	H19	0.727222576	CAST	TPT1-AS1	1
GPR126	HCP5	0.768419693	DCAF17	HCP5	0.712300052	CAST	ZNRD1-AS1	1
FAM45A	SNHG3	0.768399206	DCAF17	LINC00467	0.989891645	CASZ1	H19	1
ABCB6	H19	0.767924038	DCAF17	MCM3AP-AS1	0.831063931	CASZ1	SNHG5	1
ZNF326	DLEU2	0.767899729	DCAF17	ZNRD1-AS1	0.703775856	CASZ1	ZNRD1-AS1	1
RPS6KC1	MCM3AP-AS1	0.767844818	DCBLD2	SNHG3	0.739937367	CAT	DLEU2	1
MAP7	ZNRD1-AS1	0.767692627	DCBLD2	SNHG5	0.59233467	CAT	TPT1-AS1	1
KDM2B	DLEU2	0.767477696	DCHS1	SNHG5	0.519898836	CBLB	DLEU2	1
EIF2AK4	HCP5	0.767201211	DCK	H19	0.649684246	CBLB	TPT1-AS1	1
FRY	H19	0.766942771	DCLK2	HCP5	0.896807042	CBLN3	ZNRD1-AS1	1
EBF1	RUSC1-AS1	0.766428617	DCLRE1B	HCP5	0.829553653	CBS	RHPN1-AS1	1
BAZ1A	TPT1-AS1	0.766083088	DCLRE1B	MCM3AP-AS1	0.507755064	CBX2	DLEU2	1
RAB3D	SNHG5	0.765870193	DCTN4	SNHG3	0.831490193	CBX2	H19	1
EPHB4	HCP5	0.765696835	DCTPP1	H19	0.679002106	CBX2	LINC00467	1
DACH1	MCM3AP-AS1	0.765684002	DCUN1D3	H19	0.804234042	CBX2	TPT1-AS1	1
PRRG1	SNHG3	0.76548499	DCUN1D3	HCP5	0.667323611	CBX2	ZNRD1-AS1	1
INF2	H19	0.765423827	DCUN1D3	ZNRD1-AS1	0.624208478	CBX5	HCP5	1
ALDH5A1	RUSC1-AS1	0.765180154	DCUN1D5	TP53TG1	0.702953744	CBX5	KTN1-AS1	1
ZNF624	HCP5	0.764812985	DDB2	H19	0.674269029	CBX5	MCM3AP-AS1	1
IPO4	SNHG5	0.764768087	DDB2	MCM3AP-AS1	0.662703522	CBX5	SNHG3	1
DNMT1	H19	0.764670325	DDO	HCP5	0.571736889	CBX6	MCM3AP-AS1	1
UHRF1BP1	DLEU2	0.764622026	DDX11	TPT1-AS1	0.907328201	CCBL1	DLEU2	1
MYBL1	TPT1-AS1	0.764567718	DDX21	SNHG3	0.9294612	CCBL1	TPT1-AS1	1
CAMSAP2	DLEU2	0.764541708	DDX52	MCM3AP-AS1	0.950815932	CCDC109B	ZNRD1-AS1	1
CKAP2	H19	0.764390572	DDX52	ZNRD1-AS1	0.993804167	CCDC126	KTN1-AS1	1
STOM	SNHG3	0.764387342	DDX60	SNHG3	0.921494926	CCDC126	MCM3AP-AS1	1
EDARADD	SCARNA9	0.764185693	DECR2	SNHG3	0.766086033	CCDC138	HCP5	1
ZNF395	SNHG3	0.763841328	DEK	H19	0.778397936	CCDC15	MCM3AP-AS1	1
NUCB2	DLEU2	0.763643056	DEK	HCP5	0.741193335	CCDC28A	KTN1-AS1	1
SYDE2	H19	0.763516249	DEK	KTN1-AS1	0.744651441	CCDC28A	MCM3AP-AS1	1
EGLN3	KTN1-AS1	0.763515353	DEK	MCM3AP-AS1	0.622336684	CCDC28A	SCARNA9	1
MEIS1	SNHG5	0.763379377	DENND3	SNHG3	0.683565029	CCDC34	KTN1-AS1	1
SFMBT2	SNHG5	0.76335977	DENND4A	DLEU2	0.635468267	CCDC34	MCM3AP-AS1	1
NLN	HCP5	0.763302707	DENND4A	RUSC1-AS1	0.901859338	CCDC43	HCP5	1
ELK3	RUSC1-AS1	0.7631593	DENND4A	TPT1-AS1	0.803703603	CCDC43	SNHG3	1
CPNE3	HCP5	0.763158158	DENND4A	ZNRD1-AS1	0.624545596	CCDC50	DLEU2	1
NAPB	ZNRD1-AS1	0.763123268	DENND5A	KTN1-AS1	0.693973955	CCDC50	SNHG5	1
LAMC1	SNHG3	0.763002589	DENND5A	MCM3AP-AS1	0.801576526	CCDC50	TPT1-AS1	1
MAPKAP1	MCM3AP-AS1	0.762744682	DENND5A	ZNRD1-AS1	0.89751895	CCDC53	SCARNA9	1
RRAGD	SNHG3	0.762614768	DEPDC1	H19	0.826847529	CCDC53	TP53TG1	1
ABL1	DLEU2	0.762243658	DEPDC1	HCP5	0.544860434	CCDC58	MCM3AP-AS1	1
USP53	HCP5	0.762167649	DEPDC1	LINC00467	0.825086427	CCDC59	SNHG5	1
PCMTD2	KTN1-AS1	0.762119274	DEPDC1B	TP53TG1	0.984107704	CCDC68	H19	1
PDK1	SNHG5	0.761877944	DGKD	TPT1-AS1	0.829414577	CCDC68	ZNRD1-AS1	1
MGST1	SCARNA9	0.76170001	DHCR24	H19	0.642441764	CCDC71L	HCP5	1
PDRG1	HCP5	0.761652252	DHFR	MCM3AP-AS1	0.553850856	CCDC71L	TP53TG1	1

IFNAR2	DLEU2	0.761379541	DHODH	H19	0.761767398	CCDC8	H19	1
SOCS5	HCP5	0.761234118	DHODH	HCP5	0.587632947	CCDC82	DLEU2	1
EFNA3	SCARNA9	0.760883751	DHODH	TPT1-AS1	0.578154944	CCDC82	H19	1
DST	H19	0.760798129	DHODH	ZNRD1-AS1	0.777784752	CCDC82	TPT1-AS1	1
HADH	SNHG3	0.760369142	DHRS4L2	MCM3AP-AS1	0.879920729	CCDC82	ZNRD1-AS1	1
CDK4	SNHG3	0.760238445	DHRS4L2	ZNRD1-AS1	0.765583402	CCDC85C	HCP5	1
TRIB1	SNHG5	0.759850312	DHX15	SNHG3	0.980855633	CCDC85C	MCM3AP-AS1	1
ZFP36L2	HCP5	0.75955635	DHX15	SNHG5	0.774804458	CCDC85C	SNHG3	1
ITGB8	HCP5	0.759409256	DHX33	H19	0.70092078	CCM2	KTN1-AS1	1
TLE4	MCM3AP-AS1	0.75939862	DHX33	MCM3AP-AS1	0.752826675	CCM2	MCM3AP-AS1	1
CREM	HCP5	0.759209972	DHX33	ZNRD1-AS1	0.724763533	CCM2	RHPN1-AS1	1
CCNJL	SNHG5	0.759084098	DHX38	ZNRD1-AS1	0.616628615	CCNA1	DLEU2	1
WDR47	MCM3AP-AS1	0.758996941	DIAPH3	H19	0.756366721	CCNA1	H19	1
SEMA3C	RUSC1-AS1	0.758954011	DIRC2	HCP5	0.791608794	CCNA1	TPT1-AS1	1
TBC1D2	HCP5	0.758904438	DIRC2	MCM3AP-AS1	0.902867778	CCNA1	ZNRD1-AS1	1
C1orf74	TP53TG1	0.758898409	DIRC2	ZNRD1-AS1	0.859879668	CCNA2	HCP5	1
ARHGAP26	HCP5	0.758845881	DIS3L	H19	0.920056716	CCNA2	MCM3AP-AS1	1
PDK4	SNHG5	0.758839321	DIS3L	HCP5	0.529784636	CCNB1	MCM3AP-AS1	1
PDE4DIP	HCP5	0.75862386	DIXDC1	HCP5	0.573935819	CCNC	DLEU2	1
CD83	DLEU2	0.757862917	DKC1	H19	0.611856561	CCNC	H19	1
ITM2B	KTN1-AS1	0.756873562	DLEU1	TP53TG1	0.972035793	CCNC	RUSC1-AS1	1
CPSF3	SNHG5	0.756807656	DLGAP5	TP53TG1	0.984573923	CCNC	SNHG5	1
GMFB	HCP5	0.756772097	DMRTA2	HCP5	0.809575743	CCNC	TPT1-AS1	1
KLHL36	HCP5	0.756750153	DNA2	TP53TG1	0.899189011	CCNC	ZNRD1-AS1	1
TNFRSF10B	ZNRD1-AS1	0.756702712	DNAJA3	H19	0.991794748	CCND3	MCM3AP-AS1	1
ABTB2	HCP5	0.75639125	DNAJA4	SNHG3	0.517084406	CCNE1	MCM3AP-AS1	1
VPS26B	RHPN1-AS1	0.756306589	DNAJB11	SNHG5	0.874425535	CCNE1	RHPN1-AS1	1
DNAJC18	RUSC1-AS1	0.75617316	DNAJC18	SNHG3	0.541534639	CCNE2	HCP5	1
VGLL4	SCARNA9	0.755816908	DNAJC3	SNHG3	0.956393417	CCNE2	KTN1-AS1	1
RCOR1	ZNRD1-AS1	0.755766339	DNAJC7	SNHG3	0.520040744	CCNE2	MCM3AP-AS1	1
SPRED1	HCP5	0.755501525	DNM1	LINC00467	0.84261621	CCNJL	DLEU2	1
TXNDC9	H19	0.754918654	DNMT1	RUSC1-AS1	0.559456896	CCNJL	RUSC1-AS1	1
CIT	H19	0.754659074	DNMT1	TPT1-AS1	0.989017285	CCNJL	SNHG5	1
PAXIP1	H19	0.75465654	DNMT1	ZNRD1-AS1	0.876196451	CCNJL	TPT1-AS1	1
ACSS1	SNHG5	0.754596444	DOCK4	SNHG3	0.758671398	CCNJL	ZNRD1-AS1	1
FAM117B	TP53TG1	0.754514264	DOCK4	SNHG5	0.751658411	CCNYL1	H19	1
EBF1	TP53TG1	0.754005617	DOCK9	SNHG5	0.620538852	CCNYL1	ZNRD1-AS1	1
LMNB1	H19	0.753984257	DPF1	MCM3AP-AS1	0.574395999	CCP110	HCP5	1
NDUFB10	SNHG5	0.753753888	DPM2	HCP5	0.907634974	CCRN4L	H19	1
SMAD1	HCP5	0.75364812	DPPA4	ZNRD1-AS1	0.619120124	CCRN4L	ZNRD1-AS1	1
CLIP4	MCM3AP-AS1	0.753316094	DPY19L3	H19	0.533808326	CCT2	HCP5	1
EIF2B2	HCP5	0.753018385	DPY19L3	HCP5	0.916828347	CCT2	MCM3AP-AS1	1
PTPRC	SCARNA9	0.752823832	DPYD	H19	0.68572616	CCT2	SNHG3	1
ORAI3	RHPN1-AS1	0.752588378	DPYD	HCP5	0.795651824	CCT5	SNHG3	1
FCHO2	SNHG3	0.752494043	DPYD	KTN1-AS1	0.850939258	CCT6A	HCP5	1
STK17B	HCP5	0.752332444	DPYD	MCM3AP-AS1	0.758401071	CCT8	ZNRD1-AS1	1
GLIPR1	RUSC1-AS1	0.752314745	DPYD	ZNRD1-AS1	0.619967281	CD164	HCP5	1
ZEB1	HCP5	0.752293391	DPYSL2	H19	0.718042546	CD164	MCM3AP-AS1	1
ZBTB10	HCP5	0.752223405	DPYSL2	HCP5	0.641141516	CD164	SCARNA9	1
LSM11	SNHG5	0.752093395	DPYSL3	H19	0.634547853	CD164	SNHG3	1
SERP1	HCP5	0.752070306	DPYSL3	LINC00467	0.966005399	CD24	HCP5	1

SLC39A14	SNHG5	0.751833946	DPYSL3	MCM3AP-AS1	0.868468625	CD24	SNHG3	1
TSPYL1	LINC00467	0.751814809	DPYSL3	ZNRD1-AS1	0.759483536	CD274	H19	1
KDM2B	TPT1-AS1	0.751278256	DRAM1	LINC00467	0.804041948	CD274	ZNRD1-AS1	1
HSPA4L	HCP5	0.751211973	DRAM1	MCM3AP-AS1	0.983739891	CD276	H19	1
ERMP1	SNHG5	0.750890494	DRAM1	ZNRD1-AS1	0.937382072	CD276	ZNRD1-AS1	1
RGMA	ZNRD1-AS1	0.750720698	DRG1	LINC00467	0.938577874	CD302	KTN1-AS1	1
ZDBF2	KTN1-AS1	0.750553349	DSC2	SNHG5	0.790977211	CD302	MCM3AP-AS1	1
ZP3	RHPN1-AS1	0.750446833	DSCC1	HCP5	0.661622725	CD36	SCARNA9	1
PPAT	KTN1-AS1	0.750303995	DTL	HCP5	0.657172915	CD37	RHPN1-AS1	1
CDC7	SNHG5	0.750071794	DTWD1	HCP5	0.921562951	CD37	SNHG3	1
ACVR1C	SNHG5	0.750018726	DTYMK	RHPN1-AS1	0.79458564	CD4	RUSC1-AS1	1
FZD3	SNHG3	0.749051391	DUSP1	SNHG5	0.713063919	CD44	H19	1
WEE1	ZNRD1-AS1	0.748497031	DUSP16	KTN1-AS1	0.898485587	CD44	LINC00467	1
IFI16	MCM3AP-AS1	0.74839186	DUSP16	LINC00467	0.597135117	CD47	HCP5	1
RPS6KA2	HCP5	0.747914811	DUSP16	MCM3AP-AS1	0.949598164	CD47	MCM3AP-AS1	1
ZNF248	ZNRD1-AS1	0.747392103	DUSP19	TP53TG1	0.634125201	CD47	SNHG3	1
RUNX2	MCM3AP-AS1	0.746690696	DUSP22	SNHG5	0.501151439	CD48	DLEU2	1
FERMT2	TPT1-AS1	0.746685063	DUSP5	SNHG5	0.828564871	CD48	ZNRD1-AS1	1
FAM161A	ZNRD1-AS1	0.746515803	DUSP7	H19	0.800421458	CD59	SCARNA9	1
MKI67	H19	0.746267495	DUSP7	TPT1-AS1	0.527885849	CD69	HCP5	1
B4GALT6	HCP5	0.745851119	DUSP8	ZNRD1-AS1	0.870330198	CD69	KTN1-AS1	1
SESN3	SNHG5	0.745810185	DUT	H19	0.805401594	CD69	MCM3AP-AS1	1
USP16	ZNRD1-AS1	0.745747961	DYNLT3	SNHG5	0.847640233	CD70	MCM3AP-AS1	1
PIP5K1B	HCP5	0.745731045	DYRK1B	MCM3AP-AS1	0.992013279	CD79A	RHPN1-AS1	1
QDPR	HCP5	0.745632675	DYRK1B	ZNRD1-AS1	0.989266544	CD80	DLEU2	1
LIX1L	SNHG3	0.745396505	DZIP1	SCARNA9	0.935083391	CD80	H19	1
PPIH	ZNRD1-AS1	0.745393406	E2F1	H19	0.748669082	CD80	TPT1-AS1	1
CRAMP1L	DLEU2	0.744992637	E2F2	H19	0.744105991	CD80	ZNRD1-AS1	1
TRAPPC8	DLEU2	0.744781718	E2F2	HCP5	0.65862172	CD83	DLEU2	1
RCC1	H19	0.744778084	E2F3	SNHG5	0.812703916	CD83	H19	1
BTN3A3	MCM3AP-AS1	0.744769865	E2F5	H19	0.637126749	CD83	LINC00467	1
SNX7	SNHG5	0.744503472	E2F5	HCP5	0.520333737	CD83	TPT1-AS1	1
PRR11	H19	0.744476745	E2F5	KTN1-AS1	0.997706456	CD83	ZNRD1-AS1	1
DPYD	KTN1-AS1	0.744410623	E2F5	LINC00467	0.874216008	CD99	RUSC1-AS1	1
CUL4B	SNHG3	0.744361668	E2F5	TP53TG1	0.63798986	CDADC1	KTN1-AS1	1
ACADM	ZNRD1-AS1	0.744284966	E2F5	ZNRD1-AS1	0.905247991	CDADC1	MCM3AP-AS1	1
TPP1	HCP5	0.744201729	E2F7	H19	0.608751693	CDADC1	SCARNA9	1
RAPGEFL1	ZNRD1-AS1	0.744154315	E2F7	HCP5	0.505146312	CDC23	DLEU2	1
ACAA2	SNHG3	0.744114761	E2F8	H19	0.558820997	CDC23	H19	1
RFK	MCM3AP-AS1	0.744062414	EBF1	RUSC1-AS1	0.668082752	CDC23	SNHG5	1
ISCU	KTN1-AS1	0.743969745	ECHDC1	SNHG3	0.679058499	CDC23	TPT1-AS1	1
TTLL7	MCM3AP-AS1	0.743784452	ECT2	H19	0.763188708	CDC23	ZNRD1-AS1	1
YTHDC2	TPT1-AS1	0.743695083	ECT2	TP53TG1	0.892597952	CDC25A	HCP5	1
LRRK2	TP53TG1	0.743598152	EDA2R	H19	0.614867679	CDC25A	MCM3AP-AS1	1
ARHGEF10	H19	0.742917627	EDA2R	HCP5	0.671606445	CDC42BPB	DLEU2	1
PAICS	MCM3AP-AS1	0.742309002	EDA2R	KTN1-AS1	0.978458352	CDC42BPB	ZNRD1-AS1	1
FAM188A	TPT1-AS1	0.742213933	EDA2R	MCM3AP-AS1	0.936315647	CDC42EP2	H19	1
SPTBN1	H19	0.741757644	EDA2R	ZNRD1-AS1	0.850448053	CDC42EP2	SNHG5	1
SH3BP4	RUSC1-AS1	0.741705626	EDARADD	SNHG5	0.647541939	CDC42EP2	ZNRD1-AS1	1
FRMD6	LINC00467	0.741434454	EDEM1	SNHG5	0.904032963	CDC7	HCP5	1
RPL34	SNHG3	0.741400199	EEPD1	H19	0.730177433	CDC7	MCM3AP-AS1	1

NFIA	KTN1-AS1	0.741379326	EEPD1	HCP5	0.732305574	CDCA2	TP53TG1	1
GCA	HCP5	0.741374484	EEPD1	ZNRD1-AS1	0.667922804	CDCA3	TP53TG1	1
RUNX2	SNHG3	0.741309893	EFNA3	H19	0.552505903	CDCA4	H19	1
SPAG7	SCARNA9	0.741151402	EFNA3	HCP5	0.885422585	CDCA4	ZNRD1-AS1	1
MRPL13	SCARNA9	0.741138046	EFNA3	MCM3AP-AS1	0.734407274	CDCA5	HCP5	1
PANK1	MCM3AP-AS1	0.741094709	EFNA3	ZNRD1-AS1	0.60898173	CDCA5	SNHG3	1
DUSP1	SNHG3	0.740823133	EFNA4	H19	0.653891946	CDCA7	HCP5	1
MTSS1L	ZNRD1-AS1	0.740731116	EGLN3	HCP5	0.940201894	CDCA7	SNHG3	1
FLVCR2	HCP5	0.740518163	EGLN3	KTN1-AS1	0.785638577	CDCA8	SCARNA9	1
NRIP1	HCP5	0.740324781	EGLN3	LINC00467	0.854031787	CDCA8	SNHG3	1
PLEKHM1	HCP5	0.740127903	EGLN3	MCM3AP-AS1	0.733450859	CDCP1	H19	1
GALNT10	H19	0.74010998	EGLN3	ZNRD1-AS1	0.643336339	CDH1	DLEU2	1
EPB41L2	ZNRD1-AS1	0.739929071	EGR2	SNHG5	0.896539004	CDH1	SNHG5	1
RHOBTB1	SNHG5	0.739828178	EHD1	RHPN1-AS1	0.858425937	CDH1	TPT1-AS1	1
PCSK5	MCM3AP-AS1	0.73956923	EHD1	SNHG5	0.669494579	CDH1	ZNRD1-AS1	1
SLC16A9	SNHG5	0.739239606	EHD2	SCARNA9	0.519199192	CDH2	HCP5	1
SMAD6	KTN1-AS1	0.739229184	EHD3	H19	0.556770715	CDH2	MCM3AP-AS1	1
TARBP1	ZNRD1-AS1	0.739157204	EHD3	HCP5	0.813072916	CDH2	SCARNA9	1
INTS6	DLEU2	0.739142078	EHD3	ZNRD1-AS1	0.753330065	CDH2	SNHG3	1
MYO5A	ZNRD1-AS1	0.739085452	EHD4	SNHG3	0.960861259	CDIPT	HCP5	1
NAPEPLD	MCM3AP-AS1	0.739040687	EHMT2	HCP5	0.684026675	CDIPT	MCM3AP-AS1	1
ATP2B4	KTN1-AS1	0.738899653	EIF2AK4	HCP5	0.551960695	CDK1	MCM3AP-AS1	1
VEGFA	DLEU2	0.738879701	EIF2AK4	ZNRD1-AS1	0.766450569	CDK1	SCARNA9	1
ROBO1	H19	0.73884973	EIF2S2	H19	0.863662744	CDK1	SNHG3	1
DGKD	H19	0.738693279	EIF2S2	TP53TG1	0.976930597	CDK13	H19	1
AP1S3	DLEU2	0.738627441	EIF3J	HCP5	0.879967692	CDK13	LINC00467	1
PTPLB	HCP5	0.738621156	EIF3J	MCM3AP-AS1	0.721974457	CDK13	RUSC1-AS1	1
SP1	SCARNA9	0.738401259	EIF4E3	H19	0.887898659	CDK13	SNHG5	1
SLC48A1	LINC00467	0.738123488	EIF5	SNHG5	0.700580162	CDK13	ZNRD1-AS1	1
SIX4	TP53TG1	0.738034678	ELK3	H19	0.864589092	CDK14	DLEU2	1
SYDE2	ZNRD1-AS1	0.738031837	ELK3	KTN1-AS1	0.560736367	CDK14	H19	1
MTSS1L	H19	0.738003481	ELK3	LINC00467	0.515286043	CDK14	SNHG5	1
SESN1	SNHG5	0.737687116	ELK3	MCM3AP-AS1	0.5086464	CDK14	ZNRD1-AS1	1
FCHO1	H19	0.737572356	ELL	MCM3AP-AS1	0.626601702	CDK18	HCP5	1
MAMDC2	SNHG5	0.737333753	ELOVL6	KTN1-AS1	0.907424758	CDK18	RHPN1-AS1	1
KIAA0895	SNHG3	0.737279365	ELOVL6	MCM3AP-AS1	0.860844191	CDK2	HCP5	1
ARL13B	HCP5	0.736830954	EMB	HCP5	0.802974393	CDK4	SCARNA9	1
RRAGD	HCP5	0.73673711	EMG1	H19	0.545932987	CDK4	SNHG3	1
MPP6	KTN1-AS1	0.736723143	EML4	KTN1-AS1	0.575745435	CDK5	HCP5	1
SLC35F6	HCP5	0.736705029	EML4	TPT1-AS1	0.862472698	CDK5	MCM3AP-AS1	1
EGLN3	MCM3AP-AS1	0.73651616	EML4	ZNRD1-AS1	0.689339326	CDK5	RHPN1-AS1	1
MYCN	MCM3AP-AS1	0.736484349	EMP1	SNHG3	0.869434942	CDK6	HCP5	1
GLCE	KTN1-AS1	0.736267519	ENAH	DLEU2	0.639286149	CDK6	MCM3AP-AS1	1
SASS6	ZNRD1-AS1	0.736050886	ENC1	SNHG3	0.926467237	CDK6	SCARNA9	1
VAMP4	MCM3AP-AS1	0.736040025	ENC1	SNHG5	0.884272179	CDK6	SNHG3	1
DNAJB12	DLEU2	0.735884055	ENOX1	SNHG5	0.843493841	CDK6	TP53TG1	1
FCHO2	TP53TG1	0.735764832	ENPP2	H19	0.994460329	CDKN1A	H19	1
FOXO3	KTN1-AS1	0.735707781	ENPP2	TP53TG1	0.912182838	CDKN1A	ZNRD1-AS1	1
ZNF217	HCP5	0.735562697	ENPP4	SNHG3	0.923346036	CDKN1B	KTN1-AS1	1
THEM4	H19	0.735457376	ENPP4	SNHG5	0.742900174	CDKN1B	MCM3AP-AS1	1
TUBGCP4	KTN1-AS1	0.735394076	EPB41L2	H19	0.592513827	CDKN1B	SNHG3	1

CRIM1	RHPN1-AS1	0.735388892	EPB41L3	H19	0.550097268	CDKN2A	HCP5	1
ZXDA	SNHG5	0.735378098	EPB41L5	DLEU2	0.686788394	CDKN3	SNHG3	1
APP	HCP5	0.734915224	EPCAM	SNHG5	0.827976718	CDYL2	RUSC1-AS1	1
MRS2	ZNRD1-AS1	0.734285417	EPHB4	MCM3AP-AS1	0.890160325	CEBPG	HCP5	1
TPTE2	ZNRD1-AS1	0.73418785	EPHB4	ZNRD1-AS1	0.946386675	CEBPG	SNHG3	1
KCTD3	HCP5	0.733974296	EPN2	SNHG3	0.701259844	CELSR3	DLEU2	1
MED11	SNHG3	0.733665622	EPS8	HCP5	0.982733878	CELSR3	TPT1-AS1	1
LIX1L	HCP5	0.733347697	EPSTI1	SNHG5	0.727999778	CENPF	HCP5	1
APIP	SNHG3	0.733186528	EPT1	SNHG3	0.973086491	CENPF	MCM3AP-AS1	1
TANK	SCARNA9	0.733083629	EPT1	SNHG5	0.818586013	CENPF	SNHG3	1
PMEPA1	DLEU2	0.733053742	ERG	TPT1-AS1	0.885362538	CENPF	TP53TG1	1
USP46	LINC00467	0.732836214	ERG	ZNRD1-AS1	0.990570516	CENPK	HCP5	1
FIGNL1	H19	0.732711284	ERI1	SNHG3	0.654347098	CENPK	MCM3AP-AS1	1
NAV1	MCM3AP-AS1	0.732632487	ERI1	TP53TG1	0.547202763	CENPM	KTN1-AS1	1
PRDM1	MCM3AP-AS1	0.732626215	ERI2	H19	0.876321085	CENPO	H19	1
FANCF	SNHG5	0.73259515	ERI2	HCP5	0.580828327	CENPO	ZNRD1-AS1	1
TMTC4	DLEU2	0.732532141	ERI2	KTN1-AS1	0.592939956	CENPP	MCM3AP-AS1	1
FAM45A	KTN1-AS1	0.731292328	ERICH1	MCM3AP-AS1	0.954474073	CENPQ	HCP5	1
ASB13	SCARNA9	0.731243808	ERICH1	ZNRD1-AS1	0.994707077	CENPQ	KTN1-AS1	1
KCTD12	SNHG3	0.731121947	ERLIN2	H19	0.774731855	CENPQ	SCARNA9	1
IFNAR2	TPT1-AS1	0.731006133	ERLIN2	HCP5	0.580328969	CENPV	RHPN1-AS1	1
SMARCA2	ZNRD1-AS1	0.730952362	ERO1L	HCP5	0.958475554	CENPW	HCP5	1
DHODH	ZNRD1-AS1	0.730863977	ERO1L	KTN1-AS1	0.565694385	CENPW	SNHG3	1
ERI2	H19	0.730644495	ERO1L	TP53TG1	0.784390055	CEP128	H19	1
HOPX	TP53TG1	0.730443284	ERP29	MCM3AP-AS1	0.716741742	CEP128	ZNRD1-AS1	1
LIPT2	HCP5	0.730338462	ESCO2	H19	0.72140679	CEP135	DLEU2	1
NRP1	H19	0.72987477	ESCO2	HCP5	0.695050923	CEP135	TPT1-AS1	1
LRRFIP1	H19	0.729861226	ESCO2	LINC00467	0.844247052	CEP41	SNHG3	1
C16orf70	LINC00467	0.729548418	ESYT2	LINC00467	0.6388153	CEP55	MCM3AP-AS1	1
PAM	SNHG3	0.729346576	ETAA1	HCP5	0.722517852	CEP55	SNHG3	1
SH3BP4	SNHG5	0.729001494	ETAA1	SNHG5	0.503842455	CEP68	HCP5	1
RANBP17	SNHG5	0.728866688	ETF1	SNHG3	0.766167387	CEP97	HCP5	1
RTKN2	H19	0.728845664	ETF1	SNHG5	0.940689135	CEP97	MCM3AP-AS1	1
GAPT	HCP5	0.728038965	ETFB	SNHG3	0.618557842	CERCAM	HCP5	1
UGCG	H19	0.727999955	ETS1	SNHG3	0.883213444	CERK	HCP5	1
LAMA2	HCP5	0.727808462	ETV3	DLEU2	0.741873745	CFLAR	ZNRD1-AS1	1
PTEN	ZNRD1-AS1	0.727677601	ETV4	H19	0.99526529	CHAF1A	HCP5	1
CLIP4	SNHG3	0.727619082	ETV5	SNHG3	0.98470539	CHCHD7	HCP5	1
JMJD8	RHPN1-AS1	0.727290388	ETV5	SNHG5	0.784679934	CHCHD7	SNHG3	1
MAP3K13	TPT1-AS1	0.727287099	EWSR1	SNHG3	0.569243929	CHD2	DLEU2	1
NUCB2	TPT1-AS1	0.726953266	EXO1	H19	0.579379084	CHD2	H19	1
NOG	HCP5	0.726838349	EXOSC5	H19	0.721925115	CHD2	SNHG5	1
ALDH3B2	RHPN1-AS1	0.726703169	EXOSC5	HCP5	0.726633215	CHD2	TPT1-AS1	1
CHD2	TPT1-AS1	0.726678346	EXOSC8	H19	0.609892243	CHD2	ZNRD1-AS1	1
ABHD14A	SNHG5	0.726333498	EXOSC8	HCP5	0.871557719	CHD5	H19	1
CDK14	SNHG5	0.726194383	EXOSC8	ZNRD1-AS1	0.529419447	CHD5	ZNRD1-AS1	1
PTPLAD2	SNHG5	0.725972786	EXPH5	HCP5	0.972703942	CHD7	KTN1-AS1	1
ENPP4	SNHG5	0.725808596	EXPH5	LINC00467	0.83959811	CHD7	MCM3AP-AS1	1
WASL	SCARNA9	0.725622348	EXPH5	TP53TG1	0.75189851	CHD7	SCARNA9	1
CENPF	H19	0.725583789	EYA3	SNHG5	0.775777634	CHD7	SNHG3	1
BCL2	SNHG3	0.724582688	EZH1	H19	0.570502429	CHEK1	MCM3AP-AS1	1

LRCH2	SNHG3	0.724262027	EZH1	MCM3AP-AS1	0.991650092	CHEK1	SCARNA9	1
ISCA2	KTN1-AS1	0.72418273	EZH1	ZNRD1-AS1	0.950390044	CHEK1	SNHG3	1
TCF7L2	SNHG3	0.723961155	EZH2	H19	0.752688025	CHFR	SNHG5	1
TCEA2	LINC00467	0.723605331	EZH2	MCM3AP-AS1	0.885763262	CHM	DLEU2	1
IFI16	ZNRD1-AS1	0.723384141	EZH2	TP53TG1	0.565422808	CHM	SNHG5	1
CCM2	MCM3AP-AS1	0.723376922	EZH2	ZNRD1-AS1	0.827701396	CHM	ZNRD1-AS1	1
MXD1	HCP5	0.723345096	FADD	RHPN1-AS1	0.769992175	CHMP4A	RHPN1-AS1	1
NLGN1	TP53TG1	0.723108263	FAM102B	SNHG3	0.914995662	CHMP4C	MCM3AP-AS1	1
DHX33	H19	0.722795939	FAM107B	SNHG3	0.850812903	CHMP4C	SNHG3	1
SULF2	HCP5	0.722633049	FAM117A	ZNRD1-AS1	0.951366742	CHN1	MCM3AP-AS1	1
SYDE2	MCM3AP-AS1	0.722477534	FAM117B	H19	0.649726103	CHRNA5	HCP5	1
ATM	DLEU2	0.722392641	FAM117B	HCP5	0.84480675	CHRNA5	KTN1-AS1	1
SCAPER	ZNRD1-AS1	0.722353415	FAM117B	LINC00467	0.974509406	CHRNA5	MCM3AP-AS1	1
PIK3CD	DLEU2	0.722294957	FAM117B	MCM3AP-AS1	0.682395148	CHUK	KTN1-AS1	1
EIF5B	H19	0.722150024	FAM117B	TP53TG1	0.888710066	CHUK	MCM3AP-AS1	1
KIAA1147	TPT1-AS1	0.721587564	FAM117B	ZNRD1-AS1	0.535442571	CHUK	SCARNA9	1
BAZ2B	ZNRD1-AS1	0.721512584	FAM120C	LINC00467	0.997621764	CHUK	SNHG3	1
TSHZ1	ZNRD1-AS1	0.721263192	FAM124A	RHPN1-AS1	0.82768136	CIB2	HCP5	1
GPR137B	SNHG5	0.721000208	FAM126A	H19	0.816317667	CIDEB	MCM3AP-AS1	1
FRAT1	SNHG5	0.72067365	FAM127C	SNHG3	0.765594872	CIT	HCP5	1
KIAA0895	MCM3AP-AS1	0.720654825	FAM129A	H19	0.525017731	CIT	MCM3AP-AS1	1
PRMT5	SNHG5	0.720388825	FAM133A	DLEU2	0.615765927	CIT	SNHG3	1
FAT4	HCP5	0.720324396	FAM133A	HCP5	0.72993188	CKAP2	HCP5	1
DIS3	HCP5	0.720187236	FAM133A	SCARNA9	0.973253816	CKAP2	KTN1-AS1	1
FLI1	TP53TG1	0.720013888	FAM134A	H19	0.743972248	CKAP2	MCM3AP-AS1	1
TIPIN	H19	0.719918362	FAM134A	HCP5	0.77574487	CKAP2L	RUSC1-AS1	1
NOP2	ZNRD1-AS1	0.719772517	FAM134A	KTN1-AS1	0.743424633	CKB	ZNRD1-AS1	1
SORL1	SNHG5	0.719623593	FAM134A	MCM3AP-AS1	0.623683101	CKS2	TP53TG1	1
FCHO2	KTN1-AS1	0.719596405	FAM136A	H19	0.765986029	CLCN4	MCM3AP-AS1	1
PRR15	HCP5	0.719593186	FAM136A	HCP5	0.754427875	CLDND1	DLEU2	1
SMAD6	TP53TG1	0.719544303	FAM136A	LINC00467	0.980511824	CLDND1	H19	1
C16orf70	HCP5	0.719282216	FAM160B1	H19	0.538229421	CLDND1	RUSC1-AS1	1
LAMA5	H19	0.719240915	FAM160B1	HCP5	0.792380525	CLDND1	SNHG5	1
KDELC2	SNHG3	0.71922055	FAM160B1	MCM3AP-AS1	0.883975927	CLDND1	TPT1-AS1	1
ITM2B	LINC00467	0.71867565	FAM160B1	TP53TG1	0.71653952	CLDND1	ZNRD1-AS1	1
POLD3	H19	0.718580695	FAM160B1	TPT1-AS1	0.537662149	CLEC2D	ZNRD1-AS1	1
B4GALT4	DLEU2	0.718559342	FAM160B1	ZNRD1-AS1	0.792163499	CLEC4D	SCARNA9	1
IARS	ZNRD1-AS1	0.71847109	FAM161A	ZNRD1-AS1	0.675553343	CLGN	TP53TG1	1
ORAI3	LINC00467	0.718344568	FAM171B	HCP5	0.986217618	CLIC4	H19	1
TIAM1	H19	0.718027852	FAM173B	RHPN1-AS1	0.852249868	CLIC4	SNHG5	1
GPR63	SNHG5	0.717852488	FAM175A	HCP5	0.964839073	CLIC4	ZNRD1-AS1	1
KBTBD8	MCM3AP-AS1	0.71772649	FAM177A1	SNHG5	0.553780943	CLIC5	RUSC1-AS1	1
FRAT1	KTN1-AS1	0.717545758	FAM184A	SNHG5	0.825750476	CLIP1	MCM3AP-AS1	1
ADAT2	H19	0.717316327	FAM188A	SNHG3	0.84271646	CLIP3	RHPN1-AS1	1
CNOT6L	KTN1-AS1	0.7171074	FAM188A	SNHG5	0.923851737	CLIP4	HCP5	1
XKR8	RHPN1-AS1	0.71699603	FAM189B	HCP5	0.526344359	CLIP4	MCM3AP-AS1	1
FGD4	HCP5	0.716977135	FAM189B	MCM3AP-AS1	0.685212214	CLIP4	SNHG3	1
AIF1L	RHPN1-AS1	0.716904344	FAM189B	ZNRD1-AS1	0.524532818	CLN6	MCM3AP-AS1	1
SH3BP4	MCM3AP-AS1	0.71682011	FAM195A	H19	0.863072741	CLN6	SCARNA9	1
XIRP2	H19	0.71681254	FAM195A	HCP5	0.54785917	CLSPN	MCM3AP-AS1	1
MAP4K4	H19	0.716599627	FAM195A	LINC00467	0.977616319	CLSPN	TP53TG1	1

ATG5	HCP5	0.716572252	FAM195A	MCM3AP-AS1	0.788230497	CLU	MCM3AP-AS1	1
ALDH5A1	SNHG5	0.716467405	FAM195A	ZNRD1-AS1	0.64787435	CMTM7	SNHG3	1
BUB3	SNHG3	0.71633483	FAM198B	SNHG5	0.787542919	CNKSR1	H19	1
OTUD1	RUSC1-AS1	0.715761946	FAM214B	SNHG5	0.517622455	CNKSR1	ZNRD1-AS1	1
ARID4B	DLEU2	0.715668616	FAM217B	HCP5	0.801797067	CNKSR3	DLEU2	1
MYO5C	ZNRD1-AS1	0.715241822	FAM43A	SNHG5	0.695573183	CNKSR3	TPT1-AS1	1
SPAG9	TPT1-AS1	0.715194956	FAM45A	H19	0.853005189	CNKSR3	ZNRD1-AS1	1
CD44	SCARNA9	0.714892119	FAM45A	HCP5	0.648260603	CNNM3	HCP5	1
CD302	MCM3AP-AS1	0.714747347	FAM45A	KTN1-AS1	0.717409423	CNOT6L	HCP5	1
FAM114A1	TP53TG1	0.714597158	FAM45A	MCM3AP-AS1	0.586722719	CNOT6L	KTN1-AS1	1
HIVEP2	HCP5	0.714517451	FAM46A	HCP5	0.963657561	CNOT6L	MCM3AP-AS1	1
ALG2	H19	0.714190076	FAM46A	KTN1-AS1	0.678321156	CNPY4	SNHG3	1
SLC35D2	HCP5	0.713770516	FAM46A	MCM3AP-AS1	0.59269375	COBLL1	HCP5	1
FNDC3B	HCP5	0.713634624	FAM46A	TP53TG1	0.757533834	COBLL1	KTN1-AS1	1
ATXN7L1	HCP5	0.713571036	FAM46C	HCP5	0.599991605	COBLL1	MCM3AP-AS1	1
EXO1	H19	0.713364469	FAM46C	SCARNA9	0.851154679	COBLL1	SNHG3	1
ZSWIM6	HCP5	0.713357502	FAM46C	SNHG5	0.633661711	COL15A1	HCP5	1
EBF1	KTN1-AS1	0.713335189	FAM50B	H19	0.652472618	COL15A1	KTN1-AS1	1
SPG21	ZNRD1-AS1	0.713149928	FAM50B	HCP5	0.779053184	COL15A1	MCM3AP-AS1	1
GAB1	HCP5	0.712536159	FAM53B	H19	0.746913651	COL15A1	SNHG3	1
AHI1	H19	0.712524052	FAM53B	HCP5	0.688857746	COL24A1	ZNRD1-AS1	1
RSBN1	HCP5	0.712343911	FAM53B	ZNRD1-AS1	0.704725122	COL7A1	H19	1
CCDC53	SCARNA9	0.712246475	FAM63B	SNHG3	0.923329789	COL7A1	ZNRD1-AS1	1
DCUN1D5	ZNRD1-AS1	0.711891355	FAM65B	H19	0.612536392	COPB1	DLEU2	1
PTPN1	SCARNA9	0.711751293	FAM65B	HCP5	0.68500128	COTL1	RUSC1-AS1	1
RPS6KA5	H19	0.711703814	FAM65B	ZNRD1-AS1	0.842724462	COX11	RHPN1-AS1	1
TCF4	DLEU2	0.711591588	FAM69B	H19	0.623421391	COX17	RUSC1-AS1	1
BEND7	DLEU2	0.711491774	FAM69B	ZNRD1-AS1	0.793807625	COX7B	HCP5	1
POLR3G	SNHG5	0.711412333	FAM78A	HCP5	0.77645351	CPA3	HCP5	1
NAGPA	SNHG5	0.711365585	FAM78A	TP53TG1	0.86934053	CPA3	TP53TG1	1
KIF23	H19	0.711339562	FAM83D	H19	0.570629801	CPEB2	KTN1-AS1	1
PSMB5	LINC00467	0.711230869	FAM83D	HCP5	0.577668972	CPEB2	MCM3AP-AS1	1
ZNF607	SNHG3	0.710972859	FAM84B	H19	0.887964208	CPEB2	RHPN1-AS1	1
KIF5B	DLEU2	0.710903177	FAM84B	MCM3AP-AS1	0.80122288	CPEB2	TP53TG1	1
ZFC3H1	H19	0.710881089	FAM84B	ZNRD1-AS1	0.669891054	CPEB4	DLEU2	1
EDA2R	MCM3AP-AS1	0.710693066	FAM92A1	SNHG5	0.798290007	CPEB4	H19	1
FERMT2	DLEU2	0.710614664	FANCA	H19	0.806067211	CPEB4	SNHG5	1
TFPI2	SNHG5	0.710477962	FANCA	ZNRD1-AS1	0.767008255	CPEB4	TPT1-AS1	1
DNAJA3	SNHG5	0.710171652	FANCD2	H19	0.844089844	CPEB4	ZNRD1-AS1	1
SEMA6A	HCP5	0.710120079	FANCD2	HCP5	0.563752861	CPNE3	HCP5	1
GUCY1A3	HCP5	0.710111401	FANCM	H19	0.96581151	CPNE3	KTN1-AS1	1
NUMB	MCM3AP-AS1	0.709930677	FANCM	TP53TG1	0.958068021	CPNE3	SCARNA9	1
SCFD1	DLEU2	0.709838957	FASTKD3	KTN1-AS1	0.588922012	CPNE3	SNHG3	1
CREBBP	TPT1-AS1	0.709716282	FASTKD5	HCP5	0.840162575	CPNE8	DLEU2	1
NBEA	ZNRD1-AS1	0.709603252	FASTKD5	MCM3AP-AS1	0.667677499	CPNE8	H19	1
ZBTB44	HCP5	0.709514522	FAT4	HCP5	0.892544427	CPNE8	RUSC1-AS1	1
CCNC	RUSC1-AS1	0.709394738	FBXL5	H19	0.601397972	CPNE8	SNHG5	1
CBS	TPT1-AS1	0.709379859	FBXL5	HCP5	0.865292568	CPNE8	TPT1-AS1	1
TMEM25	HCP5	0.709205135	FBXL5	LINC00467	0.96530706	CPNE8	ZNRD1-AS1	1
CCDC85C	H19	0.709145926	FBXL5	ZNRD1-AS1	0.585750904	CPS1	DLEU2	1
OPRL1	RUSC1-AS1	0.709085788	FBXL6	MCM3AP-AS1	0.62129426	CPS1	H19	1

NUP88	RUSC1-AS1	0.709061819	FBXO4	KTN1-AS1	0.759265276	CPS1	SNHG5	1
TUBGCP4	SNHG5	0.708507631	FBXO4	MCM3AP-AS1	0.635503706	CPS1	TPT1-AS1	1
CD164	HCP5	0.708250476	FBXW7	SNHG3	0.823048632	CPS1	ZNRD1-AS1	1
PIK3CA	HCP5	0.708199537	FCHO1	H19	0.526625588	CPSF3	MCM3AP-AS1	1
GATA3	SNHG3	0.707857116	FCHO1	ZNRD1-AS1	0.95026504	CPSF4	KTN1-AS1	1
NCOR2	H19	0.707823772	FCHO2	HCP5	0.842181613	CPSF4	MCM3AP-AS1	1
TIMM10	SCARNA9	0.707405982	FERMT1	H19	0.759254906	CPVL	SNHG3	1
IL13RA1	HCP5	0.707302081	FERMT1	LINC00467	0.684565859	CRAMP1L	DLEU2	1
HNRNPDL	ZNRD1-AS1	0.707237399	FERMT2	SNHG3	0.77071559	CRAMP1L	H19	1
METAP1	SNHG5	0.707066369	FERMT2	SNHG5	0.637321886	CRAMP1L	ZNRD1-AS1	1
DEK	H19	0.706937626	FGD4	SNHG3	0.819951251	CRBN	HCP5	1
BCOR	DLEU2	0.706888721	FGD4	SNHG5	0.743734981	CRBN	KTN1-AS1	1
IMPA2	SNHG5	0.706806283	FGF2	SNHG5	0.832214945	CRBN	MCM3AP-AS1	1
PRKCH	HCP5	0.706717066	FGF9	SNHG5	0.556828269	CREB3L4	HCP5	1
TRUB1	SCARNA9	0.706643993	FGL2	HCP5	0.928606731	CREB5	HCP5	1
TCF4	HCP5	0.706430788	FHIT	MCM3AP-AS1	0.561127099	CREB5	MCM3AP-AS1	1
CPNE3	KTN1-AS1	0.706336841	FHL1	HCP5	0.517248116	CREB5	SNHG3	1
RBPMS	HCP5	0.70630364	FHOD1	MCM3AP-AS1	0.545215207	CREB5	TP53TG1	1
ELL	ZNRD1-AS1	0.705650164	FICD	RHPN1-AS1	0.811922979	CREBBP	DLEU2	1
LRP1	SNHG3	0.705440005	FIGN	DLEU2	0.737981816	CREBBP	SNHG5	1
SMG5	ZNRD1-AS1	0.704860083	FIGN	HCP5	0.510342616	CREBBP	TPT1-AS1	1
MBOAT2	SNHG5	0.704834143	FIGN	SNHG5	0.71655365	CREBRF	H19	1
NIPAL4	HCP5	0.704183941	FIGNL1	SNHG5	0.890678097	CREBRF	SNHG5	1
TMEM39B	HCP5	0.703993908	FKBP9	SCARNA9	0.957046422	CREBRF	ZNRD1-AS1	1
MDFIC	KTN1-AS1	0.703970865	FKBP9	SNHG5	0.709546659	CREG1	DLEU2	1
RBM8A	RUSC1-AS1	0.703924375	FKTN	H19	0.889083979	CREG1	H19	1
ZFAND2A	MCM3AP-AS1	0.70341057	FKTN	ZNRD1-AS1	0.57502904	CREG1	TPT1-AS1	1
RALA	SCARNA9	0.703306494	FLI1	MCM3AP-AS1	0.593331098	CREG1	ZNRD1-AS1	1
CCDC126	LINC00467	0.703289529	FLI1	TP53TG1	0.973278291	CRELD1	SCARNA9	1
LAMA3	ZNRD1-AS1	0.70324674	FLNA	RHPN1-AS1	0.61692864	CREM	HCP5	1
CKS2	TP53TG1	0.703093312	FLOT2	H19	0.657522253	CREM	MCM3AP-AS1	1
DCTN4	HCP5	0.702905426	FLOT2	MCM3AP-AS1	0.799023667	CREM	SNHG3	1
KSR1	TPT1-AS1	0.702829005	FLOT2	ZNRD1-AS1	0.671564645	CREM	TP53TG1	1
PSD3	KTN1-AS1	0.70199106	FMNL2	H19	0.874673661	CRIM1	H19	1
SLC39A14	KTN1-AS1	0.701984684	FMNL2	HCP5	0.603552871	CRIM1	LINC00467	1
ARFGEF2	DLEU2	0.70185872	FMNL2	KTN1-AS1	0.792114751	CRIM1	RUSC1-AS1	1
CARS	DLEU2	0.70169288	FMNL2	MCM3AP-AS1	0.674856542	CRIM1	SNHG5	1
INPP1	HCP5	0.701635176	FMNL2	ZNRD1-AS1	0.5106746	CRIM1	ZNRD1-AS1	1
GCNT2	ZNRD1-AS1	0.701611589	FN1	RHPN1-AS1	0.864571902	CROT	HCP5	1
KIAA1598	SNHG5	0.701529623	FN3KRP	H19	0.76202648	CROT	TP53TG1	1
MAMLD1	LINC00467	0.70150681	FN3KRP	TP53TG1	0.962744592	CRTAP	KTN1-AS1	1
OCLN	SNHG5	0.701460045	FNBP1	ZNRD1-AS1	0.76270989	CRTAP	MCM3AP-AS1	1
ATP2B4	HCP5	0.701436012	FNDC3B	SNHG3	0.684985109	CRTAP	RHPN1-AS1	1
SCAPER	MCM3AP-AS1	0.701200135	FNDC3B	SNHG5	0.622607149	CRYBG3	H19	1
ZSWIM6	DLEU2	0.701178558	FOSB	HCP5	0.960602638	CRYBG3	ZNRD1-AS1	1
AKTIP	RUSC1-AS1	0.701015662	FOXM1	H19	0.941572157	CSF1	HCP5	1
KIF13A	ZNRD1-AS1	0.700746604	FOXM1	KTN1-AS1	0.799062128	CSF1	KTN1-AS1	1
TP53BP2	DLEU2	0.700363381	FOXM1	MCM3AP-AS1	0.690773701	CSF1	MCM3AP-AS1	1
GPR124	MCM3AP-AS1	0.700158502	FOXN2	HCP5	0.945058777	CSF1	SNHG3	1
ERI1	SNHG3	0.700156666	FOXO3	SNHG3	0.821358756	CSNK1G1	DLEU2	1
ZMAT3	SNHG5	0.70009908	FOXRED1	H19	0.714525952	CSNK1G1	H19	1

UBN2	MCM3AP-AS1	0.699918037	FOXRED1	MCM3AP-AS1	0.660294125	CSNK1G1	TPT1-AS1	1
ABHD6	KTN1-AS1	0.699842925	FOXRED1	ZNRD1-AS1	0.502896323	CSNK1G1	ZNRD1-AS1	1
CLGN	SNHG5	0.699735185	FOXRED2	H19	0.731207723	CSRNP1	RUSC1-AS1	1
BTN3A2	ZNRD1-AS1	0.699557557	FOXRED2	LINC00467	0.834123787	CSRNP1	ZNRD1-AS1	1
RALGPS2	RUSC1-AS1	0.699529715	FOXRED2	ZNRD1-AS1	0.869020072	CSRP1	SNHG3	1
CYP2S1	SNHG3	0.69935685	FPGT	SNHG5	0.523485004	CSRP2BP	MCM3AP-AS1	1
PTPRM	KTN1-AS1	0.699284022	FRMD6	H19	0.881673283	CSTF3	H19	1
PLXNA2	ZNRD1-AS1	0.699271608	FRMD6	LINC00467	0.77233468	CSTF3	ZNRD1-AS1	1
RABEPK	RHPN1-AS1	0.699135469	FRMD6	MCM3AP-AS1	0.774888024	CTDSPL	MCM3AP-AS1	1
FAM171B	SNHG5	0.698854223	FRMD6	TPT1-AS1	0.630469883	CTGF	H19	1
BDH1	LINC00467	0.698846751	FRMD6	ZNRD1-AS1	0.68086905	CTGF	LINC00467	1
HOXA5	SNHG3	0.698490373	FRY	SNHG3	0.75669169	CTGF	SNHG5	1
FNDC3B	SNHG3	0.698039411	FSD1	MCM3AP-AS1	0.872942667	CTGF	ZNRD1-AS1	1
RABGAP1L	DLEU2	0.698023293	FSD1	ZNRD1-AS1	0.760469211	CTNNAL1	MCM3AP-AS1	1
WASF1	MCM3AP-AS1	0.697808631	FUBP3	SNHG3	0.654535705	CTNNBIP1	HCP5	1
KLF6	HCP5	0.697305628	FXYD5	HCP5	0.853666101	CTNNBIP1	KTN1-AS1	1
ALCAM	TP53TG1	0.697076342	FYN	H19	0.632246482	CTNNBIP1	MCM3AP-AS1	1
KLF7	TP53TG1	0.697067731	FYN	KTN1-AS1	0.937134873	CTSD	MCM3AP-AS1	1
CEP128	H19	0.697066762	FYN	ZNRD1-AS1	0.910403785	CTSO	H19	1
RIN2	KTN1-AS1	0.696868244	FZD3	SNHG3	0.943800734	CTSS	H19	1
PLD2	HCP5	0.696637389	FZD5	H19	0.927648438	CTSS	ZNRD1-AS1	1
FNDC3B	DLEU2	0.696630209	FZD5	KTN1-AS1	0.83798113	CTTNBP2NL	DLEU2	1
GTF2IRD2	ZNRD1-AS1	0.696264641	FZD5	MCM3AP-AS1	0.741408642	CTTNBP2NL	SNHG5	1
PLEKHA7	SNHG5	0.696177792	GAB1	H19	0.775240356	CTTNBP2NL	TPT1-AS1	1
BAZ1A	DLEU2	0.696052701	GAB1	HCP5	0.68606777	CUEDC2	SNHG3	1
ACADM	MCM3AP-AS1	0.696006168	GAB1	LINC00467	0.996904281	CUL4B	HCP5	1
YTHDF2	TP53TG1	0.695287106	GAB1	ZNRD1-AS1	0.658343216	CUL4B	MCM3AP-AS1	1
MAN1A1	MCM3AP-AS1	0.695193578	GABARAP	HCP5	0.834197915	CUL4B	SCARNA9	1
ITGA2	TP53TG1	0.695189136	GADD45B	SNHG5	0.673155292	CUL4B	SNHG3	1
RNF19B	SCARNA9	0.695144082	GALK1	KTN1-AS1	0.65691008	CUTC	HCP5	1
OSGEPL1	MCM3AP-AS1	0.694963574	GALK1	MCM3AP-AS1	0.551988673	CUTC	KTN1-AS1	1
NR4A2	DLEU2	0.694928125	GALNT1	SNHG3	0.972483038	CUTC	MCM3AP-AS1	1
C5orf30	SNHG5	0.694771362	GALNT10	SNHG5	0.660201	CUTC	SCARNA9	1
IRF8	HCP5	0.69469952	GAPT	H19	0.620532814	CUX1	HCP5	1
ETFB	SNHG3	0.69463207	GAPT	HCP5	0.7668903	CUX1	MCM3AP-AS1	1
SPIN4	TP53TG1	0.694554708	GAPT	ZNRD1-AS1	0.75975588	CUX1	SCARNA9	1
FHL1	SNHG3	0.694542849	GARS	H19	0.642195645	CUX1	SNHG3	1
MYBL1	DLEU2	0.694297566	GARS	HCP5	0.81493288	CXCL10	MCM3AP-AS1	1
PRKRA	MCM3AP-AS1	0.694060739	GARS	ZNRD1-AS1	0.655082283	CXCL12	KTN1-AS1	1
ARHGAP32	SNHG5	0.693947975	GART	SNHG3	0.958237689	CXCL12	SCARNA9	1
GPT2	TPT1-AS1	0.693301512	GAS2L3	H19	0.89921907	CXCL6	H19	1
TMPO	H19	0.692962873	GAS7	H19	0.768689122	CXCL6	ZNRD1-AS1	1
SEC61A2	TPT1-AS1	0.692901647	GATA2	SNHG5	0.860588609	CXorf23	H19	1
NEK9	HCP5	0.692755179	GATA6	SNHG3	0.854644158	CXorf23	ZNRD1-AS1	1
BIRC2	HCP5	0.692097342	GATA6	SNHG5	0.947780483	CXorf57	ZNRD1-AS1	1
ZHX1	RUSC1-AS1	0.692082423	GATM	KTN1-AS1	0.81211285	CYB5D1	DLEU2	1
RFC4	H19	0.691842416	GBE1	SNHG3	0.983314426	CYB5D1	H19	1
FIGN	SNHG5	0.691699968	GBP1	SNHG5	0.704609675	CYB5D1	ZNRD1-AS1	1
APPL1	RUSC1-AS1	0.691522642	GCA	H19	0.67259637	CYBRD1	HCP5	1
ARHGAP12	SNHG5	0.691407159	GCA	HCP5	0.827947837	CYBRD1	KTN1-AS1	1
STRADB	SNHG3	0.691339881	GCA	ZNRD1-AS1	0.537399648	CYBRD1	MCM3AP-AS1	1

TMEM134	HCP5	0.691297296	GCNT1	KTN1-AS1	0.84070942	CYBRD1	SCARNA9	1
DPYSL2	SNHG5	0.691290554	GCNT1	MCM3AP-AS1	0.829753827	CYBRD1	SNHG3	1
EPT1	H19	0.69096263	GCNT1	ZNRD1-AS1	0.784512852	CYC1	RUSC1-AS1	1
RFC2	H19	0.690876477	GCNT2	KTN1-AS1	0.62592627	CYP2S1	RUSC1-AS1	1
GOLPH3	HCP5	0.690816889	GCNT2	MCM3AP-AS1	0.748719512	CYP2S1	ZNRD1-AS1	1
UBE2G2	H19	0.690788636	GCNT2	RUSC1-AS1	0.747565403	CYP2U1	HCP5	1
GTPBP8	RHPN1-AS1	0.690716737	GCNT2	TPT1-AS1	0.965191373	CYP2U1	MCM3AP-AS1	1
HCFC2	ZNRD1-AS1	0.690691825	GCNT2	ZNRD1-AS1	0.864555006	CYP4V2	HCP5	1
COPS3	H19	0.690334648	GCOM1	SNHG3	0.805634537	CYTH2	SNHG5	1
PLEKHB1	RUSC1-AS1	0.690227777	GCOM1	SNHG5	0.74823554	CYTH3	HCP5	1
RNF19A	RUSC1-AS1	0.690086383	GFI1	RHPN1-AS1	0.527678152	CYTH3	SNHG3	1
DAG1	H19	0.689977314	GFPT2	SNHG5	0.959778914	CYTIP	HCP5	1
SLTM	H19	0.689933036	GGCT	HCP5	0.563062677	DAB2	HCP5	1
ZBTB44	MCM3AP-AS1	0.68983072	GGCT	LINC00467	0.541429264	DAB2	KTN1-AS1	1
CDK6	HCP5	0.689771689	GGH	SNHG3	0.91685966	DAB2	MCM3AP-AS1	1
GLCCI1	RUSC1-AS1	0.689759227	GINS1	H19	0.69162994	DACH1	DLEU2	1
OSBPL1A	ZNRD1-AS1	0.689693268	GINS1	HCP5	0.694879864	DACH1	ZNRD1-AS1	1
GLB1L2	MCM3AP-AS1	0.689690518	GINS4	H19	0.952708388	DACT1	SCARNA9	1
ALCAM	SCARNA9	0.689462717	GLCCI1	H19	0.742809385	DAG1	HCP5	1
CUTC	ZNRD1-AS1	0.689431253	GLCCI1	HCP5	0.775437955	DAG1	MCM3AP-AS1	1
SLC45A4	H19	0.689172207	GLCCI1	KTN1-AS1	0.761706525	DAG1	SCARNA9	1
USP53	MCM3AP-AS1	0.689101897	GLCCI1	MCM3AP-AS1	0.645273889	DAG1	SNHG3	1
UBE2H	HCP5	0.689035384	GLCCI1	TP53TG1	0.942371223	DAGLA	ZNRD1-AS1	1
MANBA	DLEU2	0.688942457	GLCE	H19	0.591891277	DAP3	ZNRD1-AS1	1
NFIA	HCP5	0.688814514	GLCE	KTN1-AS1	0.627671024	DAPP1	DLEU2	1
PARP3	MCM3AP-AS1	0.688680397	GLCE	MCM3AP-AS1	0.506585319	DAPP1	TPT1-AS1	1
ASCC3	SNHG5	0.688285416	GLG1	ZNRD1-AS1	0.914146276	DBI	HCP5	1
TSR1	ZNRD1-AS1	0.688083903	GLI2	TPT1-AS1	0.824136912	DBI	SNHG3	1
TMEM57	MCM3AP-AS1	0.688066787	GLI3	HCP5	0.548029738	DBP	RUSC1-AS1	1
BEND5	HCP5	0.688007025	GLIPR1	MCM3AP-AS1	0.887967118	DCAF17	HCP5	1
FOXO3	HCP5	0.687737883	GLIPR1	RUSC1-AS1	0.519422281	DCAF17	MCM3AP-AS1	1
TBC1D9	KTN1-AS1	0.687737577	GLIPR1	ZNRD1-AS1	0.948822692	DCBLD2	HCP5	1
DNAJC18	MCM3AP-AS1	0.687703513	GLIS3	H19	0.506387065	DCBLD2	KTN1-AS1	1
MYH9	H19	0.687692883	GLIS3	ZNRD1-AS1	0.819311244	DCBLD2	MCM3AP-AS1	1
ZHX3	H19	0.687411214	GLMN	RUSC1-AS1	0.586584416	DCBLD2	SNHG3	1
KCNN4	HCP5	0.687193158	GLRX	MCM3AP-AS1	0.782276959	DCHS1	SNHG5	1
TLR6	KTN1-AS1	0.687154471	GLRX	ZNRD1-AS1	0.660569622	DCLK2	H19	1
UBN2	HCP5	0.686862939	GLRX3	SNHG3	0.958018065	DCLK2	ZNRD1-AS1	1
DHODH	H19	0.68676342	GLTP	SNHG3	0.965225133	DCLRE1A	KTN1-AS1	1
KLF3	LINC00467	0.686498495	GLTP	SNHG5	0.697527094	DCLRE1A	MCM3AP-AS1	1
PDK1	TP53TG1	0.686448393	GLTSCR1	ZNRD1-AS1	0.980724853	DCLRE1B	HCP5	1
KIRREL	HCP5	0.686341661	GM2A	TP53TG1	0.89451334	DCLRE1B	MCM3AP-AS1	1
CELSR3	DLEU2	0.686326418	GMCL1	SNHG3	0.603730808	DCLRE1B	SCARNA9	1
RPS14	SNHG5	0.686244495	GMCL1	TP53TG1	0.541918189	DCLRE1B	SNHG3	1
MAMDC2	SNHG3	0.686081384	GMFB	SNHG3	0.962064202	DCTN4	DLEU2	1
C18orf54	SNHG3	0.686042713	GMFB	SNHG5	0.851261713	DCTN4	H19	1
TMEM65	HCP5	0.685937762	GMPR	HCP5	0.97233203	DCTN4	TPT1-AS1	1
BCL2	KTN1-AS1	0.685919633	GMPS	HCP5	0.820151997	DCTN4	ZNRD1-AS1	1
CYB5D1	ZNRD1-AS1	0.685801111	GNA12	HCP5	0.674297215	DCUN1D3	HCP5	1
MGST1	HCP5	0.685694905	GNAI1	H19	0.719191337	DCUN1D5	KTN1-AS1	1
GOLPH3	SNHG3	0.685473891	GNAI1	HCP5	0.512378813	DCUN1D5	MCM3AP-AS1	1

BACH2	TP53TG1	0.685362273	GNAI1	KTN1-AS1	0.983382684	DCUN1D5	TP53TG1	1
UTRN	ZNRD1-AS1	0.685325753	GNAI1	MCM3AP-AS1	0.940205302	DDB2	MCM3AP-AS1	1
UHRF1BP1L	DLEU2	0.685299777	GNAI1	TPT1-AS1	0.691246228	DDX21	H19	1
ABHD5	HCP5	0.685274746	GNAI1	ZNRD1-AS1	0.853084548	DDX21	RUSC1-AS1	1
WASF1	HCP5	0.685237494	GOLPH3	SNHG3	0.939271678	DDX52	H19	1
PTPRF	TP53TG1	0.685204041	GOLT1B	SNHG3	0.892106565	DDX52	SNHG5	1
MAF	TP53TG1	0.684822531	GORAB	KTN1-AS1	0.838303621	DDX52	ZNRD1-AS1	1
MAMDC2	KTN1-AS1	0.68472404	GORAB	MCM3AP-AS1	0.895944839	DDX56	HCP5	1
ETF1	HCP5	0.684662432	GORAB	ZNRD1-AS1	0.929462494	DDX56	MCM3AP-AS1	1
DPYD	HCP5	0.684636814	GPD1L	HCP5	0.672576223	DDX60	DLEU2	1
DCK	RUSC1-AS1	0.684017027	GPR126	HCP5	0.814265749	DECR2	HCP5	1
ARHGEF3	KTN1-AS1	0.683942939	GPR126	KTN1-AS1	0.922579867	DECR2	SNHG3	1
KIF24	H19	0.683787115	GPR126	MCM3AP-AS1	0.886516944	DEK	HCP5	1
KLF6	SCARNA9	0.68376663	GPR126	TPT1-AS1	0.555620552	DEK	KTN1-AS1	1
IQGAP2	DLEU2	0.683764055	GPR126	ZNRD1-AS1	0.809605307	DEK	MCM3AP-AS1	1
GLA	H19	0.683732066	GPR137B	SNHG5	0.93662277	DENND3	H19	1
ZNF230	SNHG3	0.683597389	GPR137C	HCP5	0.645347185	DENND3	LINC00467	1
MMD	HCP5	0.683461922	GPR137C	TP53TG1	0.63117253	DENND4A	DLEU2	1
ITPR1	MCM3AP-AS1	0.683398759	GPR180	SNHG5	0.629813124	DENND4A	H19	1
PAK1IP1	ZNRD1-AS1	0.683377543	GPR56	HCP5	0.894708846	DENND4A	RUSC1-AS1	1
NFAT5	TPT1-AS1	0.683142712	GPR63	SNHG5	0.521934377	DENND4A	SNHG5	1
EMP1	RUSC1-AS1	0.682997143	GPRIN3	H19	0.862108808	DENND4A	TPT1-AS1	1
DUSP1	RUSC1-AS1	0.682835136	GPSM2	HCP5	0.760130448	DENND4A	ZNRD1-AS1	1
MED16	HCP5	0.682419214	GPSM2	TP53TG1	0.653248294	DENND5A	DLEU2	1
BACE1	SNHG3	0.682060665	GPSM2	ZNRD1-AS1	0.845497781	DENND5A	H19	1
BCL11A	TP53TG1	0.681661142	GPT2	MCM3AP-AS1	0.902380013	DENND5A	ZNRD1-AS1	1
BTN3A3	SNHG3	0.681458423	GPT2	TPT1-AS1	0.996718572	DEPDC1	HCP5	1
BCCIP	H19	0.681380078	GPT2	ZNRD1-AS1	0.956743175	DEPDC1	KTN1-AS1	1
GPR63	MCM3AP-AS1	0.681360599	GRB10	DLEU2	0.707352533	DEPDC1	MCM3AP-AS1	1
UBE2C	H19	0.681243011	GRK5	H19	0.707282011	DEPDC1B	MCM3AP-AS1	1
PREPL	SNHG3	0.681082564	GRK5	KTN1-AS1	0.773569794	DEPDC1B	TP53TG1	1
CPEB4	DLEU2	0.68107319	GRK5	MCM3AP-AS1	0.772386458	DERA	MCM3AP-AS1	1
TXNDC9	ZNRD1-AS1	0.68096843	GRK5	TPT1-AS1	0.793902498	DGKD	DLEU2	1
CCPG1	TPT1-AS1	0.680635876	GRK5	ZNRD1-AS1	0.741524089	DGKD	H19	1
ARHGEF3	HCP5	0.679998062	GRPEL1	SNHG3	0.66136941	DGKD	TPT1-AS1	1
SHISA5	KTN1-AS1	0.679644057	GRPEL2	H19	0.983470125	DGKH	H19	1
SLC2A13	RHPN1-AS1	0.679620035	GSG2	H19	0.656381047	DGKH	ZNRD1-AS1	1
PGAP2	RHPN1-AS1	0.679521711	GSPT2	H19	0.614491308	DHCR24	HCP5	1
KCTD20	HCP5	0.679358903	GSR	SNHG5	0.597690004	DHCR24	MCM3AP-AS1	1
CPNE3	SNHG3	0.67901216	GSTCD	H19	0.627742328	DHCR24	SCARNA9	1
TCTEX1D2	SNHG5	0.678997786	GTF2I	SNHG3	0.744412707	DHCR24	SNHG3	1
CDH2	DLEU2	0.678519482	GTF2IRD2	ZNRD1-AS1	0.668804727	DHFR	MCM3AP-AS1	1
TRIAP1	HCP5	0.678280225	GTPBP8	RHPN1-AS1	0.533219131	DHODH	HCP5	1
SCAI	H19	0.678212285	GTSE1	KTN1-AS1	0.653738534	DHODH	SNHG3	1
BTAF1	H19	0.678177511	GTSE1	MCM3AP-AS1	0.521820978	DHRS4L2	MCM3AP-AS1	1
CPEB2	DLEU2	0.677693291	GUCY1A3	H19	0.551967904	DHX15	H19	1
MED10	KTN1-AS1	0.677667996	GUCY1A3	HCP5	0.50803975	DHX15	RUSC1-AS1	1
RBM8A	MCM3AP-AS1	0.67760346	GUCY1B3	SNHG5	0.819347654	DHX15	SNHG5	1
RBM47	SNHG3	0.677599603	GUF1	LINC00467	0.88660395	DHX15	ZNRD1-AS1	1
FKTN	SNHG5	0.67720261	GULP1	DLEU2	0.743610324	DHX33	MCM3AP-AS1	1
TLR3	SNHG5	0.676681269	GULP1	SCARNA9	0.896872223	DHX33	SNHG3	1

CREB5	HCP5	0.676341876	GULP1	SNHG5	0.933481992	DHX38	ZNRD1-AS1	1
DLGAP3	MCM3AP-AS1	0.675927504	H2AFJ	H19	0.557736862	DIAPH3	MCM3AP-AS1	1
PSME4	H19	0.675728064	H2AFJ	HCP5	0.527065674	DIAPH3	SNHG3	1
FAM171B	HCP5	0.675616033	H2AFJ	ZNRD1-AS1	0.940024378	DIP2C	HCP5	1
CUX1	H19	0.675596667	H2AFY	HCP5	0.825935442	DIP2C	KTN1-AS1	1
PLEKHA2	KTN1-AS1	0.675383531	H6PD	TPT1-AS1	0.986048133	DIP2C	MCM3AP-AS1	1
SLC16A9	KTN1-AS1	0.675274507	HABP4	H19	0.764238527	DIRC2	HCP5	1
ZFC3H1	TPT1-AS1	0.67500617	HABP4	HCP5	0.557641054	DIRC2	MCM3AP-AS1	1
GSG2	H19	0.674928175	HABP4	TPT1-AS1	0.605030293	DIRC2	SNHG3	1
IL12RB2	RHPN1-AS1	0.674393461	HABP4	ZNRD1-AS1	0.792596036	DIS3	HCP5	1
XIRP2	ZNRD1-AS1	0.674336628	HAS2	SNHG3	0.647168249	DIS3	MCM3AP-AS1	1
CAMK2D	MCM3AP-AS1	0.673945679	HAT1	HCP5	0.610522981	DIS3L	HCP5	1
EPHA2	HCP5	0.673780761	HAUS2	MCM3AP-AS1	0.561142176	DIXDC1	ZNRD1-AS1	1
JMJD8	MCM3AP-AS1	0.673250324	HAUS4	HCP5	0.728398019	DKC1	MCM3AP-AS1	1
TUBE1	RUSC1-AS1	0.673135583	HAUS6	LINC00467	0.739399102	DLAT	RHPN1-AS1	1
CARS	TPT1-AS1	0.672758861	HAUS8	H19	0.866136607	DLAT	SNHG3	1
BTBD2	TP53TG1	0.672751509	HBEGF	SNHG3	0.829631844	DLEU1	SNHG3	1
RASGEF1B	MCM3AP-AS1	0.672553528	HBEGF	SNHG5	0.686704155	DLEU1	TP53TG1	1
CTNNBIP1	KTN1-AS1	0.67231547	HBS1L	SNHG5	0.568361159	DLGAP5	HCP5	1
FAM134A	HCP5	0.672268563	HCFC2	H19	0.588348481	DLGAP5	TP53TG1	1
TRIAP1	LINC00467	0.672246513	HCFC2	LINC00467	0.527521839	DMKN	HCP5	1
NVL	H19	0.672075064	HCFC2	MCM3AP-AS1	0.84509539	DMRTA2	HCP5	1
PEAK1	DLEU2	0.671678169	HCFC2	TPT1-AS1	0.89259501	DNA2	SNHG3	1
TCEAL1	HCP5	0.67163466	HCFC2	ZNRD1-AS1	0.841109395	DNA2	TP53TG1	1
HOXA9	HCP5	0.67162717	HEBP1	HCP5	0.934228522	DNAH10	HCP5	1
PHF6	SCARNA9	0.671446401	HECA	H19	0.969550718	DNAJA3	HCP5	1
CD37	RHPN1-AS1	0.671045821	HECA	ZNRD1-AS1	0.522602521	DNAJA3	KTN1-AS1	1
UBE2K	KTN1-AS1	0.670808472	HECTD3	KTN1-AS1	0.907952482	DNAJA3	MCM3AP-AS1	1
SLAMF7	SNHG3	0.670773376	HECTD3	MCM3AP-AS1	0.88197557	DNAJA3	SNHG3	1
CYP2U1	HCP5	0.670744575	HEG1	SNHG5	0.806227364	DNAJA4	MCM3AP-AS1	1
OTUD5	H19	0.67058716	HELLS	SNHG3	0.768659674	DNAJA4	SNHG3	1
PANK1	SNHG3	0.670416975	HERPUD1	SNHG3	0.929408822	DNAJB11	DLEU2	1
TRERF1	MCM3AP-AS1	0.670386142	HEY1	SNHG3	0.780350848	DNAJB11	H19	1
SNAI1	DLEU2	0.67035789	HGF	MCM3AP-AS1	0.914125221	DNAJB11	SNHG5	1
EGLN1	HCP5	0.670148055	HGF	ZNRD1-AS1	0.815445702	DNAJB11	TPT1-AS1	1
KCTD3	KTN1-AS1	0.669945552	HHLA3	LINC00467	0.895987768	DNAJB12	DLEU2	1
TRIM36	SCARNA9	0.66986939	HIST1H2AB	H19	0.618852059	DNAJB12	RUSC1-AS1	1
KIAA0895	SNHG5	0.66974775	HIST1H3B	HCP5	0.536327184	DNAJB12	SNHG5	1
CAB39	ZNRD1-AS1	0.669719449	HIVEP1	KTN1-AS1	0.814795417	DNAJB12	TPT1-AS1	1
DNAJA4	ZNRD1-AS1	0.669682703	HIVEP1	TPT1-AS1	0.923423248	DNAJB12	ZNRD1-AS1	1
ATP2B1	SCARNA9	0.669433702	HIVEP1	ZNRD1-AS1	0.964688385	DNAJB5	RUSC1-AS1	1
DHX15	H19	0.669400524	HIVEP2	RUSC1-AS1	0.766280743	DNAJC12	HCP5	1
PYGL	SNHG5	0.668943204	HLA-C	RUSC1-AS1	0.590065626	DNAJC12	KTN1-AS1	1
RPS6KA6	MCM3AP-AS1	0.668926599	HLA-DQB1	ZNRD1-AS1	0.60868501	DNAJC12	MCM3AP-AS1	1
KIF20B	DLEU2	0.668710935	HLTF	SNHG3	0.887261493	DNAJC18	KTN1-AS1	1
CLIC4	HCP5	0.668483794	HMBOX1	SNHG5	0.503139004	DNAJC18	MCM3AP-AS1	1
ATAD2B	TPT1-AS1	0.66813925	HMGB1	H19	0.528967631	DNAJC18	SNHG3	1
ZDHHC17	TPT1-AS1	0.668081998	HMGB1	HCP5	0.920579197	DNAJC3	DLEU2	1
CRIM1	RUSC1-AS1	0.667654856	HMGB2	HCP5	0.746248375	DNAJC3	H19	1
KAL1	ZNRD1-AS1	0.667569136	HMGB2	KTN1-AS1	0.909382093	DNAJC3	LINC00467	1
JADE3	DLEU2	0.667404659	HMGB2	MCM3AP-AS1	0.831374194	DNAJC3	TPT1-AS1	1

PSAT1	MCM3AP-AS1	0.66737797	HMGB2	TP53TG1	0.839781665	DNAJC3	ZNRD1-AS1	1
GFI1	RHPN1-AS1	0.667256771	HMGB2	ZNRD1-AS1	0.707277074	DNAJC6	KTN1-AS1	1
ZNF695	SCARNA9	0.667197561	HMGXB3	RHPN1-AS1	0.770703917	DNAJC6	MCM3AP-AS1	1
CHUK	H19	0.667154154	HMGXB4	HCP5	0.810071811	DNAJC7	DLEU2	1
LTV1	H19	0.666989869	HMGXB4	KTN1-AS1	0.627829573	DNAJC7	RUSC1-AS1	1
GPR146	SNHG5	0.666970255	HNRNPA3	TP53TG1	0.736952095	DNAJC7	TPT1-AS1	1
GLCE	MCM3AP-AS1	0.666304703	HNRNPAB	H19	0.779964826	DNM3	HCP5	1
DUSP8	DLEU2	0.666299954	HNRNPAB	KTN1-AS1	0.680277139	DNM3	KTN1-AS1	1
FAIM3	HCP5	0.666259532	HNRNPAB	LINC00467	0.964382905	DNM3	MCM3AP-AS1	1
SNRPF	SNHG3	0.666163792	HNRNPAB	MCM3AP-AS1	0.547634251	DNM3	TP53TG1	1
POLA1	H19	0.665920667	HNRNPR	H19	0.9133634	DNMT1	HCP5	1
NEGR1	MCM3AP-AS1	0.665892204	HNRNPR	LINC00467	0.94980171	DOC2A	DLEU2	1
MREG	ZNRD1-AS1	0.665567463	HNRNPR	MCM3AP-AS1	0.752424211	DOC2A	RUSC1-AS1	1
PAPD5	HCP5	0.66523475	HNRNPR	TP53TG1	0.894913068	DOC2A	TPT1-AS1	1
MYO19	H19	0.665014752	HNRNPR	ZNRD1-AS1	0.609138675	DOCK4	HCP5	1
JMJD8	HCP5	0.664776389	HOMER1	SNHG3	0.955607185	DOCK4	KTN1-AS1	1
DDX52	ZNRD1-AS1	0.66468443	HOMER1	SNHG5	0.752324503	DOCK4	MCM3AP-AS1	1
FAM229B	MCM3AP-AS1	0.664524395	HOXA10	HCP5	0.67336936	DOCK4	SNHG3	1
YEATS4	HCP5	0.664512758	HOXA3	H19	0.591609661	DOCK9	H19	1
ELK3	HCP5	0.664112659	HOXA3	KTN1-AS1	0.934667408	DOCK9	RUSC1-AS1	1
SEMA3C	HCP5	0.66394265	HOXA3	MCM3AP-AS1	0.947650265	DOCK9	SNHG5	1
ITGB8	SNHG3	0.663849643	HOXA3	ZNRD1-AS1	0.926537081	DOCK9	ZNRD1-AS1	1
TAB3	DLEU2	0.663842309	HOXA4	ZNRD1-AS1	0.988622219	DONSON	SCARNA9	1
NCOR2	TPT1-AS1	0.663652338	HOXA5	H19	0.58742949	DPF1	MCM3AP-AS1	1
ST8SIA4	HCP5	0.663611121	HOXA5	MCM3AP-AS1	0.981305722	DPM2	HCP5	1
NFIA	TP53TG1	0.663562184	HOXA5	ZNRD1-AS1	0.94713365	DPP9	H19	1
NBEA	H19	0.663512711	HOXA7	H19	0.933242123	DPP9	ZNRD1-AS1	1
KIF5B	ZNRD1-AS1	0.663307538	HOXA7	LINC00467	0.759784484	DPPA4	H19	1
RFX2	TPT1-AS1	0.663167307	HOXA7	MCM3AP-AS1	0.684375737	DPPA4	ZNRD1-AS1	1
RPL13	SNHG5	0.663086333	HOXA7	ZNRD1-AS1	0.573346931	DPY19L3	HCP5	1
ALDH5A1	KTN1-AS1	0.662935351	HOXA9	HCP5	0.558221901	DPYD	DLEU2	1
R3HCC1L	ZNRD1-AS1	0.662090786	HOXA9	LINC00467	0.718753968	DPYD	H19	1
MYO1E	DLEU2	0.662055171	HOXB2	SNHG3	0.808169224	DPYD	TPT1-AS1	1
XPO7	SNHG5	0.661743995	HS3ST3B1	SNHG5	0.828072926	DPYD	ZNRD1-AS1	1
NBEAL1	HCP5	0.661589501	HS6ST2	SNHG5	0.708138646	DPYSL2	DLEU2	1
ZNF624	TP53TG1	0.661414585	HSPA13	SNHG3	0.843070218	DPYSL2	H19	1
RHOB	SCARNA9	0.661401315	HSPA14	SNHG3	0.945814142	DPYSL2	RUSC1-AS1	1
TIMM8B	HCP5	0.661356521	HSPB11	SNHG3	0.83876282	DPYSL2	SNHG5	1
FAM84B	SNHG3	0.661333737	HSPB11	SNHG5	0.542474495	DPYSL2	TPT1-AS1	1
ZNF485	KTN1-AS1	0.661242041	HSPD1	HCP5	0.527216136	DPYSL2	ZNRD1-AS1	1
SP1	TP53TG1	0.660968071	HSPH1	SNHG3	0.916426179	DPYSL3	MCM3AP-AS1	1
OXNAD1	HCP5	0.660937487	HTRA3	H19	0.573598831	DPYSL3	SNHG3	1
BIN1	MCM3AP-AS1	0.660906291	HTRA3	LINC00467	0.547239851	DRAM1	LINC00467	1
SGPL1	SNHG3	0.660656957	IARS	MCM3AP-AS1	0.619214979	DRAM1	RUSC1-AS1	1
ATP2A2	H19	0.659756447	ICA1L	ZNRD1-AS1	0.73909947	DRAM1	SNHG5	1
KCTD14	HCP5	0.659324738	ICAM5	RUSC1-AS1	0.778037659	DRAM1	ZNRD1-AS1	1
ANXA6	RUSC1-AS1	0.659259859	ICMT	H19	0.85281356	DRAP1	SNHG3	1
ABCD3	ZNRD1-AS1	0.65910942	ICMT	HCP5	0.625512806	DRG1	LINC00467	1
PCTP	LINC00467	0.658630129	IFI16	MCM3AP-AS1	0.977366424	DSC2	HCP5	1
MRPL46	HCP5	0.658603279	IFI16	ZNRD1-AS1	0.932836339	DSC2	KTN1-AS1	1
XPO4	MCM3AP-AS1	0.658573242	IFI30	H19	0.551177723	DSC2	MCM3AP-AS1	1

ATP2B1	SNHG3	0.658417362	IFI44L	SNHG3	0.621675762	DSCC1	HCP5	1
RCSD1	SNHG5	0.65837318	IFIT2	SNHG5	0.850955005	DSCC1	SNHG3	1
PREPL	SNHG5	0.658039901	IFIT5	SNHG3	0.997941216	DSEL	MCM3AP-AS1	1
MYO18A	TPT1-AS1	0.657848926	IFITM1	H19	0.977506195	DST	H19	1
PRKACB	KTN1-AS1	0.657542308	IFNAR2	SNHG5	0.958377274	DST	LINC00467	1
ATP1B1	SNHG3	0.657523819	IFRD2	H19	0.584507779	DST	SNHG5	1
MPP2	KTN1-AS1	0.657522796	IGF1	SNHG5	0.782126556	DST	ZNRD1-AS1	1
ATPAF1	RUSC1-AS1	0.657149525	IGF1R	RHPN1-AS1	0.971526226	DSTYK	DLEU2	1
CLUH	ZNRD1-AS1	0.656987625	IGF1R	SNHG3	0.983577458	DSTYK	H19	1
PDCD4	SNHG3	0.656854099	IGF2BP3	HCP5	0.750064037	DSTYK	SNHG5	1
DLAT	SNHG3	0.656710887	IGF2BP3	LINC00467	0.836208764	DSTYK	TPT1-AS1	1
EVI2A	SCARNA9	0.656687467	IGF2BP3	MCM3AP-AS1	0.933713695	DSTYK	ZNRD1-AS1	1
ATG14	MCM3AP-AS1	0.656595451	IGF2BP3	ZNRD1-AS1	0.882708225	DTL	HCP5	1
WASF1	ZNRD1-AS1	0.65657803	IGFBP5	RHPN1-AS1	0.799641497	DTL	KTN1-AS1	1
DNAJA3	SNHG3	0.656557729	IGFBP7	H19	0.665714939	DTL	MCM3AP-AS1	1
SLC12A9	HCP5	0.656427667	IGFBP7	HCP5	0.745602858	DTNA	MCM3AP-AS1	1
KIAA1462	MCM3AP-AS1	0.656285867	IGFBP7	ZNRD1-AS1	0.740209157	DTNA	TP53TG1	1
SOCS5	KTN1-AS1	0.656031265	IL12A	HCP5	0.790114613	DTWD1	HCP5	1
NASP	H19	0.655948717	IL12A	ZNRD1-AS1	0.836119503	DUSP1	SNHG3	1
ERI3	HCP5	0.655941354	IL15	DLEU2	0.530563891	DUSP12	MCM3AP-AS1	1
MXI1	HCP5	0.655900739	IL15	SNHG5	0.620438218	DUSP16	LINC00467	1
SLTM	ZNRD1-AS1	0.6555913	IL1RAP	SNHG3	0.937884805	DUSP18	H19	1
FMNL2	KTN1-AS1	0.655454781	IL21R	RHPN1-AS1	0.860947905	DUSP18	ZNRD1-AS1	1
NEFH	ZNRD1-AS1	0.655229377	IL6	SNHG5	0.78873068	DUSP19	ZNRD1-AS1	1
FAM188A	DLEU2	0.654934861	IMPA2	TP53TG1	0.986294329	DUSP22	H19	1
PTGER4	HCP5	0.654883059	INF2	H19	0.509896484	DUSP22	SNHG5	1
FKBP9	KTN1-AS1	0.654861453	ING2	TP53TG1	0.77193011	DUSP22	ZNRD1-AS1	1
FAM229B	ZNRD1-AS1	0.654844612	INPP5K	RHPN1-AS1	0.94409203	DUSP5	H19	1
PTPRM	MCM3AP-AS1	0.654766785	INPP5K	SNHG5	0.726202407	DUSP5	LINC00467	1
SIX4	KTN1-AS1	0.654613082	INSIG1	SNHG3	0.725741191	DUSP5	RUSC1-AS1	1
EHD1	RHPN1-AS1	0.654564978	INSIG1	SNHG5	0.6028433	DUSP5	SNHG5	1
CLU	MCM3AP-AS1	0.654355666	INSM1	SNHG5	0.815777007	DUSP5	ZNRD1-AS1	1
ZDHHC17	DLEU2	0.653950558	INSR	HCP5	0.817973368	DUSP8	DLEU2	1
THAP11	SNHG3	0.653806239	INSR	SCARNA9	0.801994442	DUSP8	H19	1
SCARB2	RUSC1-AS1	0.653788501	INTS6	MCM3AP-AS1	0.692890578	DUSP8	ZNRD1-AS1	1
ASPH	SNHG3	0.653776171	INTS6	TPT1-AS1	0.931253439	DUT	HCP5	1
CAMK2G	ZNRD1-AS1	0.653638796	INTS6	ZNRD1-AS1	0.824105147	DUT	SCARNA9	1
HSPD1	MCM3AP-AS1	0.653618425	IPO4	SNHG5	0.63083341	DYNC1I2	RUSC1-AS1	1
MLEC	SNHG5	0.653519746	IPPK	SNHG5	0.70121212	DYNLT1	H19	1
WIBG	HCP5	0.653149597	IQCA1	HCP5	0.726614362	DYNLT1	RUSC1-AS1	1
BTBD2	SNHG3	0.653074609	IQGAP1	RHPN1-AS1	0.664544445	DYNLT1	ZNRD1-AS1	1
CLIP1	H19	0.653072873	IQGAP2	H19	0.569567295	DYNLT3	DLEU2	1
RBM47	SNHG5	0.652348129	IQGAP2	HCP5	0.657631004	DYNLT3	H19	1
DUSP18	SCARNA9	0.652172507	IQGAP2	KTN1-AS1	0.986533899	DYNLT3	SNHG5	1
DUSP8	ZNRD1-AS1	0.651857239	IQGAP2	MCM3AP-AS1	0.956542123	DYNLT3	TPT1-AS1	1
NRP2	SNHG3	0.651699817	IQGAP2	TPT1-AS1	0.68471137	DYNLT3	ZNRD1-AS1	1
PPIH	H19	0.651165394	IQGAP2	ZNRD1-AS1	0.884204763	DYRK1B	MCM3AP-AS1	1
RAB31	HCP5	0.651126647	IQGAP3	MCM3AP-AS1	0.73544596	DZIP1	ZNRD1-AS1	1
ADD3	KTN1-AS1	0.651032068	IQGAP3	ZNRD1-AS1	0.604668158	DZIP3	SNHG3	1
GATM	KTN1-AS1	0.650932438	IQSEC1	MCM3AP-AS1	0.998766452	E2F1	HCP5	1
SH3BP4	KTN1-AS1	0.65091997	IQSEC1	TPT1-AS1	0.888939605	E2F2	HCP5	1

MIF4GD	HCP5	0.650749285	IQSEC1	ZNRD1-AS1	0.987934674	E2F3	HCP5	1
SPIRE1	SNHG3	0.650741215	IRF4	SNHG3	0.786802228	E2F3	MCM3AP-AS1	1
SSBP2	SNHG3	0.650693345	IRF4	SNHG5	0.801467839	E2F3	SCARNA9	1
PYCRL	MCM3AP-AS1	0.65046551	IRF8	KTN1-AS1	0.565295638	E2F5	HCP5	1
CDH2	SCARNA9	0.650292794	IRF8	MCM3AP-AS1	0.641659957	E2F5	KTN1-AS1	1
FANCD2	H19	0.650222663	IRF8	ZNRD1-AS1	0.707218027	E2F5	SNHG3	1
EMB	HCP5	0.650186451	ISCA2	HCP5	0.775213205	E2F5	TP53TG1	1
EXO5	ZNRD1-AS1	0.649978524	ITGA2	SNHG3	0.980356361	E2F7	HCP5	1
RAN	HCP5	0.649968897	ITGA4	HCP5	0.849736972	E2F7	MCM3AP-AS1	1
KIF5B	TPT1-AS1	0.649423176	ITGA4	ZNRD1-AS1	0.759088842	E2F7	SNHG3	1
PANK1	HCP5	0.649315898	ITGA6	H19	0.862645383	E2F8	KTN1-AS1	1
BAZ2B	DLEU2	0.649206265	ITGAV	SNHG3	0.749854856	E2F8	MCM3AP-AS1	1
VPS26B	HCP5	0.649160762	ITGAV	SNHG5	0.834669738	EBF1	HCP5	1
SHB	SCARNA9	0.649001199	ITGB3	SNHG5	0.955723075	EBF1	KTN1-AS1	1
C20orf194	MCM3AP-AS1	0.648978145	ITGB8	SNHG5	0.848829351	EBF1	MCM3AP-AS1	1
WDR54	KTN1-AS1	0.648708088	ITM2B	RHPN1-AS1	0.802036133	EBF1	SNHG3	1
CCDC71L	TP53TG1	0.648641159	ITM2B	SNHG3	0.949629313	EBF1	TP53TG1	1
FAM43A	MCM3AP-AS1	0.648318385	ITM2B	SNHG5	0.838244649	EBF4	H19	1
GARS	HCP5	0.648312633	ITPK1	H19	0.773949242	EBF4	ZNRD1-AS1	1
ZEB1	MCM3AP-AS1	0.648141559	ITPR1	MCM3AP-AS1	0.68467986	ECE1	H19	1
PPAP2B	SNHG5	0.647971078	ITPR1	RUSC1-AS1	0.676725839	ECE1	ZNRD1-AS1	1
KIF20A	DLEU2	0.647949513	ITPR1	ZNRD1-AS1	0.801743258	ECH1	SNHG3	1
SRSF1	ZNRD1-AS1	0.647297089	ITPR3	TPT1-AS1	0.510216018	ECHDC1	KTN1-AS1	1
ZDHHC17	HCP5	0.647137719	ITPRIP	H19	0.78160423	ECHDC1	MCM3AP-AS1	1
BLOC1S1	SNHG3	0.646783789	ITPRIP	TP53TG1	0.90918547	ECHDC1	SNHG3	1
RP9	HCP5	0.646736293	IVNS1ABP	TP53TG1	0.572501021	ECHDC1	TP53TG1	1
AIM1	HCP5	0.646599677	JAG2	KTN1-AS1	0.509150442	ECHDC2	TP53TG1	1
ADAM28	MCM3AP-AS1	0.646448747	JAG2	TPT1-AS1	0.934336912	ECT2	HCP5	1
ARL5B	DLEU2	0.645795158	JAG2	ZNRD1-AS1	0.755370844	ECT2	MCM3AP-AS1	1
SDCCAG8	RUSC1-AS1	0.64545146	JARID2	KTN1-AS1	0.661367128	ECT2	SNHG3	1
CYBRD1	SNHG3	0.645075241	JARID2	MCM3AP-AS1	0.732905796	ECT2	TP53TG1	1
DACH1	SNHG3	0.644886525	JARID2	TPT1-AS1	0.931207883	EDA2R	HCP5	1
ZNF77	MCM3AP-AS1	0.644770327	JARID2	ZNRD1-AS1	0.789212124	EDA2R	KTN1-AS1	1
SIDT1	MCM3AP-AS1	0.644664701	JMJD6	SNHG3	0.754182319	EDA2R	MCM3AP-AS1	1
CA8	MCM3AP-AS1	0.644425853	JMJD8	HCP5	0.592237736	EDARADD	HCP5	1
CBX5	KTN1-AS1	0.644381306	JMJD8	MCM3AP-AS1	0.925589042	EDARADD	MCM3AP-AS1	1
SYT1	ZNRD1-AS1	0.644289494	JUP	MCM3AP-AS1	0.948034784	EDARADD	SCARNA9	1
ZHX2	RUSC1-AS1	0.644273383	JUP	TPT1-AS1	0.811009153	EDEM1	HCP5	1
CADM1	SCARNA9	0.644193809	JUP	ZNRD1-AS1	0.892267776	EDEM1	KTN1-AS1	1
VEGFA	TPT1-AS1	0.644169183	KAL1	HCP5	0.945496452	EDEM1	MCM3AP-AS1	1
IL15	DLEU2	0.644165994	KAL1	SCARNA9	0.668082148	EEPD1	HCP5	1
PTPDC1	SNHG3	0.643369695	KANK1	SNHG3	0.90209997	EFNA3	HCP5	1
CYP4V2	HCP5	0.64321155	KATNAL1	H19	0.642283429	EFNA3	MCM3AP-AS1	1
KIF13B	H19	0.642889586	KATNAL1	HCP5	0.827475658	EFNA3	SCARNA9	1
NRP2	HCP5	0.642464523	KATNAL1	KTN1-AS1	0.555815891	EFNA4	H19	1
KLF3	RUSC1-AS1	0.642440982	KBTBD6	SCARNA9	0.83325419	EGLN1	HCP5	1
SYAP1	ZNRD1-AS1	0.642410526	KBTBD6	SNHG3	0.528013577	EGLN1	MCM3AP-AS1	1
NIPSNAP1	HCP5	0.64185105	KBTBD8	SNHG3	0.957584836	EGLN3	HCP5	1
ENOX1	SNHG5	0.641828375	KBTBD8	SNHG5	0.698463155	EGLN3	KTN1-AS1	1
PALLD	SNHG5	0.64179525	KCNA3	H19	0.608107874	EGLN3	MCM3AP-AS1	1
PTPN13	ZNRD1-AS1	0.641680138	KCNK5	SCARNA9	0.863405959	EGR2	H19	1

HS2ST1	HCP5	0.641557517	KCTD12	HCP5	0.908565625	EGR2	SNHG5	1
ADAM19	KTN1-AS1	0.641537423	KCTD12	LINC00467	0.778134382	EGR2	ZNRD1-AS1	1
LRRC61	HCP5	0.641522288	KCTD12	TP53TG1	0.807151361	EHD1	SNHG5	1
SLTM	TPT1-AS1	0.641502122	KCTD14	HCP5	0.946545661	EHD1	ZNRD1-AS1	1
KAL1	TPT1-AS1	0.641496155	KCTD15	H19	0.983658026	EHD3	HCP5	1
MCF2L	HCP5	0.641108021	KCTD20	SNHG5	0.572407867	EHD4	SNHG3	1
VRK1	H19	0.641051555	KCTD3	KTN1-AS1	0.510094203	EHMT2	HCP5	1
SLC24A1	ZNRD1-AS1	0.640948963	KCTD3	MCM3AP-AS1	0.609401365	EIF2AK4	HCP5	1
MRPL40	HCP5	0.640692573	KCTD3	TPT1-AS1	0.901528332	EIF2B2	HCP5	1
TRIM59	KTN1-AS1	0.640658989	KCTD3	ZNRD1-AS1	0.703196319	EIF2B2	MCM3AP-AS1	1
SORL1	HCP5	0.640630419	KCTD9	TPT1-AS1	0.532388215	EIF2B2	SCARNA9	1
PEBP1	RUSC1-AS1	0.640545622	KDELC1	SNHG3	0.970570319	EIF2S1	HCP5	1
RHOB	DLEU2	0.640305982	KDELC2	SNHG3	0.730504974	EIF2S2	H19	1
RPS6KA2	DLEU2	0.640133056	KDM2B	H19	0.519775446	EIF2S2	ZNRD1-AS1	1
SDC1	H19	0.640035425	KDM2B	KTN1-AS1	0.953771957	EIF3J	HCP5	1
EXPH5	HCP5	0.640003245	KDM2B	TPT1-AS1	0.917289722	EIF3J	MCM3AP-AS1	1
ATP2B4	SNHG5	0.639934553	KDM6B	TPT1-AS1	0.744287598	EIF3J	SNHG3	1
AMIGO2	KTN1-AS1	0.63945463	KIAA0020	SNHG3	0.795317889	EIF4E3	H19	1
SUFU	HCP5	0.639424837	KIAA0101	H19	0.709271644	EIF4E3	ZNRD1-AS1	1
CEBPG	SNHG3	0.639422974	KIAA0101	LINC00467	0.651369945	EIF5	SNHG5	1
CAT	KTN1-AS1	0.639369179	KIAA0101	MCM3AP-AS1	0.858076631	EIF5	ZNRD1-AS1	1
DCBLD2	SNHG5	0.639204221	KIAA0101	TPT1-AS1	0.823745279	EIF5B	H19	1
GPT2	HCP5	0.639161285	KIAA0101	ZNRD1-AS1	0.819284469	EIF5B	SNHG5	1
USP16	TPT1-AS1	0.638962394	KIAA0226	TPT1-AS1	0.543106951	ELK3	HCP5	1
CEP152	TPT1-AS1	0.638936685	KIAA0430	H19	0.569608662	ELK3	KTN1-AS1	1
MPPED2	MCM3AP-AS1	0.638837733	KIAA0430	TPT1-AS1	0.755996588	ELK3	MCM3AP-AS1	1
NAA15	HCP5	0.63878749	KIAA0430	ZNRD1-AS1	0.921578016	ELK3	SNHG3	1
SH2B3	DLEU2	0.638737141	KIAA0895	SNHG3	0.548305745	ELL	MCM3AP-AS1	1
ARL9	ZNRD1-AS1	0.638600569	KIAA0922	HCP5	0.78454463	ELL2	DLEU2	1
MED15	H19	0.638436588	KIAA1109	RUSC1-AS1	0.684204773	ELL2	H19	1
LIN9	SNHG3	0.638255328	KIAA1147	DLEU2	0.542602375	ELL2	TPT1-AS1	1
NEK9	SNHG3	0.637900949	KIAA1147	SCARNA9	0.503491264	ELL2	ZNRD1-AS1	1
MASTL	H19	0.637900863	KIAA1161	H19	0.673759075	ELOVL6	KTN1-AS1	1
ALDH16A1	SNHG3	0.637863964	KIAA1161	MCM3AP-AS1	0.746674342	ELOVL6	MCM3AP-AS1	1
TPTE2	H19	0.637823131	KIAA1161	ZNRD1-AS1	0.607447561	ELOVL6	SNHG3	1
MPP6	RUSC1-AS1	0.637764811	KIAA1211	MCM3AP-AS1	0.93088578	ELP4	DLEU2	1
ATP2B1	DLEU2	0.63764286	KIAA1211	TPT1-AS1	0.970757455	ELP4	H19	1
KCTD12	LINC00467	0.637533149	KIAA1211	ZNRD1-AS1	0.98559182	EMB	HCP5	1
PDGFD	HCP5	0.63748219	KIAA1244	SNHG3	0.914504033	EME1	SCARNA9	1
NSUN7	HCP5	0.637460833	KIAA1462	MCM3AP-AS1	0.914889803	EMG1	HCP5	1
FAM46C	KTN1-AS1	0.637361445	KIAA1462	RUSC1-AS1	0.614994569	EMG1	SCARNA9	1
PTPRM	SNHG3	0.636989478	KIAA1462	ZNRD1-AS1	0.977639806	EML4	HCP5	1
ITGA6	HCP5	0.636805491	KIAA1524	TP53TG1	0.844350521	EML4	KTN1-AS1	1
CECR1	HCP5	0.636710454	KIAA1598	SNHG5	0.823865379	EML4	SCARNA9	1
RNF150	SNHG3	0.636705121	KIF11	H19	0.878071783	EMP1	DLEU2	1
DBP	RUSC1-AS1	0.636353961	KIF11	HCP5	0.594384413	EMP1	H19	1
ENOX1	HCP5	0.636041318	KIF11	KTN1-AS1	0.635551199	EMP1	RUSC1-AS1	1
FAM46A	TP53TG1	0.635890994	KIF11	LINC00467	0.938488437	EMP1	SNHG5	1
NAA38	SNHG5	0.635600835	KIF13A	MCM3AP-AS1	0.757085593	EMP1	TPT1-AS1	1
FRMD6	SNHG3	0.634974662	KIF13A	ZNRD1-AS1	0.874094176	ENAH	HCP5	1
RAB11FIP4	HCP5	0.634806524	KIF18B	MCM3AP-AS1	0.6574406	ENAH	MCM3AP-AS1	1

DPYSL2	SCARNA9	0.63472768	KIF20A	KTN1-AS1	0.674880192	ENAH	SNHG3	1
HCFC2	TPT1-AS1	0.634682117	KIF21A	HCP5	0.81901064	ENC1	HCP5	1
RASSF6	SCARNA9	0.634465862	KIF22	MCM3AP-AS1	0.529629408	ENC1	KTN1-AS1	1
LRRK2	LINC00467	0.634392387	KIF23	H19	0.930358866	ENC1	MCM3AP-AS1	1
SLC2A13	SNHG5	0.634372276	KIF23	HCP5	0.506490299	ENC1	SNHG3	1
HIVEP2	RUSC1-AS1	0.634320812	KIF23	MCM3AP-AS1	0.555156645	ENC1	TP53TG1	1
TYMS	H19	0.634204219	KIF23	TP53TG1	0.983819192	ENOX1	HCP5	1
DLEU1	TP53TG1	0.633903052	KIF24	H19	0.972548268	ENOX1	SCARNA9	1
C9orf40	SNHG3	0.633860607	KIF24	MCM3AP-AS1	0.63554172	ENPP2	HCP5	1
CDCA7L	SNHG5	0.633756168	KIF2A	HCP5	0.758672271	ENPP2	MCM3AP-AS1	1
SBF2	SNHG3	0.63375003	KIF2A	KTN1-AS1	0.911401332	ENPP2	TP53TG1	1
WDR47	TPT1-AS1	0.63326495	KIF2A	MCM3AP-AS1	0.836207096	ENPP4	DLEU2	1
JAG2	DLEU2	0.633012665	KIF2A	ZNRD1-AS1	0.715226226	ENPP4	H19	1
B3GNT5	HCP5	0.632807817	KIF2C	H19	0.878982938	ENPP4	SNHG5	1
VKORC1L1	KTN1-AS1	0.63267773	KIF2C	LINC00467	0.783606523	ENPP4	TPT1-AS1	1
BAZ2B	TPT1-AS1	0.63266113	KIF3A	SNHG3	0.919682007	ENPP4	ZNRD1-AS1	1
REXO1	DLEU2	0.632608136	KIF5C	SNHG3	0.628479517	ENTPD1	HCP5	1
ZXDA	HCP5	0.632504881	KIFAP3	TP53TG1	0.610891718	ENTPD1	MCM3AP-AS1	1
TUFT1	RUSC1-AS1	0.632460444	KIRREL	HCP5	0.85206431	ENTPD5	KTN1-AS1	1
SLC16A9	TP53TG1	0.632441849	KLC2	ZNRD1-AS1	0.542858156	EPB41L2	DLEU2	1
SEMA6A	SNHG3	0.632420767	KLF11	DLEU2	0.866098742	EPB41L2	H19	1
PSAT1	ZNRD1-AS1	0.632401947	KLF11	SNHG5	0.665361163	EPB41L2	TPT1-AS1	1
ELL	MCM3AP-AS1	0.632353223	KLF12	HCP5	0.987058022	EPB41L2	ZNRD1-AS1	1
EHD2	SCARNA9	0.632181662	KLF12	MCM3AP-AS1	0.584916543	EPB41L5	DLEU2	1
FOXM1	H19	0.632069558	KLF12	TP53TG1	0.657251592	EPB41L5	H19	1
SFXN3	HCP5	0.631894652	KLF3	HCP5	0.584696812	EPB41L5	TPT1-AS1	1
RNF19A	MCM3AP-AS1	0.631442542	KLF3	KTN1-AS1	0.949980255	EPB41L5	ZNRD1-AS1	1
CREBBP	DLEU2	0.631441852	KLF3	LINC00467	0.708783781	EPCAM	DLEU2	1
CASP4	LINC00467	0.631421469	KLF3	MCM3AP-AS1	0.976548291	EPCAM	SNHG5	1
PNRC1	TP53TG1	0.631309837	KLF3	ZNRD1-AS1	0.970032841	EPHA2	HCP5	1
TMEM57	ZNRD1-AS1	0.631221823	KLF5	SNHG3	0.823518502	EPHB4	HCP5	1
MBOAT1	MCM3AP-AS1	0.631178561	KLF5	SNHG5	0.921976288	EPHB4	MCM3AP-AS1	1
APOL2	MCM3AP-AS1	0.631141901	KLF6	SNHG5	0.73355928	EPN2	HCP5	1
ASAP2	H19	0.630724357	KLF7	H19	0.566284123	EPN2	MCM3AP-AS1	1
SRSF7	H19	0.630625853	KLF7	MCM3AP-AS1	0.975074982	EPN2	SNHG3	1
AP3S1	SNHG3	0.630551143	KLF7	ZNRD1-AS1	0.95117083	EPS8	H19	1
BRIX1	DLEU2	0.630541088	KLHDC8B	MCM3AP-AS1	0.543509642	EPS8	SNHG5	1
SKA2	H19	0.630444553	KLHL2	SNHG3	0.958200355	EPS8	ZNRD1-AS1	1
ZNF652	RUSC1-AS1	0.630247541	KLHL2	SNHG5	0.75378423	EPSTI1	H19	1
LRCH2	MCM3AP-AS1	0.630215333	KLHL24	TPT1-AS1	0.697922487	EPSTI1	SNHG5	1
ADSS	HCP5	0.63019172	KLHL3	LINC00467	0.897748218	EPSTI1	ZNRD1-AS1	1
PCMTD1	TPT1-AS1	0.630155656	KLHL3	ZNRD1-AS1	0.781643394	EPT1	MCM3AP-AS1	1
NCAPD2	H19	0.630036659	KPNA2	SNHG3	0.630761761	EPT1	SNHG3	1
RHOBTB1	HCP5	0.629841564	KSR1	TPT1-AS1	0.969635756	ERG	DLEU2	1
RIOK3	DLEU2	0.62959087	LACTB	DLEU2	0.871244607	ERG	H19	1
BAZ1A	ZNRD1-AS1	0.629588992	LAMA2	HCP5	0.634847602	ERG	TPT1-AS1	1
LMBR1L	TPT1-AS1	0.629485301	LAMA5	H19	0.757154569	ERG	ZNRD1-AS1	1
SLC35F1	HCP5	0.629082661	LAMC1	H19	0.924881666	ERI1	HCP5	1
IGF1R	MCM3AP-AS1	0.628999858	LAMC1	TP53TG1	0.818544027	ERI1	KTN1-AS1	1
CASP7	KTN1-AS1	0.628848771	LAMP3	HCP5	0.668556928	ERI1	MCM3AP-AS1	1
ARID4B	ZNRD1-AS1	0.628780134	LANCL1	H19	0.626578402	ERI1	SNHG3	1

TFPI	TP53TG1	0.628651601	LANCL1	HCP5	0.865306877	ERI1	TP53TG1	1
DSTYK	DLEU2	0.62831217	LANCL1	KTN1-AS1	0.669307856	ERI2	HCP5	1
QDPR	TP53TG1	0.628297619	LANCL1	MCM3AP-AS1	0.550122982	ERI2	KTN1-AS1	1
CUL4B	SCARNA9	0.628261888	LARP6	H19	0.570073683	ERI2	MCM3AP-AS1	1
SMAD5	SNHG3	0.628114715	LARP6	LINC00467	0.946077753	ERI2	SNHG3	1
CCNC	SNHG3	0.627880027	LCLAT1	H19	0.551757494	ERI3	HCP5	1
TMEM50B	ZNRD1-AS1	0.627877024	LCLAT1	HCP5	0.755100552	ERICH1	DLEU2	1
SESN2	HCP5	0.627810027	LDB1	ZNRD1-AS1	0.995547486	ERICH1	H19	1
LIN9	SNHG5	0.627806553	LETMD1	ZNRD1-AS1	0.845117709	ERICH1	SNHG5	1
RAPGEF1	H19	0.627440252	LFNG	MCM3AP-AS1	0.843416346	ERICH1	ZNRD1-AS1	1
CDKN1B	KTN1-AS1	0.627034947	LGALSL	SNHG5	0.960281492	ERLIN2	HCP5	1
KIF5C	SNHG3	0.626871428	LGMN	ZNRD1-AS1	0.598420186	ERLIN2	KTN1-AS1	1
PDE8A	ZNRD1-AS1	0.626382776	LHFPL2	H19	0.569332898	ERLIN2	MCM3AP-AS1	1
ID1	HCP5	0.626207743	LHFPL2	HCP5	0.876176469	ERLIN2	SNHG3	1
PDLIM1	SNHG5	0.626004947	LHFPL2	KTN1-AS1	0.54814977	ERMP1	H19	1
ZNF493	KTN1-AS1	0.625986647	LHX6	HCP5	0.761603533	ERMP1	SNHG5	1
RRAGD	TP53TG1	0.625889124	LIMCH1	LINC00467	0.663258093	ERMP1	ZNRD1-AS1	1
SEC61A2	MCM3AP-AS1	0.625698491	LIMCH1	MCM3AP-AS1	0.685522118	ERO1L	HCP5	1
PARP16	SNHG5	0.625633952	LIMK2	SNHG3	0.821247437	ERO1L	KTN1-AS1	1
PRDM1	SNHG3	0.625575978	LIN9	H19	0.889814215	ERO1L	TP53TG1	1
ZNF75A	ZNRD1-AS1	0.625044194	LIN9	TP53TG1	0.989556902	ERP29	MCM3AP-AS1	1
ZNF492	DLEU2	0.624970405	LIPT1	HCP5	0.891916624	ERP29	SNHG3	1
MARCKS	KTN1-AS1	0.624948305	LIPT2	H19	0.722610847	ESCO2	HCP5	1
ZBTB1	DLEU2	0.624928163	LIPT2	HCP5	0.784442971	ESYT1	MCM3AP-AS1	1
TYSND1	HCP5	0.624926914	LIPT2	KTN1-AS1	0.638463834	ESYT2	MCM3AP-AS1	1
PCMTD1	HCP5	0.624684221	LIX1L	HCP5	0.977452322	ESYT2	SCARNA9	1
ZHX1	MCM3AP-AS1	0.624596704	LLGL1	LINC00467	0.724918969	ETAA1	HCP5	1
SMYD2	KTN1-AS1	0.624282492	LMBR1L	DLEU2	0.508100738	ETAA1	SNHG3	1
STK32B	HCP5	0.624081411	LMBR1L	TPT1-AS1	0.58895978	ETF1	HCP5	1
IQSEC1	TP53TG1	0.623915861	LMNB1	H19	0.682712274	ETF1	KTN1-AS1	1
ATG5	TPT1-AS1	0.623656228	LMNB1	HCP5	0.653743397	ETF1	MCM3AP-AS1	1
8-Sep	SNHG3	0.623553824	LMO4	SNHG3	0.840875434	ETF1	SNHG3	1
PHF5A	SNHG3	0.623289174	LONRF2	ZNRD1-AS1	0.521154333	ETFB	SNHG3	1
AGER	ZNRD1-AS1	0.623136583	LOX	HCP5	0.889678646	ETS1	H19	1
EIF2S2	TP53TG1	0.623106505	LOXL1	H19	0.698270282	ETS1	LINC00467	1
GPR126	SNHG5	0.623084747	LPAR6	SNHG3	0.709411571	ETS1	ZNRD1-AS1	1
KANK1	MCM3AP-AS1	0.623043846	LPCAT4	HCP5	0.543396324	ETV4	H19	1
UBN2	ZNRD1-AS1	0.622772446	LPP	SNHG3	0.653682599	ETV5	H19	1
EXTL2	ZNRD1-AS1	0.622757945	LPP	SNHG5	0.609110528	ETV5	LINC00467	1
SEMA6A	LINC00467	0.622469286	LRCH2	DLEU2	0.506888747	ETV5	SNHG5	1
MEF2A	ZNRD1-AS1	0.62231154	LRCH2	SNHG3	0.608591765	ETV5	ZNRD1-AS1	1
DST	ZNRD1-AS1	0.622161529	LRIG1	SNHG3	0.75579032	EWSR1	DLEU2	1
SPRED1	LINC00467	0.621895019	LRIG1	SNHG5	0.769023809	EWSR1	TPT1-AS1	1
CECR1	RHPN1-AS1	0.621863395	LRMP	HCP5	0.599387568	EXOC4	HCP5	1
AGMAT	SNHG5	0.621808131	LRP1	DLEU2	0.584828456	EXOC4	MCM3AP-AS1	1
ETS1	HCP5	0.621528988	LRP1	HCP5	0.831754631	EXOC4	SNHG3	1
ATXN7L1	LINC00467	0.621368602	LRP12	SNHG3	0.920684155	EXOSC1	ZNRD1-AS1	1
DGKH	ZNRD1-AS1	0.621228927	LRP2BP	H19	0.850806064	EXOSC5	HCP5	1
TAB2	DLEU2	0.621193913	LRP5	KTN1-AS1	0.802685499	EXOSC8	HCP5	1
IGF2BP3	SNHG5	0.621141867	LRP5	MCM3AP-AS1	0.774932483	EXOSC8	SNHG3	1
NAV1	ZNRD1-AS1	0.621105791	LRP5	ZNRD1-AS1	0.712648086	EXOSC9	MCM3AP-AS1	1

SP1	KTN1-AS1	0.6208247	LRRC16A	H19	0.698611632	EXPH5	HCP5	1
THOP1	H19	0.620506904	LRRC16A	HCP5	0.684171914	EXPH5	TP53TG1	1
GTF2IRD2B	ZNRD1-AS1	0.620431609	LRRC16A	KTN1-AS1	0.948467412	EXTL2	HCP5	1
PSMD12	SNHG3	0.62030674	LRRC16A	MCM3AP-AS1	0.882956645	EXTL3	MCM3AP-AS1	1
TSHZ1	H19	0.620207659	LRRC16A	ZNRD1-AS1	0.771631661	EYA3	SNHG5	1
RAVER2	HCP5	0.620013674	LRRC61	H19	0.83572786	EZH1	H19	1
RALB	H19	0.619972939	LRRK2	H19	0.544731836	EZH1	ZNRD1-AS1	1
ST3GAL6	HCP5	0.619962134	LRRK2	LINC00467	0.840257258	EZH2	HCP5	1
ADD3	SNHG5	0.619959562	LRRK2	TP53TG1	0.880817813	EZH2	MCM3AP-AS1	1
KCTD9	TPT1-AS1	0.619907577	LSM5	H19	0.648437588	EZH2	SNHG3	1
MARS	H19	0.619665711	LSM5	HCP5	0.771659108	EZH2	TP53TG1	1
NT5DC3	KTN1-AS1	0.619608665	LTBP3	MCM3AP-AS1	0.999249791	F11R	HCP5	1
TMC7	SNHG3	0.619541907	LTV1	H19	0.923079031	FABP5	MCM3AP-AS1	1
RGMA	H19	0.619528358	LURAP1L	SNHG5	0.608796573	FADD	KTN1-AS1	1
SSBP2	KTN1-AS1	0.619403868	LY75	ZNRD1-AS1	0.864663635	FADD	MCM3AP-AS1	1
DHRS4L2	MCM3AP-AS1	0.618634483	LYRM1	H19	0.628772396	FADD	RHPN1-AS1	1
RWDD2B	HCP5	0.618556343	LYRM1	HCP5	0.796898952	FAIM3	HCP5	1
MKNK1	MCM3AP-AS1	0.618516305	LYRM1	ZNRD1-AS1	0.708368115	FAM102A	HCP5	1
CARD6	SNHG5	0.618419363	LYRM5	MCM3AP-AS1	0.94098749	FAM102A	KTN1-AS1	1
PGAP2	HCP5	0.618310529	LYRM5	ZNRD1-AS1	0.879850401	FAM102A	MCM3AP-AS1	1
PATZ1	RUSC1-AS1	0.618266224	LYST	SNHG5	0.518342686	FAM102B	HCP5	1
PXK	HCP5	0.617721081	MAF	DLEU2	0.858135018	FAM102B	SNHG3	1
FAM83D	H19	0.617690689	MAGT1	SNHG3	0.888857799	FAM102B	TP53TG1	1
PAIP2B	HCP5	0.617576869	MAMDC2	H19	0.764719984	FAM107B	H19	1
BCL6	ZNRD1-AS1	0.617495905	MAMDC2	KTN1-AS1	0.882794813	FAM107B	RUSC1-AS1	1
ACTN1	H19	0.617412651	MAMDC2	LINC00467	0.719188016	FAM107B	ZNRD1-AS1	1
C4orf19	DLEU2	0.617309103	MAMDC2	MCM3AP-AS1	0.86168386	FAM110B	SNHG3	1
MAP3K3	HCP5	0.617148928	MAMDC2	TP53TG1	0.555941373	FAM111B	KTN1-AS1	1
FOXN2	SNHG3	0.617143297	MAMDC2	TPT1-AS1	0.777826552	FAM111B	MCM3AP-AS1	1
MTL5	MCM3AP-AS1	0.616952382	MAMDC2	ZNRD1-AS1	0.803807334	FAM114A1	H19	1
CLGN	TP53TG1	0.616801728	MAML3	ZNRD1-AS1	0.980490239	FAM114A1	ZNRD1-AS1	1
CA11	SNHG5	0.616468779	MAMLD1	DLEU2	0.828488719	FAM117A	HCP5	1
HLA-DPB1	SNHG5	0.616399274	MAMLD1	SNHG5	0.935957212	FAM117B	H19	1
SOCS5	MCM3AP-AS1	0.616377246	MAN1C1	H19	0.961579069	FAM117B	LINC00467	1
SHISA5	TP53TG1	0.616284748	MANBA	KTN1-AS1	0.983234311	FAM117B	SNHG5	1
CLIP1	ZNRD1-AS1	0.616088076	MANF	SNHG3	0.9687182	FAM117B	ZNRD1-AS1	1
NUP205	SNHG3	0.615942041	MAP3K13	H19	0.91169356	FAM124A	RHPN1-AS1	1
CD302	KTN1-AS1	0.615823114	MAP3K3	H19	0.600670658	FAM126A	HCP5	1
ZNF2	HCP5	0.615790576	MAP3K3	KTN1-AS1	0.828547402	FAM126A	MCM3AP-AS1	1
UBR1	H19	0.615721994	MAP3K3	MCM3AP-AS1	0.849742338	FAM126A	SCARNA9	1
BRI3BP	H19	0.615709907	MAP3K3	ZNRD1-AS1	0.842197687	FAM126A	SNHG3	1
C12orf5	HCP5	0.615694318	MAP3K5	HCP5	0.848372313	FAM127A	MCM3AP-AS1	1
ATP1B1	MCM3AP-AS1	0.615660415	MAP3K5	KTN1-AS1	0.889529692	FAM127B	SNHG3	1
PPM1G	H19	0.615449034	MAP3K5	MCM3AP-AS1	0.862523826	FAM127C	SNHG3	1
RABGAP1L	HCP5	0.615310328	MAP3K5	TPT1-AS1	0.53704759	FAM129A	H19	1
SUV39H2	H19	0.614990269	MAP3K5	ZNRD1-AS1	0.79716031	FAM129A	SNHG5	1
HOXA7	SNHG3	0.614858355	MAP3K8	SNHG3	0.600294912	FAM129A	ZNRD1-AS1	1
FLI1	SNHG3	0.614698449	MAP3K8	SNHG5	0.540339295	FAM133A	DLEU2	1
EDA2R	ZNRD1-AS1	0.614684087	MAP4	MCM3AP-AS1	0.681616444	FAM133A	H19	1
DKC1	H19	0.614616494	MAP4	ZNRD1-AS1	0.768460384	FAM133A	TPT1-AS1	1
PEX6	TP53TG1	0.614481048	MAP4K1	MCM3AP-AS1	0.929402572	FAM133A	ZNRD1-AS1	1

IL1RAP	H19	0.614198561	MAP4K4	MCM3AP-AS1	0.507497612	FAM134A	HCP5	1
KAL1	MCM3AP-AS1	0.613565061	MAP4K4	TPT1-AS1	0.794379814	FAM134A	KTN1-AS1	1
CUX1	ZNRD1-AS1	0.613558398	MAP4K4	ZNRD1-AS1	0.573937705	FAM134A	MCM3AP-AS1	1
CSNK1G1	HCP5	0.613484649	MAP7	H19	0.671932938	FAM136A	HCP5	1
SYPL1	DLEU2	0.613257612	MAP7	HCP5	0.658405565	FAM136A	RHPN1-AS1	1
SLC4A7	MCM3AP-AS1	0.613143526	MAP7	KTN1-AS1	0.96979523	FAM160B1	DLEU2	1
PNRC1	KTN1-AS1	0.61296828	MAP7	MCM3AP-AS1	0.916304769	FAM160B1	H19	1
NDUFA5	TPT1-AS1	0.612189161	MAP7	ZNRD1-AS1	0.817862779	FAM160B1	SNHG5	1
GTF2I	MCM3AP-AS1	0.612058695	MAP7D2	KTN1-AS1	0.754657984	FAM160B1	TPT1-AS1	1
PPAP2B	SNHG3	0.611258342	MAP7D2	MCM3AP-AS1	0.801928823	FAM160B1	ZNRD1-AS1	1
PSD3	SNHG3	0.611157647	MAP7D2	ZNRD1-AS1	0.826969437	FAM171B	HCP5	1
PAG1	H19	0.61109162	MAPKAP1	SNHG3	0.840336714	FAM173B	RHPN1-AS1	1
IQGAP2	HCP5	0.611008965	MARCKS	DLEU2	0.626419129	FAM175A	H19	1
TACC1	H19	0.610960605	MARCKS	SNHG3	0.618884281	FAM177A1	H19	1
SLC39A14	TP53TG1	0.61088073	MARVELD1	HCP5	0.760270856	FAM177A1	LINC00467	1
DPYSL2	HCP5	0.610634979	MAST3	HCP5	0.711631426	FAM177A1	RUSC1-AS1	1
SGMS2	HCP5	0.610562157	MAST3	TPT1-AS1	0.710838335	FAM177A1	SNHG5	1
PSIP1	ZNRD1-AS1	0.610494943	MAST3	ZNRD1-AS1	0.911411315	FAM177A1	ZNRD1-AS1	1
MTFR2	H19	0.610492874	MAST4	DLEU2	0.525684997	FAM184A	HCP5	1
RACGAP1	H19	0.610487713	MAST4	SNHG3	0.646343292	FAM184A	TP53TG1	1
CERCAM	HCP5	0.610322245	MAST4	SNHG5	0.771685122	FAM188A	HCP5	1
PHKA1	ZNRD1-AS1	0.610279062	MASTL	SNHG3	0.677966717	FAM188A	SNHG3	1
KIF20B	TPT1-AS1	0.609905501	MBNL3	H19	0.530630177	FAM189B	HCP5	1
C5	H19	0.609877447	MBOAT1	MCM3AP-AS1	0.728605796	FAM189B	MCM3AP-AS1	1
ARID4B	TPT1-AS1	0.609766752	MBOAT1	ZNRD1-AS1	0.603266333	FAM195A	HCP5	1
HIVEP2	SCARNA9	0.609693058	MBOAT2	SNHG5	0.989090133	FAM195A	MCM3AP-AS1	1
MLNR	SNHG5	0.609692693	MCF2L	DLEU2	0.588228382	FAM198B	SNHG5	1
TOMM22	KTN1-AS1	0.609664869	MCF2L	HCP5	0.634818357	FAM200A	MCM3AP-AS1	1
PRMT5	SCARNA9	0.60940786	MCF2L	TPT1-AS1	0.624740226	FAM20C	SNHG5	1
UTRN	MCM3AP-AS1	0.609351102	MCF2L	ZNRD1-AS1	0.761753192	FAM217B	HCP5	1
SLC31A1	LINC00467	0.609198764	MCM2	LINC00467	0.648731085	FAM219A	SCARNA9	1
MYLIP	SNHG3	0.609177572	MCM3	H19	0.672981354	FAM43A	DLEU2	1
PPAPDC2	SNHG5	0.609173385	MCM3	HCP5	0.809182096	FAM43A	H19	1
STK38L	MCM3AP-AS1	0.608584928	MCM4	HCP5	0.51733845	FAM43A	SNHG5	1
FAM134B	MCM3AP-AS1	0.608438098	MCM4	KTN1-AS1	0.651377691	FAM43A	TPT1-AS1	1
FRY	MCM3AP-AS1	0.608377106	MCM4	MCM3AP-AS1	0.51151867	FAM43A	ZNRD1-AS1	1
ZBTB44	KTN1-AS1	0.608305034	MCM6	SNHG3	0.661196339	FAM45A	HCP5	1
KIF11	DLEU2	0.608148483	MCM7	H19	0.581610881	FAM45A	KTN1-AS1	1
HSPD1	KTN1-AS1	0.608026508	MDH1	SNHG5	0.582143515	FAM45A	MCM3AP-AS1	1
CTSS	HCP5	0.608008535	MDM2	H19	0.507964435	FAM45A	SNHG3	1
KIAA0922	KTN1-AS1	0.607971027	MDM2	HCP5	0.751873096	FAM46A	DLEU2	1
CAMLG	LINC00467	0.607919042	MDM2	ZNRD1-AS1	0.847366867	FAM46A	H19	1
ZMYM3	SNHG3	0.607891394	ME1	H19	0.932730984	FAM46A	SNHG5	1
SUB1	SNHG3	0.607840155	MECOM	HCP5	0.7464418	FAM46A	TPT1-AS1	1
RSBN1	SNHG5	0.607734101	MECOM	MCM3AP-AS1	0.922291314	FAM46A	ZNRD1-AS1	1
NDUFB6	SNHG3	0.607733399	MECOM	TPT1-AS1	0.611310284	FAM46C	HCP5	1
PER3	ZNRD1-AS1	0.607620431	MECOM	ZNRD1-AS1	0.843394871	FAM46C	KTN1-AS1	1
PRDM5	MCM3AP-AS1	0.607559546	MED10	DLEU2	0.816115323	FAM46C	MCM3AP-AS1	1
TRIB1	LINC00467	0.607531186	MED12L	DLEU2	0.710784515	FAM46C	SCARNA9	1
DNAJC12	KTN1-AS1	0.60745969	MED12L	HCP5	0.719012844	FAM46C	TP53TG1	1
MAP2K3	HCP5	0.607378283	MED15	MCM3AP-AS1	0.773706396	FAM49A	SNHG5	1

TAF5	ZNRD1-AS1	0.606780479	MED15	ZNRD1-AS1	0.833001946	FAM50B	HCP5	1
ZNF615	SCARNA9	0.606642766	MED16	H19	0.935044339	FAM50B	KTN1-AS1	1
RBM38	LINC00467	0.606209698	MED16	ZNRD1-AS1	0.614560179	FAM50B	MCM3AP-AS1	1
ALPK1	SNHG5	0.606164696	MEF2A	SNHG3	0.517014934	FAM53B	H19	1
NKIRAS1	HCP5	0.605990754	MEIS1	KTN1-AS1	0.889847837	FAM53B	ZNRD1-AS1	1
HS2ST1	RUSC1-AS1	0.605956739	MEIS1	MCM3AP-AS1	0.829655983	FAM58A	SNHG3	1
ZHX3	MCM3AP-AS1	0.605890877	MELK	H19	0.770959416	FAM63B	DLEU2	1
TBC1D12	SNHG5	0.605883276	MEST	HCP5	0.924565939	FAM63B	H19	1
FAM134A	SNHG5	0.605831043	METAP1	HCP5	0.607162512	FAM63B	SNHG5	1
KIAA1161	SCARNA9	0.605779056	MEX3D	H19	0.887600711	FAM63B	ZNRD1-AS1	1
ST8SIA4	SNHG5	0.605741187	MEX3D	HCP5	0.518453636	FAM64A	RUSC1-AS1	1
DTL	H19	0.60559269	MFN2	SNHG5	0.704572523	FAM65B	HCP5	1
ZNF397	MCM3AP-AS1	0.605454865	MGLL	SNHG5	0.624512574	FAM78A	HCP5	1
TGFBR2	KTN1-AS1	0.60536882	MGST1	HCP5	0.926518096	FAM78A	TP53TG1	1
DENND4A	DLEU2	0.605100453	MIF4GD	H19	0.824947637	FAM81A	DLEU2	1
ZDHHC11	MCM3AP-AS1	0.604892294	MIF4GD	HCP5	0.683799147	FAM81A	TPT1-AS1	1
DIRC2	SNHG3	0.604772397	MIS18BP1	H19	0.608723456	FAM81A	ZNRD1-AS1	1
TBCEL	HCP5	0.604579829	MIS18BP1	TPT1-AS1	0.590158915	FAM83D	HCP5	1
CUTC	SNHG5	0.603541768	MKNK1	MCM3AP-AS1	0.606780741	FAM84B	H19	1
DIS3	MCM3AP-AS1	0.603113119	MLEC	H19	0.667688649	FAM84B	ZNRD1-AS1	1
TRUB1	HCP5	0.602866017	MLEC	MCM3AP-AS1	0.542476661	FAM92A1	MCM3AP-AS1	1
HCFC2	MCM3AP-AS1	0.602677041	MLLT3	HCP5	0.962228251	FANCB	HCP5	1
DLEU1	SNHG3	0.602640673	MLLT3	LINC00467	0.842695126	FANCB	KTN1-AS1	1
NBEA	MCM3AP-AS1	0.602090328	MLLT3	MCM3AP-AS1	0.678639808	FANCB	SNHG3	1
ARL13B	RUSC1-AS1	0.60191627	MLLT3	TP53TG1	0.667476559	FANCC	HCP5	1
KCNK2	HCP5	0.601908655	MLLT3	ZNRD1-AS1	0.581879979	FANCD2	HCP5	1
RBPMS	SNHG3	0.601693824	MMAA	HCP5	0.713780474	FANCD2	SNHG3	1
TRAPPC8	HCP5	0.601633616	MMD	DLEU2	0.639193693	FANCE	SNHG3	1
THRA	HCP5	0.601421937	MMD	HCP5	0.540829356	FANCI	KTN1-AS1	1
FER	H19	0.601332355	MMD	SNHG5	0.728517976	FANCI	MCM3AP-AS1	1
ERLIN2	KTN1-AS1	0.601231113	MN1	MCM3AP-AS1	0.793899379	FANCI	SNHG3	1
DHODH	TPT1-AS1	0.601222222	MN1	ZNRD1-AS1	0.814971364	FANCM	HCP5	1
TGFBR2	HCP5	0.601183552	MNAT1	SNHG3	0.766330232	FANCM	TP53TG1	1
RAB13	KTN1-AS1	0.601103626	MORN2	H19	0.987771128	FASTKD3	KTN1-AS1	1
CPEB4	HCP5	0.601050481	MORN2	LINC00467	0.821933084	FASTKD3	MCM3AP-AS1	1
BMPR1B	SCARNA9	0.601000707	MOSPD3	HCP5	0.823431228	FASTKD5	HCP5	1
HNRNPAB	H19	0.600945149	MPDZ	HCP5	0.907950576	FASTKD5	MCM3AP-AS1	1
STK38L	SNHG3	0.600839376	MPP7	ZNRD1-AS1	0.604866799	FASTKD5	SNHG3	1
SYPL1	SNHG3	0.600810106	MPPED2	H19	0.644620245	FAT4	HCP5	1
FOXO3	SNHG5	0.600669178	MPPED2	KTN1-AS1	0.913946278	FAT4	KTN1-AS1	1
SCPEP1	SNHG3	0.600564692	MPPED2	MCM3AP-AS1	0.842842872	FAT4	MCM3AP-AS1	1
RCOR1	H19	0.600460477	MPPED2	ZNRD1-AS1	0.726410117	FBXL5	HCP5	1
VKORC1L1	SNHG3	0.600216884	MREG	H19	0.528544095	FBXL6	MCM3AP-AS1	1
TRUB1	KTN1-AS1	0.599690083	MREG	ZNRD1-AS1	0.741623072	FBXO22	SNHG3	1
KLHDC8B	MCM3AP-AS1	0.599230461	MRPL2	H19	0.994774616	FBXO4	KTN1-AS1	1
UBE2E2	MCM3AP-AS1	0.599043209	MRPL3	RHPN1-AS1	0.976878142	FBXO4	MCM3AP-AS1	1
FAM195A	ZNRD1-AS1	0.598999451	MRPL30	TP53TG1	0.900650969	FBXO5	SCARNA9	1
C2CD2	ZNRD1-AS1	0.59884001	MRPL37	KTN1-AS1	0.628586527	FBXW7	DLEU2	1
L2HGDH	ZNRD1-AS1	0.598675536	MRPL37	TP53TG1	0.973460965	FBXW7	H19	1
SCARB2	RHPN1-AS1	0.598648745	MRPL40	H19	0.666691582	FBXW7	SNHG5	1
PRMT2	SNHG5	0.59864008	MRPS35	H19	0.513721135	FBXW7	ZNRD1-AS1	1

AKT3	DLEU2	0.59827591	MRRF	HCP5	0.936439691	FCHO1	HCP5	1
DUSP8	HCP5	0.597217892	MRRF	LINC00467	0.734109975	FCHO2	HCP5	1
ASPHD1	ZNRD1-AS1	0.597064207	MRRF	MCM3AP-AS1	0.715841132	FCHO2	KTN1-AS1	1
SHISA5	SNHG5	0.596938992	MRRF	ZNRD1-AS1	0.662874328	FCHO2	MCM3AP-AS1	1
PEBP1	KTN1-AS1	0.596785294	MRS2	HCP5	0.917421676	FCHO2	SNHG3	1
FASTKD5	MCM3AP-AS1	0.596307793	MRS2	ZNRD1-AS1	0.654205005	FCHO2	TP53TG1	1
IMMP2L	LINC00467	0.595948589	MSH2	SNHG3	0.981000894	FER	H19	1
LANCL1	KTN1-AS1	0.595908855	MSH2	SNHG5	0.790994753	FER	SNHG5	1
HSPH1	H19	0.595907836	MSRB2	MCM3AP-AS1	0.515809703	FER	ZNRD1-AS1	1
STK17A	ZNRD1-AS1	0.595883816	MTHFD2	SNHG3	0.997808565	FERMT1	MCM3AP-AS1	1
STK24	KTN1-AS1	0.595772123	MTHFD2	SNHG5	0.648476284	FERMT2	HCP5	1
CHD2	MCM3AP-AS1	0.595739813	MTL5	KTN1-AS1	0.833031965	FERMT2	KTN1-AS1	1
SLAIN1	TPT1-AS1	0.595522398	MTL5	MCM3AP-AS1	0.812374949	FERMT2	MCM3AP-AS1	1
TMEFF1	MCM3AP-AS1	0.595506931	MTMR10	ZNRD1-AS1	0.706370751	FERMT2	SNHG3	1
LRP12	SNHG3	0.595301843	MTMR14	SNHG3	0.784637362	FERMT2	TP53TG1	1
TAB3	RUSC1-AS1	0.595281765	MTMR9	SNHG3	0.966405803	FGD4	HCP5	1
EZH1	HCP5	0.595151101	MTMR9	SNHG5	0.644411542	FGD4	SNHG3	1
FLI1	MCM3AP-AS1	0.594645804	MTSS1L	ZNRD1-AS1	0.958609625	FGF2	MCM3AP-AS1	1
GPR137C	ZNRD1-AS1	0.594410211	MTX2	SNHG5	0.503428799	FGF2	SCARNA9	1
ARID5B	TP53TG1	0.594269877	MXI1	H19	0.825060112	FGF9	MCM3AP-AS1	1
NDFIP1	TP53TG1	0.594175593	MXI1	ZNRD1-AS1	0.552126466	FGF9	SCARNA9	1
BSDC1	ZNRD1-AS1	0.593335185	MYBL1	H19	0.943031269	FGFR2	DLEU2	1
FHIT	MCM3AP-AS1	0.593324139	MYBL2	H19	0.854255836	FGFR2	LINC00467	1
NAA50	HCP5	0.593178831	MYBL2	HCP5	0.646682794	FGL2	HCP5	1
MXI1	SNHG5	0.592959955	MYCBP2	DLEU2	0.516962756	FHL1	HCP5	1
ZW10	SNHG3	0.592727417	MYCN	HCP5	0.990893468	FHL1	MCM3AP-AS1	1
KCTD9	DLEU2	0.592718893	MYCN	LINC00467	0.783656874	FHL1	SNHG3	1
TMEM45A	SNHG5	0.59243805	MYD88	SNHG3	0.822727438	FICD	HCP5	1
TRIP10	RUSC1-AS1	0.592424036	MYH9	TPT1-AS1	0.789727878	FICD	RHPN1-AS1	1
ASCC3	MCM3AP-AS1	0.592229237	MYH9	ZNRD1-AS1	0.527768268	FIGN	HCP5	1
TMEM38B	SNHG3	0.592161615	MYLIP	H19	0.567232904	FIGN	MCM3AP-AS1	1
PRR15	LINC00467	0.592011672	MYLIP	TPT1-AS1	0.849860034	FIGN	SNHG3	1
TLE4	ZNRD1-AS1	0.591951708	MYLIP	ZNRD1-AS1	0.95377569	FIGN	TP53TG1	1
CPNE8	SNHG3	0.59165269	MYO18A	TPT1-AS1	0.908420768	FIGNL1	HCP5	1
RSRP1	ZNRD1-AS1	0.591603627	MYO19	SNHG3	0.636241368	FKBP10	HCP5	1
EXO5	H19	0.591123188	MYO1D	SNHG5	0.574685925	FKBP11	H19	1
KIAA0895	HCP5	0.591007563	MYO1E	SNHG3	0.776900459	FKBP11	ZNRD1-AS1	1
NAA15	MCM3AP-AS1	0.59094801	MYO1G	MCM3AP-AS1	0.846851951	FKBP1B	ZNRD1-AS1	1
ZC3H6	MCM3AP-AS1	0.590850371	MYO1G	ZNRD1-AS1	0.750883122	FKBP9	KTN1-AS1	1
SEH1L	HCP5	0.590639706	MYO5A	DLEU2	0.692294773	FKBP9	SCARNA9	1
IGF2BP3	HCP5	0.590611278	MYO5A	MCM3AP-AS1	0.569009813	FKTN	H19	1
BTN3A1	MCM3AP-AS1	0.590385118	MYO5A	TPT1-AS1	0.815826592	FKTN	SNHG5	1
TBC1D4	RUSC1-AS1	0.590352877	MYO5A	ZNRD1-AS1	0.722594519	FKTN	ZNRD1-AS1	1
CRBN	MCM3AP-AS1	0.590292949	MYO5C	H19	0.50782993	FLI1	MCM3AP-AS1	1
XRRA1	ZNRD1-AS1	0.590264688	MYO5C	KTN1-AS1	0.985664386	FLI1	SNHG3	1
DCAF17	MCM3AP-AS1	0.590016086	MYO5C	LINC00467	0.75032844	FLI1	TP53TG1	1
NFKBIA	DLEU2	0.589837086	MYO5C	MCM3AP-AS1	0.997232825	FLNB	H19	1
KLF5	KTN1-AS1	0.58952386	MYO5C	ZNRD1-AS1	0.972401574	FLNB	LINC00467	1
BAG4	SNHG5	0.589523372	N6AMT2	H19	0.573494615	FLNB	ZNRD1-AS1	1
MYCBP2	H19	0.589517332	NAA15	H19	0.884992159	FLOT2	MCM3AP-AS1	1
NUP35	HCP5	0.589471743	NAA15	TP53TG1	0.625186358	FLT1	H19	1

INPP1	DLEU2	0.589376593	NAA16	MCM3AP-AS1	0.834785713	FLT1	ZNRD1-AS1	1
RAD18	H19	0.589177936	NAA16	ZNRD1-AS1	0.855300669	FLVCR2	DLEU2	1
AMPD2	HCP5	0.588880245	NAA50	H19	0.520615898	FLVCR2	TPT1-AS1	1
LRMP	HCP5	0.58879507	NAA50	HCP5	0.916441837	FMNL2	H19	1
AAK1	H19	0.588210189	NAA50	KTN1-AS1	0.597702766	FMNL2	ZNRD1-AS1	1
CHN1	MCM3AP-AS1	0.588209269	NAA50	LINC00467	0.885399747	FMNL3	H19	1
CARD16	MCM3AP-AS1	0.588173647	NAGPA	HCP5	0.733070684	FMNL3	ZNRD1-AS1	1
RSRP1	H19	0.588093203	NAP1L2	HCP5	0.588022735	FN1	HCP5	1
CLIP4	HCP5	0.588020297	NAP1L5	SNHG5	0.783979453	FN1	RHPN1-AS1	1
HGSNAT	SNHG5	0.587921682	NAPB	ZNRD1-AS1	0.846975255	FN3KRP	TP53TG1	1
CCDC15	MCM3AP-AS1	0.587821189	NAPEPLD	H19	0.597423725	FNBP1	H19	1
ARL2	HCP5	0.587797489	NAPEPLD	HCP5	0.833116533	FNBP1	LINC00467	1
NDUFA5	MCM3AP-AS1	0.587757967	NAPEPLD	MCM3AP-AS1	0.799716987	FNBP1	ZNRD1-AS1	1
USP53	KTN1-AS1	0.587564491	NAPEPLD	ZNRD1-AS1	0.679998954	FNDC3B	DLEU2	1
FAM127C	SNHG3	0.587552372	NASP	H19	0.955235614	FNDC3B	H19	1
DNPH1	SNHG5	0.587447754	NASP	ZNRD1-AS1	0.537818448	FNDC3B	LINC00467	1
SLC41A2	DLEU2	0.587436723	NAV1	MCM3AP-AS1	0.752037496	FNDC3B	SNHG5	1
KIF11	H19	0.586867643	NAV1	TPT1-AS1	0.914992895	FNDC3B	TPT1-AS1	1
PTPRM	RUSC1-AS1	0.586827994	NAV1	ZNRD1-AS1	0.789977813	FNDC3B	ZNRD1-AS1	1
SAP30BP	DLEU2	0.586801737	NBEA	H19	0.727850804	FNTB	RHPN1-AS1	1
CPVL	SNHG3	0.586688096	NBEA	HCP5	0.643409911	FOS	HCP5	1
ELOVL6	RUSC1-AS1	0.586556577	NCAPD2	H19	0.890529939	FOSB	HCP5	1
UHRF1	H19	0.586523143	NCAPD2	HCP5	0.587098329	FOSB	KTN1-AS1	1
KLF4	SNHG3	0.586064521	NCAPG	KTN1-AS1	0.655122386	FOSB	MCM3AP-AS1	1
BUB1	H19	0.585938936	NCAPG	MCM3AP-AS1	0.517522671	FOSL1	MCM3AP-AS1	1
RNF19A	TPT1-AS1	0.585766497	NCF2	MCM3AP-AS1	0.630760594	FOSL2	SNHG5	1
DAB2	KTN1-AS1	0.585396311	NCKAP1	SNHG3	0.995868091	FOXM1	KTN1-AS1	1
DGKH	H19	0.585309725	NCKAP1	SNHG5	0.720338138	FOXM1	MCM3AP-AS1	1
SCAMP5	HCP5	0.585079752	NCOR2	MCM3AP-AS1	0.89241769	FOXN2	HCP5	1
PLK2	HCP5	0.584996208	NCOR2	TPT1-AS1	0.982474677	FOXN2	SNHG3	1
PLK3	ZNRD1-AS1	0.584885704	NCOR2	ZNRD1-AS1	0.930921106	FOXO3	RUSC1-AS1	1
BTN3A1	HCP5	0.584806956	NDFIP1	H19	0.532166671	FOXO3	SNHG5	1
GMCL1	H19	0.584574899	NDFIP1	LINC00467	0.535112418	FOXRED1	MCM3AP-AS1	1
ZC3HAV1L	SNHG5	0.584556164	NDFIP1	TP53TG1	0.788157138	FOXRED1	SNHG3	1
MFSD2A	H19	0.584542998	NDUFA5	TP53TG1	0.885860074	FOXRED2	HCP5	1
DSTYK	H19	0.584372539	NDUFB6	SNHG3	0.940563551	FRAT1	KTN1-AS1	1
IGF2BP3	MCM3AP-AS1	0.584106466	NECAB3	ZNRD1-AS1	0.710467658	FRAT1	MCM3AP-AS1	1
MYLIP	HCP5	0.583888697	NEFH	H19	0.780131418	FRMD4A	MCM3AP-AS1	1
HNRNPR	H19	0.58368113	NEFH	ZNRD1-AS1	0.701755888	FRMD6	HCP5	1
MAP4	H19	0.583584037	NEK6	SNHG5	0.686301802	FRMD6	MCM3AP-AS1	1
FAT4	SNHG5	0.583138855	NEK9	HCP5	0.841409956	FRMD6	SCARNA9	1
INA	SCARNA9	0.582949421	NEK9	LINC00467	0.830081294	FRMD6	SNHG3	1
APC	ZNRD1-AS1	0.582482883	NEK9	ZNRD1-AS1	0.809533576	FRY	H19	1
TMEM65	RHPN1-AS1	0.582020081	NET1	SNHG3	0.759304187	FRY	ZNRD1-AS1	1
LIMA1	ZNRD1-AS1	0.58201166	NET1	SNHG5	0.931637782	FSD1	MCM3AP-AS1	1
PAM	MCM3AP-AS1	0.581861382	NETO2	SNHG3	0.721972526	FSD1L	H19	1
LRRFIP1	MCM3AP-AS1	0.581843087	NFAT5	DLEU2	0.559683516	FSD1L	SNHG5	1
ZDHHC1	LINC00467	0.581001887	NFE2L1	H19	0.641642614	FSD1L	ZNRD1-AS1	1
UQCRB	SNHG5	0.58086198	NFE2L1	MCM3AP-AS1	0.783067775	FSIP1	HCP5	1
SIX4	SNHG3	0.580681571	NFE2L1	ZNRD1-AS1	0.769876574	FUBP3	HCP5	1
GSTK1	RHPN1-AS1	0.580561008	NFIA	DLEU2	0.585582684	FUBP3	KTN1-AS1	1

NKIRAS1	ZNRD1-AS1	0.580087748	NFIA	HCP5	0.780235131	FUBP3	MCM3AP-AS1	1
AMPD2	ZNRD1-AS1	0.579988608	NFIA	SCARNA9	0.950121548	FUBP3	SNHG3	1
PHYH	SNHG3	0.579823006	NFKBIA	SNHG3	0.80775896	FUCA1	KTN1-AS1	1
ARHGAP10	HCP5	0.579703049	NFYB	MCM3AP-AS1	0.64113313	FUCA2	HCP5	1
PLAA	H19	0.579606594	NHEJ1	MCM3AP-AS1	0.508743249	FUCA2	KTN1-AS1	1
CCNE2	H19	0.579523863	NID1	SNHG3	0.767461478	FXN	H19	1
ZMAT3	ZNRD1-AS1	0.579359585	NID1	SNHG5	0.675487222	FXYD5	HCP5	1
TLE4	H19	0.579129305	NIP7	SNHG3	0.914941624	FYN	H19	1
NBR1	MCM3AP-AS1	0.579122594	NIPAL2	LINC00467	0.638820549	FYN	ZNRD1-AS1	1
IL12A	ZNRD1-AS1	0.579083754	NIPAL2	MCM3AP-AS1	0.858420584	FZD3	HCP5	1
TOM1L2	ZNRD1-AS1	0.578980002	NIPAL2	ZNRD1-AS1	0.859302868	FZD3	MCM3AP-AS1	1
ZBTB10	ZNRD1-AS1	0.578954699	NIPAL3	H19	0.616961808	FZD3	SNHG3	1
KIAA0430	SNHG3	0.578933338	NIPAL3	LINC00467	0.97229941	FZD5	DLEU2	1
ZBTB10	MCM3AP-AS1	0.578653396	NIPAL4	HCP5	0.956857039	FZD5	H19	1
KIAA1211	ZNRD1-AS1	0.578591412	NIPAL4	TP53TG1	0.540647481	FZD5	RUSC1-AS1	1
SCAPER	HCP5	0.578563778	NIPAL4	ZNRD1-AS1	0.62242663	GAB1	DLEU2	1
YTHDF2	HCP5	0.578398058	NIPSNAP1	HCP5	0.87378691	GAB1	H19	1
RAPGEF1	ZNRD1-AS1	0.578394396	NIPSNAP1	MCM3AP-AS1	0.629579616	GAB1	LINC00467	1
CRBN	KTN1-AS1	0.578223065	NKIRAS1	H19	0.910726497	GAB1	RUSC1-AS1	1
DSTYK	ZNRD1-AS1	0.578146627	NKIRAS1	LINC00467	0.912719918	GAB1	TPT1-AS1	1
PTPN13	RUSC1-AS1	0.577707736	NKIRAS1	ZNRD1-AS1	0.655531264	GAB1	ZNRD1-AS1	1
TM4SF1	DLEU2	0.577529258	NLE1	LINC00467	0.970150058	GAB2	SNHG5	1
C16orf72	HCP5	0.577448034	NLGN1	DLEU2	0.755476693	GABARAP	HCP5	1
SLC9A8	TPT1-AS1	0.57743932	NLGN1	MCM3AP-AS1	0.583691332	GABARAP	MCM3AP-AS1	1
SHMT1	SNHG5	0.577418305	NLGN1	TPT1-AS1	0.688531171	GABARAPL2	DLEU2	1
DHX33	MCM3AP-AS1	0.577388289	NLK	SCARNA9	0.721752947	GABARAPL2	LINC00467	1
MIF4GD	RHPN1-AS1	0.577335833	NLK	SNHG3	0.722148778	GADD45B	MCM3AP-AS1	1
FAM49A	SNHG5	0.577322919	NLK	SNHG5	0.956628184	GAK	H19	1
ARPC5L	H19	0.577225917	NLN	HCP5	0.723927816	GAK	ZNRD1-AS1	1
BMP4	LINC00467	0.577124709	NME4	H19	0.631059506	GALK1	KTN1-AS1	1
PLEKHA6	SNHG5	0.576780513	NME4	HCP5	0.807539064	GALK1	MCM3AP-AS1	1
ELK3	MCM3AP-AS1	0.576703686	NME4	ZNRD1-AS1	0.686685505	GALNT1	HCP5	1
SEPN1	HCP5	0.57654429	NMT2	KTN1-AS1	0.826154026	GALNT1	MCM3AP-AS1	1
CDH2	HCP5	0.576485414	NMT2	MCM3AP-AS1	0.741958908	GALNT1	SCARNA9	1
MAPKAPK3	HCP5	0.57625205	NMT2	ZNRD1-AS1	0.614338972	GALNT1	SNHG3	1
SMDT1	MCM3AP-AS1	0.576153631	NOG	HCP5	0.65147588	GALNT10	HCP5	1
MPDZ	HCP5	0.576125426	NOLC1	MCM3AP-AS1	0.902967435	GALNT10	SCARNA9	1
ASCC3	SNHG3	0.575914451	NOP2	MCM3AP-AS1	0.79578431	GALNT10	TP53TG1	1
SEH1L	LINC00467	0.575797062	NOP2	ZNRD1-AS1	0.679464403	GALNT12	SCARNA9	1
MCAM	DLEU2	0.575737722	NOTCH2	DLEU2	0.879083591	GAPT	HCP5	1
GLCCI1	SCARNA9	0.575615827	NOTCH2	RUSC1-AS1	0.619276358	GARS	HCP5	1
PTPN1	HCP5	0.575103066	NOTCH2	SNHG5	0.731681568	GART	SNHG3	1
RIOK3	TPT1-AS1	0.574913136	NPEPL1	H19	0.687175459	GAS2L3	HCP5	1
CCDC82	TPT1-AS1	0.57483428	NPEPL1	ZNRD1-AS1	0.897879468	GAS7	H19	1
PRDM2	H19	0.574781435	NPLOC4	DLEU2	0.657420636	GATA2	HCP5	1
TAGAP	HCP5	0.574672165	NPLOC4	SNHG5	0.99216622	GATA2	TP53TG1	1
ACVR1C	KTN1-AS1	0.574323961	NPR3	H19	0.583089775	GATA3	HCP5	1
ITM2B	SNHG5	0.574268934	NPR3	HCP5	0.773372853	GATA3	MCM3AP-AS1	1
ZHX1	ZNRD1-AS1	0.574157458	NPR3	KTN1-AS1	0.93699308	GATA3	SNHG3	1
PINK1	TP53TG1	0.574031819	NPR3	MCM3AP-AS1	0.882113754	GATA6	DLEU2	1
ITPR1	H19	0.57398551	NPR3	TPT1-AS1	0.531211971	GATA6	H19	1

RIC1	MCM3AP-AS1	0.573945693	NPR3	ZNRD1-AS1	0.78326438	GATA6	RUSC1-AS1	1
ELL2	DLEU2	0.573865712	NR2C2AP	H19	0.712950352	GATA6	SNHG5	1
PLEKHA8	DLEU2	0.573823777	NR2C2AP	HCP5	0.728084939	GATA6	TPT1-AS1	1
SEH1L	KTN1-AS1	0.573580206	NR2C2AP	TP53TG1	0.96061059	GATA6	ZNRD1-AS1	1
MYLIP	ZNRD1-AS1	0.573460531	NR4A1	DLEU2	0.576085059	GATM	KTN1-AS1	1
ATP2B1	HCP5	0.573451243	NR4A1	SNHG3	0.629416482	GATM	SNHG3	1
HOXB3	ZNRD1-AS1	0.573423673	NR4A2	SNHG3	0.916025829	GBE1	HCP5	1
EMB	SNHG5	0.573369275	NRCAM	SNHG5	0.924549443	GBE1	MCM3AP-AS1	1
NFYB	TPT1-AS1	0.573348447	NREP	H19	0.664730654	GBE1	SNHG3	1
ACTA2	HCP5	0.573258672	NREP	HCP5	0.837779032	GBF1	H19	1
EPN2	HCP5	0.57315649	NREP	LINC00467	0.974816495	GBF1	ZNRD1-AS1	1
CCDC82	MCM3AP-AS1	0.573105932	NRIP1	TPT1-AS1	0.624314486	GBP1	DLEU2	1
BRD2	ZNRD1-AS1	0.57310067	NRIP1	ZNRD1-AS1	0.74662133	GBP1	SNHG5	1
CHM	SNHG3	0.573070205	NRP1	SNHG3	0.681000771	GBP1	TPT1-AS1	1
TMEM187	SNHG3	0.573006547	NRP2	SNHG3	0.953764896	GBP1	ZNRD1-AS1	1
TIMP3	H19	0.572981242	NSMCE2	SNHG5	0.720295477	GCA	HCP5	1
PLEKHA6	H19	0.572727106	NSUN7	HCP5	0.712347616	GCH1	H19	1
CIDEB	ZNRD1-AS1	0.572683907	NSUN7	ZNRD1-AS1	0.896828904	GCH1	ZNRD1-AS1	1
PPOX	TPT1-AS1	0.572677465	NT5DC2	LINC00467	0.642867656	GCNT1	DLEU2	1
COL15A1	H19	0.572535496	NUAK2	SNHG5	0.761761443	GCNT1	ZNRD1-AS1	1
CASP7	MCM3AP-AS1	0.572453092	NUCB2	HCP5	0.744722626	GCNT2	DLEU2	1
PCTP	KTN1-AS1	0.572453009	NUCB2	MCM3AP-AS1	0.856515086	GCNT2	RUSC1-AS1	1
PLEKHA1	HCP5	0.572345896	NUDCD2	HCP5	0.684294539	GCNT2	TPT1-AS1	1
AATF	MCM3AP-AS1	0.572329978	NUDCD2	KTN1-AS1	0.970654251	GCNT2	ZNRD1-AS1	1
YPEL5	MCM3AP-AS1	0.572080937	NUDCD2	MCM3AP-AS1	0.963431106	GCOM1	KTN1-AS1	1
PSTPIP2	H19	0.572038537	NUDCD2	ZNRD1-AS1	0.91759578	GCOM1	SCARNA9	1
PDK4	HCP5	0.57196485	NUDCD3	H19	0.585253359	GCOM1	SNHG3	1
SH2D4A	SNHG3	0.571743518	NUDT6	H19	0.970066115	GDE1	SNHG3	1
ZNF367	H19	0.571668553	NUDT8	H19	0.53864253	GDF15	RHPN1-AS1	1
HMMR	DLEU2	0.571509943	NUDT8	ZNRD1-AS1	0.551953492	GDNF	KTN1-AS1	1
LYRM1	SCARNA9	0.571291931	NUMB	H19	0.765793022	GDNF	MCM3AP-AS1	1
GNAI1	MCM3AP-AS1	0.571242263	NUP205	SNHG3	0.517407806	GFI1	RHPN1-AS1	1
DEK	MCM3AP-AS1	0.571206037	NUP210	H19	0.665881822	GFPT1	MCM3AP-AS1	1
PTPN22	SNHG5	0.570826812	NUP210	HCP5	0.675361875	GFPT2	DLEU2	1
TUBGCP4	MCM3AP-AS1	0.57078718	NUP210	MCM3AP-AS1	0.910280441	GFPT2	SNHG5	1
AGTRAP	SNHG3	0.570645487	NUP210	ZNRD1-AS1	0.810218094	GFPT2	TPT1-AS1	1
C12orf4	ZNRD1-AS1	0.570513374	NUP35	H19	0.625419427	GGCT	HCP5	1
LRCH2	RUSC1-AS1	0.570507169	NUP62CL	HCP5	0.807303836	GGCT	KTN1-AS1	1
HELLS	H19	0.570290715	NUP62CL	SCARNA9	0.789465993	GGCT	MCM3AP-AS1	1
POLR3G	H19	0.57019111	NUP88	HCP5	0.829275582	GGH	KTN1-AS1	1
ZNF395	RUSC1-AS1	0.570086768	NUPL1	SNHG5	0.747966447	GGH	MCM3AP-AS1	1
HS6ST2	LINC00467	0.56956938	NUS1	DLEU2	0.811878208	GGH	SNHG3	1
ARRDC4	H19	0.569513002	NUS1	SNHG5	0.943399948	GINS1	HCP5	1
SOBP	TPT1-AS1	0.5694608	NVL	H19	0.891677553	GINS1	KTN1-AS1	1
ACADM	RUSC1-AS1	0.569442919	NYNRIN	MCM3AP-AS1	0.765791891	GINS1	MCM3AP-AS1	1
HNRNPA3	TPT1-AS1	0.569390673	NYNRIN	ZNRD1-AS1	0.665165313	GINS1	SCARNA9	1
SESN1	MCM3AP-AS1	0.569116584	OAS1	SNHG3	0.922780393	GINS1	SNHG3	1
ATPAF1	HCP5	0.569097206	OAS2	HCP5	0.588535849	GINS4	HCP5	1
MPPED2	H19	0.569048927	OAS2	MCM3AP-AS1	0.979088535	GIPC2	RUSC1-AS1	1
ADORA2B	TP53TG1	0.568907709	OAS2	TPT1-AS1	0.82199721	GLA	H19	1
CCNYL1	ZNRD1-AS1	0.568670073	OAS2	ZNRD1-AS1	0.96860992	GLA	ZNRD1-AS1	1

DPP9	H19	0.568618953	OAS3	SNHG3	0.776485272	GLB1L2	MCM3AP-AS1	1
NOG	SNHG5	0.568535467	OGG1	H19	0.898900039	GLCCI1	HCP5	1
SGK1	KTN1-AS1	0.568476149	OGG1	LINC00467	0.782783935	GLCCI1	KTN1-AS1	1
SMAD7	HCP5	0.568114102	OIP5	H19	0.904117012	GLCCI1	MCM3AP-AS1	1
RGS16	HCP5	0.567992016	OMA1	ZNRD1-AS1	0.56295418	GLCCI1	SCARNA9	1
C16orf70	SNHG3	0.567789943	ORAI2	HCP5	0.705567801	GLCCI1	SNHG3	1
SGK3	SNHG5	0.56775393	ORAI3	H19	0.676978308	GLCCI1	TP53TG1	1
C1orf112	H19	0.567648375	ORAI3	LINC00467	0.934598012	GLCE	KTN1-AS1	1
TRIB1	HCP5	0.56762833	ORAI3	ZNRD1-AS1	0.84326699	GLCE	MCM3AP-AS1	1
CAMSAP2	ZNRD1-AS1	0.567615997	OSBPL1A	H19	0.648971396	GLCE	SNHG3	1
E2F8	H19	0.567168537	OSBPL1A	ZNRD1-AS1	0.783848323	GLG1	DLEU2	1
TCF4	RHPN1-AS1	0.567129826	OTUD1	SNHG3	0.814860836	GLG1	ZNRD1-AS1	1
AP3D1	DLEU2	0.566918727	OTUD1	SNHG5	0.976949234	GLI2	HCP5	1
ALAS1	MCM3AP-AS1	0.566715862	OTUD6B	SNHG3	0.933840381	GLI3	HCP5	1
CCNF	DLEU2	0.566565787	OTUD6B	SNHG5	0.731544389	GLI3	KTN1-AS1	1
ZNF326	ZNRD1-AS1	0.566554262	OXA1L	H19	0.864092652	GLI3	MCM3AP-AS1	1
CDK6	SNHG3	0.566307475	OXA1L	HCP5	0.586425785	GLI3	RHPN1-AS1	1
KDM6B	TPT1-AS1	0.566183729	P4HA1	SNHG3	0.907506958	GLI3	SNHG3	1
MLLT3	TP53TG1	0.565885462	P4HA1	SNHG5	0.878082112	GLIPR1	DLEU2	1
LANCL1	SNHG3	0.565499474	PAFAH1B3	KTN1-AS1	0.886648238	GLIPR1	RUSC1-AS1	1
ATXN1	MCM3AP-AS1	0.565456956	PAICS	H19	0.930064083	GLIPR1	SNHG5	1
SLC6A9	HCP5	0.565425902	PAIP2B	HCP5	0.840156879	GLIPR1	ZNRD1-AS1	1
HSPA9	DLEU2	0.564854453	PALLD	SNHG3	0.722390886	GLIS3	H19	1
TSHZ1	MCM3AP-AS1	0.564756953	PALLD	SNHG5	0.835689747	GLIS3	ZNRD1-AS1	1
PPARD	RUSC1-AS1	0.564720111	ALM2-AKAI	TP53TG1	0.958418699	GLMN	RHPN1-AS1	1
MED12L	H19	0.56429165	PAM	SNHG3	0.923185586	GLRB	MCM3AP-AS1	1
ZEB1	ZNRD1-AS1	0.563935975	PAM	SNHG5	0.841329144	GLRX	MCM3AP-AS1	1
MRS2	RUSC1-AS1	0.563790389	PANK1	HCP5	0.688653404	GLRX3	H19	1
CCDC8	H19	0.563641382	PANK1	MCM3AP-AS1	0.948408792	GLRX3	RUSC1-AS1	1
BCL6	DLEU2	0.563511236	PANK1	TPT1-AS1	0.665506281	GLRX3	SNHG5	1
IL21R	DLEU2	0.563228828	PANK1	ZNRD1-AS1	0.875416276	GLTP	HCP5	1
PPARA	HCP5	0.563164284	PAPD5	H19	0.79957683	GLTP	SNHG3	1
TMEM109	LINC00467	0.56316115	PAPD5	RHPN1-AS1	0.52179366	GLTSCR1	H19	1
ALPK1	KTN1-AS1	0.563107082	PAPSS2	SNHG5	0.769467482	GLTSCR1	ZNRD1-AS1	1
PKP4	SNHG3	0.562717981	PAQR4	HCP5	0.973965205	GLUL	SNHG3	1
STK17A	MCM3AP-AS1	0.562682836	PARK2	MCM3AP-AS1	0.992642783	GM2A	TP53TG1	1
FGFR2	TP53TG1	0.562531238	PARK2	ZNRD1-AS1	0.961521552	GMCL1	SNHG3	1
BTAF1	ZNRD1-AS1	0.562417311	PARP1	H19	0.519932213	GMCL1	TP53TG1	1
MACC1	SCARNA9	0.562365033	PARP1	HCP5	0.924282299	GMDS	HCP5	1
NUP205	SCARNA9	0.562074503	PARP1	TP53TG1	0.84403644	GMFB	H19	1
CCPG1	DLEU2	0.561368799	PARP12	RHPN1-AS1	0.799260853	GMFB	SNHG5	1
TET1	ZNRD1-AS1	0.561277919	PARP16	HCP5	0.74714161	GMFB	ZNRD1-AS1	1
CCDC50	SNHG5	0.560891523	PARP3	MCM3AP-AS1	0.99300875	GMNN	KTN1-AS1	1
MRPL39	SNHG3	0.560536741	PARP9	SNHG5	0.649351568	GMNN	SCARNA9	1
SLC4A7	ZNRD1-AS1	0.560428295	PATZ1	H19	0.572679058	GMPPB	HCP5	1
RELL1	HCP5	0.560270726	PATZ1	KTN1-AS1	0.999044372	GMPR	HCP5	1
ZEB1	SNHG3	0.560113655	PATZ1	MCM3AP-AS1	0.990038441	GMPS	HCP5	1
PAPSS2	SNHG5	0.559887683	PATZ1	ZNRD1-AS1	0.941221951	GNA12	HCP5	1
STX3	ZNRD1-AS1	0.559559574	PAXIP1	H19	0.637921474	GNA12	MCM3AP-AS1	1
KIF13A	H19	0.559463741	PAXIP1	SNHG3	0.519179623	GNAI1	HCP5	1
PRDM5	TPT1-AS1	0.55945118	PBLD	ZNRD1-AS1	0.984522077	GNAI1	KTN1-AS1	1

C17orf75	ZNRD1-AS1	0.559237139	PBX3	H19	0.729239854	GNAI1	MCM3AP-AS1	1
KLHL3	SNHG3	0.559080061	PBX3	LINC00467	0.864982411	GNAI1	SCARNA9	1
PRDM2	RUSC1-AS1	0.559059367	PBX3	MCM3AP-AS1	0.943183729	GNAI1	SNHG3	1
FADD	RHPN1-AS1	0.558992988	PBX3	ZNRD1-AS1	0.86725814	GOLPH3	H19	1
GLCCI1	SNHG5	0.558913569	PBXIP1	H19	0.502409441	GOLPH3	RUSC1-AS1	1
PLXNA2	TPT1-AS1	0.558888615	PBXIP1	HCP5	0.587617527	GOLPH3	ZNRD1-AS1	1
TBC1D17	HCP5	0.558531577	PBXIP1	LINC00467	0.831574872	GOLT1B	DLEU2	1
ACOT12	HCP5	0.558525783	PBXIP1	ZNRD1-AS1	0.940918801	GOLT1B	H19	1
ADAM10	SNHG5	0.557884268	PC	SNHG3	0.723006444	GOLT1B	TPT1-AS1	1
PTPN12	MCM3AP-AS1	0.557702884	PCDH9	HCP5	0.754996241	GOLT1B	ZNRD1-AS1	1
RTKN2	ZNRD1-AS1	0.557498833	PCGF2	SCARNA9	0.637879754	GORAB	HCP5	1
BAG2	KTN1-AS1	0.557492747	PCGF2	SNHG3	0.793923912	GORAB	KTN1-AS1	1
MRRF	ZNRD1-AS1	0.557274934	PCMTD1	H19	0.619646371	GORAB	MCM3AP-AS1	1
ZNF32	RUSC1-AS1	0.557219225	PCMTD1	TPT1-AS1	0.80414453	GORAB	SNHG3	1
ELK3	KTN1-AS1	0.55717795	PCMTD2	H19	0.591700415	GPAM	HCP5	1
UQCRC1	HCP5	0.557169822	PCMTD2	KTN1-AS1	0.559125487	GPAM	KTN1-AS1	1
AMACR	SNHG5	0.556748982	PCMTD2	LINC00467	0.888952173	GPAM	MCM3AP-AS1	1
DRAM1	MCM3AP-AS1	0.556709587	PCSK5	MCM3AP-AS1	0.96503936	GPAM	TP53TG1	1
DOCK4	HCP5	0.556310841	PCTP	SNHG3	0.643246704	GPD1L	HCP5	1
FOSB	KTN1-AS1	0.556238523	PCTP	SNHG5	0.562104337	GPD1L	SNHG3	1
DNAJC7	DLEU2	0.556146404	PCYOX1L	H19	0.550898509	GPR107	H19	1
APP	SNHG5	0.555804677	PCYOX1L	MCM3AP-AS1	0.723455122	GPR107	ZNRD1-AS1	1
SVIL	RUSC1-AS1	0.555719796	PCYOX1L	ZNRD1-AS1	0.596403328	GPR124	DLEU2	1
BRIX1	TPT1-AS1	0.555702373	PDCD4	KTN1-AS1	0.986925805	GPR124	TPT1-AS1	1
DOCK9	HCP5	0.555364986	PDCD4	MCM3AP-AS1	0.997350563	GPR124	ZNRD1-AS1	1
GSTCD	SNHG3	0.555179068	PDCD4	ZNRD1-AS1	0.971089928	GPR126	DLEU2	1
RNF150	MCM3AP-AS1	0.555146812	PDCD5	SNHG3	0.880187969	GPR126	SNHG5	1
BAG4	SNHG3	0.555100405	PDE4B	SNHG3	0.812630424	GPR126	TPT1-AS1	1
PCYOX1L	SNHG3	0.554984033	PDE4B	SNHG5	0.842046621	GPR126	ZNRD1-AS1	1
DTNA	TP53TG1	0.554819548	PDGFD	H19	0.662746856	GPR137B	H19	1
KIF18A	DLEU2	0.554709748	PDGFD	HCP5	0.840941337	GPR137B	SNHG5	1
C12orf4	H19	0.554678054	PDGFRB	DLEU2	0.795447807	GPR137B	ZNRD1-AS1	1
ENC1	H19	0.554578887	PDIK1L	H19	0.776863772	GPR137C	HCP5	1
GNA12	HCP5	0.554528212	PDIK1L	HCP5	0.602542967	GPR137C	SCARNA9	1
KCTD15	HCP5	0.554364309	PDIK1L	MCM3AP-AS1	0.867802759	GPR137C	TP53TG1	1
GPR137C	RUSC1-AS1	0.55395927	PDIK1L	ZNRD1-AS1	0.748582781	GPR180	MCM3AP-AS1	1
PRAME	KTN1-AS1	0.553958674	PDK1	H19	0.93544551	GPR183	H19	1
SOCS5	TP53TG1	0.553847637	PDK1	TP53TG1	0.842459745	GPR183	ZNRD1-AS1	1
MYO1H	SCARNA9	0.553650684	PDK4	HCP5	0.827161775	GPR56	HCP5	1
SSTR2	DLEU2	0.553602206	PDLIM1	HCP5	0.871571457	GPR56	SNHG3	1
CSTF3	ZNRD1-AS1	0.553538833	PDRG1	H19	0.981641291	GPR63	HCP5	1
C6orf120	LINC00467	0.553430152	PEAK1	TPT1-AS1	0.682673467	GPR63	MCM3AP-AS1	1
CDCA7	SNHG3	0.553414925	PECR	SCARNA9	0.6016195	GPRIN3	H19	1
ARFGEF2	H19	0.553232816	PER3	H19	0.555101505	GPRIN3	LINC00467	1
FAM63B	H19	0.553101297	PER3	ZNRD1-AS1	0.711570088	GPRIN3	ZNRD1-AS1	1
PDIK1L	SNHG3	0.552736833	PERP	SNHG3	0.718605006	GPSM2	H19	1
NAA50	ZNRD1-AS1	0.552668915	PEX6	TP53TG1	0.848767148	GPSM2	SNHG5	1
CTSF	LINC00467	0.552623118	PFKFB3	SNHG3	0.722083201	GPSM2	ZNRD1-AS1	1
CCDC82	H19	0.552622574	PGAP2	H19	0.70636377	GPT2	HCP5	1
HMBOX1	H19	0.55243609	PGAP2	HCP5	0.802908662	GPT2	MCM3AP-AS1	1
CENPP	ZNRD1-AS1	0.552279503	PGBD2	H19	0.516177083	GRB10	MCM3AP-AS1	1

SAMD9L	H19	0.552096337	PGBD2	TP53TG1	0.719222644	GRK5	HCP5	1
PPIH	MCM3AP-AS1	0.552038593	PGM3	SNHG5	0.507838442	GRK5	KTN1-AS1	1
NDFIP1	SNHG5	0.551934195	PGPEP1	TPT1-AS1	0.910868229	GRK5	MCM3AP-AS1	1
LCLAT1	HCP5	0.551853835	PHACTR1	H19	0.77579623	GRK5	SNHG3	1
ZNF397	ZNRD1-AS1	0.551474056	PHF1	ZNRD1-AS1	0.910916471	GRN	H19	1
SPEF2	SNHG3	0.551459184	PHF19	H19	0.780615442	GRN	ZNRD1-AS1	1
TUBE1	RHPN1-AS1	0.55139295	PHF19	KTN1-AS1	0.816214893	GRPEL1	SCARNA9	1
DNAJA3	KTN1-AS1	0.551341119	PHF19	LINC00467	0.998067691	GRPEL1	SNHG3	1
LYAR	ZNRD1-AS1	0.551286263	PHF19	MCM3AP-AS1	0.707290537	GRPEL2	HCP5	1
GAB1	RUSC1-AS1	0.550849195	PHF5A	H19	0.502650336	GRPEL2	SNHG3	1
TRMT6	ZNRD1-AS1	0.550596968	PHF5A	SNHG3	0.637918736	GSG2	HCP5	1
MAPKAP1	H19	0.550353693	PHF6	SNHG3	0.980650745	GSG2	KTN1-AS1	1
FHL1	HCP5	0.550341361	PHF6	SNHG5	0.808384188	GSPT2	MCM3AP-AS1	1
TDG	H19	0.55008361	PHGDH	HCP5	0.701958688	GSR	SNHG5	1
ZNF521	MCM3AP-AS1	0.550081753	PHKA1	SCARNA9	0.53415591	GSTCD	H19	1
TRIO	MCM3AP-AS1	0.550039973	PHYH	HCP5	0.812124716	GSTCD	LINC00467	1
IGFBP7	HCP5	0.549896758	PI4K2B	SNHG5	0.88450133	GSTCD	ZNRD1-AS1	1
ZFP36L2	MCM3AP-AS1	0.54987503	PIBF1	KTN1-AS1	0.731986937	GSTK1	RHPN1-AS1	1
NBEAL1	ZNRD1-AS1	0.549833229	PIBF1	TPT1-AS1	0.830907127	GTF2F2	KTN1-AS1	1
PCTP	SNHG5	0.549813263	PICALM	SNHG3	0.774650838	GTF2F2	MCM3AP-AS1	1
FAM126A	HCP5	0.549733325	PICALM	SNHG5	0.753934134	GTF2F2	SNHG3	1
ABCD3	H19	0.549722411	PIEZO2	DLEU2	0.738353343	GTF2I	MCM3AP-AS1	1
BLVRB	SNHG5	0.549679811	PIK3CD	TPT1-AS1	0.64858835	GTF2I	SNHG3	1
ATL2	DLEU2	0.549656087	PIK3IP1	MCM3AP-AS1	0.947275931	GTF2IRD2	HCP5	1
TIMP2	HCP5	0.549311566	PIK3IP1	RUSC1-AS1	0.551514559	GTPBP4	TPT1-AS1	1
IQCA1	SNHG3	0.549306983	PIK3IP1	ZNRD1-AS1	0.990896024	GTPBP4	ZNRD1-AS1	1
EIF2AK4	ZNRD1-AS1	0.549272529	PIK3R1	H19	0.692391735	GTPBP8	KTN1-AS1	1
SAMD4A	MCM3AP-AS1	0.549261191	PIK3R1	HCP5	0.60623607	GTPBP8	MCM3AP-AS1	1
CREG1	HCP5	0.549019478	PIK3R1	KTN1-AS1	0.978581042	GTPBP8	RHPN1-AS1	1
MGLL	HCP5	0.54890865	PIK3R1	MCM3AP-AS1	0.928707956	GTSE1	KTN1-AS1	1
GPT2	ZNRD1-AS1	0.548769866	PIK3R1	TP53TG1	0.743508231	GTSE1	MCM3AP-AS1	1
WASL	TP53TG1	0.548714768	PIK3R1	ZNRD1-AS1	0.834083859	GUCY1A3	HCP5	1
BMP8B	HCP5	0.548713274	PIM2	SNHG3	0.634156839	GUF1	SNHG3	1
EMP1	SNHG5	0.548516419	PINK1	HCP5	0.542128546	GULP1	HCP5	1
ZDHHC17	ZNRD1-AS1	0.548462299	PINK1	TP53TG1	0.70218093	GULP1	SCARNA9	1
AMOT	MCM3AP-AS1	0.548287768	PIP5K1A	SNHG3	0.505539504	H2AFJ	HCP5	1
TTYH3	H19	0.547947121	PIP5K1B	HCP5	0.839809742	H2AFX	MCM3AP-AS1	1
BSDC1	H19	0.54793892	PITPNC1	LINC00467	0.942784088	H2AFY	HCP5	1
ATAD2B	DLEU2	0.547832236	PKMYT1	H19	0.968985514	HABP4	HCP5	1
TOX	RHPN1-AS1	0.547775767	PKP4	KTN1-AS1	0.959048351	HADH	MCM3AP-AS1	1
PIK3CA	ZNRD1-AS1	0.547760844	PKP4	MCM3AP-AS1	0.896409915	HADH	SNHG3	1
DCLK2	H19	0.547587744	PLA2G4A	SNHG3	0.953935955	HAPLN3	H19	1
FAM117B	LINC00467	0.547529364	PLAA	SNHG5	0.815704269	HAS2	H19	1
MRPL37	SNHG5	0.547385613	PLAT	SNHG3	0.792099999	HAS2	SNHG5	1
DUSP22	KTN1-AS1	0.547261366	PLCL2	H19	0.611972777	HAS2	ZNRD1-AS1	1
RRAGD	MCM3AP-AS1	0.54704268	PLCL2	MCM3AP-AS1	0.962886111	HAT1	HCP5	1
LAMP3	LINC00467	0.546806542	PLCL2	ZNRD1-AS1	0.8893157	HAT1	SCARNA9	1
GABARAP	HCP5	0.546647532	PLD2	H19	0.701749681	HAT1	SNHG3	1
TJP2	H19	0.546369965	PLEC	RUSC1-AS1	0.668764561	HAUS1	SNHG3	1
VGLL4	SNHG3	0.54629242	PLEKHA1	HCP5	0.873965818	HAUS2	MCM3AP-AS1	1
DPYSL3	MCM3AP-AS1	0.546136927	PLEKHB1	ZNRD1-AS1	0.712084557	HAUS4	HCP5	1

ARL5B	HCP5	0.545986763	PLK1	KTN1-AS1	0.62448702	HAUS6	SNHG3	1
CBX5	MCM3AP-AS1	0.545894134	PLK2	H19	0.832091702	HAUS8	HCP5	1
CAB39L	HCP5	0.545847442	PLK2	ZNRD1-AS1	0.74795469	HAUS8	KTN1-AS1	1
AGFG2	H19	0.545696315	PLK4	H19	0.831953997	HBEGF	H19	1
UHRF1BP1	HCP5	0.545685767	PLK4	HCP5	0.544543484	HBEGF	RUSC1-AS1	1
NAGPA	HCP5	0.54565613	PLK4	TP53TG1	0.980799179	HBEGF	SNHG5	1
LRP1	HCP5	0.545212923	PLLP	ZNRD1-AS1	0.887295457	HBEGF	ZNRD1-AS1	1
PHKA1	SNHG5	0.545053438	PLS1	SNHG5	0.561189313	HBS1L	DLEU2	1
PSIP1	DLEU2	0.544487817	PLSCR4	H19	0.563312889	HBS1L	H19	1
HEMGN	SCARNA9	0.544114654	PLSCR4	HCP5	0.775468981	HBS1L	SNHG5	1
SUFU	SNHG3	0.543942235	PLSCR4	LINC00467	0.767663086	HBS1L	TPT1-AS1	1
OTUD6B	SNHG3	0.543896547	PLXNA2	TPT1-AS1	0.599212518	HBS1L	ZNRD1-AS1	1
CCDC43	HCP5	0.543843112	PMEPA1	DLEU2	0.608335537	HCCS	H19	1
PAK1IP1	HCP5	0.543783741	PMM2	ZNRD1-AS1	0.801965868	HCCS	ZNRD1-AS1	1
SERP1	SNHG3	0.543765581	PNKD	H19	0.613152491	HCFC2	MCM3AP-AS1	1
KBTBD6	SNHG3	0.543575964	PNKD	HCP5	0.764401939	HCFC2	SCARNA9	1
ZNF217	RUSC1-AS1	0.543518804	PNKD	TPT1-AS1	0.516843771	HCFC2	TP53TG1	1
DLAT	RHPN1-AS1	0.543466029	PNKD	ZNRD1-AS1	0.769371697	HEBP1	HCP5	1
SHB	KTN1-AS1	0.543192317	PNP	SNHG3	0.969068012	HEBP1	KTN1-AS1	1
PKMYT1	H19	0.543130561	PNPLA4	H19	0.541195318	HECA	H19	1
TMEM201	HCP5	0.543083797	PNRC1	SNHG5	0.526169106	HECA	SNHG5	1
FAM126A	SCARNA9	0.543037657	POC1A	H19	0.887220585	HECA	ZNRD1-AS1	1
MRPL52	H19	0.542787608	POLA1	H19	0.795559873	HECTD3	SNHG5	1
IGF2BP3	SNHG3	0.542602277	POLD3	H19	0.798651229	HECW2	H19	1
NEGR1	HCP5	0.542364417	POLD3	HCP5	0.711882858	HECW2	ZNRD1-AS1	1
TNFSF9	MCM3AP-AS1	0.542292712	POLD3	MCM3AP-AS1	0.530368065	HEG1	HCP5	1
AHNAK	SNHG3	0.542029965	POLE	TPT1-AS1	0.829581313	HEG1	TP53TG1	1
GM2A	SNHG5	0.541884929	POLE3	H19	0.785556856	HELLS	MCM3AP-AS1	1
SGCB	SNHG3	0.54183092	POLE3	HCP5	0.507357148	HELLS	SNHG3	1
TMEM25	ZNRD1-AS1	0.541799344	POLE3	LINC00467	0.753460185	HERPUD1	KTN1-AS1	1
FERMT1	MCM3AP-AS1	0.541762211	POLQ	H19	0.953647343	HERPUD1	SNHG3	1
RAB5B	HCP5	0.541676939	POLQ	LINC00467	0.607427562	HERPUD1	TP53TG1	1
PITPNC1	SNHG5	0.541656486	POLR3G	HCP5	0.785665672	HEY1	H19	1
STK32B	MCM3AP-AS1	0.541555352	PPAP2B	SNHG3	0.948340444	HEY1	ZNRD1-AS1	1
WWTR1	SNHG5	0.541314379	PPAP2B	SNHG5	0.854970252	HGF	MCM3AP-AS1	1
STUB1	SNHG5	0.541138688	PPAPDC2	HCP5	0.841443463	HGF	SNHG3	1
E2F5	SNHG3	0.541109487	PPARA	HCP5	0.797269045	HIST1H2BG	HCP5	1
PRKRA	SNHG3	0.541055824	PPARA	TP53TG1	0.502887163	HIST1H3B	HCP5	1
NIPAL4	TP53TG1	0.541037127	PPARA	ZNRD1-AS1	0.85550449	HIST2H2BE	MCM3AP-AS1	1
ITM2B	RHPN1-AS1	0.540799939	PPARD	MCM3AP-AS1	0.571261783	HIVEP1	DLEU2	1
TRIP10	ZNRD1-AS1	0.540749682	PPARD	RUSC1-AS1	0.58234569	HIVEP1	SNHG5	1
ZBTB10	TPT1-AS1	0.540274795	PPARD	ZNRD1-AS1	0.6878802	HIVEP1	TPT1-AS1	1
C11orf74	KTN1-AS1	0.54020038	PPIF	RHPN1-AS1	0.983954874	HIVEP1	ZNRD1-AS1	1
BAX	ZNRD1-AS1	0.539797222	PPIH	H19	0.915024652	HIVEP2	H19	1
CDYL2	RUSC1-AS1	0.539569222	PPM1G	H19	0.654554106	HIVEP2	LINC00467	1
PIK3R1	MCM3AP-AS1	0.539372839	PPM1G	HCP5	0.819106513	HIVEP2	RUSC1-AS1	1
RALB	ZNRD1-AS1	0.539316343	PPM1H	MCM3AP-AS1	0.763207816	HIVEP2	ZNRD1-AS1	1
HAT1	SCARNA9	0.539138147	PPM1H	ZNRD1-AS1	0.721470342	HK2	MCM3AP-AS1	1
CYBRD1	MCM3AP-AS1	0.539137148	PPOX	TPT1-AS1	0.610988979	HLA-C	KTN1-AS1	1
EIF2S2	HCP5	0.539032653	PPP1R3E	KTN1-AS1	0.977223272	HLA-DPB1	SNHG5	1
SGMS1	LINC00467	0.538597424	PPP2R5C	SNHG3	0.71709167	HLA-DQB1	SNHG3	1

CASK	MCM3AP-AS1	0.538496087	PPP2R5C	SNHG5	0.791343254	HLA-E	MCM3AP-AS1	1
PDK1	SNHG3	0.538306463	PPRC1	KTN1-AS1	0.991422605	HLA-F	H19	1
GLI2	DLEU2	0.538204903	PPRC1	MCM3AP-AS1	0.984851383	HLA-G	H19	1
TRERF1	SNHG3	0.538032658	PPRC1	ZNRD1-AS1	0.939156177	HLA-G	ZNRD1-AS1	1
SLC4A7	H19	0.538009465	PRC1	KTN1-AS1	0.504766816	HLTF	MCM3AP-AS1	1
SALL2	SNHG3	0.53792801	PRDM1	SNHG3	0.650801365	HLTF	SNHG3	1
KIF2C	H19	0.53782888	PRDM16	SNHG3	0.727532335	HMBOX1	H19	1
DCUN1D3	HCP5	0.537728435	PRDM2	KTN1-AS1	0.767129432	HMBOX1	LINC00467	1
DPY19L3	ZNRD1-AS1	0.537414227	PRDM2	MCM3AP-AS1	0.836158754	HMBOX1	SNHG5	1
ASPH	MCM3AP-AS1	0.537400452	PRDM2	ZNRD1-AS1	0.885443369	HMBOX1	ZNRD1-AS1	1
RNF19B	HCP5	0.537281014	PRDX3	SNHG5	0.801654347	HMGA1	MCM3AP-AS1	1
TMEM80	H19	0.536977285	PREPL	SNHG3	0.712390758	HMGA1	SNHG3	1
DZIP1	MCM3AP-AS1	0.536843458	PREPL	SNHG5	0.746216084	HMGB1	HCP5	1
PDE4B	SNHG3	0.536841945	PREX1	ZNRD1-AS1	0.99515811	HMGB2	HCP5	1
PRKRA	HCP5	0.536047982	PRICKLE1	SNHG3	0.982661749	HMGB2	KTN1-AS1	1
GTF2F2	MCM3AP-AS1	0.535972639	PRICKLE1	SNHG5	0.774792315	HMGB2	MCM3AP-AS1	1
CCDC15	ZNRD1-AS1	0.535878629	PRIM1	H19	0.693061699	HMGB2	SCARNA9	1
SNX9	DLEU2	0.53580388	PRIM1	HCP5	0.660211455	HMGB2	SNHG3	1
GULP1	SCARNA9	0.535801838	PRIM2	LINC00467	0.67265668	HMGB2	TP53TG1	1
ARHGAP20	SNHG3	0.535785317	PRKACB	HCP5	0.938426592	HMGB3	H19	1
CPNE8	HCP5	0.535719577	PRKACB	KTN1-AS1	0.788732133	HMGB3	LINC00467	1
TARBP2	HCP5	0.535690511	PRKACB	LINC00467	0.860881667	HMGB3	ZNRD1-AS1	1
UBE2H	SCARNA9	0.535358034	PRKACB	MCM3AP-AS1	0.734750633	HMGXB4	HCP5	1
CREM	TPT1-AS1	0.535080827	PRKACB	ZNRD1-AS1	0.642477491	HMGXB4	KTN1-AS1	1
ARHGAP32	ZNRD1-AS1	0.535049279	PRKAR2B	HCP5	0.928620525	HMGXB4	MCM3AP-AS1	1
KIF2A	MCM3AP-AS1	0.535033089	PRKAR2B	TP53TG1	0.80269401	HMMR	KTN1-AS1	1
CPNE8	SNHG5	0.534860378	PRKAR2B	ZNRD1-AS1	0.525646727	HN1	HCP5	1
UQCC1	MCM3AP-AS1	0.534674335	PRKCA	SNHG3	0.920615573	HN1L	HCP5	1
SCAI	MCM3AP-AS1	0.534608061	PRKDC	SNHG3	0.811596532	HN1L	RHPN1-AS1	1
MRPL35	DLEU2	0.534419644	PRKRA	SNHG3	0.572316319	HN1L	SNHG3	1
STK17A	HCP5	0.533372446	PROS1	SCARNA9	0.532615524	HNRNPA3	DLEU2	1
HPS3	SNHG5	0.533311052	PRR11	H19	0.811322983	HNRNPA3	TPT1-AS1	1
P4HA1	SCARNA9	0.533259069	PRR11	ZNRD1-AS1	0.728908538	HNRNPAB	KTN1-AS1	1
JMJD8	SNHG3	0.533177539	PRR15	HCP5	0.975740537	HNRNPAB	MCM3AP-AS1	1
ITPR1	RUSC1-AS1	0.532723052	PRR15	LINC00467	0.830925686	HNRNPAB	SNHG3	1
CREB3L4	RUSC1-AS1	0.532701789	PRR15	ZNRD1-AS1	0.527574767	HNRNPR	HCP5	1
MEST	RHPN1-AS1	0.532675673	PRR16	DLEU2	0.986110454	HNRNPR	MCM3AP-AS1	1
IPPK	ZNRD1-AS1	0.532561687	PRR16	SCARNA9	0.564066588	HNRNPR	SCARNA9	1
ATAD2B	MCM3AP-AS1	0.532462069	PRRG1	SNHG3	0.929433045	HNRNPR	SNHG3	1
ISCU	LINC00467	0.532344016	PRRG1	SNHG5	0.714053404	HNRNPR	TP53TG1	1
ISCU	SNHG5	0.531940307	PRRT3	MCM3AP-AS1	0.947889953	HOMER1	HCP5	1
TOMM22	SNHG5	0.531844203	PRUNE	LINC00467	0.51030539	HOMER1	MCM3AP-AS1	1
PRKCB	HCP5	0.531750282	PSAT1	H19	0.969596366	HOMER1	SNHG3	1
ANKRA2	RUSC1-AS1	0.531270163	PSAT1	LINC00467	0.701569487	HOMER1	TP53TG1	1
ZXDB	DLEU2	0.531176664	PSAT1	TP53TG1	0.884285405	HOPX	TP53TG1	1
HIVEP1	TPT1-AS1	0.530974465	PSD3	SNHG3	0.996024002	HOXA10	HCP5	1
PRKCA	MCM3AP-AS1	0.530789941	PSD3	SNHG5	0.659131081	HOXA10	MCM3AP-AS1	1
ANKRD46	HCP5	0.53070946	PSIP1	KTN1-AS1	0.917570728	HOXA10	SNHG3	1
TOMM34	MCM3AP-AS1	0.53067824	PSIP1	MCM3AP-AS1	0.972247271	HOXA3	H19	1
RAB2B	ZNRD1-AS1	0.530663973	PSIP1	TPT1-AS1	0.936233358	HOXA3	ZNRD1-AS1	1
CD164	MCM3AP-AS1	0.530345067	PSIP1	ZNRD1-AS1	0.999108286	HOXA4	HCP5	1

ZW10	HCP5	0.530245129	PSMB3	SNHG3	0.948819554	HOXA5	MCM3AP-AS1	1
SIX4	LINC00467	0.529994605	PSMB7	SNHG3	0.929649388	HOXA5	SNHG3	1
AIFM2	SNHG5	0.529981666	PSMC6	SNHG5	0.758772047	HOXA7	MCM3AP-AS1	1
C5orf30	KTN1-AS1	0.529849711	PSMD1	SNHG5	0.80665754	HOXA7	SNHG3	1
ATG14	H19	0.52982347	PSMD11	SNHG3	0.889025979	HOXA9	HCP5	1
GLCE	SNHG3	0.529659509	PSMD12	SNHG3	0.984144706	HOXA9	SCARNA9	1
POC1A	H19	0.529589883	PSMD14	SNHG5	0.828229242	HOXA9	SNHG3	1
FER	ZNRD1-AS1	0.529516769	PSMD3	SNHG3	0.89160161	HOXA9	TP53TG1	1
MAP3K5	H19	0.529430658	PSMD4	SNHG3	0.942108181	HOXB2	SNHG3	1
SAP30BP	H19	0.52942288	PSME4	DLEU2	0.529725074	HOXB3	HCP5	1
SOCS5	SNHG5	0.528805843	PSRC1	H19	0.857722503	HOXB3	SNHG3	1
SESN1	HCP5	0.528776302	PTDSS1	HCP5	0.638159062	HOXB4	KTN1-AS1	1
KIF13B	ZNRD1-AS1	0.528460037	PTEN	H19	0.95361274	HOXB4	MCM3AP-AS1	1
NUPL1	DLEU2	0.528422336	PTEN	KTN1-AS1	0.753688076	HPDL	SNHG3	1
DENND4A	TPT1-AS1	0.528175188	PTEN	LINC00467	0.933714258	HPGD	DLEU2	1
ARL5B	RUSC1-AS1	0.527929143	PTEN	MCM3AP-AS1	0.635403085	HPS3	MCM3AP-AS1	1
JARID2	H19	0.527774741	PTGER2	H19	0.676922249	HPS3	SCARNA9	1
PNP	SNHG3	0.527756496	PTGER2	LINC00467	0.953886517	HRSP12	KTN1-AS1	1
STEAP2	SNHG3	0.52775041	PTGER2	ZNRD1-AS1	0.811656733	HS2ST1	HCP5	1
AMER1	ZNRD1-AS1	0.527698954	PTGER4	SNHG5	0.534848017	HS2ST1	KTN1-AS1	1
PGP	H19	0.527479279	PTGFRN	H19	0.596263696	HS2ST1	MCM3AP-AS1	1
ITGB8	MCM3AP-AS1	0.527397425	PTGFRN	HCP5	0.877245379	HS2ST1	SCARNA9	1
SENP5	HCP5	0.527373093	PTGFRN	KTN1-AS1	0.618599956	HS3ST1	H19	1
GCSAM	HCP5	0.527369855	PTGFRN	LINC00467	0.913961004	HS3ST3B1	H19	1
PPARD	ZNRD1-AS1	0.527166423	PTGFRN	TP53TG1	0.899467867	HS3ST3B1	SNHG5	1
FAM124A	RHPN1-AS1	0.527024181	PTGS2	SNHG5	0.914434593	HS3ST3B1	ZNRD1-AS1	1
ZBTB38	SNHG5	0.526954366	PTK2B	RHPN1-AS1	0.807375407	HS6ST2	KTN1-AS1	1
CCNC	SNHG5	0.526875824	PTPDC1	DLEU2	0.759268162	HS6ST2	MCM3AP-AS1	1
MYO1E	HCP5	0.526602337	PTPDC1	RUSC1-AS1	0.660036284	HS6ST2	SCARNA9	1
MAF	SNHG3	0.526422824	PTPLB	HCP5	0.678653716	HSD17B11	H19	1
CPNE8	ZNRD1-AS1	0.526186391	PTPN1	SNHG3	0.911520555	HSD17B11	ZNRD1-AS1	1
MYH9	DLEU2	0.526184652	PTPN13	H19	0.893383316	HSP90B1	KTN1-AS1	1
HLA-DQB1	RUSC1-AS1	0.525869318	PTPN13	LINC00467	0.946857049	HSP90B1	MCM3AP-AS1	1
USP16	MCM3AP-AS1	0.525844605	PTPN13	TP53TG1	0.868012188	HSPA13	HCP5	1
LDB1	ZNRD1-AS1	0.525728724	PTPN13	ZNRD1-AS1	0.65727487	HSPA13	SCARNA9	1
PREPL	MCM3AP-AS1	0.525720923	PTPN14	H19	0.505559352	HSPA13	SNHG3	1
CLIP4	SNHG5	0.525673209	PTPN18	ZNRD1-AS1	0.910576934	HSPA14	HCP5	1
PTGER4	KTN1-AS1	0.525486482	PTPN4	HCP5	0.887755123	HSPA14	SNHG3	1
ZNF395	KTN1-AS1	0.525435724	PTPN9	H19	0.686069551	HSPA4	SCARNA9	1
C6orf211	KTN1-AS1	0.525372124	PTPN9	HCP5	0.624621241	HSPA4L	HCP5	1
PLXNC1	MCM3AP-AS1	0.525300542	PTPN9	LINC00467	0.750513481	HSPA4L	MCM3AP-AS1	1
HS6ST2	KTN1-AS1	0.525128232	PTPRA	SNHG5	0.74244041	HSPA9	MCM3AP-AS1	1
PTPDC1	MCM3AP-AS1	0.525057886	PTPRF	TP53TG1	0.621002753	HSPA9	TP53TG1	1
PBX3	RHPN1-AS1	0.524969219	PTPRJ	RHPN1-AS1	0.920300652	HSPB11	HCP5	1
CDCA8	DLEU2	0.524577661	PTPRJ	SNHG5	0.64537004	HSPB11	SNHG3	1
ARL13B	TPT1-AS1	0.524572288	PTPRK	SNHG3	0.54155817	HSPD1	HCP5	1
RIOK3	HCP5	0.524570996	PTPRM	SNHG3	0.660921266	HSPD1	KTN1-AS1	1
KIAA0101	TPT1-AS1	0.524533998	PUS7	H19	0.577947425	HSPD1	MCM3AP-AS1	1
GDE1	RUSC1-AS1	0.524222385	PUS7	HCP5	0.893740949	HSPE1	RUSC1-AS1	1
TOMM34	ZNRD1-AS1	0.524062183	PVRL2	SNHG3	0.922782756	HSPH1	HCP5	1
MAML3	ZNRD1-AS1	0.524057777	PXK	H19	0.74223671	HSPH1	KTN1-AS1	1

DUT	SCARNA9	0.523657022	PXK	HCP5	0.659958871	HSPH1	MCM3AP-AS1	1
PIK3C2B	LINC00467	0.523568009	PXK	TPT1-AS1	0.51618172	HSPH1	SNHG3	1
RTKN2	DLEU2	0.523393616	PXK	ZNRD1-AS1	0.743667734	HTRA3	H19	1
RPF2	RUSC1-AS1	0.523322266	PYGO2	H19	0.805253246	HTRA3	LINC00467	1
LRBA	HCP5	0.523223594	QARS	ZNRD1-AS1	0.720303457	HTRA3	ZNRD1-AS1	1
GCNT2	DLEU2	0.523122104	QDPR	HCP5	0.561503462	HYOU1	MCM3AP-AS1	1
C6orf120	MCM3AP-AS1	0.523118761	QDPR	TP53TG1	0.758582488	IARS	MCM3AP-AS1	1
MREG	H19	0.523095655	RAB11FIP1	SNHG3	0.67641072	IARS	SCARNA9	1
FIGN	TP53TG1	0.522986994	RAB11FIP1	SNHG5	0.687224555	ICA1L	H19	1
SAMD4A	ZNRD1-AS1	0.52254997	RAB11FIP4	HCP5	0.939010764	ICA1L	ZNRD1-AS1	1
CENPA	DLEU2	0.522480388	RAB11FIP4	ZNRD1-AS1	0.650498932	ICAM1	H19	1
YEATS4	DLEU2	0.522265399	RAB11FIP5	SNHG3	0.797473437	ICAM1	ZNRD1-AS1	1
ENPP2	H19	0.522137455	RAB12	DLEU2	0.550773079	ICMT	HCP5	1
ANKRD26	H19	0.521655449	RAB12	SNHG5	0.899856908	ICMT	SCARNA9	1
ATP2B1	RUSC1-AS1	0.521628657	RAB3D	SNHG5	0.64202204	ICMT	SNHG3	1
SKIL	ZNRD1-AS1	0.520914951	RAB5B	RUSC1-AS1	0.533821468	ID1	HCP5	1
RASGEF1B	SNHG5	0.520886162	RAB5B	SNHG5	0.828943563	IER3	H19	1
EXPH5	TP53TG1	0.520528628	RABEPK	RHPN1-AS1	0.8159196	IER3	RUSC1-AS1	1
ADCY1	MCM3AP-AS1	0.520407746	RABGAP1	LINC00467	0.6626521	IER3	ZNRD1-AS1	1
THAP11	HCP5	0.520219068	RABGAP1	MCM3AP-AS1	0.987116163	IFI16	ZNRD1-AS1	1
TAB2	SCARNA9	0.519771326	RABGAP1	ZNRD1-AS1	0.985331269	IFI30	H19	1
ADAM28	KTN1-AS1	0.519477226	RABGAP1L	SNHG5	0.64601035	IFIT1	DLEU2	1
ENAH	HCP5	0.519422124	RACGAP1	H19	0.861797763	IFIT2	KTN1-AS1	1
NFIL3	SCARNA9	0.519017914	RACGAP1	HCP5	0.603683261	IFIT5	DLEU2	1
RPF2	HCP5	0.518776159	RAD18	H19	0.800772065	IFIT5	H19	1
TCF4	SNHG3	0.518572604	RAD18	HCP5	0.641222706	IFIT5	LINC00467	1
PHF6	SNHG3	0.518467828	RAD18	TP53TG1	0.987350519	IFIT5	RUSC1-AS1	1
MMD	SNHG3	0.518114623	RAD51AP1	H19	0.979008844	IFIT5	TPT1-AS1	1
TNFAIP2	ZNRD1-AS1	0.518095817	RAD51AP1	LINC00467	0.773797849	IFIT5	ZNRD1-AS1	1
GRK5	MCM3AP-AS1	0.517994163	RAD51B	H19	0.767063496	IFNAR2	DLEU2	1
CBX2	H19	0.517980274	RAD51C	H19	0.802205101	IFNAR2	H19	1
MRPL21	MCM3AP-AS1	0.517928536	RAD51C	LINC00467	0.721521861	IFNAR2	LINC00467	1
HOMER1	TP53TG1	0.517847158	RALA	HCP5	0.91557101	IFNAR2	SNHG5	1
MAP7	HCP5	0.517740101	RALGPS1	HCP5	0.966568801	IFNAR2	TPT1-AS1	1
ANGPT1	ZNRD1-AS1	0.517693043	RALGPS2	H19	0.638966034	IFNAR2	ZNRD1-AS1	1
CHEK2	ZNRD1-AS1	0.517607043	RALGPS2	ZNRD1-AS1	0.708322999	IFRD2	H19	1
TP53INP1	H19	0.517539087	RAPGEF5	RHPN1-AS1	0.878329122	IFRD2	ZNRD1-AS1	1
MICAL2	H19	0.516992816	RAPGEFL1	HCP5	0.885681096	IGBP1	SCARNA9	1
CMTM7	SNHG3	0.516925683	RAPGEFL1	ZNRD1-AS1	0.73903995	IGF1R	MCM3AP-AS1	1
VAV3	KTN1-AS1	0.516780134	RAPH1	DLEU2	0.557586903	IGF1R	RHPN1-AS1	1
MAP3K3	SNHG3	0.516652589	RAPH1	SNHG5	0.789691012	IGF1R	SCARNA9	1
TBC1D4	KTN1-AS1	0.516523971	RASGEF1A	H19	0.548356213	IGF1R	SNHG3	1
FSD1	MCM3AP-AS1	0.516486649	RASGEF1A	HCP5	0.76357589	IGF2BP2	HCP5	1
SBF2	ZNRD1-AS1	0.516462668	RASGEF1A	ZNRD1-AS1	0.816453579	IGF2BP3	H19	1
MORN2	HCP5	0.516194751	RASGEF1B	SNHG5	0.826925039	IGF2BP3	LINC00467	1
METTL2A	H19	0.515910556	RASSF5	SNHG3	0.758391666	IGF2BP3	SNHG5	1
TET3	TPT1-AS1	0.515736092	RAVER2	H19	0.684214861	IGF2BP3	ZNRD1-AS1	1
AHDC1	ZNRD1-AS1	0.515641303	RAVER2	HCP5	0.705771915	IGFBP5	HCP5	1
ZNF532	HCP5	0.514792269	RAVER2	LINC00467	0.972284217	IGFBP5	MCM3AP-AS1	1
ETS1	TP53TG1	0.514493447	RAVER2	TPT1-AS1	0.528305129	IGFBP5	RHPN1-AS1	1
DCLRE1B	SCARNA9	0.51432791	RAVER2	ZNRD1-AS1	0.765646607	IGFBP5	TP53TG1	1

TBC1D14	MCM3AP-AS1	0.513960446	RBL1	SNHG3	0.754214346	IGFBP7	DLEU2	1
STK17B	SNHG3	0.513834291	RBM10	H19	0.718090633	IGFBP7	H19	1
AP3S1	LINC00467	0.513771057	RBM47	RUSC1-AS1	0.553450931	IGFBP7	ZNRD1-AS1	1
C6orf120	HCP5	0.513479546	RBM47	SNHG5	0.854211638	IKZF2	DLEU2	1
GPD1L	HCP5	0.513430691	RBM8A	HCP5	0.864344903	IKZF2	SNHG5	1
TUBGCP5	TPT1-AS1	0.513381616	RBM8A	LINC00467	0.848960108	IKZF2	TPT1-AS1	1
ZNF138	KTN1-AS1	0.513246396	RBMS3	SNHG5	0.649718402	IKZF2	ZNRD1-AS1	1
TANK	TPT1-AS1	0.513167949	RBPMS	H19	0.623901682	IL11	KTN1-AS1	1
KIF3B	MCM3AP-AS1	0.512919396	RBPMS	HCP5	0.86495255	IL11	MCM3AP-AS1	1
PBK	H19	0.512857004	RBPMS	ZNRD1-AS1	0.514118592	IL12A	HCP5	1
SCPEP1	SCARNA9	0.512697858	RCAN3	MCM3AP-AS1	0.808419031	IL13RA1	HCP5	1
DOC2A	TPT1-AS1	0.512586535	RCAN3	ZNRD1-AS1	0.685766021	IL15	DLEU2	1
CAMLG	HCP5	0.512125212	RCC1	H19	0.933767201	IL15	H19	1
SAMD4A	DLEU2	0.51203973	RCOR1	DLEU2	0.684952969	IL15	SNHG5	1
NUMB	ZNRD1-AS1	0.512029756	RCOR1	RUSC1-AS1	0.834273411	IL15	TPT1-AS1	1
SEMA6D	RUSC1-AS1	0.511986479	RDH10	SNHG3	0.9275938	IL15	ZNRD1-AS1	1
ERICH1	MCM3AP-AS1	0.511771581	RDH13	MCM3AP-AS1	0.817678464	IL15RA	H19	1
MDK	RUSC1-AS1	0.511755583	RELL1	SNHG3	0.984172753	IL16	SCARNA9	1
ERICH1	DLEU2	0.51168511	RELL1	SNHG5	0.766202544	IL1R1	MCM3AP-AS1	1
HNRNPA3	DLEU2	0.511461529	REPS2	KTN1-AS1	0.719372079	IL1RAP	H19	1
DSCC1	SNHG3	0.511288905	REPS2	MCM3AP-AS1	0.716614293	IL1RAP	LINC00467	1
MDH1	SNHG5	0.511077899	RERE	MCM3AP-AS1	0.857882284	IL1RAP	SNHG5	1
NCOR2	ZNRD1-AS1	0.510854513	RERE	ZNRD1-AS1	0.90629343	IL1RAP	ZNRD1-AS1	1
CPA3	H19	0.510793837	REXO1	ZNRD1-AS1	0.789605543	IL21R	DLEU2	1
ARL5A	H19	0.510771243	RFC3	H19	0.602106261	IL21R	TPT1-AS1	1
ABL1	H19	0.510369452	RFC3	HCP5	0.8371724	IL2RB	ZNRD1-AS1	1
SLC35F1	DLEU2	0.510220391	RFC4	H19	0.879659066	IL32	SCARNA9	1
CDCA5	H19	0.510106347	RFC4	LINC00467	0.979163602	IL6	H19	1
RFC5	H19	0.509899973	RFC5	SNHG3	0.639896884	IL6	SNHG5	1
SYAP1	H19	0.509776901	RFK	H19	0.683292635	IL6	ZNRD1-AS1	1
CPEB2	RHPN1-AS1	0.509742971	RFK	HCP5	0.827378084	IL7	KTN1-AS1	1
KCTD12	HCP5	0.509581677	RFK	MCM3AP-AS1	0.585617545	IL7	MCM3AP-AS1	1
GPR21	SCARNA9	0.50953704	RFWD3	H19	0.760537608	ILDR1	H19	1
TMEM19	HCP5	0.509409856	RFX2	DLEU2	0.637644569	ILDR1	ZNRD1-AS1	1
NCKAP1	DLEU2	0.509330837	RFXAP	SNHG5	0.623922632	ILDR2	ZNRD1-AS1	1
ARAP2	MCM3AP-AS1	0.5092225	RGL1	SNHG3	0.719718995	IMPA2	TP53TG1	1
PDE5A	TPT1-AS1	0.509214059	RGMA	HCP5	0.932845173	INA	SCARNA9	1
TMEFF1	SNHG3	0.509207736	RGMA	TP53TG1	0.592291794	INADL	H19	1
CDK14	H19	0.509057193	RGMA	ZNRD1-AS1	0.677232554	INADL	LINC00467	1
PA2G4	ZNRD1-AS1	0.508445032	RHOB	DLEU2	0.617260349	INADL	SNHG5	1
POLQ	ZNRD1-AS1	0.508332261	RHOBTB1	HCP5	0.891373735	INCENP	HCP5	1
SEMA6D	KTN1-AS1	0.508268925	RHOU	DLEU2	0.544029249	INCENP	RHPN1-AS1	1
FSD1L	HCP5	0.507973672	RHOU	SNHG3	0.608501128	INF2	H19	1
KDM6B	HCP5	0.507429679	RHOU	SNHG5	0.746954119	ING2	TP53TG1	1
MFN2	H19	0.507396408	RIN2	KTN1-AS1	0.895974532	INPP1	HCP5	1
CCT2	SNHG3	0.506979864	RIN2	MCM3AP-AS1	0.95912323	INPP1	KTN1-AS1	1
TCP11L2	KTN1-AS1	0.506830927	RIN2	ZNRD1-AS1	0.996499402	INPP1	MCM3AP-AS1	1
MDFIC	HCP5	0.506278499	RIOK3	SNHG3	0.814008426	INPP5K	RHPN1-AS1	1
IL15	HCP5	0.506270043	RNASEL	H19	0.642971346	INSIG1	HCP5	1
MDFIC	TP53TG1	0.50562617	RNF115	ZNRD1-AS1	0.915164666	INSIG1	MCM3AP-AS1	1
MRPS35	KTN1-AS1	0.505458902	RNF150	MCM3AP-AS1	0.73189163	INSIG1	SNHG3	1

APPL1	MCM3AP-AS1	0.50543039	RNF150	ZNRD1-AS1	0.698417811	INSM1	KTN1-AS1	1
APPL1	HCP5	0.505421862	RNF170	SNHG5	0.768147629	INSM1	MCM3AP-AS1	1
EFNA4	SCARNA9	0.50539246	RNF187	HCP5	0.674296708	INSM1	TP53TG1	1
HIVEP1	ZNRD1-AS1	0.505190101	RNF187	LINC00467	0.988148671	INSR	SNHG5	1
LRRC28	SNHG3	0.504880202	RNF19A	SNHG3	0.508359057	INSR	ZNRD1-AS1	1
SESN3	HCP5	0.504707309	RNF213	TPT1-AS1	0.764658286	INTS6	HCP5	1
BAZ2B	RUSC1-AS1	0.50467323	RNF24	HCP5	0.931804776	INTS6	MCM3AP-AS1	1
COBLL1	KTN1-AS1	0.50461088	RNF24	MCM3AP-AS1	0.718688561	IPPK	KTN1-AS1	1
CENPF	ZNRD1-AS1	0.504497146	RNF24	ZNRD1-AS1	0.609857095	IPPK	MCM3AP-AS1	1
JUP	DLEU2	0.504437439	RNF44	TPT1-AS1	0.796668714	IQCA1	HCP5	1
SLC16A1	TP53TG1	0.504421165	RNF44	ZNRD1-AS1	0.557649784	IQCA1	SNHG3	1
NUCB2	HCP5	0.504300695	ROBO1	HCP5	0.676655717	IQGAP1	H19	1
PDK2	RUSC1-AS1	0.503951639	ROBO1	SCARNA9	0.54437494	IQGAP1	RUSC1-AS1	1
KIAA0226	H19	0.503938973	ROBO1	TP53TG1	0.52876091	IQGAP1	SNHG5	1
HECTD3	RHPN1-AS1	0.503828135	RP9	SNHG3	0.843524319	IQGAP1	ZNRD1-AS1	1
ERG	DLEU2	0.503748531	RPAP3	SNHG3	0.839133901	IQGAP2	HCP5	1
BTN3A3	KTN1-AS1	0.50373697	RPGRIP1L	HCP5	0.614710062	IQGAP2	KTN1-AS1	1
TMEM161B	ZNRD1-AS1	0.503426743	RPGRIP1L	TP53TG1	0.973843775	IQGAP2	MCM3AP-AS1	1
FKBP9	SNHG5	0.503388337	RPL13	SNHG3	0.991558918	IQGAP3	ZNRD1-AS1	1
FAM184A	H19	0.50321569	RPL13	SNHG5	0.740959626	IQSEC1	HCP5	1
SLC38A1	DLEU2	0.503079769	RPL22	HCP5	0.712847519	IQSEC1	MCM3AP-AS1	1
MEST	SNHG3	0.502985451	RPL22	KTN1-AS1	0.947520654	IQSEC1	TP53TG1	1
CIT	DLEU2	0.5029705	RPL22	MCM3AP-AS1	0.948460265	IRAK2	ZNRD1-AS1	1
PARD3B	SCARNA9	0.502239627	RPL23	SNHG5	0.752601724	IRF1	H19	1
GLIPR1	MCM3AP-AS1	0.502107679	RPL39L	H19	0.974337834	IRF1	SNHG5	1
FAM200A	MCM3AP-AS1	0.502073563	RPP25	MCM3AP-AS1	0.608883112	IRF1	ZNRD1-AS1	1
PRIM2	LINC00467	0.501931023	RPS23	LINC00467	0.959133552	IRF4	DLEU2	1
SMARCA1	SCARNA9	0.501863714	RPS6KA2	DLEU2	0.687590534	IRF4	H19	1
MYO5A	MCM3AP-AS1	0.501653776	RPS6KA4	HCP5	0.5407652	IRF4	SNHG5	1
ANKAR	RUSC1-AS1	0.501555724	RPS6KA4	ZNRD1-AS1	0.95608552	IRF4	TPT1-AS1	1
ZNF583	TP53TG1	0.501459879	RPS6KA5	H19	0.611749677	IRF4	ZNRD1-AS1	1
LCLAT1	MCM3AP-AS1	0.501404635	RPS6KA5	KTN1-AS1	0.91848458	IRF8	H19	1
CDK1	H19	0.501207132	RPS6KA5	LINC00467	0.662502099	IRF8	ZNRD1-AS1	1
CUTC	H19	0.501181412	RPS6KA5	MCM3AP-AS1	0.929899269	ISCA2	HCP5	1
CBLB	TP53TG1	0.501084327	RPS6KA5	ZNRD1-AS1	0.907650826	ISCA2	KTN1-AS1	1
ZFP14	SNHG5	0.501071732	RPS6KC1	DLEU2	0.627575884	ISCU	KTN1-AS1	1
PLSCR4	RUSC1-AS1	0.500959191	RPSA	SNHG3	0.928074541	ISCU	MCM3AP-AS1	1
PPAP2A	SCARNA9	0.50083701	RRAGD	HCP5	0.984078264	ISCU	SCARNA9	1
PTPN9	SNHG3	0.500774149	RRAGD	TP53TG1	0.695138442	ITGA1	ZNRD1-AS1	1
FAM117B	SNHG5	0.500693633	RRN3	SNHG5	0.542255524	ITGA2	HCP5	1
CDADC1	H19	0.500563271	RSBN1	H19	0.617515057	ITGA2	MCM3AP-AS1	1
RCAN3	MCM3AP-AS1	0.500405633	RSBN1	HCP5	0.602529846	ITGA2	SNHG3	1
MSRB2	MCM3AP-AS1	0.500385086	RTKN	H19	0.561224537	ITGA2	TP53TG1	1
RELL1	SNHG3	0.50025348	RTKN	ZNRD1-AS1	0.933805142	ITGA4	HCP5	1
AMOT	SNHG5	0.500162393	RTKN2	H19	0.774676719	ITGA4	SCARNA9	1
SEPP1	HCP5	0.500132051	RTKN2	HCP5	0.707138418	ITGA6	HCP5	1
			RTKN2	KTN1-AS1	0.566472898	ITGA6	MCM3AP-AS1	1
			RUNX1T1	SCARNA9	0.975495215	ITGA6	SNHG3	1
			RUNX1T1	SNHG5	0.827115293	ITGAV	H19	1
			RUNX2	H19	0.755306209	ITGAV	SNHG5	1
			RUNX2	MCM3AP-AS1	0.574332075	ITGAV	ZNRD1-AS1	1

RUNX2	TPT1-AS1	0.631948324	ITGB5	HCP5	1
RUNX2	ZNRD1-AS1	0.532113494	ITGB8	H19	1
RUNX3	RUSC1-AS1	0.676639361	ITGB8	LINC00467	1
RUNX3	ZNRD1-AS1	0.915715976	ITGB8	RUSC1-AS1	1
RWDD2B	H19	0.765838838	ITGB8	SNHG5	1
RWDD2B	HCP5	0.736123141	ITGB8	ZNRD1-AS1	1
S1PR1	SNHG3	0.724072405	ITM2B	DLEU2	1
S1PR1	SNHG5	0.960938269	ITM2B	H19	1
SAAL1	SNHG3	0.660999491	ITM2B	LINC00467	1
SAMD10	H19	0.536909567	ITM2B	RUSC1-AS1	1
SAMD10	MCM3AP-AS1	0.995266552	ITM2B	SNHG5	1
SAMD10	ZNRD1-AS1	0.954855395	ITM2B	TPT1-AS1	1
SAMD12	HCP5	0.97192252	ITM2B	ZNRD1-AS1	1
SAMD4A	MCM3AP-AS1	0.741109069	ITPR1	HCP5	1
SAMD4A	TPT1-AS1	0.96105892	ITPR1	MCM3AP-AS1	1
SAMD4A	ZNRD1-AS1	0.818156653	ITPR1	SCARNA9	1
SAMD4B	RUSC1-AS1	0.655012137	ITPR1	SNHG3	1
SAMD8	SNHG3	0.578382045	ITPR1	TP53TG1	1
SAMD8	SNHG5	0.605770141	ITPR3	DLEU2	1
SAMSN1	SNHG5	0.63222685	ITPR3	TPT1-AS1	1
SAP30BP	H19	0.949845008	ITPRIP	H19	1
SAP30L	HCP5	0.900889902	ITPRIP	SNHG5	1
SASS6	H19	0.762224422	ITPRIP	ZNRD1-AS1	1
SASS6	HCP5	0.590874102	IVNS1ABP	H19	1
SBF2	H19	0.605455138	IVNS1ABP	RUSC1-AS1	1
SBF2	KTN1-AS1	0.723073173	IVNS1ABP	ZNRD1-AS1	1
SBF2	MCM3AP-AS1	0.745469574	JAG2	DLEU2	1
SBF2	ZNRD1-AS1	0.743442853	JAG2	H19	1
SCAI	HCP5	0.922707566	JAG2	TPT1-AS1	1
SCAMP5	HCP5	0.99305161	JAG2	ZNRD1-AS1	1
SCAPER	H19	0.636309332	JARID2	DLEU2	1
SCAPER	LINC00467	0.599191511	JARID2	H19	1
SCAPER	MCM3AP-AS1	0.871404279	JARID2	RUSC1-AS1	1
SCAPER	ZNRD1-AS1	0.852108371	JARID2	SNHG5	1
SCARB1	MCM3AP-AS1	0.796187725	JARID2	TPT1-AS1	1
SCARB1	ZNRD1-AS1	0.660781027	JARID2	ZNRD1-AS1	1
SCARB2	RHPN1-AS1	0.899268838	JMJD8	HCP5	1
SCARB2	SNHG3	0.852348084	JMJD8	MCM3AP-AS1	1
SCG5	HCP5	0.690533391	JMJD8	RHPN1-AS1	1
SCN9A	SNHG5	0.689240463	JMJD8	SNHG3	1
SDC4	SNHG3	0.882211501	JUNB	MCM3AP-AS1	1
SDCCAG3	MCM3AP-AS1	0.562899422	JUP	DLEU2	1
SEC24A	DLEU2	0.582407741	JUP	TPT1-AS1	1
SEC24A	SNHG3	0.691131457	JUP	ZNRD1-AS1	1
SEC24A	SNHG5	0.899147013	KAL1	HCP5	1
SEC24D	SNHG3	0.982138814	KAL1	KTN1-AS1	1
SEC61A2	MCM3AP-AS1	0.891764602	KAL1	MCM3AP-AS1	1
SEC61A2	TPT1-AS1	0.94823916	KAL1	SCARNA9	1
SEC61A2	ZNRD1-AS1	0.906504186	KANK1	MCM3AP-AS1	1
SEH1L	HCP5	0.876538344	KANK1	SNHG3	1
SEH1L	LINC00467	0.715444491	KAT2B	HCP5	1

SEMA3A	SNHG3	0.846594536	KAT2B	MCM3AP-AS1	1
SEMA3A	SNHG5	0.67552562	KAT2B	SCARNA9	1
SEMA4D	ZNRD1-AS1	0.598651865	KAT2B	TP53TG1	1
SEMA6A	DLEU2	0.628901526	KATNAL1	H19	1
SEMA6A	SNHG3	0.529270695	KATNAL1	ZNRD1-AS1	1
SEMA6D	DLEU2	0.782881797	KATNB1	MCM3AP-AS1	1
SEMA6D	RUSC1-AS1	0.641389287	KBTBD6	KTN1-AS1	1
SEMA6D	SNHG5	0.767938081	KBTBD6	SCARNA9	1
SENP5	SNHG5	0.669562141	KBTBD6	SNHG3	1
SEPN1	H19	0.813189982	KBTBD8	HCP5	1
SEPN1	HCP5	0.61009137	KBTBD8	KTN1-AS1	1
SEPN1	LINC00467	0.98668187	KBTBD8	MCM3AP-AS1	1
10-Sep	SNHG5	0.648625563	KBTBD8	SNHG3	1
6-Sep	H19	0.568772884	KCNA3	H19	1
6-Sep	ZNRD1-AS1	0.943145741	KCNA3	ZNRD1-AS1	1
8-Sep	SNHG3	0.936217211	KCNK2	HCP5	1
8-Sep	SNHG5	0.769157694	KCNK5	KTN1-AS1	1
SERP1	HCP5	0.980756235	KCNK5	MCM3AP-AS1	1
SERPINB8	H19	0.56087338	KCNK5	SCARNA9	1
SERPINB8	ZNRD1-AS1	0.585670169	KCNMB1	H19	1
SESN1	H19	0.57657614	KCNN4	H19	1
SESN1	HCP5	0.79608079	KCNN4	ZNRD1-AS1	1
SESN1	MCM3AP-AS1	0.863359502	KCTD10	H19	1
SESN1	ZNRD1-AS1	0.760634577	KCTD10	ZNRD1-AS1	1
SESN2	H19	0.768684491	KCTD12	HCP5	1
SESN2	KTN1-AS1	0.869033434	KCTD12	MCM3AP-AS1	1
SESN2	MCM3AP-AS1	0.848064947	KCTD12	SNHG3	1
SESN2	ZNRD1-AS1	0.790895966	KCTD12	TP53TG1	1
SESN3	H19	0.570069145	KCTD14	H19	1
SESN3	HCP5	0.884101108	KCTD14	RUSC1-AS1	1
SESN3	ZNRD1-AS1	0.580642676	KCTD15	HCP5	1
SFMBT1	SNHG5	0.660883856	KCTD15	SNHG3	1
SFT2D1	SNHG5	0.631678908	KCTD20	H19	1
SFXN1	SNHG3	0.996119109	KCTD20	SNHG5	1
SFXN1	SNHG5	0.72679299	KCTD20	ZNRD1-AS1	1
SFXN2	HCP5	0.814684493	KCTD3	HCP5	1
SFXN2	MCM3AP-AS1	0.865628805	KCTD3	KTN1-AS1	1
SFXN2	TP53TG1	0.724972828	KCTD3	MCM3AP-AS1	1
SFXN2	ZNRD1-AS1	0.77046634	KCTD9	DLEU2	1
SFXN3	H19	0.58484587	KCTD9	SNHG5	1
SFXN3	HCP5	0.809607138	KCTD9	TPT1-AS1	1
SFXN4	HCP5	0.556074678	KDELC1	DLEU2	1
SGCB	SNHG3	0.947204491	KDELC1	H19	1
SGK1	SNHG5	0.64413713	KDELC1	LINC00467	1
SGK3	DLEU2	0.719166288	KDELC1	SNHG5	1
SGK3	SNHG5	0.967920541	KDELC2	HCP5	1
SGMS1	SNHG3	0.992964134	KDELC2	MCM3AP-AS1	1
SGMS1	SNHG5	0.757910804	KDELC2	SNHG3	1
SGMS2	TP53TG1	0.735350929	KDM2B	DLEU2	1
SGOL1	H19	0.657354166	KDM2B	H19	1
SGPL1	SNHG3	0.940287008	KDM2B	TPT1-AS1	1

SH3BP4	SNHG5	0.913683306	KDM6B	DLEU2	1
SH3D19	HCP5	0.563232268	KDM6B	H19	1
SH3RF3	SNHG3	0.855152352	KDM6B	TPT1-AS1	1
SHB	KTN1-AS1	0.631427316	KDM6B	ZNRD1-AS1	1
SHB	MCM3AP-AS1	0.572257312	KIAA0101	MCM3AP-AS1	1
SHCBP1	H19	0.502224156	KIAA0226	DLEU2	1
SHCBP1	TP53TG1	0.730805632	KIAA0226	H19	1
SHISA5	HCP5	0.530501907	KIAA0226	LINC00467	1
SHISA5	SNHG5	0.626580554	KIAA0226	TPT1-AS1	1
SHMT2	H19	0.828607995	KIAA0226	ZNRD1-AS1	1
SIDT1	MCM3AP-AS1	0.823170919	KIAA0430	DLEU2	1
SIX4	SNHG3	0.947804674	KIAA0430	H19	1
SKA1	H19	0.725152265	KIAA0430	RUSC1-AS1	1
SKA2	H19	0.566075182	KIAA0430	SNHG5	1
SKA2	KTN1-AS1	0.791488481	KIAA0430	TPT1-AS1	1
SKA2	ZNRD1-AS1	0.569163868	KIAA0430	ZNRD1-AS1	1
SKA3	HCP5	0.803568182	KIAA0895	HCP5	1
SKAP2	MCM3AP-AS1	0.67838757	KIAA0895	MCM3AP-AS1	1
SKAP2	ZNRD1-AS1	0.515503535	KIAA0895	SNHG3	1
SKIL	SNHG5	0.744422025	KIAA0922	HCP5	1
SLA	LINC00467	0.844831583	KIAA0922	KTN1-AS1	1
SLAIN1	H19	0.749635092	KIAA0922	MCM3AP-AS1	1
SLAIN1	HCP5	0.758965638	KIAA0922	SNHG3	1
SLAIN1	MCM3AP-AS1	0.702169004	KIAA1109	RUSC1-AS1	1
SLAIN1	TP53TG1	0.927069913	KIAA1109	SNHG5	1
SLAIN1	ZNRD1-AS1	0.547952577	KIAA1147	HCP5	1
SLAMF7	SNHG3	0.827942311	KIAA1147	SCARNA9	1
SLAMF7	SNHG5	0.812701056	KIAA1161	MCM3AP-AS1	1
SLBP	SNHG5	0.600489829	KIAA1161	SCARNA9	1
SLC11A2	SNHG5	0.579075207	KIAA1161	SNHG3	1
SLC16A1	HCP5	0.780638655	KIAA1211	MCM3AP-AS1	1
SLC16A1	MCM3AP-AS1	0.817877453	KIAA1244	MCM3AP-AS1	1
SLC16A1	TP53TG1	0.834612294	KIAA1244	SNHG3	1
SLC16A1	ZNRD1-AS1	0.693926375	KIAA1377	SNHG3	1
SLC16A10	SCARNA9	0.607651372	KIAA1462	H19	1
SLC16A9	HCP5	0.898735113	KIAA1462	RUSC1-AS1	1
SLC16A9	TP53TG1	0.765617433	KIAA1462	ZNRD1-AS1	1
SLC1A4	SNHG5	0.510041362	KIAA1524	SCARNA9	1
SLC20A2	SNHG3	0.68026878	KIAA1524	SNHG3	1
SLC24A1	MCM3AP-AS1	0.972260537	KIAA1524	TP53TG1	1
SLC24A1	ZNRD1-AS1	0.90473453	KIAA1598	HCP5	1
SLC25A15	RHPN1-AS1	0.777536311	KIAA1598	MCM3AP-AS1	1
SLC25A24	SNHG3	0.957569974	KIAA2022	MCM3AP-AS1	1
SLC25A24	SNHG5	0.530851781	KIF11	HCP5	1
SLC25A25	SNHG5	0.563297492	KIF11	KTN1-AS1	1
SLC27A3	ZNRD1-AS1	0.752581843	KIF13A	H19	1
SLC29A1	MCM3AP-AS1	0.549948091	KIF13A	ZNRD1-AS1	1
SLC2A13	H19	0.509199329	KIF13B	H19	1
SLC2A4RG	H19	0.974616356	KIF13B	ZNRD1-AS1	1
SLC30A7	SNHG5	0.931765274	KIF15	HCP5	1
SLC30A9	TP53TG1	0.614376928	KIF15	SNHG3	1

SLC31A1	H19	0.603172182	KIF18A	SNHG3	1
SLC31A1	HCP5	0.556844226	KIF18B	MCM3AP-AS1	1
SLC31A1	LINC00467	0.625736881	KIF20A	KTN1-AS1	1
SLC35D2	SNHG3	0.60726441	KIF20A	SCARNA9	1
SLC35E1	SNHG3	0.86470356	KIF20B	SNHG3	1
SLC35F1	DLEU2	0.690488885	KIF21A	HCP5	1
SLC35F1	TPT1-AS1	0.674429805	KIF21A	KTN1-AS1	1
SLC35F1	ZNRD1-AS1	0.746149904	KIF21A	MCM3AP-AS1	1
SLC38A1	H19	0.579454094	KIF22	MCM3AP-AS1	1
SLC39A14	SNHG3	0.564878963	KIF23	HCP5	1
SLC39A14	TP53TG1	0.592668258	KIF23	MCM3AP-AS1	1
SLC40A1	HCP5	0.986736345	KIF23	SNHG3	1
SLC41A2	DLEU2	0.79022828	KIF23	TP53TG1	1
SLC44A5	SNHG3	0.914198768	KIF24	MCM3AP-AS1	1
SLC45A4	SNHG5	0.964495415	KIF24	RHPN1-AS1	1
SLC46A1	KTN1-AS1	0.913850976	KIF2A	ZNRD1-AS1	1
SLC46A1	MCM3AP-AS1	0.893183182	KIF2C	MCM3AP-AS1	1
SLC46A1	ZNRD1-AS1	0.8341282	KIF3A	HCP5	1
SLC48A1	H19	0.760471547	KIF3A	SNHG3	1
SLC48A1	HCP5	0.755651141	KIF3B	H19	1
SLC48A1	LINC00467	0.991574732	KIF3B	LINC00467	1
SLC48A1	ZNRD1-AS1	0.505585732	KIF3B	SNHG5	1
SLC4A7	H19	0.737058393	KIF3B	ZNRD1-AS1	1
SLC4A7	HCP5	0.632515782	KIF5B	DLEU2	1
SLC5A3	SNHG3	0.663703305	KIF5B	H19	1
SLC6A9	H19	0.677042989	KIF5B	SNHG5	1
SLC6A9	HCP5	0.832444299	KIF5B	TPT1-AS1	1
SLC9A8	TPT1-AS1	0.627007435	KIF5B	ZNRD1-AS1	1
SLCO5A1	ZNRD1-AS1	0.670177965	KIF5C	H19	1
SLTM	TPT1-AS1	0.795347957	KIF5C	SNHG5	1
SLTM	ZNRD1-AS1	0.534188834	KIF5C	ZNRD1-AS1	1
SMAD1	H19	0.705955471	KIRREL	HCP5	1
SMAD3	SNHG3	0.763588097	KLC2	ZNRD1-AS1	1
SMAD5	HCP5	0.944819415	KLF11	HCP5	1
SMAD6	SNHG3	0.798618278	KLF11	KTN1-AS1	1
SMAD6	SNHG5	0.937266617	KLF11	MCM3AP-AS1	1
SMAD7	SNHG3	0.674634218	KLF11	SCARNA9	1
SMAD7	SNHG5	0.778071095	KLF12	HCP5	1
SMARCA1	HCP5	0.713776094	KLF12	MCM3AP-AS1	1
SMARCA1	KTN1-AS1	0.921732756	KLF12	SCARNA9	1
SMARCA1	MCM3AP-AS1	0.935010938	KLF12	TP53TG1	1
SMARCA1	ZNRD1-AS1	0.914359101	KLF3	H19	1
SMARCA2	H19	0.54255416	KLF3	LINC00467	1
SMARCA2	LINC00467	0.775985911	KLF3	RUSC1-AS1	1
SMARCA2	ZNRD1-AS1	0.961361823	KLF3	SNHG5	1
SMC2	MCM3AP-AS1	0.668638246	KLF3	ZNRD1-AS1	1
SMC2	ZNRD1-AS1	0.506212184	KLF4	HCP5	1
SMG5	ZNRD1-AS1	0.643035379	KLF4	KTN1-AS1	1
SMYD2	H19	0.556031967	KLF4	MCM3AP-AS1	1
SNAPC1	SNHG5	0.799825759	KLF4	RHPN1-AS1	1
SNAPC4	RUSC1-AS1	0.58073792	KLF4	SNHG3	1

SNRNP40	SNHG3	0.625945366	KLF5	HCP5	1
SNRPF	SNHG3	0.744168669	KLF5	KTN1-AS1	1
SNTB1	ZNRD1-AS1	0.758395136	KLF5	MCM3AP-AS1	1
SNX25	SNHG3	0.896177198	KLF5	SNHG3	1
SNX25	SNHG5	0.819448262	KLF6	H19	1
SNX29	TPT1-AS1	0.831568961	KLF6	LINC00467	1
SNX4	SNHG3	0.921528971	KLF6	RUSC1-AS1	1
SNX7	HCP5	0.707368499	KLF6	SNHG5	1
SNX9	SNHG3	0.701486613	KLF6	ZNRD1-AS1	1
SOBP	DLEU2	0.777570696	KLF7	H19	1
SOBP	SNHG5	0.876926756	KLF7	ZNRD1-AS1	1
SOCS1	DLEU2	0.663283497	KLHDC8B	MCM3AP-AS1	1
SOCS5	HCP5	0.812080163	KLHL2	HCP5	1
SOCS5	LINC00467	0.592169137	KLHL2	KTN1-AS1	1
SOCS5	TP53TG1	0.712820561	KLHL2	MCM3AP-AS1	1
SORBS1	SNHG5	0.996207302	KLHL2	SNHG3	1
SORD	H19	0.620875111	KLHL2	TP53TG1	1
SORL1	HCP5	0.862310514	KLHL23	SCARNA9	1
SORL1	ZNRD1-AS1	0.785381147	KLHL23	SNHG3	1
SP1	H19	0.863791819	KLHL3	SNHG3	1
SP1	HCP5	0.518713131	KLHL36	HCP5	1
SP1	KTN1-AS1	0.894474652	KPNA2	H19	1
SP1	MCM3AP-AS1	0.806628625	KPNA2	SNHG5	1
SP1	TP53TG1	0.875889172	KPNA2	ZNRD1-AS1	1
SP1	ZNRD1-AS1	0.672065941	KSR1	DLEU2	1
SPAG7	SNHG5	0.536896803	KSR1	TPT1-AS1	1
SPAG9	RUSC1-AS1	0.597505118	L2HGDH	MCM3AP-AS1	1
SPAG9	TPT1-AS1	0.820032358	LACTB	DLEU2	1
SPATS2	SNHG5	0.841449089	LACTB	TPT1-AS1	1
SPG21	SCARNA9	0.602275734	LAMA2	HCP5	1
SPIN4	TP53TG1	0.583704452	LAMA3	HCP5	1
SPIRE1	H19	0.797014692	LAMC1	HCP5	1
SPIRE1	HCP5	0.657809731	LAMC1	MCM3AP-AS1	1
SPIRE1	MCM3AP-AS1	0.796031755	LAMC1	SCARNA9	1
SPIRE1	ZNRD1-AS1	0.656682193	LAMC1	SNHG3	1
SPOCK3	MCM3AP-AS1	0.606911875	LAMC1	TP53TG1	1
SPOCK3	ZNRD1-AS1	0.530991891	LAMP3	H19	1
SPPL2B	H19	0.531045905	LAMP3	LINC00467	1
SPRED1	SNHG3	0.983614255	LAMP3	ZNRD1-AS1	1
SPRED1	SNHG5	0.737017683	LAMTOR1	HCP5	1
SPRYD3	SNHG3	0.573108426	LANCL1	HCP5	1
SPSB4	HCP5	0.758177353	LANCL1	KTN1-AS1	1
SPTLC2	H19	0.80134176	LANCL1	MCM3AP-AS1	1
SPTLC2	HCP5	0.570900872	LANCL1	SNHG3	1
SPTLC2	MCM3AP-AS1	0.859883518	LAT2	SNHG3	1
SPTLC2	TPT1-AS1	0.538062118	LCLAT1	HCP5	1
SPTLC2	ZNRD1-AS1	0.738528629	LCLAT1	MCM3AP-AS1	1
SPTY2D1	RHPN1-AS1	0.980643735	LDLRAD3	H19	1
SPTY2D1	SNHG3	0.962914891	LDLRAD3	ZNRD1-AS1	1
SRF	SNHG5	0.537294309	LETMD1	ZNRD1-AS1	1
SRGAP1	SNHG5	0.874255109	LFNG	MCM3AP-AS1	1

SRP19	TP53TG1	0.632394352	LGMN	ZNRD1-AS1	1
SRRT	ZNRD1-AS1	0.843430834	LHFPL2	HCP5	1
SRSF1	HCP5	0.634781505	LHFPL2	KTN1-AS1	1
SRSF12	H19	0.675436974	LHFPL2	MCM3AP-AS1	1
SRSF12	HCP5	0.819403044	LHFPL2	SNHG3	1
SRSF12	ZNRD1-AS1	0.568497366	LHX6	H19	1
SRSF5	SNHG5	0.820014682	LHX6	ZNRD1-AS1	1
SSBP2	KTN1-AS1	0.991978938	LIF	HCP5	1
SSBP2	TPT1-AS1	0.699064722	LILRB1	SCARNA9	1
SSU72	RHPN1-AS1	0.833590101	LIMA1	H19	1
ST3GAL4	SNHG3	0.910793836	LIMA1	LINC00467	1
ST8SIA4	H19	0.708131309	LIMA1	ZNRD1-AS1	1
ST8SIA4	HCP5	0.546148335	LIMCH1	MCM3AP-AS1	1
STC2	H19	0.878019616	LIMCH1	SNHG3	1
STC2	MCM3AP-AS1	0.741078377	LIMCH1	TP53TG1	1
STC2	ZNRD1-AS1	0.652815702	LIMK2	ZNRD1-AS1	1
STEAP2	HCP5	0.503040819	LIMS3	HCP5	1
STEAP2	SNHG5	0.726935505	LIN9	HCP5	1
STK17A	H19	0.689211676	LIN9	KTN1-AS1	1
STK17A	HCP5	0.814206358	LIN9	MCM3AP-AS1	1
STK17A	LINC00467	0.941115876	LIN9	SCARNA9	1
STK17A	MCM3AP-AS1	0.510731068	LIN9	SNHG3	1
STK17B	SNHG3	0.802440446	LIN9	TP53TG1	1
STK24	SNHG3	0.90537859	LIPT1	HCP5	1
STK32B	H19	0.659142726	LIPT2	HCP5	1
STK32B	HCP5	0.82768461	LIPT2	KTN1-AS1	1
STK32B	MCM3AP-AS1	0.725220042	LIX1L	HCP5	1
STK32B	ZNRD1-AS1	0.583984606	LIX1L	SNHG3	1
STK38L	H19	0.628227924	LLGL1	SNHG3	1
STK38L	KTN1-AS1	0.877196048	LMBR1L	DLEU2	1
STK38L	MCM3AP-AS1	0.889558968	LMBR1L	SNHG5	1
STK38L	TPT1-AS1	0.882011455	LMBR1L	TPT1-AS1	1
STK38L	ZNRD1-AS1	0.870034604	LMLN	KTN1-AS1	1
STK39	SNHG3	0.614904016	LMLN	MCM3AP-AS1	1
STK39	SNHG5	0.512340662	LMNB1	HCP5	1
STMN1	HCP5	0.902209833	LMNB1	KTN1-AS1	1
STOM	H19	0.774373822	LMNB1	MCM3AP-AS1	1
STOM	HCP5	0.669414951	LMO4	H19	1
STOM	MCM3AP-AS1	0.817709325	LMO4	LINC00467	1
STOM	ZNRD1-AS1	0.684348191	LMO4	SNHG5	1
STOX1	HCP5	0.956994922	LMO4	ZNRD1-AS1	1
STRADB	HCP5	0.973316007	LONRF2	MCM3AP-AS1	1
STRADB	MCM3AP-AS1	0.563616408	LOX	DLEU2	1
STRAP	SNHG3	0.900854071	LOX	SNHG5	1
STX11	SNHG5	0.754421098	LOX	TPT1-AS1	1
STX3	H19	0.662179974	LOXL1	MCM3AP-AS1	1
STX3	LINC00467	0.894243869	LPAR1	DLEU2	1
STX3	ZNRD1-AS1	0.885928856	LPAR1	SNHG5	1
SUB1	SNHG3	0.537415169	LPCAT4	HCP5	1
SUFU	H19	0.801399548	LPCAT4	SNHG3	1
SUFU	HCP5	0.506012811	LPL	HCP5	1

SUFU	LINC00467	0.943419872	LPP	DLEU2	1
SUFU	ZNRD1-AS1	0.774977693	LPP	LINC00467	1
SULF2	H19	0.761162282	LPP	RUSC1-AS1	1
SULF2	KTN1-AS1	0.578470935	LPP	SNHG5	1
SULF2	MCM3AP-AS1	0.558780486	LPP	TPT1-AS1	1
SUMO1	H19	0.931238684	LPP	ZNRD1-AS1	1
SUMO1	LINC00467	0.580151395	LRCH2	DLEU2	1
SUN2	H19	0.724641863	LRCH2	H19	1
SUN2	MCM3AP-AS1	0.808149145	LRCH2	RUSC1-AS1	1
SUN2	ZNRD1-AS1	0.769728152	LRCH2	TPT1-AS1	1
SUSD1	SNHG5	0.975604057	LRCH2	ZNRD1-AS1	1
SUV39H1	H19	0.707663312	LRIG1	H19	1
SUV39H2	H19	0.588106126	LRIG1	RUSC1-AS1	1
SUV39H2	HCP5	0.790571168	LRIG1	SNHG5	1
SUV39H2	LINC00467	0.804007583	LRIG1	ZNRD1-AS1	1
SVIL	ZNRD1-AS1	0.529029851	LRMP	HCP5	1
SVIP	SNHG5	0.874121093	LRP1	HCP5	1
SYAP1	SNHG3	0.894103072	LRP1	SNHG3	1
SYBU	HCP5	0.929399609	LRP12	H19	1
SYDE2	SNHG3	0.535085819	LRP12	ZNRD1-AS1	1
SYDE2	SNHG5	0.847147411	LRP2BP	TP53TG1	1
SYNGR1	MCM3AP-AS1	0.613209218	LRP5	HCP5	1
SYPL1	DLEU2	0.94760254	LRP5	KTN1-AS1	1
SYPL1	SCARNA9	0.882757916	LRP5	MCM3AP-AS1	1
SYPL1	SNHG5	0.724060758	LRP5	SCARNA9	1
SYT1	H19	0.888812391	LRRC16A	H19	1
TAB2	KTN1-AS1	0.912479875	LRRC16A	SNHG5	1
TAB2	MCM3AP-AS1	0.968615667	LRRC16A	ZNRD1-AS1	1
TAB2	TPT1-AS1	0.929008222	LRRC61	H19	1
TAB3	SNHG3	0.794847153	LRRC61	RUSC1-AS1	1
TACC1	DLEU2	0.527145032	LRRFIP1	DLEU2	1
TACC1	SNHG5	0.780924636	LRRFIP1	H19	1
TAF13	SNHG3	0.785666565	LRRFIP1	LINC00467	1
TAF13	SNHG5	0.686341577	LRRFIP1	SNHG5	1
TAGAP	SNHG5	0.749931409	LRRK2	TP53TG1	1
TANK	SNHG3	0.628345623	LSM11	MCM3AP-AS1	1
TARBP1	MCM3AP-AS1	0.977943355	LSM4	HCP5	1
TARBP1	ZNRD1-AS1	0.992191828	LSM5	HCP5	1
TARS	SNHG5	0.651218056	LSM5	MCM3AP-AS1	1
TBC1D12	SNHG3	0.648444613	LTA	MCM3AP-AS1	1
TBC1D12	TP53TG1	0.551810997	LTA4H	ZNRD1-AS1	1
TBC1D14	MCM3AP-AS1	0.905493109	LTBP3	MCM3AP-AS1	1
TBC1D14	TP53TG1	0.675092018	LTV1	HCP5	1
TBC1D14	ZNRD1-AS1	0.824234797	LTV1	KTN1-AS1	1
TBC1D17	ZNRD1-AS1	0.908228503	LURAP1L	MCM3AP-AS1	1
TBC1D2	HCP5	0.937427843	LXN	HCP5	1
TBC1D2	ZNRD1-AS1	0.606664491	LY6E	MCM3AP-AS1	1
TBC1D2B	TPT1-AS1	0.640579052	LY75	H19	1
TBC1D4	KTN1-AS1	0.936387316	LY75	ZNRD1-AS1	1
TBC1D4	MCM3AP-AS1	0.862337293	LYAR	KTN1-AS1	1
TBC1D4	ZNRD1-AS1	0.741782308	LYAR	MCM3AP-AS1	1

TBC1D9	SNHG5	0.589975125	LYRM1	HCP5	1
TBCEL	HCP5	0.915394934	LYRM1	RHPN1-AS1	1
TBCEL	LINC00467	0.803926194	LYRM1	SCARNA9	1
TBX19	H19	0.5020705	LYRM5	MCM3AP-AS1	1
TBX19	HCP5	0.862343746	LYST	DLEU2	1
TBX19	ZNRD1-AS1	0.717442384	LYST	H19	1
TCEA2	H19	0.790342026	LYST	SNHG5	1
TCEA2	LINC00467	0.96243638	LYST	ZNRD1-AS1	1
TCEA2	ZNRD1-AS1	0.75956822	MACC1	SCARNA9	1
TCERG1	RUSC1-AS1	0.6564117	MAD2L1	HCP5	1
TCERG1	ZNRD1-AS1	0.532426987	MAD2L1	KTN1-AS1	1
TCF4	HCP5	0.707403292	MAD2L1	MCM3AP-AS1	1
TCF4	MCM3AP-AS1	0.952077736	MAD2L1	SNHG3	1
TCF4	TP53TG1	0.596216874	MAF	DLEU2	1
TCF4	TPT1-AS1	0.684093087	MAF	H19	1
TCF4	ZNRD1-AS1	0.892955554	MAF	TPT1-AS1	1
TCF7L2	KTN1-AS1	0.754230014	MAF	ZNRD1-AS1	1
TCF7L2	MCM3AP-AS1	0.833259099	MAGT1	HCP5	1
TCF7L2	TPT1-AS1	0.987148633	MAGT1	MCM3AP-AS1	1
TCF7L2	ZNRD1-AS1	0.894411707	MAGT1	SNHG3	1
TCP11L2	KTN1-AS1	0.967534051	MAK16	HCP5	1
TCP11L2	TPT1-AS1	0.86672872	MAK16	KTN1-AS1	1
TCTEX1D2	MCM3AP-AS1	0.536492037	MAMDC2	DLEU2	1
TCTN1	MCM3AP-AS1	0.566486215	MAMDC2	H19	1
TDG	SNHG3	0.863794112	MAMDC2	LINC00467	1
TDRD7	SNHG5	0.702877092	MAMDC2	SNHG5	1
TEAD4	H19	0.826051859	MAMDC2	TPT1-AS1	1
TET1	H19	0.600437104	MAMDC2	ZNRD1-AS1	1
TET1	HCP5	0.856341081	MAMLD1	MCM3AP-AS1	1
TET1	ZNRD1-AS1	0.618302172	MAN1A1	DLEU2	1
TET3	KTN1-AS1	0.95787832	MAN1A1	RUSC1-AS1	1
TET3	MCM3AP-AS1	0.985741766	MAN1A1	TPT1-AS1	1
TET3	TPT1-AS1	0.924866017	MAN1B1	RUSC1-AS1	1
TET3	ZNRD1-AS1	0.980594388	MAN1C1	HCP5	1
TEX2	SNHG3	0.680328046	MANBA	KTN1-AS1	1
TEX2	SNHG5	0.721799572	MANF	H19	1
TEX30	SNHG5	0.716411262	MANF	ZNRD1-AS1	1
TFB1M	H19	0.817150277	MAP2K1	MCM3AP-AS1	1
TFB2M	SNHG3	0.737622856	MAP2K3	HCP5	1
TFDP1	H19	0.694748959	MAP2K3	MCM3AP-AS1	1
TFPI	HCP5	0.9606178	MAP3K13	HCP5	1
TFPI	MCM3AP-AS1	0.689672116	MAP3K13	MCM3AP-AS1	1
TFPI	TP53TG1	0.58256951	MAP3K13	SNHG3	1
TFPI	ZNRD1-AS1	0.61310954	MAP3K14	H19	1
TFPI2	DLEU2	0.581334633	MAP3K14	ZNRD1-AS1	1
TFPI2	SNHG5	0.66548592	MAP3K3	HCP5	1
TFR2	MCM3AP-AS1	0.803690645	MAP3K3	KTN1-AS1	1
TFRC	RHPN1-AS1	0.6809624	MAP3K3	MCM3AP-AS1	1
TFRC	SNHG3	0.725434807	MAP3K3	SNHG3	1
TGFBR2	SNHG3	0.874158034	MAP3K3	TP53TG1	1
TGM2	RHPN1-AS1	0.846473365	MAP3K5	HCP5	1

THAP11	H19	0.87732966	MAP3K5	KTN1-AS1	1
THAP2	H19	0.666747202	MAP3K5	MCM3AP-AS1	1
THBS1	SNHG3	0.813286889	MAP3K5	SNHG3	1
THEM4	HCP5	0.993176775	MAP3K8	H19	1
THEM4	KTN1-AS1	0.556513385	MAP3K8	SNHG5	1
THOP1	H19	0.874836669	MAP3K8	ZNRD1-AS1	1
THRA	H19	0.622296535	MAP4	H19	1
THRA	HCP5	0.663568388	MAP4	ZNRD1-AS1	1
THRA	ZNRD1-AS1	0.850938995	MAP4K1	MCM3AP-AS1	1
TIAM1	H19	0.667551145	MAP4K4	DLEU2	1
TIMM50	H19	0.872849594	MAP4K4	H19	1
TIMM50	LINC00467	0.959401829	MAP4K4	TPT1-AS1	1
TIMM50	ZNRD1-AS1	0.6737604	MAP4K4	ZNRD1-AS1	1
TIMP2	TPT1-AS1	0.830647693	MAP6D1	RHPN1-AS1	1
TIMP2	ZNRD1-AS1	0.630463396	MAP7	HCP5	1
TIMP3	HCP5	0.91833837	MAP7	KTN1-AS1	1
TJP2	SNHG5	0.818997696	MAP7	MCM3AP-AS1	1
TK1	H19	0.662005524	MAP7	SNHG3	1
TK1	LINC00467	0.856996911	MAP7D2	H19	1
TLE1	SNHG3	0.789336114	MAP7D2	ZNRD1-AS1	1
TLE4	H19	0.541001583	MAPKAP1	DLEU2	1
TLN1	RUSC1-AS1	0.78357599	MAPKAP1	H19	1
TM7SF3	H19	0.742268481	MAPKAP1	RUSC1-AS1	1
TMC7	HCP5	0.622333675	MAPKAP1	TPT1-AS1	1
TMCO4	TPT1-AS1	0.595362035	MAPKAPK3	HCP5	1
TMEFF1	HCP5	0.808545381	MAPKAPK3	MCM3AP-AS1	1
TMEM109	KTN1-AS1	0.524354756	MAPKBP1	H19	1
TMEM109	LINC00467	0.881924396	MAPKBP1	SNHG5	1
TMEM110	RHPN1-AS1	0.506687221	MARCKS	DLEU2	1
TMEM134	HCP5	0.917128798	MARCKS	H19	1
TMEM134	ZNRD1-AS1	0.708907306	MARCKS	TPT1-AS1	1
TMEM136	SNHG5	0.688639894	MARCKS	ZNRD1-AS1	1
TMEM141	H19	0.735187603	MARS	HCP5	1
TMEM141	HCP5	0.782457512	MARVELD1	HCP5	1
TMEM167A	HCP5	0.881395359	MAST3	DLEU2	1
TMEM180	H19	0.665702316	MAST3	H19	1
TMEM187	SNHG3	0.93811137	MAST3	SNHG5	1
TMEM19	HCP5	0.911877317	MAST3	TPT1-AS1	1
TMEM19	TP53TG1	0.818264535	MAST3	ZNRD1-AS1	1
TMEM194A	H19	0.546854116	MAST4	DLEU2	1
TMEM194A	HCP5	0.851253916	MAST4	H19	1
TMEM2	SCARNA9	0.537281648	MAST4	SNHG5	1
TMEM2	SNHG3	0.732111921	MAST4	TPT1-AS1	1
TMEM2	SNHG5	0.978014089	MAST4	ZNRD1-AS1	1
TMEM201	H19	0.818244855	MASTL	H19	1
TMEM201	HCP5	0.62331403	MASTL	LINC00467	1
TMEM201	MCM3AP-AS1	0.79867958	MASTL	ZNRD1-AS1	1
TMEM201	ZNRD1-AS1	0.659570672	MBOAT1	MCM3AP-AS1	1
TMEM208	SCARNA9	0.735268132	MBOAT2	TP53TG1	1
TMEM242	H19	0.754611445	MCAM	DLEU2	1
TMEM242	HCP5	0.761361476	MCF2L	DLEU2	1

TMEM242	ZNRD1-AS1	0.507115694	MCF2L	H19	1
TMEM25	H19	0.766807466	MCF2L	TPT1-AS1	1
TMEM25	HCP5	0.691017876	MCF2L	ZNRD1-AS1	1
TMEM25	LINC00467	0.996059738	MCM2	KTN1-AS1	1
TMEM25	ZNRD1-AS1	0.667284195	MCM2	MCM3AP-AS1	1
TMEM38B	RHPN1-AS1	0.747882228	MCM3	HCP5	1
TMEM38B	SNHG3	0.907986613	MCM4	HCP5	1
TMEM38B	SNHG5	0.709943329	MCM4	KTN1-AS1	1
TMEM39A	SNHG5	0.677296843	MCM4	MCM3AP-AS1	1
TMEM45A	SNHG5	0.620716926	MCM4	SNHG3	1
TMEM50B	SNHG3	0.710500651	MCM5	MCM3AP-AS1	1
TMEM50B	SNHG5	0.887791945	MCM5	SCARNA9	1
TMEM57	H19	0.714629732	MCM6	SCARNA9	1
TMEM57	MCM3AP-AS1	0.703172077	MCM6	SNHG3	1
TMEM57	ZNRD1-AS1	0.672801132	MCM8	SNHG3	1
TMEM63B	SNHG3	0.557963561	MDFIC	H19	1
TMEM63B	SNHG5	0.540474206	MDFIC	ZNRD1-AS1	1
TMEM65	H19	0.638883672	MDK	SNHG3	1
TMEM65	HCP5	0.745766887	MDM2	HCP5	1
TMEM65	MCM3AP-AS1	0.875347264	ME1	DLEU2	1
TMEM65	ZNRD1-AS1	0.767654486	ME1	H19	1
TMEM80	MCM3AP-AS1	0.868265112	ME1	SNHG5	1
TMEM87B	SNHG5	0.707075199	MECOM	HCP5	1
TMEM97	HCP5	0.686594254	MECOM	MCM3AP-AS1	1
TMOD2	SNHG3	0.788775059	MECOM	SNHG3	1
TMPO	SNHG3	0.749689289	MED11	MCM3AP-AS1	1
TMTC4	HCP5	0.507549986	MED11	SNHG3	1
TMTC4	KTN1-AS1	0.795808336	MED12L	HCP5	1
TMTC4	TP53TG1	0.946194704	MED12L	KTN1-AS1	1
TNFAIP2	ZNRD1-AS1	0.665983997	MED12L	MCM3AP-AS1	1
TNFAIP3	SNHG3	0.625625836	MED12L	TP53TG1	1
TNFAIP8	SCARNA9	0.517640392	MED15	DLEU2	1
TNFAIP8	SNHG3	0.767558392	MED15	H19	1
TNFRSF10B	MCM3AP-AS1	0.590840623	MED15	RUSC1-AS1	1
TNFRSF10B	TPT1-AS1	0.78992897	MED15	ZNRD1-AS1	1
TNFRSF10B	ZNRD1-AS1	0.618451539	MED16	H19	1
TNFRSF10D	SNHG3	0.628317029	MED16	ZNRD1-AS1	1
TNFSF10	SNHG3	0.728866501	MEF2A	DLEU2	1
TNFSF10	SNHG5	0.671370604	MEF2A	H19	1
TNFSF12	H19	0.515225375	MEF2A	SNHG5	1
TNFSF12	HCP5	0.924922067	MEF2A	ZNRD1-AS1	1
TNIK	ZNRD1-AS1	0.656325911	MEIS1	KTN1-AS1	1
TNIP1	SNHG3	0.642073871	MEIS1	MCM3AP-AS1	1
TOB1	SNHG3	0.885548079	MEIS1	SCARNA9	1
TOM1L2	ZNRD1-AS1	0.981619992	MEIS1	SNHG3	1
TOMM22	SNHG5	0.707229684	MELK	HCP5	1
TOMM34	H19	0.707685057	MELK	KTN1-AS1	1
TOMM34	MCM3AP-AS1	0.869375765	MELK	SNHG3	1
TOMM34	ZNRD1-AS1	0.753451507	MESDC1	HCP5	1
TOP2A	KTN1-AS1	0.675136208	MESDC1	SCARNA9	1
TOP2A	MCM3AP-AS1	0.543065101	MEST	HCP5	1

TOPBP1	TPT1-AS1	0.681226416	MEST	RHPN1-AS1	1
TOR1A	RHPN1-AS1	0.730986074	MEST	SNHG3	1
TOR1A	SNHG3	0.884434672	METAP1	HCP5	1
TOX	RHPN1-AS1	0.872506423	METAP1	KTN1-AS1	1
TP53	H19	0.645114585	METAP1	MCM3AP-AS1	1
TP53	HCP5	0.538175243	METAP1	SNHG3	1
TP53	TPT1-AS1	0.73429488	METTL13	MCM3AP-AS1	1
TP53	ZNRD1-AS1	0.895518191	METTL2A	H19	1
TP53INP1	DLEU2	0.656945854	METTL2A	SNHG5	1
TP53INP1	MCM3AP-AS1	0.601769291	METTL2A	ZNRD1-AS1	1
TP53INP1	TPT1-AS1	0.842619332	METTL9	SNHG3	1
TP53INP1	ZNRD1-AS1	0.750584026	METTL9	TP53TG1	1
TPCN1	SNHG5	0.78092126	MEX3D	HCP5	1
TPP1	HCP5	0.878377233	MFN2	HCP5	1
TPP1	LINC00467	0.906648376	MFN2	MCM3AP-AS1	1
TPP1	MCM3AP-AS1	0.815154746	MFSD2A	HCP5	1
TPP1	ZNRD1-AS1	0.721270254	MFSD2A	MCM3AP-AS1	1
TPX2	H19	0.907214077	MFSD2A	SCARNA9	1
TPX2	HCP5	0.551140049	MFSD9	ZNRD1-AS1	1
TRAF3IP2	DLEU2	0.552892755	MGAT3	SCARNA9	1
TRAF3IP2	TPT1-AS1	0.894857376	MGLL	H19	1
TRAPPC8	SNHG3	0.662819808	MGLL	SNHG5	1
TRAPPC9	H19	0.680583975	MGLL	ZNRD1-AS1	1
TRERF1	H19	0.805070527	MGST1	HCP5	1
TRERF1	KTN1-AS1	0.951552864	MGST1	MCM3AP-AS1	1
TRERF1	MCM3AP-AS1	0.892940427	MGST1	SCARNA9	1
TRERF1	TPT1-AS1	0.636288135	MGST1	SNHG3	1
TRERF1	ZNRD1-AS1	0.789512374	MGST3	SNHG3	1
TRIAP1	H19	0.868997095	MICA	HCP5	1
TRIAP1	LINC00467	0.725409469	MICAL2	MCM3AP-AS1	1
TRIB1	SNHG5	0.804849757	MIF4GD	HCP5	1
TRIB2	SNHG3	0.808119302	MIF4GD	RHPN1-AS1	1
TRIB2	SNHG5	0.552985691	MIPOL1	TP53TG1	1
TRIM14	H19	0.632455576	MKI67	HCP5	1
TRIM14	MCM3AP-AS1	0.944107313	MKNK1	RUSC1-AS1	1
TRIM14	ZNRD1-AS1	0.859744387	MKNK1	SNHG5	1
TRIM16	SNHG3	0.836723485	MKNK1	ZNRD1-AS1	1
TRIM36	SNHG3	0.776260433	MLEC	MCM3AP-AS1	1
TRIM4	H19	0.927535774	MLEC	SNHG3	1
TRIM4	KTN1-AS1	0.741223805	MLF1	DLEU2	1
TRIM4	ZNRD1-AS1	0.565305901	MLF1	ZNRD1-AS1	1
TRIM45	ZNRD1-AS1	0.889801086	MLLT3	HCP5	1
TRIM59	H19	0.546673525	MLLT3	MCM3AP-AS1	1
TRIM59	KTN1-AS1	0.829721908	MLLT3	TP53TG1	1
TRIM59	MCM3AP-AS1	0.751326575	MMAA	H19	1
TRIM59	ZNRD1-AS1	0.629739796	MMAA	ZNRD1-AS1	1
TRIM65	H19	0.815720176	MMD	HCP5	1
TRIO	ZNRD1-AS1	0.588892808	MMD	MCM3AP-AS1	1
TRIP10	H19	0.561116741	MMD	SNHG3	1
TRIP13	HCP5	0.676072707	MMP14	DLEU2	1
TRIT1	MCM3AP-AS1	0.517669139	MMS22L	HCP5	1

TRMT6	SNHG5	0.690935398	MN1	H19	1
TRUB1	H19	0.649321838	MN1	ZNRD1-AS1	1
TRUB1	HCP5	0.663625531	MNAT1	MCM3AP-AS1	1
TRUB1	LINC00467	0.748623665	MNAT1	SNHG3	1
TSEN15	MCM3AP-AS1	0.641408683	MOB3B	MCM3AP-AS1	1
TSEN15	ZNRD1-AS1	0.552209072	MOB3C	SCARNA9	1
TSHZ1	H19	0.735462196	MORN2	HCP5	1
TSHZ1	KTN1-AS1	0.961251915	MORN2	RHPN1-AS1	1
TSHZ1	MCM3AP-AS1	0.933805198	MORN2	SNHG3	1
TSHZ1	TP53TG1	0.62780325	MOSPD3	HCP5	1
TSHZ1	TPT1-AS1	0.771824878	MPDZ	HCP5	1
TSHZ1	ZNRD1-AS1	0.865567932	MPDZ	MCM3AP-AS1	1
TSHZ3	H19	0.892379564	MPDZ	TP53TG1	1
TSKU	HCP5	0.553750024	MPI	MCM3AP-AS1	1
TSPAN14	H19	0.560543844	MPP2	H19	1
TSPO	H19	0.611710325	MPP6	KTN1-AS1	1
TSPO	MCM3AP-AS1	0.593499321	MPP6	MCM3AP-AS1	1
TSR1	H19	0.678098105	MPP6	SCARNA9	1
TTC28	SNHG3	0.641307696	MPPED2	KTN1-AS1	1
TTC30B	H19	0.779828912	MPPED2	MCM3AP-AS1	1
TTC30B	HCP5	0.677618693	MPV17L	SNHG3	1
TTC30B	KTN1-AS1	0.517844277	MPZL2	MCM3AP-AS1	1
TTC7B	SNHG3	0.846882007	MRC2	LINC00467	1
TTC9	HCP5	0.588697916	MRC2	ZNRD1-AS1	1
TTC9	KTN1-AS1	0.840419886	MRE11A	RHPN1-AS1	1
TTC9	MCM3AP-AS1	0.89856259	MREG	H19	1
TTC9	ZNRD1-AS1	0.932171075	MREG	SNHG5	1
TTK	H19	0.593706218	MREG	ZNRD1-AS1	1
TTLL7	H19	0.877124532	MRPL13	SCARNA9	1
TTLL7	KTN1-AS1	0.718020161	MRPL16	SNHG3	1
TTLL7	LINC00467	0.649572384	MRPL3	RHPN1-AS1	1
TTLL7	MCM3AP-AS1	0.66837472	MRPL30	SNHG3	1
TTLL7	TPT1-AS1	0.593262622	MRPL30	TP53TG1	1
TTLL7	ZNRD1-AS1	0.584451947	MRPL35	MCM3AP-AS1	1
TTYH3	H19	0.701071559	MRPL35	SNHG3	1
TTYH3	ZNRD1-AS1	0.871712714	MRPL37	KTN1-AS1	1
TUBGCP4	H19	0.728229947	MRPL37	MCM3AP-AS1	1
TUBGCP4	KTN1-AS1	0.701050736	MRPL37	TP53TG1	1
TUBGCP4	MCM3AP-AS1	0.575798339	MRPL39	SNHG3	1
TXLNB	TP53TG1	0.536183215	MRPL40	H19	1
TXN	SNHG3	0.987565644	MRPL40	ZNRD1-AS1	1
TXNDC9	ZNRD1-AS1	0.725347083	MRPS23	KTN1-AS1	1
TYSND1	HCP5	0.685346387	MRPS23	MCM3AP-AS1	1
TYSND1	ZNRD1-AS1	0.75838934	MRPS34	MCM3AP-AS1	1
UBA2	H19	0.640125704	MRPS35	HCP5	1
UBA2	TP53TG1	0.837033425	MRPS35	KTN1-AS1	1
UBASH3B	SNHG5	0.622332371	MRPS35	MCM3AP-AS1	1
UBE2C	H19	0.883085553	MRRF	HCP5	1
UBE2C	TP53TG1	0.983617577	MRRF	MCM3AP-AS1	1
UBE2E2	HCP5	0.647062643	MRRF	SCARNA9	1
UBE2G2	KTN1-AS1	0.712762275	MRS2	HCP5	1

UBE2G2	ZNRD1-AS1	0.785942005	MRS2	SNHG3	1
UBE2H	ZNRD1-AS1	0.536328057	MRTO4	HCP5	1
UBE2K	H19	0.696304424	MSH2	HCP5	1
UBE2K	HCP5	0.807740665	MSH2	MCM3AP-AS1	1
UBE2K	KTN1-AS1	0.638663511	MSH2	SNHG3	1
UBE2K	MCM3AP-AS1	0.507267846	MSH2	TP53TG1	1
UBE2L6	HCP5	0.503282739	MSH6	KTN1-AS1	1
UBE2T	SNHG3	0.605652478	MSRB2	MCM3AP-AS1	1
UBE2V2	H19	0.827952825	MSRB3	SNHG3	1
UBE2V2	TP53TG1	0.897787889	MT1E	KTN1-AS1	1
UBN2	KTN1-AS1	0.937416402	MT1E	MCM3AP-AS1	1
UBN2	MCM3AP-AS1	0.980078903	MT1X	KTN1-AS1	1
UBN2	TPT1-AS1	0.891571168	MT1X	MCM3AP-AS1	1
UBN2	ZNRD1-AS1	0.992436786	MT1X	SCARNA9	1
UBR1	SNHG3	0.757891108	MTBP	HCP5	1
UBR1	SNHG5	0.883121704	MTBP	SNHG3	1
UBXN2B	MCM3AP-AS1	0.744887668	MTG1	HCP5	1
UBXN8	H19	0.767528871	MTHFD2	HCP5	1
UEVLD	H19	0.923932544	MTHFD2	SNHG3	1
UGCG	SNHG3	0.950306123	MTHFD2	TP53TG1	1
UHRF1	H19	0.826818374	MTMR10	SNHG5	1
UHRF1	ZNRD1-AS1	0.731633032	MTMR10	ZNRD1-AS1	1
UHRF1BP1	HCP5	0.742617598	MTMR14	H19	1
ULK1	ZNRD1-AS1	0.976239058	MTMR14	ZNRD1-AS1	1
UNC5B	SCARNA9	0.795692671	MTMR9	H19	1
UNG	H19	0.51143033	MTMR9	RUSC1-AS1	1
UNG	LINC00467	0.758157631	MTMR9	SNHG5	1
UPF3B	MCM3AP-AS1	0.81223455	MTMR9	ZNRD1-AS1	1
URB2	SNHG5	0.523350989	MTRF1L	SCARNA9	1
USP11	ZNRD1-AS1	0.630825553	MTSS1L	H19	1
USP13	H19	0.578395079	MTSS1L	ZNRD1-AS1	1
USP2	H19	0.675593144	MTUS1	H19	1
USP2	MCM3AP-AS1	0.812582702	MTUS1	ZNRD1-AS1	1
USP2	ZNRD1-AS1	0.685928584	MTX2	DLEU2	1
UTP14A	MCM3AP-AS1	0.761448288	MTX2	SNHG5	1
UTRN	KTN1-AS1	0.786935791	MUC1	SNHG3	1
UTRN	MCM3AP-AS1	0.858905985	MXD1	HCP5	1
UTRN	ZNRD1-AS1	0.910744043	MXD1	MCM3AP-AS1	1
VAMP4	MCM3AP-AS1	0.934058778	MXD1	TP53TG1	1
VAMP4	ZNRD1-AS1	0.879914236	MXD4	H19	1
VAMP8	H19	0.750840616	MXI1	H19	1
VAMP8	MCM3AP-AS1	0.734441595	MXI1	SNHG5	1
VAMP8	ZNRD1-AS1	0.585569632	MXI1	ZNRD1-AS1	1
VANGL1	HCP5	0.839208816	MYBL1	HCP5	1
VBP1	SNHG5	0.562109992	MYBL1	MCM3AP-AS1	1
VCAN	SNHG3	0.95548916	MYBL1	SNHG3	1
VCAN	SNHG5	0.822837589	MYBL2	HCP5	1
VEGFA	SNHG5	0.810927392	MYCBP2	DLEU2	1
VGLL4	H19	0.885847844	MYCBP2	H19	1
VGLL4	ZNRD1-AS1	0.701054808	MYCBP2	SNHG5	1
VKORC1L1	HCP5	0.705428958	MYCBP2	TPT1-AS1	1

VMP1	SNHG5	0.863129534	MYCBP2	ZNRD1-AS1	1
VPS26B	HCP5	0.792942678	MYCN	H19	1
VPS37C	SNHG3	0.901218783	MYCN	LINC00467	1
VRK1	H19	0.766919478	MYCN	ZNRD1-AS1	1
VRK1	HCP5	0.515287924	MYD88	SNHG3	1
WASF1	HCP5	0.505833668	MYH9	DLEU2	1
WASF1	KTN1-AS1	0.913115137	MYH9	H19	1
WASF1	MCM3AP-AS1	0.96243635	MYH9	SNHG5	1
WASF1	ZNRD1-AS1	0.983282841	MYH9	TPT1-AS1	1
WASF2	RUSC1-AS1	0.621505365	MYH9	ZNRD1-AS1	1
WASL	DLEU2	0.688434165	MYLIP	DLEU2	1
WASL	HCP5	0.749716466	MYLIP	H19	1
WASL	SCARNA9	0.838770915	MYLIP	SNHG5	1
WBP1	ZNRD1-AS1	0.966909115	MYLIP	TPT1-AS1	1
WDFY3	SNHG3	0.897192666	MYLIP	ZNRD1-AS1	1
WDR12	SNHG3	0.651092595	MYO18A	DLEU2	1
WDR35	SNHG3	0.871532433	MYO18A	SNHG5	1
WDR43	KTN1-AS1	0.878231301	MYO18A	TPT1-AS1	1
WDR43	MCM3AP-AS1	0.825163057	MYO19	HCP5	1
WDR43	ZNRD1-AS1	0.730556147	MYO19	SNHG3	1
WDR47	H19	0.82864934	MYO19	TP53TG1	1
WDR47	HCP5	0.682127675	MYO1D	HCP5	1
WDR47	MCM3AP-AS1	0.603195973	MYO1E	DLEU2	1
WDR47	TP53TG1	0.977670664	MYO1E	H19	1
WDR5	RHPN1-AS1	0.610716631	MYO1G	ZNRD1-AS1	1
WDR5	SNHG3	0.74541498	MYO5A	DLEU2	1
WDR5B	HCP5	0.911218948	MYO5A	SNHG5	1
WDR81	ZNRD1-AS1	0.958441278	MYO5A	TPT1-AS1	1
WDTC1	LINC00467	0.810479216	MYO5A	ZNRD1-AS1	1
WDTC1	MCM3AP-AS1	0.930362331	MYO5C	KTN1-AS1	1
WDTC1	ZNRD1-AS1	0.864978828	MYO5C	MCM3AP-AS1	1
WEE1	HCP5	0.871258069	MYOF	LINC00467	1
WEE1	KTN1-AS1	0.856836242	MYOZ3	SNHG5	1
WEE1	MCM3AP-AS1	0.835319745	N6AMT2	H19	1
WEE1	ZNRD1-AS1	0.77743819	NAA15	HCP5	1
WFS1	RHPN1-AS1	0.90735122	NAA15	KTN1-AS1	1
WHAMM	KTN1-AS1	0.751176823	NAA15	MCM3AP-AS1	1
WIBG	MCM3AP-AS1	0.620458909	NAA15	SCARNA9	1
WIBG	ZNRD1-AS1	0.519110871	NAA15	SNHG3	1
WLS	SNHG3	0.938605325	NAA15	TP53TG1	1
WNK3	MCM3AP-AS1	0.683669394	NAA16	HCP5	1
WNK3	ZNRD1-AS1	0.814227812	NAA16	MCM3AP-AS1	1
WNT1	ZNRD1-AS1	0.822011344	NAA16	SNHG3	1
WWTR1	SNHG5	0.903926335	NAA38	SNHG5	1
XPO7	H19	0.869126121	NAA50	HCP5	1
XPO7	HCP5	0.610159699	NAA50	KTN1-AS1	1
XPO7	TP53TG1	0.997843966	NAA50	SCARNA9	1
XPOT	H19	0.79848952	NAE1	KTN1-AS1	1
XPOT	ZNRD1-AS1	0.71860358	NAE1	MCM3AP-AS1	1
XYLT1	LINC00467	0.540146641	NAE1	SCARNA9	1
XYLT1	MCM3AP-AS1	0.955308345	NAGLU	SNHG3	1

YARS	HCP5	0.570743129	NAGPA	DLEU2	1
YEATS4	H19	0.659762773	NAGPA	SNHG5	1
YEATS4	HCP5	0.714190432	NAGPA	TPT1-AS1	1
YIPF6	SNHG3	0.973442968	NAGPA	ZNRD1-AS1	1
YPEL1	ZNRD1-AS1	0.842046931	NAP1L2	HCP5	1
YPEL2	DLEU2	0.796519634	NAP1L5	HCP5	1
YPEL2	TPT1-AS1	0.659393439	NAP1L5	KTN1-AS1	1
YPEL2	ZNRD1-AS1	0.664608995	NAPB	HCP5	1
YPEL5	DLEU2	0.547048763	NAPEPLD	HCP5	1
YPEL5	RHPN1-AS1	0.63128392	NAPEPLD	MCM3AP-AS1	1
YRDC	SNHG3	0.977879893	NAPG	DLEU2	1
YTHDF2	SNHG5	0.846748837	NAPG	TPT1-AS1	1
ZBTB1	TPT1-AS1	0.892289955	NAPG	ZNRD1-AS1	1
ZBTB10	MCM3AP-AS1	0.509422975	NASP	HCP5	1
ZBTB10	TPT1-AS1	0.848767379	NAV1	DLEU2	1
ZBTB10	ZNRD1-AS1	0.616449517	NAV1	H19	1
ZBTB17	ZNRD1-AS1	0.617488348	NAV1	LINC00467	1
ZBTB20	MCM3AP-AS1	0.620955465	NAV1	TPT1-AS1	1
ZBTB20	ZNRD1-AS1	0.526768024	NAV1	ZNRD1-AS1	1
ZBTB38	HCP5	0.875901386	NBEA	HCP5	1
ZBTB38	ZNRD1-AS1	0.6667001	NBEA	KTN1-AS1	1
ZBTB4	HCP5	0.632586832	NBEA	MCM3AP-AS1	1
ZBTB4	LINC00467	0.784524766	NBEA	SNHG3	1
ZBTB4	MCM3AP-AS1	0.977851455	NBEAL1	H19	1
ZBTB4	ZNRD1-AS1	0.948057791	NBEAL1	ZNRD1-AS1	1
ZBTB43	H19	0.529334925	NBR1	HCP5	1
ZBTB44	H19	0.560319662	NBR1	MCM3AP-AS1	1
ZBTB44	HCP5	0.77160118	NCAPD2	HCP5	1
ZBTB44	KTN1-AS1	0.942678975	NCAPD3	SCARNA9	1
ZBTB44	MCM3AP-AS1	0.89354528	NCAPG	KTN1-AS1	1
ZBTB44	TPT1-AS1	0.553114032	NCAPG	MCM3AP-AS1	1
ZBTB44	ZNRD1-AS1	0.801018893	NCKAP1	HCP5	1
ZC3H6	KTN1-AS1	0.988110795	NCKAP1	MCM3AP-AS1	1
ZC3H6	MCM3AP-AS1	0.989861915	NCKAP1	SNHG3	1
ZC3H6	ZNRD1-AS1	0.953643279	NCOR2	MCM3AP-AS1	1
ZC3HAV1L	HCP5	0.823419559	NDFIP1	MCM3AP-AS1	1
ZC3HAV1L	LINC00467	0.684063312	NDFIP1	SNHG3	1
ZDBF2	HCP5	0.53240844	NDFIP1	TP53TG1	1
ZDHHC1	LINC00467	0.672715947	NDUFA5	KTN1-AS1	1
ZDHHC1	ZNRD1-AS1	0.986368037	NDUFA5	MCM3AP-AS1	1
ZDHHC17	RUSC1-AS1	0.741774567	NDUFA5	TP53TG1	1
ZDHHC17	TPT1-AS1	0.828976829	NDUFAB1	HCP5	1
ZDHHC17	ZNRD1-AS1	0.595900967	NDUFAB1	SCARNA9	1
ZDHHC2	H19	0.535379666	NDUFB6	SNHG3	1
ZFP36L2	H19	0.502918372	NDUFS6	H19	1
ZFP36L2	HCP5	0.520559185	NECAB3	ZNRD1-AS1	1
ZFP36L2	MCM3AP-AS1	0.996334275	NEDD9	DLEU2	1
ZFP36L2	TPT1-AS1	0.822017496	NEDD9	H19	1
ZFP36L2	ZNRD1-AS1	0.959430884	NEDD9	LINC00467	1
ZFP90	H19	0.69267361	NEDD9	TPT1-AS1	1
ZFP90	HCP5	0.736951334	NEFH	HCP5	1

ZFP90	ZNRD1-AS1	0.718722761	NEGR1	DLEU2	1
ZHX1	HCP5	0.685873576	NEGR1	SNHG5	1
ZHX1	MCM3AP-AS1	0.962779486	NEGR1	TPT1-AS1	1
ZHX1	TP53TG1	0.532724765	NEGR1	ZNRD1-AS1	1
ZHX1	ZNRD1-AS1	0.918930249	NEIL3	SCARNA9	1
ZHX2	RUSC1-AS1	0.719246792	NEIL3	SNHG3	1
ZHX2	ZNRD1-AS1	0.920072569	NEK2	HCP5	1
ZHX3	MCM3AP-AS1	0.795544987	NEK6	SNHG5	1
ZHX3	ZNRD1-AS1	0.838383215	NEK9	HCP5	1
ZKSCAN1	KTN1-AS1	0.989770507	NEK9	SNHG3	1
ZKSCAN1	MCM3AP-AS1	0.984308991	NET1	HCP5	1
ZKSCAN1	TPT1-AS1	0.835773651	NET1	SNHG3	1
ZKSCAN3	HCP5	0.807717539	NETO2	H19	1
ZKSCAN4	H19	0.848610751	NETO2	RUSC1-AS1	1
ZKSCAN4	ZNRD1-AS1	0.714845573	NETO2	ZNRD1-AS1	1
ZMAT3	SNHG5	0.785958024	NFAT5	HCP5	1
ZMPSTE24	SNHG3	0.9820382	NFAT5	SCARNA9	1
ZMYM3	ZNRD1-AS1	0.723920578	NFAT5	SNHG3	1
ZNF100	TPT1-AS1	0.712095705	NFE2L1	HCP5	1
ZNF117	KTN1-AS1	0.822008506	NFE2L1	MCM3AP-AS1	1
ZNF138	KTN1-AS1	0.980641671	NFE2L1	SNHG3	1
ZNF138	MCM3AP-AS1	0.93840562	NFIA	HCP5	1
ZNF155	MCM3AP-AS1	0.627351851	NFIA	KTN1-AS1	1
ZNF2	H19	0.657310955	NFIA	MCM3AP-AS1	1
ZNF2	HCP5	0.832919856	NFIA	SCARNA9	1
ZNF217	H19	0.716948254	NFIA	TP53TG1	1
ZNF217	HCP5	0.790865326	NFIL3	SCARNA9	1
ZNF217	MCM3AP-AS1	0.694843105	NFKB2	H19	1
ZNF217	ZNRD1-AS1	0.542426384	NFKBIA	DLEU2	1
ZNF22	HCP5	0.847769046	NFKBIA	H19	1
ZNF223	TP53TG1	0.546665368	NFKBIA	TPT1-AS1	1
ZNF230	H19	0.847474429	NFYB	MCM3AP-AS1	1
ZNF239	ZNRD1-AS1	0.963720677	NHEJ1	MCM3AP-AS1	1
ZNF248	H19	0.534017263	NID1	DLEU2	1
ZNF248	HCP5	0.792379673	NID1	H19	1
ZNF248	KTN1-AS1	0.933364089	NID1	SNHG5	1
ZNF248	ZNRD1-AS1	0.794719351	NID1	TPT1-AS1	1
ZNF250	H19	0.954546872	NID1	ZNRD1-AS1	1
ZNF286A	DLEU2	0.604404245	NIF3L1	HCP5	1
ZNF326	KTN1-AS1	0.825790486	NIF3L1	SNHG3	1
ZNF326	MCM3AP-AS1	0.877407998	NIP7	SCARNA9	1
ZNF326	TPT1-AS1	0.962238769	NIP7	SNHG3	1
ZNF326	ZNRD1-AS1	0.904579405	NIPAL2	MCM3AP-AS1	1
ZNF362	H19	0.604699472	NIPAL3	RHPN1-AS1	1
ZNF362	HCP5	0.754919711	NIPAL4	HCP5	1
ZNF362	MCM3AP-AS1	0.887335615	NIPAL4	TP53TG1	1
ZNF362	ZNRD1-AS1	0.787023454	NIPSNAP1	HCP5	1
ZNF367	SNHG3	0.596536829	NIPSNAP1	MCM3AP-AS1	1
ZNF395	HCP5	0.861222171	NKIRAS1	H19	1
ZNF395	KTN1-AS1	0.868460907	NKIRAS1	LINC00467	1
ZNF395	MCM3AP-AS1	0.801004368	NKIRAS1	SNHG5	1

ZNF395	ZNRD1-AS1	0.690399867	NKIRAS1	ZNRD1-AS1	1
ZNF397	MCM3AP-AS1	0.532525448	NLGN1	KTN1-AS1	1
ZNF397	ZNRD1-AS1	0.658642066	NLGN1	MCM3AP-AS1	1
ZNF485	KTN1-AS1	0.703141923	NLGN1	TP53TG1	1
ZNF485	MCM3AP-AS1	0.571459115	NLK	HCP5	1
ZNF492	TP53TG1	0.709192698	NLK	KTN1-AS1	1
ZNF493	KTN1-AS1	0.98577148	NLK	MCM3AP-AS1	1
ZNF516	H19	0.984391749	NLK	RHPN1-AS1	1
ZNF516	TP53TG1	0.824974728	NLK	SCARNA9	1
ZNF521	H19	0.614965821	NLK	SNHG3	1
ZNF521	KTN1-AS1	0.994117621	NLK	TP53TG1	1
ZNF521	MCM3AP-AS1	0.963254841	NLN	HCP5	1
ZNF521	TPT1-AS1	0.708170105	NME4	HCP5	1
ZNF521	ZNRD1-AS1	0.889677367	NME4	SNHG3	1
ZNF566	HCP5	0.57686899	NMT2	KTN1-AS1	1
ZNF566	TPT1-AS1	0.809329942	NMT2	MCM3AP-AS1	1
ZNF569	TP53TG1	0.95726571	NOG	HCP5	1
ZNF583	H19	0.710733207	NOG	SCARNA9	1
ZNF583	TP53TG1	0.895802692	NOG	SNHG3	1
ZNF583	ZNRD1-AS1	0.598608897	NOL12	DLEU2	1
ZNF607	MCM3AP-AS1	0.884965226	NOL12	TPT1-AS1	1
ZNF609	MCM3AP-AS1	0.680993572	NOLC1	MCM3AP-AS1	1
ZNF609	ZNRD1-AS1	0.772300557	NOLC1	RHPN1-AS1	1
ZNF624	HCP5	0.675830228	NOP2	MCM3AP-AS1	1
ZNF624	MCM3AP-AS1	0.599072608	NOTCH2	DLEU2	1
ZNF624	TP53TG1	0.979405356	NOTCH2	H19	1
ZNF652	H19	0.603615763	NOTCH2	LINC00467	1
ZNF652	HCP5	0.75913531	NOTCH2	RUSC1-AS1	1
ZNF652	KTN1-AS1	0.940949087	NOTCH2	SNHG5	1
ZNF652	MCM3AP-AS1	0.884305325	NOTCH2	TPT1-AS1	1
ZNF652	TPT1-AS1	0.534770717	NOTCH2	ZNRD1-AS1	1
ZNF652	ZNRD1-AS1	0.783306565	NPC2	DLEU2	1
ZNF672	HCP5	0.792316953	NPC2	TPT1-AS1	1
ZNF681	H19	0.616468697	NPC2	ZNRD1-AS1	1
ZNF681	HCP5	0.873837406	NPLOC4	DLEU2	1
ZNF695	H19	0.705349184	NPLOC4	H19	1
ZNF695	ZNRD1-AS1	0.758743139	NPLOC4	LINC00467	1
ZNF730	KTN1-AS1	0.854111937	NPLOC4	SNHG5	1
ZNF738	MCM3AP-AS1	0.923175456	NPLOC4	ZNRD1-AS1	1
ZNF738	ZNRD1-AS1	0.856457524	NPR3	HCP5	1
ZNF75A	H19	0.611961141	NPR3	KTN1-AS1	1
ZNF75A	ZNRD1-AS1	0.822911303	NPR3	MCM3AP-AS1	1
ZNF77	KTN1-AS1	0.909338087	NQO1	H19	1
ZNF77	MCM3AP-AS1	0.940903765	NQO1	ZNRD1-AS1	1
ZNF823	HCP5	0.541478946	NR2C2AP	HCP5	1
ZNRF1	MCM3AP-AS1	0.792599062	NR2C2AP	TP53TG1	1
ZNRF1	ZNRD1-AS1	0.65495405	NR4A1	MCM3AP-AS1	1
ZSWIM6	TPT1-AS1	0.674738323	NR4A1	SNHG3	1
ZW10	SNHG3	0.911618305	NR4A2	HCP5	1
ZWILCH	LINC00467	0.501480396	NR4A2	MCM3AP-AS1	1
ZXDA	HCP5	0.942629294	NR4A2	SNHG3	1

ZXDA	SCARNA9	0.803893807	NR4A2	TP53TG1	1
ZXDB	HCP5	0.963091751	NRCAM	SNHG5	1
ZXDB	MCM3AP-AS1	0.678164085	NREP	HCP5	1
ZXDB	ZNRD1-AS1	0.582787679	NRIP1	DLEU2	1
AAAS	MCM3AP-AS1	0.688450018	NRIP1	TPT1-AS1	1
ACP6	ZNRD1-AS1	0.643008875	NRIP1	ZNRD1-AS1	1
ADAM28	KTN1-AS1	0.975682321	NRIP3	H19	1
ADAM28	MCM3AP-AS1	0.980993115	NRIP3	ZNRD1-AS1	1
ADAMTS9	SCARNA9	0.738433681	NRP1	DLEU2	1
AGER	MCM3AP-AS1	0.781934809	NRP1	H19	1
AGER	ZNRD1-AS1	0.853169739	NRP1	RUSC1-AS1	1
AMER1	H19	0.69785947	NRP1	TPT1-AS1	1
AMER1	HCP5	0.694059925	NRP1	ZNRD1-AS1	1
AMER1	ZNRD1-AS1	0.762835015	NRP2	H19	1
ARL9	H19	0.858277969	NRP2	RUSC1-AS1	1
BCS1L	KTN1-AS1	0.909138222	NRP2	ZNRD1-AS1	1
BEND5	HCP5	0.825588219	NSMCE2	DLEU2	1
BSPRY	MCM3AP-AS1	0.734240145	NSMCE2	SNHG5	1
BSPRY	ZNRD1-AS1	0.626072749	NSMCE2	TPT1-AS1	1
C14orf93	MCM3AP-AS1	0.758835276	NSUN7	HCP5	1
CCDC171	TP53TG1	0.768766207	NT5DC3	KTN1-AS1	1
CCSER1	SNHG3	0.939952733	NT5DC3	MCM3AP-AS1	1
CD34	ZNRD1-AS1	0.846253494	NTN1	H19	1
CD8A	SNHG3	0.674080016	NUAK2	SNHG5	1
CDH9	SNHG5	0.547870878	NUAK2	ZNRD1-AS1	1
CEP152	TPT1-AS1	0.517549322	NUCB2	HCP5	1
CH25H	SNHG5	0.935418447	NUCB2	MCM3AP-AS1	1
CHEK2	HCP5	0.560910935	NUDCD2	HCP5	1
CLUH	MCM3AP-AS1	0.96293867	NUDCD2	KTN1-AS1	1
CLUH	ZNRD1-AS1	0.911991605	NUDCD2	MCM3AP-AS1	1
CRHBP	MCM3AP-AS1	0.970024689	NUDCD3	MCM3AP-AS1	1
CRHBP	ZNRD1-AS1	0.904706194	NUDCD3	SNHG3	1
CXCL13	SNHG5	0.824783077	NUDT15	H19	1
DLGAP3	MCM3AP-AS1	0.777491332	NUDT15	SNHG5	1
ELMSAN1	H19	0.791565255	NUDT6	MCM3AP-AS1	1
EXO5	H19	0.963183659	NUDT8	DLEU2	1
FAM213A	H19	0.611197497	NUDT8	H19	1
GAS2	SNHG5	0.645780675	NUDT8	TPT1-AS1	1
GCSAM	HCP5	0.888749289	NUDT8	ZNRD1-AS1	1
GCSAML	HCP5	0.81988752	NUF2	SNHG3	1
GLI1	RUSC1-AS1	0.951492168	NUMB	H19	1
GLI1	TPT1-AS1	0.724077145	NUMB	ZNRD1-AS1	1
GPLD1	KTN1-AS1	0.903049388	NUP107	HCP5	1
GPR155	HCP5	0.995714501	NUP155	SNHG3	1
GSTM5	SCARNA9	0.948207151	NUP205	HCP5	1
GTF2IRD2B	ZNRD1-AS1	0.70199927	NUP205	SCARNA9	1
HGD	SNHG5	0.621040353	NUP205	SNHG3	1
HIST1H1A	TPT1-AS1	0.511825789	NUP210	HCP5	1
HIST1H2AM	SNHG5	0.52216765	NUP210	MCM3AP-AS1	1
HIST2H3C	RHPN1-AS1	0.721682806	NUP210	SCARNA9	1
HIST2H4B	KTN1-AS1	0.93618463	NUP35	HCP5	1

HIST2H4B	MCM3AP-AS1	0.959212705	NUP35	KTN1-AS1	1
HIST2H4B	ZNRD1-AS1	0.949154944	NUP35	MCM3AP-AS1	1
IDNK	H19	0.660440182	NUP62CL	H19	1
IDNK	MCM3AP-AS1	0.897104281	NUP62CL	SNHG5	1
IDNK	ZNRD1-AS1	0.793306932	NUP88	HCP5	1
IL1R2	SNHG5	0.781933433	NUPL1	HCP5	1
IL24	SNHG5	0.843794802	NUPL1	MCM3AP-AS1	1
KCNA4	SCARNA9	0.532791975	NUPL1	SCARNA9	1
KLHL42	SNHG3	0.584501766	NUPL1	TP53TG1	1
LAMC2	RHPN1-AS1	0.784900207	NUS1	DLEU2	1
LILRA2	H19	0.714228727	NUS1	H19	1
LILRA2	ZNRD1-AS1	0.879661864	NUS1	SNHG5	1
MMP10	SNHG5	0.96275652	NUS1	TPT1-AS1	1
MMP8	RUSC1-AS1	0.992088702	NUS1	ZNRD1-AS1	1
MTFR2	H19	0.862696673	NUSAP1	SCARNA9	1
NABP1	ZNRD1-AS1	0.59922382	NUSAP1	SNHG3	1
NSMF	TPT1-AS1	0.924463575	NXPH4	ZNRD1-AS1	1
PDE1B	RUSC1-AS1	0.779214344	NYNRIN	MCM3AP-AS1	1
PRIMPOL	MCM3AP-AS1	0.946270653	OAS1	DLEU2	1
PRIMPOL	ZNRD1-AS1	0.885832045	OAS2	DLEU2	1
RMDN3	H19	0.788869897	OAS2	RUSC1-AS1	1
ROPN1L	ZNRD1-AS1	0.611037178	OAS2	SNHG5	1
RPL21	HCP5	0.948377033	OAS2	TPT1-AS1	1
RPS2	HCP5	0.582465427	OAS2	ZNRD1-AS1	1
RRP9	MCM3AP-AS1	0.72621085	OAS3	MCM3AP-AS1	1
RSG1	ZNRD1-AS1	0.816605132	OAS3	SNHG3	1
S100B	H19	0.687794114	OCLN	RUSC1-AS1	1
S100B	HCP5	0.768306124	OCLN	SNHG5	1
SDE2	RHPN1-AS1	0.987018159	OGG1	MCM3AP-AS1	1
SERPINB2	SNHG5	0.68311387	OIP5	HCP5	1
SGIP1	SCARNA9	0.693298565	OIP5	MCM3AP-AS1	1
SKIDA1	TPT1-AS1	0.693482671	OPRL1	RUSC1-AS1	1
SMDT1	HCP5	0.681509422	OPTN	SNHG5	1
SMDT1	MCM3AP-AS1	0.938913382	ORAI2	HCP5	1
SMDT1	ZNRD1-AS1	0.856333472	ORAI3	RHPN1-AS1	1
TCAF2	MCM3AP-AS1	0.887016679	OSBPL10	HCP5	1
TCAF2	ZNRD1-AS1	0.793810189	OSBPL1A	H19	1
TLR3	SNHG5	0.86560497	OSBPL1A	ZNRD1-AS1	1
TLR6	KTN1-AS1	0.893065015	OSBPL6	HCP5	1
TLR6	MCM3AP-AS1	0.806558547	OSBPL6	MCM3AP-AS1	1
TNIP3	HCP5	0.590351536	OSGEP	SCARNA9	1
TPK1	H19	0.655762115	OSGEP	SNHG3	1
UBA7	MCM3AP-AS1	0.741193557	OSGEPL1	MCM3AP-AS1	1
WDR45B	H19	0.966088061	OTUD1	H19	1
XIRP2	H19	0.623558932	OTUD1	RUSC1-AS1	1
XIRP2	ZNRD1-AS1	0.88573586	OTUD1	SNHG5	1
ZBTB18	H19	0.725098265	OTUD1	ZNRD1-AS1	1
ZBTB18	HCP5	0.725351902	OTUD5	MCM3AP-AS1	1
ZBTB18	ZNRD1-AS1	0.688072263	OTUD6B	DLEU2	1
ZNF391	MCM3AP-AS1	0.793230833	OTUD6B	H19	1
ZNF391	ZNRD1-AS1	0.660404104	OTUD6B	LINC00467	1

ZNF454	ZNRD1-AS1	0.669671743	OTUD6B	SNHG5	1
			OTUD6B	TPT1-AS1	1
			OXA1L	H19	1
			OXA1L	ZNRD1-AS1	1
			OXNAD1	HCP5	1
			OXNAD1	TP53TG1	1
			P2RY10	HCP5	1
			P4HA1	SCARNA9	1
			P4HA1	SNHG3	1
			P4HA2	DLEU2	1
			P4HA2	TPT1-AS1	1
			PA2G4	MCM3AP-AS1	1
			PAFAH1B3	KTN1-AS1	1
			PAG1	DLEU2	1
			PAG1	H19	1
			PAG1	TPT1-AS1	1
			PAG1	ZNRD1-AS1	1
			PAICS	HCP5	1
			PAICS	MCM3AP-AS1	1
			PAIP2B	HCP5	1
			PAK1IP1	HCP5	1
			PALLD	H19	1
			PALLD	LINC00467	1
			PALLD	SNHG5	1
			PALLD	ZNRD1-AS1	1
			PALM2-AKAP	ZNRD1-AS1	1
			PAM	DLEU2	1
			PAM	H19	1
			PAM	SNHG5	1
			PAM	TPT1-AS1	1
			PAM	ZNRD1-AS1	1
			PANK1	HCP5	1
			PANK1	MCM3AP-AS1	1
			PANK1	SNHG3	1
			PANX1	HCP5	1
			PANX1	KTN1-AS1	1
			PANX1	MCM3AP-AS1	1
			PAPD5	HCP5	1
			PAPD5	KTN1-AS1	1
			PAPD5	MCM3AP-AS1	1
			PAPD5	RHPN1-AS1	1
			PAPD5	SNHG3	1
			PAPSS2	H19	1
			PAPSS2	LINC00467	1
			PAPSS2	SNHG5	1
			PAPSS2	ZNRD1-AS1	1
			PAQR4	HCP5	1
			PAQR5	ZNRD1-AS1	1
			PARD3B	SCARNA9	1
			PARK2	MCM3AP-AS1	1
			PARP1	HCP5	1

		PARP1	TP53TG1	1
		PARP12	ZNRD1-AS1	1
		PARP16	HCP5	1
		PARP3	MCM3AP-AS1	1
		PARP3	SNHG3	1
		PARP9	H19	1
		PARP9	SNHG5	1
		PARP9	ZNRD1-AS1	1
		PATZ1	HCP5	1
		PATZ1	KTN1-AS1	1
		PATZ1	MCM3AP-AS1	1
		PATZ1	SNHG3	1
		PAXIP1	HCP5	1
		PAXIP1	MCM3AP-AS1	1
		PAXIP1	SNHG3	1
		PBK	HCP5	1
		PBLD	DLEU2	1
		PBLD	H19	1
		PBLD	SNHG5	1
		PBLD	ZNRD1-AS1	1
		PBX3	HCP5	1
		PBX3	MCM3AP-AS1	1
		PBX3	RHPN1-AS1	1
		PBX3	SNHG3	1
		PBXIP1	H19	1
		PBXIP1	LINC00467	1
		PBXIP1	ZNRD1-AS1	1
		PCDH17	DLEU2	1
		PCDH9	HCP5	1
		PCDH9	MCM3AP-AS1	1
		PCGF2	MCM3AP-AS1	1
		PCGF2	SCARNA9	1
		PCGF2	SNHG3	1
		PCMTD1	DLEU2	1
		PCMTD1	H19	1
		PCMTD1	SNHG5	1
		PCMTD1	TPT1-AS1	1
		PCMTD2	KTN1-AS1	1
		PCMTD2	SNHG3	1
		PCNA	TP53TG1	1
		PCNXL2	H19	1
		PCSK5	MCM3AP-AS1	1
		PCTP	HCP5	1
		PCTP	KTN1-AS1	1
		PCTP	SNHG3	1
		PCYOX1L	MCM3AP-AS1	1
		PCYOX1L	SNHG3	1
		PCYT1A	LINC00467	1
		PDAP1	MCM3AP-AS1	1
		PDCD1LG2	H19	1
		PDCD1LG2	ZNRD1-AS1	1

PDCD4	KTN1-AS1	1
PDCD4	MCM3AP-AS1	1
PDCD4	SNHG3	1
PDCD5	DLEU2	1
PDCD5	TPT1-AS1	1
PDE4A	MCM3AP-AS1	1
PDE4B	H19	1
PDE4B	RUSC1-AS1	1
PDE4B	SNHG5	1
PDE4B	ZNRD1-AS1	1
PDE4DIP	H19	1
PDE4DIP	SNHG5	1
PDE4DIP	ZNRD1-AS1	1
PDE7B	H19	1
PDE7B	ZNRD1-AS1	1
PDE8A	H19	1
PDE8A	SNHG5	1
PDE8A	ZNRD1-AS1	1
PDGFA	KTN1-AS1	1
PDGFA	SNHG3	1
PDGFD	HCP5	1
PDGFRB	DLEU2	1
PDGFRB	H19	1
PDGFRB	TPT1-AS1	1
PDGFRB	ZNRD1-AS1	1
PDIK1L	HCP5	1
PDIK1L	MCM3AP-AS1	1
PDIK1L	SNHG3	1
PDK1	DLEU2	1
PDK1	H19	1
PDK1	RUSC1-AS1	1
PDK1	SNHG5	1
PDK1	TPT1-AS1	1
PDK1	ZNRD1-AS1	1
PDK4	HCP5	1
PDK4	KTN1-AS1	1
PDK4	MCM3AP-AS1	1
PDLIM1	HCP5	1
PDRG1	HCP5	1
PDRG1	MCM3AP-AS1	1
PDSS1	DLEU2	1
PDSS1	H19	1
PDSS1	SNHG5	1
PDSS1	TPT1-AS1	1
PDSS1	ZNRD1-AS1	1
PDXK	HCP5	1
PDXK	MCM3AP-AS1	1
PDXP	KTN1-AS1	1
PEAK1	DLEU2	1
PEAK1	H19	1
PEAK1	SNHG5	1

PEAK1	TPT1-AS1	1
PEAK1	ZNRD1-AS1	1
PEBP1	KTN1-AS1	1
PEBP1	MCM3AP-AS1	1
PECR	DLEU2	1
PERP	SNHG3	1
PEX6	TP53TG1	1
PFKFB3	H19	1
PFKFB3	SNHG5	1
PFKFB3	ZNRD1-AS1	1
PGAP2	HCP5	1
PGAP2	RHPN1-AS1	1
PGBD2	HCP5	1
PGBD2	KTN1-AS1	1
PGBD2	MCM3AP-AS1	1
PGBD2	TP53TG1	1
PGM3	DLEU2	1
PGM3	SNHG5	1
PGM3	TPT1-AS1	1
PGP	HCP5	1
PGP	MCM3AP-AS1	1
PHACTR1	H19	1
PHACTR1	ZNRD1-AS1	1
PHF1	HCP5	1
PHF1	SCARNA9	1
PHF11	DLEU2	1
PHF11	H19	1
PHF11	SNHG5	1
PHF11	ZNRD1-AS1	1
PHF19	KTN1-AS1	1
PHF19	MCM3AP-AS1	1
PHF5A	HCP5	1
PHF5A	MCM3AP-AS1	1
PHF5A	SNHG3	1
PHF6	DLEU2	1
PHF6	H19	1
PHF6	SNHG5	1
PHF6	TPT1-AS1	1
PHF6	ZNRD1-AS1	1
PHGDH	HCP5	1
PHKA1	MCM3AP-AS1	1
PHKA1	SCARNA9	1
PHYH	HCP5	1
PHYH	SNHG3	1
PI4K2B	DLEU2	1
PI4K2B	SNHG5	1
PI4K2B	TPT1-AS1	1
PI4K2B	ZNRD1-AS1	1
PIBF1	KTN1-AS1	1
PIBF1	TP53TG1	1
PICALM	DLEU2	1

		PICALM	H19	1
		PICALM	SNHG5	1
		PICALM	TPT1-AS1	1
		PICALM	ZNRD1-AS1	1
		PIGA	DLEU2	1
		PIGA	H19	1
		PIGA	TPT1-AS1	1
		PIGA	ZNRD1-AS1	1
		PIGM	KTN1-AS1	1
		PIK3C2B	DLEU2	1
		PIK3C2B	H19	1
		PIK3C2B	LINC00467	1
		PIK3C2B	TPT1-AS1	1
		PIK3C2B	ZNRD1-AS1	1
		PIK3CA	H19	1
		PIK3CA	ZNRD1-AS1	1
		PIK3CD	RHPN1-AS1	1
		PIK3CG	SCARNA9	1
		PIK3IP1	MCM3AP-AS1	1
		PIK3R1	HCP5	1
		PIK3R1	KTN1-AS1	1
		PIK3R1	MCM3AP-AS1	1
		PIK3R1	SCARNA9	1
		PIK3R1	TP53TG1	1
		PIK3R5	DLEU2	1
		PIM2	SNHG3	1
		PIP5K1B	HCP5	1
		PIP5K1B	MCM3AP-AS1	1
		PITPNC1	DLEU2	1
		PITPNC1	LINC00467	1
		PITPNC1	SNHG5	1
		PKMYT1	HCP5	1
		PKP2	KTN1-AS1	1
		PKP2	MCM3AP-AS1	1
		PKP4	KTN1-AS1	1
		PKP4	MCM3AP-AS1	1
		PKP4	SCARNA9	1
		PKP4	SNHG3	1
		PLA2G4A	HCP5	1
		PLA2G4A	SNHG3	1
		PLAA	H19	1
		PLAA	SNHG5	1
		PLAA	ZNRD1-AS1	1
		PLAG1	HCP5	1
		PLAG1	KTN1-AS1	1
		PLAG1	MCM3AP-AS1	1
		PLAG1	SCARNA9	1
		PLAT	RUSC1-AS1	1
		PLAUR	DLEU2	1
		PLCL2	MCM3AP-AS1	1
		PLD2	HCP5	1

		PLEC	RUSC1-AS1	1
		PLEK2	H19	1
		PLEKHA1	HCP5	1
		PLEKHA1	KTN1-AS1	1
		PLEKHA1	MCM3AP-AS1	1
		PLEKHA2	KTN1-AS1	1
		PLEKHA2	MCM3AP-AS1	1
		PLEKHA2	SCARNA9	1
		PLEKHA6	H19	1
		PLEKHA6	LINC00467	1
		PLEKHA6	SNHG5	1
		PLEKHA7	MCM3AP-AS1	1
		PLEKHA8	HCP5	1
		PLEKHA8	KTN1-AS1	1
		PLEKHA8	MCM3AP-AS1	1
		PLEKHA8	TP53TG1	1
		PLEKHB1	RHPN1-AS1	1
		PLEKHG3	RUSC1-AS1	1
		PLEKHG3	SNHG5	1
		PLEKHM1	H19	1
		PLEKHM1	LINC00467	1
		PLEKHM1	SNHG5	1
		PLEKHM1	ZNRD1-AS1	1
		PLK1	KTN1-AS1	1
		PLK1	MCM3AP-AS1	1
		PLK2	HCP5	1
		PLK3	HCP5	1
		PLK4	HCP5	1
		PLK4	KTN1-AS1	1
		PLK4	MCM3AP-AS1	1
		PLK4	TP53TG1	1
		PLS1	HCP5	1
		PLS1	MCM3AP-AS1	1
		PLSCR4	H19	1
		PLSCR4	LINC00467	1
		PLSCR4	RUSC1-AS1	1
		PLSCR4	ZNRD1-AS1	1
		PLXNA1	HCP5	1
		PLXNA2	MCM3AP-AS1	1
		PLXNA3	SNHG5	1
		PLXNC1	MCM3AP-AS1	1
		PMEPA1	DLEU2	1
		PMEPA1	H19	1
		PMEPA1	SNHG5	1
		PMEPA1	ZNRD1-AS1	1
		PMM2	SNHG5	1
		PMM2	ZNRD1-AS1	1
		PMP22	HCP5	1
		PMP22	MCM3AP-AS1	1
		PNKD	DLEU2	1
		PNKD	H19	1

PNKD	TPT1-AS1	1
PNKD	ZNRD1-AS1	1
PNP	MCM3AP-AS1	1
PNP	SNHG3	1
PNPLA4	HCP5	1
PNPLA6	MCM3AP-AS1	1
PNPO	MCM3AP-AS1	1
PNPT1	KTN1-AS1	1
PNRC1	H19	1
PNRC1	SNHG5	1
PNRC1	ZNRD1-AS1	1
POFUT2	MCM3AP-AS1	1
POLD3	HCP5	1
POLD3	MCM3AP-AS1	1
POLE2	MCM3AP-AS1	1
POLE3	HCP5	1
POLQ	HCP5	1
POLR2E	MCM3AP-AS1	1
POLR3B	TP53TG1	1
POLR3G	HCP5	1
PPA1	HCP5	1
PPA1	MCM3AP-AS1	1
PPAP2A	MCM3AP-AS1	1
PPAP2A	SCARNA9	1
PPAP2B	HCP5	1
PPAP2B	KTN1-AS1	1
PPAP2B	MCM3AP-AS1	1
PPAP2B	SCARNA9	1
PPAP2B	SNHG3	1
PPAPDC2	HCP5	1
PPAPDC2	MCM3AP-AS1	1
PPARA	H19	1
PPARA	ZNRD1-AS1	1
PPARD	H19	1
PPARD	RUSC1-AS1	1
PPARD	ZNRD1-AS1	1
PPAT	HCP5	1
PPAT	KTN1-AS1	1
PPAT	MCM3AP-AS1	1
PPAT	RHPN1-AS1	1
PPIF	H19	1
PPIF	SNHG5	1
PPIF	ZNRD1-AS1	1
PPIH	HCP5	1
PPIH	KTN1-AS1	1
PPIH	MCM3AP-AS1	1
PPM1G	HCP5	1
PPM1H	MCM3AP-AS1	1
PPM1H	SNHG3	1
PPP1R14A	H19	1
PPP1R14A	ZNRD1-AS1	1

PPP1R3E	KTN1-AS1	1
PPP2R3A	H19	1
PPP2R3A	ZNRD1-AS1	1
PPP2R5C	MCM3AP-AS1	1
PPP2R5C	SNHG3	1
PPRC1	SNHG5	1
PPRC1	ZNRD1-AS1	1
PQLC3	HCP5	1
PRAME	KTN1-AS1	1
PRAME	MCM3AP-AS1	1
PRC1	HCP5	1
PRC1	KTN1-AS1	1
PRC1	MCM3AP-AS1	1
PRC1	SNHG3	1
PRCP	DLEU2	1
PRCP	H19	1
PRCP	TPT1-AS1	1
PRCP	ZNRD1-AS1	1
PRDM1	HCP5	1
PRDM1	KTN1-AS1	1
PRDM1	MCM3AP-AS1	1
PRDM1	SNHG3	1
PRDM2	H19	1
PRDM2	RUSC1-AS1	1
PRDM2	ZNRD1-AS1	1
PRDM5	MCM3AP-AS1	1
PRDM5	SNHG3	1
PRDX3	KTN1-AS1	1
PRDX3	MCM3AP-AS1	1
PREPL	MCM3AP-AS1	1
PREPL	SNHG3	1
PREX1	H19	1
PREX1	ZNRD1-AS1	1
PRICKLE1	SNHG3	1
PRIM1	HCP5	1
PRIM1	SNHG3	1
PRKACB	HCP5	1
PRKACB	KTN1-AS1	1
PRKACB	MCM3AP-AS1	1
PRKACB	SNHG3	1
PRKAR2B	HCP5	1
PRKAR2B	TP53TG1	1
PRKCA	RUSC1-AS1	1
PRKCB	DLEU2	1
PRKCB	H19	1
PRKCB	TPT1-AS1	1
PRKCB	ZNRD1-AS1	1
PRKCH	H19	1
PRKCH	ZNRD1-AS1	1
PRKDC	SNHG3	1
PRKRA	HCP5	1

PRKRA	MCM3AP-AS1	1
PRKRA	SNHG3	1
PRMT2	SNHG5	1
PRMT5	SCARNA9	1
PROS1	MCM3AP-AS1	1
PROS1	SCARNA9	1
PRPF18	DLEU2	1
PRR15	HCP5	1
PRR16	HCP5	1
PRR16	SCARNA9	1
PRR5L	H19	1
PRR5L	SNHG5	1
PRR5L	ZNRD1-AS1	1
PRRG1	HCP5	1
PRRG1	KTN1-AS1	1
PRRG1	SCARNA9	1
PRRG1	SNHG3	1
PRUNE	MCM3AP-AS1	1
PSAT1	HCP5	1
PSAT1	MCM3AP-AS1	1
PSAT1	TP53TG1	1
PSD3	DLEU2	1
PSD3	H19	1
PSD3	LINC00467	1
PSD3	SNHG5	1
PSD3	TPT1-AS1	1
PSD3	ZNRD1-AS1	1
PSEN2	ZNRD1-AS1	1
PSIP1	HCP5	1
PSIP1	KTN1-AS1	1
PSIP1	MCM3AP-AS1	1
PSIP1	TP53TG1	1
PSMA5	MCM3AP-AS1	1
PSMB1	H19	1
PSMB1	LINC00467	1
PSMB2	H19	1
PSMB2	ZNRD1-AS1	1
PSMB3	SNHG3	1
PSMB5	H19	1
PSMB5	LINC00467	1
PSMB5	ZNRD1-AS1	1
PSMC6	DLEU2	1
PSMC6	H19	1
PSMC6	LINC00467	1
PSMC6	SNHG5	1
PSMC6	TPT1-AS1	1
PSMC6	ZNRD1-AS1	1
PSMD1	SNHG5	1
PSMD1	ZNRD1-AS1	1
PSMD11	H19	1
PSMD12	H19	1

PSMD12	RUSC1-AS1	1
PSMD12	ZNRD1-AS1	1
PSMD14	H19	1
PSMD14	SNHG5	1
PSMD14	ZNRD1-AS1	1
PSMD2	H19	1
PSMD2	ZNRD1-AS1	1
PSMD3	RUSC1-AS1	1
PSMD4	ZNRD1-AS1	1
PSMD9	MCM3AP-AS1	1
PSME4	DLEU2	1
PSME4	H19	1
PSME4	LINC00467	1
PSRC1	RHPN1-AS1	1
PSRC1	SCARNA9	1
PTDSS1	HCP5	1
PTDSS1	SCARNA9	1
PTDSS1	SNHG3	1
PTEN	HCP5	1
PTEN	KTN1-AS1	1
PTEN	MCM3AP-AS1	1
PTEN	SNHG3	1
PTGER4	H19	1
PTGER4	SNHG5	1
PTGER4	ZNRD1-AS1	1
PTGFRN	HCP5	1
PTGFRN	KTN1-AS1	1
PTGFRN	MCM3AP-AS1	1
PTGFRN	SNHG3	1
PTGFRN	TP53TG1	1
PTGS2	HCP5	1
PTGS2	MCM3AP-AS1	1
PTHLH	HCP5	1
PTHLH	MCM3AP-AS1	1
PTHLH	TP53TG1	1
PTPDC1	DLEU2	1
PTPDC1	H19	1
PTPDC1	RUSC1-AS1	1
PTPDC1	TPT1-AS1	1
PTPDC1	ZNRD1-AS1	1
PTPLAD2	SNHG5	1
PTPLB	H19	1
PTPLB	ZNRD1-AS1	1
PTPN1	H19	1
PTPN1	LINC00467	1
PTPN12	H19	1
PTPN12	LINC00467	1
PTPN12	RUSC1-AS1	1
PTPN12	SNHG5	1
PTPN12	ZNRD1-AS1	1
PTPN13	DLEU2	1

		PTPN13	H19	1
		PTPN13	LINC00467	1
		PTPN13	RUSC1-AS1	1
		PTPN13	SNHG5	1
		PTPN13	TPT1-AS1	1
		PTPN13	ZNRD1-AS1	1
		PTPN14	H19	1
		PTPN14	SNHG5	1
		PTPN14	ZNRD1-AS1	1
		PTPN18	HCP5	1
		PTPN4	HCP5	1
		PTPN4	MCM3AP-AS1	1
		PTPN9	HCP5	1
		PTPN9	SNHG3	1
		PTPRA	H19	1
		PTPRA	SNHG5	1
		PTPRA	ZNRD1-AS1	1
		PTPRD	HCP5	1
		PTPRD	MCM3AP-AS1	1
		PTPRJ	H19	1
		PTPRJ	SNHG5	1
		PTPRJ	ZNRD1-AS1	1
		PTPRK	HCP5	1
		PTPRK	KTN1-AS1	1
		PTPRK	MCM3AP-AS1	1
		PTPRK	SNHG3	1
		PTPRM	DLEU2	1
		PTPRM	H19	1
		PTPRM	RUSC1-AS1	1
		PTPRM	TPT1-AS1	1
		PTPRM	ZNRD1-AS1	1
		PTTG1	HCP5	1
		PTTG1	SCARNA9	1
		PUS7	HCP5	1
		PVR	H19	1
		PVR	RUSC1-AS1	1
		PVR	ZNRD1-AS1	1
		PVRL2	RUSC1-AS1	1
		PXK	DLEU2	1
		PXK	H19	1
		PXK	TPT1-AS1	1
		PXK	ZNRD1-AS1	1
		PYCRL	MCM3AP-AS1	1
		PYGL	SNHG5	1
		PYGO2	MCM3AP-AS1	1
		PYROXD1	H19	1
		PYROXD1	ZNRD1-AS1	1
		QARS	ZNRD1-AS1	1
		QDPR	HCP5	1
		QDPR	TP53TG1	1
		QSOX2	SCARNA9	1

R3HDM4	H19	1
RAB11FIP1	H19	1
RAB11FIP1	SNHG5	1
RAB11FIP1	TPT1-AS1	1
RAB11FIP1	ZNRD1-AS1	1
RAB11FIP4	HCP5	1
RAB11FIP5	HCP5	1
RAB11FIP5	MCM3AP-AS1	1
RAB11FIP5	SNHG3	1
RAB12	DLEU2	1
RAB12	H19	1
RAB12	LINC00467	1
RAB12	SNHG5	1
RAB12	TPT1-AS1	1
RAB12	ZNRD1-AS1	1
RAB13	KTN1-AS1	1
RAB27B	MCM3AP-AS1	1
RAB2B	LINC00467	1
RAB2B	ZNRD1-AS1	1
RAB30	HCP5	1
RAB30	MCM3AP-AS1	1
RAB31	HCP5	1
RAB3D	SNHG5	1
RAB4B	DLEU2	1
RAB4B	TPT1-AS1	1
RAB4B	ZNRD1-AS1	1
RAB5B	H19	1
RAB5B	RUSC1-AS1	1
RAB5B	SNHG5	1
RAB5B	ZNRD1-AS1	1
RABEPK	RHPN1-AS1	1
RABEPK	SCARNA9	1
RABGAP1	H19	1
RABGAP1	LINC00467	1
RABGAP1	SNHG5	1
RABGAP1	ZNRD1-AS1	1
RABGAP1L	DLEU2	1
RABGAP1L	H19	1
RABGAP1L	SNHG5	1
RABGAP1L	TPT1-AS1	1
RABGAP1L	ZNRD1-AS1	1
RACGAP1	HCP5	1
RACGAP1	MCM3AP-AS1	1
RACGAP1	SNHG3	1
RAD18	HCP5	1
RAD18	SNHG3	1
RAD18	TP53TG1	1
RAD51	MCM3AP-AS1	1
RAD51	SNHG3	1
RAD51AP1	KTN1-AS1	1
RAD51AP1	MCM3AP-AS1	1

		RAD51AP1	SNHG3	1
		RAD51C	SCARNA9	1
		RAD51C	SNHG3	1
		RAE1	MCM3AP-AS1	1
		RAI14	DLEU2	1
		RAI14	H19	1
		RAI14	SNHG5	1
		RAI14	TPT1-AS1	1
		RAI14	ZNRD1-AS1	1
		RALA	H19	1
		RALA	RUSC1-AS1	1
		RALA	SNHG5	1
		RALA	ZNRD1-AS1	1
		RALB	SNHG3	1
		RALGPS1	HCP5	1
		RALGPS2	SCARNA9	1
		RALGPS2	SNHG3	1
		RAN	HCP5	1
		RAN	MCM3AP-AS1	1
		RANBP1	MCM3AP-AS1	1
		RANBP10	SNHG5	1
		RANBP17	HCP5	1
		RANBP17	MCM3AP-AS1	1
		RANBP17	SNHG3	1
		RAP1GAP2	SNHG3	1
		RAPGEF1	H19	1
		RAPGEF1	ZNRD1-AS1	1
		RAPGEF5	ZNRD1-AS1	1
		RAPGEFL1	H19	1
		RAPGEFL1	ZNRD1-AS1	1
		RAPH1	DLEU2	1
		RAPH1	H19	1
		RAPH1	SNHG5	1
		RAPH1	TPT1-AS1	1
		RAPH1	ZNRD1-AS1	1
		RASGEF1A	H19	1
		RASGEF1A	ZNRD1-AS1	1
		RASGEF1B	SNHG5	1
		RASGEF1B	ZNRD1-AS1	1
		RASGRP3	DLEU2	1
		RASGRP3	SNHG5	1
		RASGRP3	TPT1-AS1	1
		RASGRP3	ZNRD1-AS1	1
		RASSF5	H19	1
		RASSF5	RUSC1-AS1	1
		RASSF5	ZNRD1-AS1	1
		RAVER2	HCP5	1
		RAVER2	SNHG3	1
		RBL1	HCP5	1
		RBL1	SNHG3	1
		RBM10	H19	1

		RBM10	SNHG5	1
		RBM34	DLEU2	1
		RBM34	TPT1-AS1	1
		RBM38	MCM3AP-AS1	1
		RBM47	RUSC1-AS1	1
		RBM47	SNHG5	1
		RBM8A	HCP5	1
		RBM8A	MCM3AP-AS1	1
		RBM8A	SNHG3	1
		RBMS3	HCP5	1
		RBMS3	MCM3AP-AS1	1
		RBMS3	TP53TG1	1
		RBPMS	H19	1
		RBPMS	RUSC1-AS1	1
		RBPMS	ZNRD1-AS1	1
		RCAN1	DLEU2	1
		RCAN1	TPT1-AS1	1
		RCAN3	ZNRD1-AS1	1
		RCL1	ZNRD1-AS1	1
		RCOR1	DLEU2	1
		RCOR1	H19	1
		RCOR1	LINC00467	1
		RCOR1	RUSC1-AS1	1
		RCOR1	SNHG5	1
		RCOR1	TPT1-AS1	1
		RCOR1	ZNRD1-AS1	1
		RCSD1	DLEU2	1
		RCSD1	SNHG5	1
		RCSD1	TPT1-AS1	1
		RDH10	MCM3AP-AS1	1
		RDH10	SCARNA9	1
		RDH10	SNHG3	1
		RDH13	MCM3AP-AS1	1
		REL	MCM3AP-AS1	1
		RELB	MCM3AP-AS1	1
		RELB	RHPN1-AS1	1
		RELL1	HCP5	1
		RELL1	SNHG3	1
		REPS2	KTN1-AS1	1
		REPS2	MCM3AP-AS1	1
		RERE	H19	1
		RERE	RUSC1-AS1	1
		RERE	ZNRD1-AS1	1
		REXO1	DLEU2	1
		REXO1	SNHG5	1
		REXO1	ZNRD1-AS1	1
		RFC3	HCP5	1
		RFC3	MCM3AP-AS1	1
		RFC3	SNHG3	1
		RFC5	SNHG3	1
		RFK	HCP5	1

RFK	MCM3AP-AS1	1
RFK	SCARNA9	1
RFWD3	MCM3AP-AS1	1
RFWD3	SNHG3	1
RFX2	DLEU2	1
RFX2	TPT1-AS1	1
RFXAP	HCP5	1
RGL1	DLEU2	1
RGL1	H19	1
RGL1	SNHG5	1
RGL1	TPT1-AS1	1
RGL1	ZNRD1-AS1	1
RGMA	HCP5	1
RGMA	TP53TG1	1
RGS2	DLEU2	1
RGS2	H19	1
RGS2	TPT1-AS1	1
RGS2	ZNRD1-AS1	1
RHOB	DLEU2	1
RHOB	H19	1
RHOB	TPT1-AS1	1
RHOB	ZNRD1-AS1	1
RHOBTB1	HCP5	1
RHOBTB3	HCP5	1
RHOBTB3	MCM3AP-AS1	1
RHOBTB3	SCARNA9	1
RHOU	DLEU2	1
RHOU	SNHG5	1
RHOU	TPT1-AS1	1
RIN2	HCP5	1
RIN2	KTN1-AS1	1
RIN2	MCM3AP-AS1	1
RIN2	SNHG3	1
RIOK3	DLEU2	1
RIOK3	H19	1
RIOK3	TPT1-AS1	1
RNASEL	HCP5	1
RNF115	H19	1
RNF115	SNHG5	1
RNF115	ZNRD1-AS1	1
RNF121	KTN1-AS1	1
RNF145	HCP5	1
RNF145	MCM3AP-AS1	1
RNF150	MCM3AP-AS1	1
RNF150	SNHG3	1
RNF187	HCP5	1
RNF19A	HCP5	1
RNF19A	KTN1-AS1	1
RNF19A	MCM3AP-AS1	1
RNF19A	SCARNA9	1
RNF19A	SNHG3	1

RNF19A	TP53TG1	1
RNF19B	H19	1
RNF213	DLEU2	1
RNF213	TPT1-AS1	1
RNF213	ZNRD1-AS1	1
RNF24	H19	1
RNF24	ZNRD1-AS1	1
RNF44	DLEU2	1
RNF44	H19	1
RNF44	SNHG5	1
RNF44	TPT1-AS1	1
RNF44	ZNRD1-AS1	1
ROBO1	HCP5	1
ROBO1	KTN1-AS1	1
ROBO1	MCM3AP-AS1	1
ROBO1	SCARNA9	1
ROBO1	SNHG3	1
ROBO1	TP53TG1	1
RP9	HCP5	1
RP9	SNHG3	1
RPA1	MCM3AP-AS1	1
RPAP3	MCM3AP-AS1	1
RPAP3	SCARNA9	1
RPAP3	SNHG3	1
RPGRIP1L	HCP5	1
RPGRIP1L	KTN1-AS1	1
RPGRIP1L	RHPN1-AS1	1
RPGRIP1L	TP53TG1	1
RPL13	H19	1
RPL13	SNHG5	1
RPL13	ZNRD1-AS1	1
RPL17	HCP5	1
RPL22	HCP5	1
RPL22	KTN1-AS1	1
RPL22	MCM3AP-AS1	1
RPL32	TP53TG1	1
RPL34	SNHG3	1
RPP25	MCM3AP-AS1	1
RPS14	SNHG5	1
RPS14	ZNRD1-AS1	1
RPS23	LINC00467	1
RPS6KA2	HCP5	1
RPS6KA4	H19	1
RPS6KA4	SNHG5	1
RPS6KA4	ZNRD1-AS1	1
RPS6KA5	HCP5	1
RPS6KA5	KTN1-AS1	1
RPS6KA5	MCM3AP-AS1	1
RPS6KA5	SNHG3	1
RPS6KA6	MCM3AP-AS1	1
RPS6KC1	DLEU2	1

			RPS6KC1	TPT1-AS1	1
			RPSA	H19	1
			RPSA	SNHG5	1
			RPSA	ZNRD1-AS1	1
			RRAGD	HCP5	1
			RRAGD	MCM3AP-AS1	1
			RRAGD	SNHG3	1
			RRAGD	TP53TG1	1
			RRM1	HCP5	1
			RRM1	KTN1-AS1	1
			RRM1	MCM3AP-AS1	1
			RRM2	HCP5	1
			RRM2	MCM3AP-AS1	1
			RRN3	HCP5	1
			RRN3	MCM3AP-AS1	1
			RRP15	KTN1-AS1	1
			RRP15	MCM3AP-AS1	1
			RSBN1	HCP5	1
			RSBN1	MCM3AP-AS1	1
			RSRC2	H19	1
			RSRC2	SNHG5	1
			RTKN2	DLEU2	1
			RTKN2	H19	1
			RTKN2	SNHG5	1
			RTKN2	TPT1-AS1	1
			RTKN2	ZNRD1-AS1	1
			RUNDC3B	DLEU2	1
			RUNDC3B	TPT1-AS1	1
			RUNDC3B	ZNRD1-AS1	1
			RUNX1T1	H19	1
			RUNX1T1	SNHG5	1
			RUNX1T1	ZNRD1-AS1	1
			RUNX2	DLEU2	1
			RUNX2	H19	1
			RUNX2	TPT1-AS1	1
			RUNX2	ZNRD1-AS1	1
			RUNX3	H19	1
			RUNX3	RUSC1-AS1	1
			RUNX3	ZNRD1-AS1	1
			RUVBL1	SCARNA9	1
			RUVBL2	HCP5	1
			RWDD2B	HCP5	1
			S1PR1	H19	1
			S1PR1	SNHG5	1
			S1PR1	ZNRD1-AS1	1
			SAAL1	RUSC1-AS1	1
			SAE1	MCM3AP-AS1	1
			SAE1	SNHG3	1
			SAMD10	H19	1
			SAMD10	ZNRD1-AS1	1
			SAMD12	HCP5	1

SAMD4A	DLEU2	1
SAMD4A	H19	1
SAMD4A	TPT1-AS1	1
SAMD4A	ZNRD1-AS1	1
SAMD4B	RUSC1-AS1	1
SAMD8	DLEU2	1
SAMD8	H19	1
SAMD8	RUSC1-AS1	1
SAMD8	SNHG5	1
SAMD8	TPT1-AS1	1
SAMD8	ZNRD1-AS1	1
SAMD9L	HCP5	1
SAMSN1	KTN1-AS1	1
SAMSN1	MCM3AP-AS1	1
SAP30BP	DLEU2	1
SAP30BP	H19	1
SAP30BP	TPT1-AS1	1
SAP30BP	ZNRD1-AS1	1
SAP30L	SNHG5	1
SARS2	TPT1-AS1	1
SASS6	HCP5	1
SASS6	MCM3AP-AS1	1
SBF2	H19	1
SBF2	LINC00467	1
SBF2	RUSC1-AS1	1
SBF2	ZNRD1-AS1	1
SCAI	HCP5	1
SCAI	MCM3AP-AS1	1
SCAMP5	HCP5	1
SCAMP5	MCM3AP-AS1	1
SCAPER	HCP5	1
SCAPER	MCM3AP-AS1	1
SCAPER	SCARNA9	1
SCAPER	SNHG3	1
SCARB1	MCM3AP-AS1	1
SCARB2	HCP5	1
SCARB2	MCM3AP-AS1	1
SCARB2	RHPN1-AS1	1
SCARB2	SNHG3	1
SCFD1	HCP5	1
SCFD1	SCARNA9	1
SCG5	HCP5	1
SCN4B	HCP5	1
SCP2	HCP5	1
SDC1	H19	1
SDC1	ZNRD1-AS1	1
SDC4	H19	1
SDC4	LINC00467	1
SDC4	ZNRD1-AS1	1
SDCCAG3	MCM3AP-AS1	1
SDCCAG3	TP53TG1	1

SDCCAG8	H19	1
SDCCAG8	RUSC1-AS1	1
SDCCAG8	SNHG5	1
SDCCAG8	ZNRD1-AS1	1
SDF2L1	SCARNA9	1
SEC14L1	H19	1
SEC14L1	RUSC1-AS1	1
SEC14L1	ZNRD1-AS1	1
SEC23B	MCM3AP-AS1	1
SEC23B	SCARNA9	1
SEC24A	DLEU2	1
SEC24A	SNHG5	1
SEC24A	TPT1-AS1	1
SEC24A	ZNRD1-AS1	1
SEC24D	HCP5	1
SEC24D	SNHG3	1
SEC61A2	MCM3AP-AS1	1
SEH1L	HCP5	1
SEH1L	KTN1-AS1	1
SEH1L	MCM3AP-AS1	1
SEH1L	SNHG3	1
SEMA3A	SNHG3	1
SEMA3A	TP53TG1	1
SEMA3C	HCP5	1
SEMA3C	KTN1-AS1	1
SEMA3C	MCM3AP-AS1	1
SEMA3C	SNHG3	1
SEMA6A	HCP5	1
SEMA6A	MCM3AP-AS1	1
SEMA6A	SNHG3	1
SEMA6D	HCP5	1
SEMA6D	KTN1-AS1	1
SEMA6D	MCM3AP-AS1	1
SEMA6D	SNHG3	1
SEMA7A	HCP5	1
SEMA7A	TP53TG1	1
SENP5	DLEU2	1
SENP5	H19	1
SENP5	SNHG5	1
SENP5	TPT1-AS1	1
SENP5	ZNRD1-AS1	1
SEPN1	H19	1
SEPN1	LINC00467	1
10-Sep	MCM3AP-AS1	1
6-Sep	HCP5	1
8-Sep	HCP5	1
8-Sep	SNHG3	1
SERP1	HCP5	1
SERP1	MCM3AP-AS1	1
SERP1	RHPN1-AS1	1
SERP1	SNHG3	1

SERPINB8	HCP5	1
SERPINB8	TP53TG1	1
SERPINE2	MCM3AP-AS1	1
SESN1	HCP5	1
SESN1	MCM3AP-AS1	1
SESN2	HCP5	1
SESN2	KTN1-AS1	1
SESN2	MCM3AP-AS1	1
SESN3	H19	1
SESN3	SNHG5	1
SESN3	ZNRD1-AS1	1
SFMBT1	HCP5	1
SFMBT2	HCP5	1
SFT2D1	HCP5	1
SFXN1	HCP5	1
SFXN1	MCM3AP-AS1	1
SFXN1	SNHG3	1
SFXN2	HCP5	1
SFXN2	MCM3AP-AS1	1
SFXN2	TP53TG1	1
SFXN3	HCP5	1
SGCB	SNHG3	1
SGK1	DLEU2	1
SGK1	H19	1
SGK1	SNHG5	1
SGK1	TPT1-AS1	1
SGK1	ZNRD1-AS1	1
SGK3	DLEU2	1
SGK3	SNHG5	1
SGK3	TPT1-AS1	1
SGK3	ZNRD1-AS1	1
SGMS1	HCP5	1
SGMS1	SNHG3	1
SGMS1	TP53TG1	1
SGMS2	DLEU2	1
SGMS2	H19	1
SGMS2	TPT1-AS1	1
SGMS2	ZNRD1-AS1	1
SGOL2	KTN1-AS1	1
SGPL1	H19	1
SGPL1	ZNRD1-AS1	1
SGSH	ZNRD1-AS1	1
SH2B3	HCP5	1
SH2B3	KTN1-AS1	1
SH2B3	MCM3AP-AS1	1
SH2B3	TP53TG1	1
SH2D2A	H19	1
SH2D2A	ZNRD1-AS1	1
SH2D4A	SNHG3	1
SH3BP4	H19	1
SH3BP4	LINC00467	1

SH3BP4	RUSC1-AS1	1
SH3BP4	SNHG5	1
SH3BP4	ZNRD1-AS1	1
SH3BP5	H19	1
SH3BP5	RUSC1-AS1	1
SH3BP5	SNHG5	1
SH3BP5	ZNRD1-AS1	1
SH3D19	HCP5	1
SH3GL1	DLEU2	1
SH3GL1	TPT1-AS1	1
SH3GL3	HCP5	1
SH3RF1	HCP5	1
SH3RF3	H19	1
SHCBP1	HCP5	1
SHCBP1	KTN1-AS1	1
SHCBP1	MCM3AP-AS1	1
SHCBP1	SNHG3	1
SHCBP1	TP53TG1	1
SHISA5	HCP5	1
SHISA5	KTN1-AS1	1
SHISA5	TP53TG1	1
SHPK	SNHG3	1
SIDT1	SNHG5	1
SIK1	HCP5	1
SIK1	MCM3AP-AS1	1
SIK1	TP53TG1	1
SIPA1L1	LINC00467	1
SIVA1	MCM3AP-AS1	1
SIX4	DLEU2	1
SIX4	H19	1
SIX4	LINC00467	1
SIX4	TPT1-AS1	1
SIX4	ZNRD1-AS1	1
SKA1	MCM3AP-AS1	1
SKA1	RHPN1-AS1	1
SKA2	KTN1-AS1	1
SKA3	HCP5	1
SKAP2	MCM3AP-AS1	1
SKIL	H19	1
SKIL	SNHG5	1
SKIL	ZNRD1-AS1	1
SLA	LINC00467	1
SLA	ZNRD1-AS1	1
SLAIN1	HCP5	1
SLAIN1	MCM3AP-AS1	1
SLAIN1	SNHG3	1
SLAIN1	TP53TG1	1
SLAMF1	RUSC1-AS1	1
SLAMF7	RUSC1-AS1	1
SLAMF7	SNHG5	1
SLBP	KTN1-AS1	1

SLBP	MCM3AP-AS1	1
SLC11A2	H19	1
SLC11A2	SNHG5	1
SLC11A2	ZNRD1-AS1	1
SLC12A4	SNHG5	1
SLC12A9	ZNRD1-AS1	1
SLC15A3	ZNRD1-AS1	1
SLC16A1	HCP5	1
SLC16A1	MCM3AP-AS1	1
SLC16A1	SCARNA9	1
SLC16A1	TP53TG1	1
SLC16A10	H19	1
SLC16A10	ZNRD1-AS1	1
SLC16A9	HCP5	1
SLC16A9	KTN1-AS1	1
SLC16A9	MCM3AP-AS1	1
SLC16A9	TP53TG1	1
SLC1A5	HCP5	1
SLC20A2	HCP5	1
SLC20A2	MCM3AP-AS1	1
SLC20A2	SNHG3	1
SLC24A1	ZNRD1-AS1	1
SLC25A15	HCP5	1
SLC25A15	RHPN1-AS1	1
SLC25A24	DLEU2	1
SLC25A24	H19	1
SLC25A24	RUSC1-AS1	1
SLC25A24	SNHG5	1
SLC25A24	ZNRD1-AS1	1
SLC25A25	HCP5	1
SLC25A25	TP53TG1	1
SLC25A33	HCP5	1
SLC25A45	SNHG5	1
SLC26A11	DLEU2	1
SLC26A11	TPT1-AS1	1
SLC29A1	MCM3AP-AS1	1
SLC2A13	MCM3AP-AS1	1
SLC2A13	RHPN1-AS1	1
SLC2A4RG	H19	1
SLC2A4RG	ZNRD1-AS1	1
SLC30A4	MCM3AP-AS1	1
SLC30A7	H19	1
SLC30A7	SNHG5	1
SLC30A7	ZNRD1-AS1	1
SLC30A9	HCP5	1
SLC30A9	KTN1-AS1	1
SLC30A9	MCM3AP-AS1	1
SLC30A9	TP53TG1	1
SLC31A1	HCP5	1
SLC31A1	MCM3AP-AS1	1
SLC31A1	SNHG3	1

SLC35B4	KTN1-AS1	1
SLC35B4	MCM3AP-AS1	1
SLC35D2	H19	1
SLC35D2	LINC00467	1
SLC35E1	H19	1
SLC35E1	LINC00467	1
SLC35E1	ZNRD1-AS1	1
SLC35F1	HCP5	1
SLC35F1	SNHG3	1
SLC37A1	HCP5	1
SLC37A1	MCM3AP-AS1	1
SLC37A4	MCM3AP-AS1	1
SLC38A1	DLEU2	1
SLC38A1	H19	1
SLC38A1	LINC00467	1
SLC38A1	SNHG5	1
SLC38A1	TPT1-AS1	1
SLC38A1	ZNRD1-AS1	1
SLC39A11	HCP5	1
SLC39A13	H19	1
SLC39A14	KTN1-AS1	1
SLC39A14	MCM3AP-AS1	1
SLC39A14	SCARNA9	1
SLC39A14	SNHG3	1
SLC39A14	TP53TG1	1
SLC39A7	HCP5	1
SLC40A1	HCP5	1
SLC41A2	DLEU2	1
SLC41A2	TPT1-AS1	1
SLC41A2	ZNRD1-AS1	1
SLC44A5	KTN1-AS1	1
SLC44A5	MCM3AP-AS1	1
SLC44A5	SNHG3	1
SLC45A4	HCP5	1
SLC46A1	KTN1-AS1	1
SLC46A1	MCM3AP-AS1	1
SLC46A3	HCP5	1
SLC46A3	MCM3AP-AS1	1
SLC46A3	SCARNA9	1
SLC48A1	HCP5	1
SLC4A7	DLEU2	1
SLC4A7	H19	1
SLC4A7	RUSC1-AS1	1
SLC4A7	SNHG5	1
SLC4A7	TPT1-AS1	1
SLC4A7	ZNRD1-AS1	1
SLC5A3	HCP5	1
SLC5A3	KTN1-AS1	1
SLC5A3	MCM3AP-AS1	1
SLC5A3	SNHG3	1
SLC5A6	SCARNA9	1

		SLC6A9	HCP5	1
		SLC7A1	KTN1-AS1	1
		SLC7A1	MCM3AP-AS1	1
		SLC7A2	H19	1
		SLC7A2	ZNRD1-AS1	1
		SLC7A5	DLEU2	1
		SLC7A5	TPT1-AS1	1
		SLC9A8	DLEU2	1
		SLC9A8	TPT1-AS1	1
		SLC9A8	ZNRD1-AS1	1
		SLCO5A1	H19	1
		SLCO5A1	ZNRD1-AS1	1
		SLFN12	SCARNA9	1
		SLTM	DLEU2	1
		SLTM	H19	1
		SLTM	SNHG5	1
		SLTM	TPT1-AS1	1
		SLTM	ZNRD1-AS1	1
		SMAD1	DLEU2	1
		SMAD1	H19	1
		SMAD1	SNHG5	1
		SMAD1	TPT1-AS1	1
		SMAD1	ZNRD1-AS1	1
		SMAD3	H19	1
		SMAD3	RUSC1-AS1	1
		SMAD3	ZNRD1-AS1	1
		SMAD5	HCP5	1
		SMAD5	KTN1-AS1	1
		SMAD5	MCM3AP-AS1	1
		SMAD5	SNHG3	1
		SMAD6	H19	1
		SMAD6	RUSC1-AS1	1
		SMAD6	SNHG5	1
		SMAD6	ZNRD1-AS1	1
		SMAD7	H19	1
		SMAD7	SNHG5	1
		SMAD7	ZNRD1-AS1	1
		SMARCA1	HCP5	1
		SMARCA1	KTN1-AS1	1
		SMARCA1	MCM3AP-AS1	1
		SMARCA1	SCARNA9	1
		SMARCA1	SNHG3	1
		SMARCA1	TP53TG1	1
		SMARCA2	HCP5	1
		SMARCA2	SCARNA9	1
		SMC1A	SNHG3	1
		SMC2	HCP5	1
		SMC2	MCM3AP-AS1	1
		SMC4	HCP5	1
		SMC4	SNHG3	1
		SMG5	ZNRD1-AS1	1

SMO	SNHG3	1
SMURF1	HCP5	1
SMURF1	MCM3AP-AS1	1
SMYD2	KTN1-AS1	1
SMYD2	MCM3AP-AS1	1
SNAI1	DLEU2	1
SNAI1	TPT1-AS1	1
SNAPC1	HCP5	1
SNRNP40	HCP5	1
SNRNP40	KTN1-AS1	1
SNRNP40	SNHG3	1
SNRPC	H19	1
SNRPC	ZNRD1-AS1	1
SNRPF	SNHG3	1
SNX11	HCP5	1
SNX11	MCM3AP-AS1	1
SNX11	TP53TG1	1
SNX25	HCP5	1
SNX25	KTN1-AS1	1
SNX25	SNHG3	1
SNX25	TP53TG1	1
SNX29	DLEU2	1
SNX29	TPT1-AS1	1
SNX4	RUSC1-AS1	1
SNX4	ZNRD1-AS1	1
SNX7	HCP5	1
SNX9	DLEU2	1
SNX9	H19	1
SNX9	RUSC1-AS1	1
SNX9	TPT1-AS1	1
SNX9	ZNRD1-AS1	1
SOBP	HCP5	1
SOBP	MCM3AP-AS1	1
SOCS1	MCM3AP-AS1	1
SOCS3	DLEU2	1
SOCS3	H19	1
SOCS3	TPT1-AS1	1
SOCS3	ZNRD1-AS1	1
SOCS5	HCP5	1
SOCS5	KTN1-AS1	1
SOCS5	MCM3AP-AS1	1
SOCS5	TP53TG1	1
SORL1	HCP5	1
SORL1	SNHG3	1
SOX5	H19	1
SOX5	ZNRD1-AS1	1
SP1	DLEU2	1
SP1	H19	1
SP1	SNHG5	1
SP1	ZNRD1-AS1	1
SPAG17	H19	1

		SPAG17	ZNRD1-AS1	1
		SPAG5	SNHG3	1
		SPAG7	SNHG5	1
		SPAG7	ZNRD1-AS1	1
		SPAG9	DLEU2	1
		SPAG9	H19	1
		SPAG9	RUSC1-AS1	1
		SPAG9	TPT1-AS1	1
		SPATS2	HCP5	1
		SPATS2	KTN1-AS1	1
		SPATS2	MCM3AP-AS1	1
		SPEF2	HCP5	1
		SPEF2	SNHG3	1
		SPG21	KTN1-AS1	1
		SPG21	SCARNA9	1
		SPIN4	KTN1-AS1	1
		SPIN4	MCM3AP-AS1	1
		SPIN4	TP53TG1	1
		SPIRE1	DLEU2	1
		SPIRE1	H19	1
		SPIRE1	TPT1-AS1	1
		SPIRE1	ZNRD1-AS1	1
		SPOCK3	ZNRD1-AS1	1
		SPPL2B	HCP5	1
		SPRED1	H19	1
		SPRED1	LINC00467	1
		SPRED1	SNHG5	1
		SPRED1	ZNRD1-AS1	1
		SPRY4	H19	1
		SPRY4	ZNRD1-AS1	1
		SPRYD3	HCP5	1
		SPRYD3	MCM3AP-AS1	1
		SPRYD3	SNHG3	1
		SPSB4	H19	1
		SPSB4	ZNRD1-AS1	1
		SPTBN1	H19	1
		SPTBN1	SNHG5	1
		SPTBN1	ZNRD1-AS1	1
		SPTLC2	HCP5	1
		SPTLC2	MCM3AP-AS1	1
		SPTLC2	SNHG3	1
		SPTY2D1	HCP5	1
		SPTY2D1	KTN1-AS1	1
		SPTY2D1	MCM3AP-AS1	1
		SPTY2D1	RHPN1-AS1	1
		SPTY2D1	SNHG3	1
		SRF	SNHG5	1
		SRF	ZNRD1-AS1	1
		SRGAP1	H19	1
		SRGAP1	SNHG5	1
		SRGAP1	ZNRD1-AS1	1

		SRGAP2	DLEU2	1
		SRGAP2	H19	1
		SRGAP2	TPT1-AS1	1
		SRGAP2	ZNRD1-AS1	1
		SRP19	HCP5	1
		SRP19	TP53TG1	1
		SRPRB	MCM3AP-AS1	1
		SRRT	HCP5	1
		SRRT	SCARNA9	1
		SRRT	SNHG3	1
		SRSF1	HCP5	1
		SRSF1	MCM3AP-AS1	1
		SRSF10	HCP5	1
		SRSF12	H19	1
		SRSF12	ZNRD1-AS1	1
		SSBP2	DLEU2	1
		SSBP2	RUSC1-AS1	1
		SSBP2	SNHG5	1
		SSBP2	TPT1-AS1	1
		SSH1	DLEU2	1
		SSH1	H19	1
		SSH1	TPT1-AS1	1
		SSH1	ZNRD1-AS1	1
		SSTR2	DLEU2	1
		SSTR2	H19	1
		SSTR2	TPT1-AS1	1
		SSTR2	ZNRD1-AS1	1
		SSU72	H19	1
		SSU72	ZNRD1-AS1	1
		ST3GAL4	SCARNA9	1
		ST3GAL4	SNHG3	1
		ST3GAL5	DLEU2	1
		ST3GAL5	TPT1-AS1	1
		ST8SIA4	HCP5	1
		STARD4	HCP5	1
		STARD4	SCARNA9	1
		STARD8	SCARNA9	1
		STAT6	H19	1
		STAT6	SNHG5	1
		STC2	HCP5	1
		STC2	MCM3AP-AS1	1
		STEAP2	HCP5	1
		STEAP2	KTN1-AS1	1
		STEAP2	MCM3AP-AS1	1
		STEAP2	SNHG3	1
		STEAP3	HCP5	1
		STEAP3	MCM3AP-AS1	1
		STIL	HCP5	1
		STK17A	HCP5	1
		STK17A	MCM3AP-AS1	1
		STK17B	DLEU2	1

STK17B	H19	1
STK17B	TPT1-AS1	1
STK17B	ZNRD1-AS1	1
STK24	HCP5	1
STK24	KTN1-AS1	1
STK24	MCM3AP-AS1	1
STK24	SNHG3	1
STK32B	H19	1
STK32B	ZNRD1-AS1	1
STK38L	DLEU2	1
STK38L	H19	1
STK38L	RUSC1-AS1	1
STK38L	SNHG5	1
STK38L	TPT1-AS1	1
STK38L	ZNRD1-AS1	1
STK39	HCP5	1
STK39	MCM3AP-AS1	1
STK39	SNHG3	1
STMN1	HCP5	1
STMN1	MCM3AP-AS1	1
STOM	H19	1
STOM	SNHG5	1
STOM	ZNRD1-AS1	1
STOX1	HCP5	1
STOX1	RHPN1-AS1	1
STRADB	HCP5	1
STRADB	MCM3AP-AS1	1
STRADB	SNHG3	1
STRAP	MCM3AP-AS1	1
STRAP	SNHG3	1
STX11	TP53TG1	1
STX7	DLEU2	1
STX7	H19	1
STX7	RUSC1-AS1	1
STX7	TPT1-AS1	1
STX7	ZNRD1-AS1	1
STYK1	HCP5	1
SUB1	H19	1
SUB1	ZNRD1-AS1	1
SUFU	HCP5	1
SUFU	SNHG3	1
SULF2	HCP5	1
SULF2	KTN1-AS1	1
SULF2	MCM3AP-AS1	1
SULF2	SNHG3	1
SUMO1	H19	1
SUMO1	LINC00467	1
SUN2	H19	1
SUN2	ZNRD1-AS1	1
SUOX	SNHG3	1
SUSD1	HCP5	1

SUSD3	SNHG5	1
SUV39H1	SNHG3	1
SUV39H2	HCP5	1
SUV39H2	SNHG3	1
SVIL	H19	1
SVIL	RUSC1-AS1	1
SVIL	ZNRD1-AS1	1
SVIP	TP53TG1	1
SYAP1	HCP5	1
SYAP1	SNHG3	1
SYBU	HCP5	1
SYBU	KTN1-AS1	1
SYBU	MCM3AP-AS1	1
SYDE2	H19	1
SYDE2	SNHG5	1
SYDE2	ZNRD1-AS1	1
SYNGR1	MCM3AP-AS1	1
SYP	DLEU2	1
SYPL1	KTN1-AS1	1
SYPL1	MCM3AP-AS1	1
SYPL1	SCARNA9	1
SYPL1	SNHG3	1
SYT1	H19	1
SYT1	SNHG5	1
SYT1	ZNRD1-AS1	1
SYT11	MCM3AP-AS1	1
TAB2	HCP5	1
TAB2	KTN1-AS1	1
TAB2	MCM3AP-AS1	1
TAB2	SCARNA9	1
TAB2	SNHG3	1
TAB2	TP53TG1	1
TAB3	HCP5	1
TAB3	KTN1-AS1	1
TAB3	MCM3AP-AS1	1
TAB3	SNHG3	1
TACC1	DLEU2	1
TACC1	H19	1
TACC1	SNHG5	1
TACC1	TPT1-AS1	1
TACC1	ZNRD1-AS1	1
TACC3	RHPN1-AS1	1
TAF13	RUSC1-AS1	1
TAF13	SNHG5	1
TAF13	ZNRD1-AS1	1
TAF5	HCP5	1
TAF5	MCM3AP-AS1	1
TAGAP	HCP5	1
TANK	DLEU2	1
TANK	H19	1
TANK	RUSC1-AS1	1

TANK	SNHG5	1
TANK	TPT1-AS1	1
TANK	ZNRD1-AS1	1
TARBP1	MCM3AP-AS1	1
TARBP2	HCP5	1
TARBP2	MCM3AP-AS1	1
TARS	KTN1-AS1	1
TARS	MCM3AP-AS1	1
TARS	TP53TG1	1
TBC1D12	HCP5	1
TBC1D12	KTN1-AS1	1
TBC1D12	SNHG3	1
TBC1D12	TP53TG1	1
TBC1D14	MCM3AP-AS1	1
TBC1D14	TP53TG1	1
TBC1D16	SNHG5	1
TBC1D17	HCP5	1
TBC1D2	H19	1
TBC1D2	ZNRD1-AS1	1
TBC1D2B	DLEU2	1
TBC1D2B	TPT1-AS1	1
TBC1D4	DLEU2	1
TBC1D4	RUSC1-AS1	1
TBC1D4	SNHG5	1
TBC1D4	ZNRD1-AS1	1
TBC1D8	HCP5	1
TBC1D8	MCM3AP-AS1	1
TBC1D8	TP53TG1	1
TBC1D9	H19	1
TBC1D9	SNHG5	1
TBC1D9	ZNRD1-AS1	1
TBCEL	DLEU2	1
TBCEL	H19	1
TBCEL	LINC00467	1
TBCEL	TPT1-AS1	1
TBCEL	ZNRD1-AS1	1
TBPL1	HCP5	1
TBPL1	MCM3AP-AS1	1
TBPL1	SCARNA9	1
TBX19	H19	1
TBX19	ZNRD1-AS1	1
TCEAL1	HCP5	1
TCEAL7	LINC00467	1
TCEAL7	SNHG5	1
TCEB3	DLEU2	1
TCEB3	H19	1
TCEB3	SNHG5	1
TCEB3	ZNRD1-AS1	1
TCERG1	RUSC1-AS1	1
TCERG1	SNHG5	1
TCERG1	ZNRD1-AS1	1

TCF19	KTN1-AS1	1
TCF19	SNHG3	1
TCF4	DLEU2	1
TCF4	H19	1
TCF4	RUSC1-AS1	1
TCF4	SNHG5	1
TCF4	TPT1-AS1	1
TCF4	ZNRD1-AS1	1
TCF7	HCP5	1
TCF7L2	DLEU2	1
TCF7L2	H19	1
TCF7L2	LINC00467	1
TCF7L2	RUSC1-AS1	1
TCF7L2	SNHG5	1
TCF7L2	TPT1-AS1	1
TCF7L2	ZNRD1-AS1	1
TCP11L2	KTN1-AS1	1
TCTEX1D2	HCP5	1
TCTEX1D2	MCM3AP-AS1	1
TCTN1	MCM3AP-AS1	1
TDG	DLEU2	1
TDG	H19	1
TDG	TPT1-AS1	1
TDRD7	DLEU2	1
TDRD7	SNHG5	1
TDRD7	TPT1-AS1	1
TET1	HCP5	1
TET3	DLEU2	1
TET3	H19	1
TET3	SNHG5	1
TET3	TPT1-AS1	1
TET3	ZNRD1-AS1	1
TEX2	HCP5	1
TEX2	MCM3AP-AS1	1
TEX2	SNHG3	1
TEX30	SNHG5	1
TFB1M	KTN1-AS1	1
TFB1M	MCM3AP-AS1	1
TFB2M	HCP5	1
TFB2M	SNHG3	1
TFB2M	TP53TG1	1
TFCP2L1	H19	1
TFCP2L1	ZNRD1-AS1	1
TFDP1	SNHG3	1
TFEC	DLEU2	1
TFPI	HCP5	1
TFPI	MCM3AP-AS1	1
TFPI	RHPN1-AS1	1
TFPI	TP53TG1	1
TFPI2	HCP5	1
TFPI2	KTN1-AS1	1

TFPI2	MCM3AP-AS1	1
TFR2	MCM3AP-AS1	1
TFRC	HCP5	1
TFRC	MCM3AP-AS1	1
TFRC	RHPN1-AS1	1
TFRC	SNHG3	1
TGFB1I1	H19	1
TGFB1I1	ZNRD1-AS1	1
TGFBR2	HCP5	1
TGFBR2	KTN1-AS1	1
TGFBR2	MCM3AP-AS1	1
TGFBR2	SNHG3	1
TGM2	H19	1
TGM2	ZNRD1-AS1	1
THAP11	HCP5	1
THAP11	SCARNA9	1
THAP11	SNHG3	1
THAP2	MCM3AP-AS1	1
THBS1	H19	1
THBS1	LINC00467	1
THBS1	ZNRD1-AS1	1
THEM4	H19	1
THEM4	SNHG5	1
THEM4	ZNRD1-AS1	1
THOP1	MCM3AP-AS1	1
THRA	HCP5	1
THY1	HCP5	1
TIAM1	HCP5	1
TIAM1	SCARNA9	1
TIAM1	SNHG3	1
TIMM10	SCARNA9	1
TIMM17A	HCP5	1
TIMM22	MCM3AP-AS1	1
TIMM8B	HCP5	1
TIMP2	DLEU2	1
TIMP2	H19	1
TIMP2	RUSC1-AS1	1
TIMP2	TPT1-AS1	1
TIMP2	ZNRD1-AS1	1
TIMP3	HCP5	1
TIPIN	HCP5	1
TJP2	KTN1-AS1	1
TJP2	MCM3AP-AS1	1
TJP2	SNHG3	1
TLE1	DLEU2	1
TLE1	H19	1
TLE1	SNHG5	1
TLE1	TPT1-AS1	1
TLE1	ZNRD1-AS1	1
TLE4	DLEU2	1
TLE4	H19	1

TLE4	SNHG5	1
TLE4	TPT1-AS1	1
TLE4	ZNRD1-AS1	1
TLN1	RUSC1-AS1	1
TM4SF1	MCM3AP-AS1	1
TM7SF3	MCM3AP-AS1	1
TMC7	HCP5	1
TMC7	MCM3AP-AS1	1
TMC7	SNHG3	1
TMCC3	H19	1
TMCC3	SNHG5	1
TMCC3	ZNRD1-AS1	1
TMED4	MCM3AP-AS1	1
TMED8	RHPN1-AS1	1
TMED9	HCP5	1
TMED9	MCM3AP-AS1	1
TMEFF1	DLEU2	1
TMEFF1	H19	1
TMEFF1	TPT1-AS1	1
TMEFF1	ZNRD1-AS1	1
TMEM106B	MCM3AP-AS1	1
TMEM106B	RHPN1-AS1	1
TMEM109	KTN1-AS1	1
TMEM109	MCM3AP-AS1	1
TMEM110	RHPN1-AS1	1
TMEM134	HCP5	1
TMEM136	MCM3AP-AS1	1
TMEM141	HCP5	1
TMEM161B	HCP5	1
TMEM161B	MCM3AP-AS1	1
TMEM161B	SNHG3	1
TMEM165	DLEU2	1
TMEM165	TPT1-AS1	1
TMEM165	ZNRD1-AS1	1
TMEM167A	HCP5	1
TMEM167A	MCM3AP-AS1	1
TMEM187	SNHG3	1
TMEM19	HCP5	1
TMEM19	SNHG3	1
TMEM19	TP53TG1	1
TMEM194A	HCP5	1
TMEM194A	KTN1-AS1	1
TMEM194A	MCM3AP-AS1	1
TMEM194A	SNHG3	1
TMEM194B	ZNRD1-AS1	1
TMEM2	H19	1
TMEM2	LINC00467	1
TMEM2	SNHG5	1
TMEM2	ZNRD1-AS1	1
TMEM201	HCP5	1
TMEM201	MCM3AP-AS1	1

TMEM242	H19	1
TMEM242	ZNRD1-AS1	1
TMEM25	H19	1
TMEM25	LINC00467	1
TMEM25	ZNRD1-AS1	1
TMEM38B	HCP5	1
TMEM38B	KTN1-AS1	1
TMEM38B	MCM3AP-AS1	1
TMEM38B	RHPN1-AS1	1
TMEM38B	SCARNA9	1
TMEM38B	SNHG3	1
TMEM39A	MCM3AP-AS1	1
TMEM39B	HCP5	1
TMEM39B	KTN1-AS1	1
TMEM39B	MCM3AP-AS1	1
TMEM45A	HCP5	1
TMEM45A	MCM3AP-AS1	1
TMEM45A	SCARNA9	1
TMEM50B	H19	1
TMEM50B	SNHG5	1
TMEM50B	ZNRD1-AS1	1
TMEM57	HCP5	1
TMEM57	MCM3AP-AS1	1
TMEM57	SCARNA9	1
TMEM63B	H19	1
TMEM63B	SNHG5	1
TMEM63B	ZNRD1-AS1	1
TMEM65	H19	1
TMEM65	ZNRD1-AS1	1
TMEM80	H19	1
TMEM87B	DLEU2	1
TMEM87B	SNHG5	1
TMEM87B	TPT1-AS1	1
TMEM87B	ZNRD1-AS1	1
TMEM9	RHPN1-AS1	1
TMEM9	SCARNA9	1
TMEM97	HCP5	1
TMEM97	KTN1-AS1	1
TMEM97	RHPN1-AS1	1
TMPO	H19	1
TMPO	LINC00467	1
TMPO	SNHG5	1
TMPO	ZNRD1-AS1	1
TMTC1	H19	1
TMTC1	ZNRD1-AS1	1
TMTC4	HCP5	1
TMTC4	KTN1-AS1	1
TMTC4	TP53TG1	1
TNFAIP1	H19	1
TNFAIP1	ZNRD1-AS1	1
TNFAIP2	ZNRD1-AS1	1

		TNFAIP3	H19	1
		TNFAIP3	LINC00467	1
		TNFAIP3	RUSC1-AS1	1
		TNFAIP3	ZNRD1-AS1	1
		TNFAIP8	H19	1
		TNFAIP8	ZNRD1-AS1	1
		TNFRSF10B	HCP5	1
		TNFRSF10B	MCM3AP-AS1	1
		TNFRSF10D	HCP5	1
		TNFRSF10D	SCARNA9	1
		TNFRSF10D	SNHG3	1
		TNFRSF1B	H19	1
		TNFRSF1B	ZNRD1-AS1	1
		TNFRSF8	HCP5	1
		TNFSF10	H19	1
		TNFSF10	SNHG5	1
		TNFSF10	ZNRD1-AS1	1
		TNFSF11	H19	1
		TNFSF11	ZNRD1-AS1	1
		TNFSF12	HCP5	1
		TNFSF12	SNHG3	1
		TNFSF15	HCP5	1
		TNFSF9	DLEU2	1
		TNFSF9	TPT1-AS1	1
		TNFSF9	ZNRD1-AS1	1
		TNIK	HCP5	1
		TNIP1	DLEU2	1
		TNIP1	TPT1-AS1	1
		TNIP1	ZNRD1-AS1	1
		TNIP2	H19	1
		TNIP2	ZNRD1-AS1	1
		TNS3	HCP5	1
		TNS3	MCM3AP-AS1	1
		TOB1	HCP5	1
		TOB1	SNHG3	1
		TOM1L2	H19	1
		TOM1L2	ZNRD1-AS1	1
		TOMM22	SNHG5	1
		TOMM22	ZNRD1-AS1	1
		TOMM34	MCM3AP-AS1	1
		TOMM34	SNHG3	1
		TOP2A	HCP5	1
		TOP2A	KTN1-AS1	1
		TOP2A	MCM3AP-AS1	1
		TOP2A	SNHG3	1
		TOPBP1	HCP5	1
		TOPBP1	KTN1-AS1	1
		TOPBP1	MCM3AP-AS1	1
		TOPBP1	SCARNA9	1
		TOR1A	KTN1-AS1	1
		TOR1A	MCM3AP-AS1	1

		TOR1A	RHPN1-AS1	1
		TOR1A	SNHG3	1
		TP53	HCP5	1
		TP53BP2	DLEU2	1
		TP53BP2	SNHG5	1
		TP53INP1	DLEU2	1
		TP53INP1	H19	1
		TP53INP1	SNHG5	1
		TP53INP1	TPT1-AS1	1
		TP53INP1	ZNRD1-AS1	1
		TPCN1	KTN1-AS1	1
		TPCN1	SCARNA9	1
		TPP1	HCP5	1
		TPP1	MCM3AP-AS1	1
		TPX2	HCP5	1
		TRAF3IP2	DLEU2	1
		TRAF3IP2	H19	1
		TRAF3IP2	TPT1-AS1	1
		TRAF4	HCP5	1
		TRAF4	MCM3AP-AS1	1
		TRAPPC8	DLEU2	1
		TRAPPC8	H19	1
		TRAPPC8	LINC00467	1
		TRERF1	KTN1-AS1	1
		TRERF1	MCM3AP-AS1	1
		TRERF1	SNHG3	1
		TRIAP1	HCP5	1
		TRIAP1	MCM3AP-AS1	1
		TRIAP1	SNHG3	1
		TRIB1	H19	1
		TRIB1	LINC00467	1
		TRIB1	SNHG5	1
		TRIB1	ZNRD1-AS1	1
		TRIB2	H19	1
		TRIB2	LINC00467	1
		TRIB2	SNHG5	1
		TRIB2	ZNRD1-AS1	1
		TRIM14	MCM3AP-AS1	1
		TRIM14	SNHG3	1
		TRIM16	HCP5	1
		TRIM16	SNHG3	1
		TRIM36	HCP5	1
		TRIM36	MCM3AP-AS1	1
		TRIM36	SCARNA9	1
		TRIM36	SNHG3	1
		TRIM4	KTN1-AS1	1
		TRIM4	SNHG3	1
		TRIM59	KTN1-AS1	1
		TRIM59	MCM3AP-AS1	1
		TRIM59	SNHG3	1
		TRIM65	HCP5	1

TRIM65	MCM3AP-AS1	1
TRIO	RUSC1-AS1	1
TRIO	SNHG5	1
TRIO	ZNRD1-AS1	1
TRIP10	H19	1
TRIP10	RUSC1-AS1	1
TRIP10	ZNRD1-AS1	1
TRIP13	HCP5	1
TRIP13	KTN1-AS1	1
TRIP13	SNHG3	1
TRIT1	MCM3AP-AS1	1
TRMT6	HCP5	1
TROAP	RHPN1-AS1	1
TRUB1	HCP5	1
TRUB1	KTN1-AS1	1
TRUB1	MCM3AP-AS1	1
TRUB1	SCARNA9	1
TSEN15	MCM3AP-AS1	1
TSEN15	SNHG3	1
TSHZ1	HCP5	1
TSHZ1	KTN1-AS1	1
TSHZ1	MCM3AP-AS1	1
TSHZ1	TP53TG1	1
TSHZ3	HCP5	1
TSHZ3	KTN1-AS1	1
TSHZ3	MCM3AP-AS1	1
TSKU	HCP5	1
TSKU	TP53TG1	1
TSPAN14	H19	1
TSPAN14	RUSC1-AS1	1
TSPAN14	ZNRD1-AS1	1
TSPAN2	SCARNA9	1
TSPAN7	ZNRD1-AS1	1
TSPO	MCM3AP-AS1	1
TSPYL1	HCP5	1
TSR1	HCP5	1
TSR1	MCM3AP-AS1	1
TSR1	SNHG3	1
TTC28	SNHG3	1
TTC28	TP53TG1	1
TTC30B	HCP5	1
TTC30B	KTN1-AS1	1
TTC9	HCP5	1
TTC9	KTN1-AS1	1
TTC9	MCM3AP-AS1	1
TTK	SNHG3	1
TTLL7	DLEU2	1
TTLL7	H19	1
TTLL7	LINC00467	1
TTLL7	TPT1-AS1	1
TTLL7	ZNRD1-AS1	1

TTYH3	HCP5	1
TTYH3	SNHG3	1
TUBB6	RHPN1-AS1	1
TUBB6	SCARNA9	1
TUBD1	SCARNA9	1
TUBE1	RHPN1-AS1	1
TUBGCP3	HCP5	1
TUBGCP4	KTN1-AS1	1
TUBGCP4	MCM3AP-AS1	1
TUBGCP4	SNHG3	1
TUBGCP5	DLEU2	1
TUBGCP5	SNHG5	1
TUBGCP5	TPT1-AS1	1
TUFM	HCP5	1
TXLNB	DLEU2	1
TXN	SNHG3	1
TXNDC9	DLEU2	1
TXNDC9	H19	1
TXNDC9	ZNRD1-AS1	1
TXNL4A	KTN1-AS1	1
TXNL4A	SCARNA9	1
TYMS	HCP5	1
TYMS	KTN1-AS1	1
TYMS	MCM3AP-AS1	1
TYSND1	HCP5	1
UBA2	MCM3AP-AS1	1
UBA2	SNHG3	1
UBA2	TP53TG1	1
UBASH3B	MCM3AP-AS1	1
UBASH3B	SCARNA9	1
UBE2C	H19	1
UBE2C	ZNRD1-AS1	1
UBE2E2	SNHG5	1
UBE2E2	ZNRD1-AS1	1
UBE2G2	HCP5	1
UBE2G2	KTN1-AS1	1
UBE2G2	SNHG3	1
UBE2H	HCP5	1
UBE2H	MCM3AP-AS1	1
UBE2H	SCARNA9	1
UBE2H	SNHG3	1
UBE2K	HCP5	1
UBE2K	KTN1-AS1	1
UBE2K	MCM3AP-AS1	1
UBE2L6	HCP5	1
UBE2T	SNHG3	1
UBE2V2	HCP5	1
UBE2V2	MCM3AP-AS1	1
UBE2V2	SCARNA9	1
UBE2V2	SNHG3	1
UBE2V2	TP53TG1	1

UBL4A	KTN1-AS1	1
UBN2	HCP5	1
UBN2	KTN1-AS1	1
UBN2	MCM3AP-AS1	1
UBN2	SCARNA9	1
UBN2	TP53TG1	1
UBR1	HCP5	1
UBR1	MCM3AP-AS1	1
UBR1	SNHG3	1
UBR1	TP53TG1	1
UBR3	H19	1
UBR3	SNHG5	1
UBR3	ZNRD1-AS1	1
UBR7	MCM3AP-AS1	1
UBTF	HCP5	1
UBTF	SCARNA9	1
UBXN2B	MCM3AP-AS1	1
UBXN8	HCP5	1
UBXN8	SNHG3	1
UCHL5	SNHG3	1
UEVLD	HCP5	1
UFD1L	HCP5	1
UGCG	DLEU2	1
UGCG	H19	1
UGCG	TPT1-AS1	1
UGCG	ZNRD1-AS1	1
UHRF1	SNHG3	1
UHRF1BP1	HCP5	1
UHRF1BP1L	KTN1-AS1	1
UHRF1BP1L	MCM3AP-AS1	1
ULK1	H19	1
ULK1	SNHG5	1
ULK1	ZNRD1-AS1	1
UNG	MCM3AP-AS1	1
UPF3B	MCM3AP-AS1	1
UQCR10	KTN1-AS1	1
UQCR10	MCM3AP-AS1	1
UQCRH	SCARNA9	1
URB2	MCM3AP-AS1	1
URB2	SCARNA9	1
URB2	TP53TG1	1
USP12	HCP5	1
USP12	MCM3AP-AS1	1
USP13	H19	1
USP13	ZNRD1-AS1	1
USP16	DLEU2	1
USP16	H19	1
USP16	TPT1-AS1	1
USP16	ZNRD1-AS1	1
USP2	MCM3AP-AS1	1
USP46	HCP5	1

USP46	KTN1-AS1	1
USP51	SCARNA9	1
USP53	DLEU2	1
USP53	H19	1
USP53	LINC00467	1
USP53	SNHG5	1
USP53	ZNRD1-AS1	1
UTP14A	HCP5	1
UTP14A	MCM3AP-AS1	1
UTP14A	RHPN1-AS1	1
UTP14A	SNHG3	1
UTRN	HCP5	1
UTRN	KTN1-AS1	1
UTRN	MCM3AP-AS1	1
UTRN	SCARNA9	1
UTRN	SNHG3	1
VAMP4	MCM3AP-AS1	1
VAMP8	MCM3AP-AS1	1
VAMP8	RHPN1-AS1	1
VANGL1	HCP5	1
VASH1	DLEU2	1
VASH1	TPT1-AS1	1
VAT1	DLEU2	1
VAT1	TPT1-AS1	1
VAT1	ZNRD1-AS1	1
VAV3	KTN1-AS1	1
VAV3	MCM3AP-AS1	1
VBP1	RUSC1-AS1	1
VBP1	SNHG5	1
VCAN	DLEU2	1
VCAN	RUSC1-AS1	1
VCAN	SNHG5	1
VCAN	TPT1-AS1	1
VDR	RUSC1-AS1	1
VDR	ZNRD1-AS1	1
VEGFA	DLEU2	1
VEGFA	H19	1
VEGFA	SNHG5	1
VEGFA	TPT1-AS1	1
VEGFA	ZNRD1-AS1	1
VGLL4	SCARNA9	1
VGLL4	SNHG3	1
VKORC1	MCM3AP-AS1	1
VKORC1L1	HCP5	1
VKORC1L1	KTN1-AS1	1
VKORC1L1	MCM3AP-AS1	1
VKORC1L1	SNHG3	1
VMP1	SNHG5	1
VMP1	ZNRD1-AS1	1
VPS26B	HCP5	1
VPS26B	RHPN1-AS1	1

VPS37C	ZNRD1-AS1	1
VRK1	HCP5	1
VRK1	MCM3AP-AS1	1
VRK1	SNHG3	1
WARS	HCP5	1
WASF1	H19	1
WASF1	RUSC1-AS1	1
WASF1	SNHG5	1
WASF1	ZNRD1-AS1	1
WASF2	H19	1
WASF2	RUSC1-AS1	1
WASF2	ZNRD1-AS1	1
WASL	HCP5	1
WASL	KTN1-AS1	1
WASL	MCM3AP-AS1	1
WASL	SCARNA9	1
WASL	TP53TG1	1
WBP2	KTN1-AS1	1
WBP2	MCM3AP-AS1	1
WBP4	KTN1-AS1	1
WBP4	MCM3AP-AS1	1
WDFY3	HCP5	1
WDFY3	SCARNA9	1
WDFY3	SNHG3	1
WDFY3	TP53TG1	1
WDR12	SNHG3	1
WDR35	SNHG3	1
WDR43	KTN1-AS1	1
WDR43	MCM3AP-AS1	1
WDR47	DLEU2	1
WDR47	H19	1
WDR47	TPT1-AS1	1
WDR47	ZNRD1-AS1	1
WDR5	HCP5	1
WDR5	RHPN1-AS1	1
WDR5	SNHG3	1
WDR54	KTN1-AS1	1
WDR5B	HCP5	1
WDR5B	SNHG3	1
WDR77	SCARNA9	1
WDR81	HCP5	1
WDTC1	LINC00467	1
WDTC1	ZNRD1-AS1	1
WEE1	HCP5	1
WEE1	KTN1-AS1	1
WEE1	MCM3AP-AS1	1
WFS1	HCP5	1
WFS1	RHPN1-AS1	1
WIBG	HCP5	1
WIBG	MCM3AP-AS1	1
WIPI1	MCM3AP-AS1	1

WLS	HCP5	1
WLS	SNHG3	1
WNK3	HCP5	1
WNK3	MCM3AP-AS1	1
WWC1	HCP5	1
WWC1	MCM3AP-AS1	1
WWTR1	HCP5	1
WWTR1	TP53TG1	1
XK	MCM3AP-AS1	1
XK	TP53TG1	1
XKR8	RHPN1-AS1	1
XPO4	HCP5	1
XPO4	MCM3AP-AS1	1
XPO7	HCP5	1
XPO7	MCM3AP-AS1	1
XPO7	SCARNA9	1
XPO7	SNHG3	1
XPO7	TP53TG1	1
XPOT	SNHG3	1
XRCC4	HCP5	1
XRRA1	HCP5	1
XYLT1	MCM3AP-AS1	1
XYLT1	SCARNA9	1
YARS	HCP5	1
YEATS4	HCP5	1
YEATS4	KTN1-AS1	1
YEATS4	SCARNA9	1
YIF1B	MCM3AP-AS1	1
YIPF6	KTN1-AS1	1
YIPF6	MCM3AP-AS1	1
YIPF6	SNHG3	1
YPEL2	DLEU2	1
YPEL2	H19	1
YPEL2	TPT1-AS1	1
YPEL2	ZNRD1-AS1	1
YPEL5	DLEU2	1
YPEL5	H19	1
YPEL5	LINC00467	1
YPEL5	TPT1-AS1	1
YRDC	MCM3AP-AS1	1
YRDC	SNHG3	1
YTHDC2	TP53TG1	1
YTHDF2	HCP5	1
YTHDF2	MCM3AP-AS1	1
YTHDF2	TP53TG1	1
ZBTB1	SCARNA9	1
ZBTB10	HCP5	1
ZBTB10	MCM3AP-AS1	1
ZBTB10	SNHG3	1
ZBTB17	SNHG5	1
ZBTB17	ZNRD1-AS1	1

ZBTB20	ZNRD1-AS1	1
ZBTB38	HCP5	1
ZBTB4	HCP5	1
ZBTB4	MCM3AP-AS1	1
ZBTB4	RHPN1-AS1	1
ZBTB43	DLEU2	1
ZBTB43	H19	1
ZBTB43	RUSC1-AS1	1
ZBTB43	ZNRD1-AS1	1
ZBTB44	HCP5	1
ZBTB44	KTN1-AS1	1
ZBTB44	MCM3AP-AS1	1
ZC3H12A	ZNRD1-AS1	1
ZC3H6	KTN1-AS1	1
ZC3H6	MCM3AP-AS1	1
ZC3H6	SCARNA9	1
ZC3H6	SNHG3	1
ZC3HAV1L	HCP5	1
ZC3HAV1L	MCM3AP-AS1	1
ZDBF2	HCP5	1
ZDBF2	KTN1-AS1	1
ZDBF2	MCM3AP-AS1	1
ZDBF2	SNHG3	1
ZDHHC1	HCP5	1
ZDHHC17	DLEU2	1
ZDHHC17	H19	1
ZDHHC17	RUSC1-AS1	1
ZDHHC17	SNHG5	1
ZDHHC17	TPT1-AS1	1
ZDHHC17	ZNRD1-AS1	1
ZDHHC2	H19	1
ZDHHC2	RUSC1-AS1	1
ZDHHC2	ZNRD1-AS1	1
ZEB1	H19	1
ZEB1	SNHG5	1
ZEB1	ZNRD1-AS1	1
ZER1	DLEU2	1
ZER1	H19	1
ZER1	SNHG5	1
ZER1	ZNRD1-AS1	1
ZFAND2A	MCM3AP-AS1	1
ZFC3H1	DLEU2	1
ZFC3H1	H19	1
ZFC3H1	RUSC1-AS1	1
ZFC3H1	SNHG5	1
ZFC3H1	TPT1-AS1	1
ZFC3H1	ZNRD1-AS1	1
ZFP36	HCP5	1
ZFP36L2	HCP5	1
ZFP36L2	MCM3AP-AS1	1
ZFP36L2	SNHG3	1

ZFP90	HCP5	1
ZFP90	SNHG3	1
ZFPM2	H19	1
ZFPM2	RUSC1-AS1	1
ZFPM2	ZNRD1-AS1	1
ZHX1	HCP5	1
ZHX1	MCM3AP-AS1	1
ZHX1	SNHG3	1
ZHX1	TP53TG1	1
ZHX2	HCP5	1
ZHX2	SNHG3	1
ZHX3	HCP5	1
ZHX3	MCM3AP-AS1	1
ZKSCAN1	HCP5	1
ZKSCAN1	KTN1-AS1	1
ZKSCAN1	MCM3AP-AS1	1
ZKSCAN1	SCARNA9	1
ZKSCAN1	SNHG3	1
ZKSCAN3	HCP5	1
ZKSCAN4	HCP5	1
ZKSCAN4	SCARNA9	1
ZMAT3	HCP5	1
ZMAT3	MCM3AP-AS1	1
ZMAT3	TP53TG1	1
ZMPSTE24	SNHG3	1
ZMYM3	SNHG3	1
ZNF100	DLEU2	1
ZNF100	TPT1-AS1	1
ZNF138	KTN1-AS1	1
ZNF138	MCM3AP-AS1	1
ZNF155	MCM3AP-AS1	1
ZNF155	RHPN1-AS1	1
ZNF2	HCP5	1
ZNF217	H19	1
ZNF217	RUSC1-AS1	1
ZNF217	SNHG5	1
ZNF217	ZNRD1-AS1	1
ZNF22	HCP5	1
ZNF22	MCM3AP-AS1	1
ZNF223	SNHG3	1
ZNF223	TP53TG1	1
ZNF230	HCP5	1
ZNF230	SCARNA9	1
ZNF230	SNHG3	1
ZNF239	HCP5	1
ZNF248	HCP5	1
ZNF248	KTN1-AS1	1
ZNF248	SNHG3	1
ZNF286A	HCP5	1
ZNF326	HCP5	1
ZNF326	KTN1-AS1	1

ZNF326	MCM3AP-AS1	1
ZNF362	HCP5	1
ZNF362	MCM3AP-AS1	1
ZNF362	RHPN1-AS1	1
ZNF362	SNHG3	1
ZNF367	HCP5	1
ZNF367	MCM3AP-AS1	1
ZNF367	SNHG3	1
ZNF395	HCP5	1
ZNF395	KTN1-AS1	1
ZNF395	MCM3AP-AS1	1
ZNF395	RHPN1-AS1	1
ZNF395	SNHG3	1
ZNF397	HCP5	1
ZNF397	MCM3AP-AS1	1
ZNF485	KTN1-AS1	1
ZNF485	MCM3AP-AS1	1
ZNF492	DLEU2	1
ZNF492	SNHG5	1
ZNF493	SNHG5	1
ZNF513	LINC00467	1
ZNF516	H19	1
ZNF516	SNHG5	1
ZNF521	KTN1-AS1	1
ZNF521	MCM3AP-AS1	1
ZNF521	SCARNA9	1
ZNF528	DLEU2	1
ZNF532	H19	1
ZNF532	SNHG5	1
ZNF532	ZNRD1-AS1	1
ZNF566	HCP5	1
ZNF569	TP53TG1	1
ZNF583	TP53TG1	1
ZNF607	MCM3AP-AS1	1
ZNF607	SNHG3	1
ZNF609	H19	1
ZNF609	SNHG5	1
ZNF609	ZNRD1-AS1	1
ZNF615	SCARNA9	1
ZNF622	MCM3AP-AS1	1
ZNF624	HCP5	1
ZNF624	MCM3AP-AS1	1
ZNF624	TP53TG1	1
ZNF652	HCP5	1
ZNF652	KTN1-AS1	1
ZNF652	MCM3AP-AS1	1
ZNF652	SNHG3	1
ZNF672	HCP5	1
ZNF672	RHPN1-AS1	1
ZNF681	HCP5	1
ZNF695	HCP5	1

ZNF695	SCARNA9	1
ZNF695	TP53TG1	1
ZNF724P	KTN1-AS1	1
ZNF730	KTN1-AS1	1
ZNF738	H19	1
ZNF738	ZNRD1-AS1	1
ZNF75A	H19	1
ZNF75A	SNHG5	1
ZNF75A	ZNRD1-AS1	1
ZNF77	KTN1-AS1	1
ZNF77	MCM3AP-AS1	1
ZNF823	HCP5	1
ZNF844	SCARNA9	1
ZNF85	DLEU2	1
ZNF85	SNHG5	1
ZNF93	H19	1
ZNF93	ZNRD1-AS1	1
ZNFX1	H19	1
ZNFX1	LINC00467	1
ZNFX1	ZNRD1-AS1	1
ZNRF1	DLEU2	1
ZNRF1	TPT1-AS1	1
ZNRF1	ZNRD1-AS1	1
ZNRF2	HCP5	1
ZNRF2	KTN1-AS1	1
ZNRF2	MCM3AP-AS1	1
ZNRF2	RHPN1-AS1	1
ZSWIM6	DLEU2	1
ZSWIM6	H19	1
ZSWIM6	SNHG5	1
ZSWIM6	TPT1-AS1	1
ZSWIM6	ZNRD1-AS1	1
ZW10	HCP5	1
ZW10	SNHG3	1
ZWILCH	MCM3AP-AS1	1
ZWINT	SCARNA9	1
ZXDA	SNHG5	1
ZXDA	ZNRD1-AS1	1
ZXDB	DLEU2	1
ZXDB	H19	1
ZXDB	RUSC1-AS1	1
ZXDB	TPT1-AS1	1
ZXDB	ZNRD1-AS1	1
AAAS	MCM3AP-AS1	1
ACOT12	HCP5	1
ACOT12	SNHG3	1
ADAMTS9	SCARNA9	1
ADRM1	ZNRD1-AS1	1
AGER	ZNRD1-AS1	1
AGO2	ZNRD1-AS1	1
ALDH16A1	HCP5	1

ALDH16A1	SNHG3	1
ALDH3B2	RHPN1-AS1	1
AMER1	HCP5	1
ARID5A	TPT1-AS1	1
ARL9	H19	1
ARL9	ZNRD1-AS1	1
ARSA	H19	1
ARSA	ZNRD1-AS1	1
ATP1A3	SCARNA9	1
AZIN2	DLEU2	1
AZIN2	TPT1-AS1	1
BCAS3	RUSC1-AS1	1
BCS1L	KTN1-AS1	1
BEND5	HCP5	1
BEND5	SNHG3	1
BLOC1S4	SCARNA9	1
BSPRY	MCM3AP-AS1	1
C14orf93	MCM3AP-AS1	1
CA11	SNHG5	1
CCDC169	SCARNA9	1
CCDC171	TP53TG1	1
CCL5	H19	1
CCL5	ZNRD1-AS1	1
CD244	ZNRD1-AS1	1
CD8A	SNHG3	1
CHEK2	HCP5	1
CHEK2	MCM3AP-AS1	1
CHRFAM7A	SCARNA9	1
CLUH	MCM3AP-AS1	1
CRHBP	MCM3AP-AS1	1
CTPS1	SNHG3	1
CXCL13	SNHG5	1
DLGAP3	MCM3AP-AS1	1
DNAAF5	MCM3AP-AS1	1
ELMSAN1	H19	1
ERP27	SCARNA9	1
EXO5	HCP5	1
FAM213A	HCP5	1
FAM229B	MCM3AP-AS1	1
FANCG	KTN1-AS1	1
FGGY	SNHG5	1
FMN1	SNHG5	1
GCHFR	SNHG5	1
GCSAM	DLEU2	1
GCSAM	TPT1-AS1	1
GCSAML	HCP5	1
GCSAML	SNHG3	1
GIMAP4	DLEU2	1
GPLD1	KTN1-AS1	1
GPR155	HCP5	1
GPR157	HCP5	1

GPR19	RHPN1-AS1	1
GSTM5	SCARNA9	1
GTF2IRD2B	HCP5	1
HEMGN	SCARNA9	1
HGD	SNHG5	1
HIST1H1A	DLEU2	1
HIST1H1A	TPT1-AS1	1
HIST2H3C	RHPN1-AS1	1
HIST2H4B	KTN1-AS1	1
HIST2H4B	MCM3AP-AS1	1
HLF	SCARNA9	1
HNRNPDL	ZNRD1-AS1	1
HSD17B8	MCM3AP-AS1	1
IDNK	MCM3AP-AS1	1
IL24	MCM3AP-AS1	1
JADE3	DLEU2	1
JADE3	TPT1-AS1	1
KIAA1958	SNHG5	1
KLHL42	SCARNA9	1
KLHL42	SNHG3	1
KRTAP5-3	SNHG5	1
KRTAP5-7	SNHG5	1
LAMC2	HCP5	1
LAMC2	RHPN1-AS1	1
LAMTOR2	MCM3AP-AS1	1
LANCL3	SCARNA9	1
LRRC63	SCARNA9	1
MMP10	SNHG5	1
MMP8	RUSC1-AS1	1
MMP8	SNHG5	1
MMP9	MCM3AP-AS1	1
MRPL21	MCM3AP-AS1	1
MRPL52	H19	1
MRPL52	ZNRD1-AS1	1
MUC4	MCM3AP-AS1	1
MYO1H	SCARNA9	1
NABP1	H19	1
NABP1	SNHG5	1
NABP1	ZNRD1-AS1	1
NDC1	RHPN1-AS1	1
NSMF	TPT1-AS1	1
PDE1B	RUSC1-AS1	1
PRIMPOL	MCM3AP-AS1	1
PTGIS	HCP5	1
R3HCC1L	H19	1
R3HCC1L	ZNRD1-AS1	1
RASEF	ZNRD1-AS1	1
RPF2	HCP5	1
RPL21	HCP5	1
RRP9	MCM3AP-AS1	1
RSRP1	H19	1

RSRP1	ZNRD1-AS1	1
S100B	H19	1
SGIP1	SCARNA9	1
SKIDA1	DLEU2	1
SKIDA1	TPT1-AS1	1
SLC35F6	HCP5	1
SLIRP	MCM3AP-AS1	1
SMDT1	HCP5	1
SMDT1	MCM3AP-AS1	1
SUSD6	H19	1
SUSD6	ZNRD1-AS1	1
TCAF2	MCM3AP-AS1	1
TLR6	KTN1-AS1	1
TLR6	MCM3AP-AS1	1
TMEM239	SNHG5	1
TNIP3	H19	1
TNIP3	ZNRD1-AS1	1
TPK1	HCP5	1
TPTE2	H19	1
TPTE2	ZNRD1-AS1	1
UBA7	MCM3AP-AS1	1
UQCRC1	H19	1
UQCRC1	ZNRD1-AS1	1
WDR45B	MCM3AP-AS1	1
WDR66	SCARNA9	1
XIRP2	H19	1
XIRP2	ZNRD1-AS1	1
ZBTB16	MCM3AP-AS1	1
ZBTB18	HCP5	1
ZNF391	MCM3AP-AS1	1
ZNF454	H19	1
ZNF454	ZNRD1-AS1	1
ZP3	RHPN1-AS1	1
