# Supplementary material for: Identifying Dysregulated lncRNA-Associated ceRNA Network Biomarkers in CML Based on Dynamical Network Biomarkers
Source: Biomed Res Int. 2020 Feb 18;2020:5189549. doi: 10.1155/2020/5189549 (PMC7049421; doi:10.1155/2020/5189549)
Supplement: Supplementary Materials — Supplementary Table 1: dysregulated lncRNA-mRNA competing interactions of CP, AP, BC for CML. Supplementary Table 2: dysregulated lncRNA-associated ceRNA networks of CP, AP, and BC for CML (DLCN_CP, DLCN_AP, and DLCN_BC). Supplementary Table 3: CeRNA network biomarkers in DLCN_CP, DLCN_AP, and DLCN_BC. Supplementary Table 4: significantly enriched pathways in DLCN_CP, DLCN_AP, and DLCN_BC. . [file 5189549.f1.zip › 5189549.f1/Supplementary Table S2.pdf]

Supplementary Table 2: Dysregulated lncRNA-associated ceRNA networks of CP, AP, BC for CML (DLCN_CP, DLCN_AP, DLCN_BC).

dysregulated lncRNA-associated ceRNA networks of CP for CML(DLCN_CP)			dysregulated lncRNA-associated ceRNA networks of AP for CML(DLCN_AP)			dysregulated lncRNA-associated ceRNA networks of CP for CML(DLCN_BC)		
Node type	Node name	Degree	Node type	Node name	Degree	Node type	Node name	Degree
lncRNA	HCP5	430	lncRNA	H19	546	lncRNA	HCP5	908
lncRNA	SNHG3	286	lncRNA	ZNRD1-AS1	509	lncRNA	MCM3AP-AS1	861
lncRNA	H19	258	lncRNA	HCP5	477	lncRNA	ZNRD1-AS1	694
lncRNA	ZNRD1-AS1	240	lncRNA	MCM3AP-AS1	407	lncRNA	H19	626
lncRNA	MCM3AP-AS1	235	lncRNA	SNHG3	339	lncRNA	SNHG3	545
lncRNA	SNHG5	214	lncRNA	SNHG5	305	lncRNA	SNHG5	392
lncRNA	KTN1-AS1	167	lncRNA	KTN1-AS1	191	lncRNA	KTN1-AS1	375
lncRNA	DLEU2	151	lncRNA	LINC00467	152	lncRNA	DLEU2	344
lncRNA	RUSC1-AS1	112	lncRNA	TPT1-AS1	143	lncRNA	TPT1-AS1	275
lncRNA	SCARNA9	84	lncRNA	TP53TG1	129	lncRNA	SCARNA9	255
lncRNA	TPT1-AS1	79	lncRNA	DLEU2	79	lncRNA	TP53TG1	204
lncRNA	TP53TG1	71	lncRNA	RHPN1-AS1	52	lncRNA	RUSC1-AS1	174
lncRNA	LINC00467	61	lncRNA	RUSC1-AS1	42	lncRNA	LINC00467	133
lncRNA	RHPN1-AS1	48	lncRNA	SCARNA9	40	lncRNA	RHPN1-AS1	105
mRNA	GLCCI1	7	mRNA	MAMDC2	7	mRNA	ATP2B1	7
mRNA	CPNE8	6	mRNA	ATXN1	6	mRNA	ITM2B	7
mRNA	AMOT	5	mRNA	CBX5	6	mRNA	NLK	7
mRNA	ATP2B1	5	mRNA	CPNE8	6	mRNA	NOTCH2	7
mRNA	EBF1	5	mRNA	E2F5	6	mRNA	PTPN13	7
mRNA	FAM117B	5	mRNA	FAM117B	6	mRNA	RCOR1	7
mRNA	FCHO2	5	mRNA	FAM160B1	6	mRNA	TCF7L2	7
mRNA	ITM2B	5	mRNA	GNAI1	6	mRNA	ARID4B	6
mRNA	KCTD12	5	mRNA	IQGAP2	6	mRNA	ARID5B	6
mRNA	KIAA0895	5	mRNA	NPR3	6	mRNA	ASPH	6
mRNA	KLF3	5	mRNA	PIK3R1	6	mRNA	BAZ2B	6
mRNA	SOCS5	5	mRNA	SP1	6	mRNA	BTG3	6
mRNA	TBC1D12	5	mRNA	TSHZ1	6	mRNA	BUB3	6
mRNA	ZDHHC17	5	mRNA	TTLL7	6	mRNA	CCNC	6
mRNA	ZNF395	5	mRNA	ZBTB44	6	mRNA	CLDND1	6
mRNA	ALCAM	4	mRNA	ZNF652	6	mRNA	CPNE8	6
mRNA	APPL1	4	mRNA	ADD3	5	mRNA	DENND4A	6
mRNA	ARL13B	4	mRNA	AKT3	5	mRNA	DPYSL2	6
mRNA	ASCC3	4	mRNA	ARHGEF3	5	mRNA	FNDC3B	6
mRNA	ASPH	4	mRNA	ARRDC4	5	mRNA	GAB1	6
mRNA	ATP2B4	4	mRNA	BAZ2B	5	mRNA	GATA6	6
mRNA	BAZ2B	4	mRNA	BNIP3L	5	mRNA	GLCCI1	6
mRNA	BTN3A1	4	mRNA	BTN3A1	5	mRNA	HMGB2	6
mRNA	CBX5	4	mRNA	BTN3A2	5	mRNA	IFIT5	6
mRNA	CCDC82	4	mRNA	BTN3A3	5	mRNA	IFNAR2	6
mRNA	CLIP4	4	mRNA	C16orf72	5	mRNA	JARID2	6
mRNA	CRIM1	4	mRNA	C1orf21	5	mRNA	KIAA0430	6
mRNA	CUTC	4	mRNA	C5orf30	5	mRNA	LIN9	6
mRNA	DENND4A	4	mRNA	CBX2	5	mRNA	LPP	6
mRNA	DPYSL2	4	mRNA	CDK6	5	mRNA	MAMDC2	6
mRNA	ELK3	4	mRNA	CREG1	5	mRNA	NAA15	6
mRNA	EMP1	4	mRNA	CTNNBIP1	5	mRNA	PDK1	6

mRNA	FLI1	4	mRNA	DCAF17	5	mRNA	PSD3	6
mRNA	FOXO3	4	mRNA	DPYD	5	mRNA	PSMC6	6
mRNA	FRMD6	4	mRNA	EDA2R	5	mRNA	RAB12	6
mRNA	GLCE	4	mRNA	EGLN3	5	mRNA	RNF19A	6
mRNA	GPR126	4	mRNA	FAM195A	5	mRNA	ROBO1	6
mRNA	IGF2BP3	4	mRNA	FMNL2	5	mRNA	SAMD8	6
mRNA	ISCU	4	mRNA	FRMD6	5	mRNA	SLC38A1	6
mRNA	ITGA2	4	mRNA	GCNT2	5	mRNA	SLC4A7	6
mRNA	ITGB8	4	mRNA	GLCCI1	5	mRNA	SMARCA1	6
mRNA	ITPR1	4	mRNA	GPR126	5	mRNA	STK38L	6
mRNA	JMJD8	4	mRNA	GRK5	5	mRNA	TAB2	6
mRNA	MAMDC2	4	mRNA	HCFC2	5	mRNA	TANK	6
mRNA	PRDM1	4	mRNA	HMGB2	5	mRNA	TCF4	6
mRNA	PRDM2	4	mRNA	HNRNPR	5	mRNA	TMEM38B	6
mRNA	PRKRA	4	mRNA	KIAA0101	5	mRNA	ZDHHC17	6
mRNA	PTPRM	4	mRNA	KLF3	5	mRNA	ZFC3H1	6
mRNA	RALA	4	mRNA	LRRC16A	5	mRNA	ACSL4	5
mRNA	RNF19A	4	mRNA	MAP3K5	5	mRNA	ADAM19	5
mRNA	RRAGD	4	mRNA	MAP7	5	mRNA	ADRA2A	5
mRNA	SAMD4A	4	mRNA	MLLT3	5	mRNA	AHNAK	5
mRNA	SBF2	4	mRNA	MYO5C	5	mRNA	AKAP12	5
mRNA	SCARB2	4	mRNA	PRKACB	5	mRNA	ARFGEF2	5
mRNA	SEH1L	4	mRNA	PTGFRN	5	mRNA	ARHGAP26	5
mRNA	SEMA6D	4	mRNA	RAVER2	5	mRNA	ARL5B	5
mRNA	SH3BP4	4	mRNA	RPS6KA5	5	mRNA	ATL2	5
mRNA	SIX4	4	mRNA	SLAIN1	5	mRNA	ATM	5
mRNA	SP1	4	mRNA	SPTLC2	5	mRNA	ATXN7L1	5
mRNA	STK17A	4	mRNA	STK38L	5	mRNA	BAZ1A	5
mRNA	STK17B	4	mRNA	TCF4	5	mRNA	BCL11A	5
mRNA	STK38L	4	mRNA	TRERF1	5	mRNA	BCL2	5
mRNA	TCF4	4	mRNA	ZFP36L2	5	mRNA	BCL6	5
mRNA	TRIB1	4	mRNA	ZNF521	5	mRNA	BECN1	5
mRNA	TRUB1	4	mRNA	AKAP1	4	mRNA	C5orf30	5
mRNA	TUBGCP4	4	mRNA	ANKH	4	mRNA	CAMSAP2	5
mRNA	WASF1	4	mRNA	ANKRD26	4	mRNA	CASP7	5
mRNA	ZBTB10	4	mRNA	APPL1	4	mRNA	CBX2	5
mRNA	ZEB1	4	mRNA	ASPH	4	mRNA	CCNJL	5
mRNA	ZFP36L2	4	mRNA	ATP2B1	4	mRNA	CD83	5
mRNA	ZNF217	4	mRNA	ATP2B4	4	mRNA	CDC23	5
mRNA	ZNF652	4	mRNA	BBS2	4	mRNA	CDK13	5
mRNA	ACADM	3	mRNA	BCL11A	4	mRNA	CDK6	5
mRNA	ALDH5A1	3	mRNA	C16orf70	4	mRNA	CHD2	5
mRNA	ANKRA2	3	mRNA	CASP2	4	mRNA	CPEB4	5
mRNA	AP3S1	3	mRNA	CAST	4	mRNA	CPS1	5
mRNA	APP	3	mRNA	CD302	4	mRNA	CRIM1	5
mRNA	ARHGAP32	3	mRNA	CDIPT	4	mRNA	CYBRD1	5
mRNA	ARHGEF3	3	mRNA	CDK13	4	mRNA	DNAJB12	5
mRNA	ARID4B	3	mRNA	CIT	4	mRNA	DNAJC3	5
mRNA	ARL5B	3	mRNA	CPNE3	4	mRNA	DSTYK	5
mRNA	ATAD2B	3	mRNA	CRAMP1L	4	mRNA	DUSP5	5
mRNA	ATG14	3	mRNA	CUTC	4	mRNA	DYNLT3	5
mRNA	ATG5	3	mRNA	CYBRD1	4	mRNA	EBF1	5
mRNA	ATP1B1	3	mRNA	DEK	4	mRNA	EMP1	5

mRNA	ATPAF1	3	mRNA	DENND4A	4	mRNA	ENC1	5
mRNA	ATXN1	3	mRNA	DHODH	4	mRNA	ENPP4	5
mRNA	B4GALT6	3	mRNA	DPYSL3	4	mRNA	ERI1	5
mRNA	BAZ1A	3	mRNA	EFNA3	4	mRNA	FAM160B1	5
mRNA	BCL11A	3	mRNA	ELK3	4	mRNA	FAM177A1	5
mRNA	BTN3A3	3	mRNA	EZH2	4	mRNA	FAM43A	5
mRNA	C16orf70	3	mRNA	FAM134A	4	mRNA	FAM46A	5
mRNA	C6orf120	3	mRNA	FAM45A	4	mRNA	FAM46C	5
mRNA	CCDC15	3	mRNA	FAM46A	4	mRNA	FCHO2	5
mRNA	CCDC71L	3	mRNA	FBXL5	4	mRNA	FERMT2	5
mRNA	CCNC	3	mRNA	GAB1	4	mRNA	GINS1	5
mRNA	CD164	3	mRNA	HABP4	4	mRNA	GLI3	5
mRNA	CDH2	3	mRNA	HNRNPAB	4	mRNA	GNAI1	5
mRNA	CHD2	3	mRNA	HOXA3	4	mRNA	HBS1L	5
mRNA	CLIC4	3	mRNA	HOXA7	4	mRNA	HNRNPR	5
mRNA	COBLL1	3	mRNA	IGF2BP3	4	mRNA	IL15	5
mRNA	CPNE3	3	mRNA	JARID2	4	mRNA	IRF4	5
mRNA	CRBN	3	mRNA	KCTD3	4	mRNA	ITGB8	5
mRNA	CREM	3	mRNA	KIF11	4	mRNA	ITPR1	5
mRNA	CUL4B	3	mRNA	KIF23	4	mRNA	KIAA0226	5
mRNA	CYBRD1	3	mRNA	KIF2A	4	mRNA	KIF5B	5
mRNA	DCTN4	3	mRNA	LANCL1	4	mRNA	KLF3	5
mRNA	DCUN1D5	3	mRNA	MAP3K3	4	mRNA	KLF4	5
mRNA	DHODH	3	mRNA	MCF2L	4	mRNA	KLF6	5
mRNA	DHX33	3	mRNA	MECOM	4	mRNA	KLHL2	5
mRNA	DNAJA3	3	mRNA	MPPED2	4	mRNA	LAMC1	5
mRNA	DRAM1	3	mRNA	MRRF	4	mRNA	LRCH2	5
mRNA	DSTYK	3	mRNA	MYO5A	4	mRNA	MAP3K3	5
mRNA	DUSP8	3	mRNA	NAA50	4	mRNA	MAST3	5
mRNA	ERICH1	3	mRNA	NAPEPLD	4	mRNA	MAST4	5
mRNA	ETS1	3	mRNA	NUDCD2	4	mRNA	MYCBP2	5
mRNA	FAM126A	3	mRNA	NUP210	4	mRNA	MYH9	5
mRNA	FAM134A	3	mRNA	OAS2	4	mRNA	MYLIP	5
mRNA	FAM46A	3	mRNA	PANK1	4	mRNA	NAV1	5
mRNA	FAT4	3	mRNA	PATZ1	4	mRNA	NFIA	5
mRNA	FNDC3B	3	mRNA	PBX3	4	mRNA	NID1	5
mRNA	FSD1L	3	mRNA	PBXIP1	4	mRNA	NPLOC4	5
mRNA	GAB1	3	mRNA	PDIK1L	4	mRNA	NRP1	5
mRNA	GCNT2	3	mRNA	PHF19	4	mRNA	NUS1	5
mRNA	GOLPH3	3	mRNA	PNKD	4	mRNA	OAS2	5
mRNA	GPAM	3	mRNA	PSIP1	4	mRNA	OTUD6B	5
mRNA	GPT2	3	mRNA	PTEN	4	mRNA	PAM	5
mRNA	GRK5	3	mRNA	PTPN13	4	mRNA	PAPD5	5
mRNA	HCFC2	3	mRNA	PXK	4	mRNA	PDSS1	5
mRNA	HIVEP1	3	mRNA	RUNX2	4	mRNA	PEAK1	5
mRNA	HIVEP2	3	mRNA	SBF2	4	mRNA	PHF6	5
mRNA	HNRNPR	3	mRNA	SCAPER	4	mRNA	PICALM	5
mRNA	HS2ST1	3	mRNA	SESN1	4	mRNA	PIK3C2B	5
mRNA	HS6ST2	3	mRNA	SESN2	4	mRNA	PIK3R1	5
mRNA	HSPD1	3	mRNA	SFXN2	4	mRNA	PPAP2B	5
mRNA	IVNS1ABP	3	mRNA	SLC16A1	4	mRNA	PTGFRN	5
mRNA	JAG2	3	mRNA	SLC48A1	4	mRNA	PTPDC1	5
mRNA	KAL1	3	mRNA	SMARCA1	4	mRNA	PTPN12	5

mRNA	KDM6B	3	mRNA	SPIRE1	4	mRNA	PTPRM	5
mRNA	KIF3B	3	mRNA	STK17A	4	mRNA	RABGAP1L	5
mRNA	KIF5B	3	mRNA	STK32B	4	mRNA	RAI14	5
mRNA	KIF5C	3	mRNA	STOM	4	mRNA	RAPH1	5
mRNA	KLF4	3	mRNA	SUFU	4	mRNA	RGL1	5
mRNA	LRCH2	3	mRNA	TCF7L2	4	mRNA	RNF44	5
mRNA	LYRM1	3	mRNA	TET3	4	mRNA	RTKN2	5
mRNA	MAP7	3	mRNA	TFPI	4	mRNA	SENP5	5
mRNA	MARCKS	3	mRNA	TMEM201	4	mRNA	SGK1	5
mRNA	MBOAT2	3	mRNA	TMEM25	4	mRNA	SH3BP4	5
mRNA	MDFIC	3	mRNA	TMEM65	4	mRNA	SHCBP1	5
mRNA	MECOM	3	mRNA	TP53	4	mRNA	SIX4	5
mRNA	MGST1	3	mRNA	TP53INP1	4	mRNA	SLC25A24	5
mRNA	MRPL37	3	mRNA	TPP1	4	mRNA	SLC39A14	5
mRNA	MXI1	3	mRNA	TRIM59	4	mRNA	SLTM	5
mRNA	MYLIP	3	mRNA	TTC9	4	mRNA	SMAD1	5
mRNA	NAA15	3	mRNA	UBE2K	4	mRNA	SNX9	5
mRNA	NBEA	3	mRNA	UBN2	4	mRNA	SPTY2D1	5
mRNA	NCOR2	3	mRNA	WASF1	4	mRNA	STX7	5
mRNA	NDFIP1	3	mRNA	WDR47	4	mRNA	TACC1	5
mRNA	NFIA	3	mRNA	WEE1	4	mRNA	TBCEL	5
mRNA	NOG	3	mRNA	ZBTB4	4	mRNA	TET3	5
mRNA	NUCB2	3	mRNA	ZHX1	4	mRNA	TIMP2	5
mRNA	OTUD1	3	mRNA	ZNF217	4	mRNA	TLE1	5
mRNA	PAICS	3	mRNA	ZNF248	4	mRNA	TLE4	5
mRNA	PAM	3	mRNA	ZNF326	4	mRNA	TP53INP1	5
mRNA	PANK1	3	mRNA	ZNF362	4	mRNA	TTLL7	5
mRNA	PCTP	3	mRNA	ZNF395	4	mRNA	UBE2V2	5
mRNA	PDK1	3	mRNA	ABCA5	3	mRNA	UBN2	5
mRNA	PDK4	3	mRNA	ACTL6A	3	mRNA	USP53	5
mRNA	PHKA1	3	mRNA	ADK	3	mRNA	UTRN	5
mRNA	PLXNA2	3	mRNA	ADRA2A	3	mRNA	VEGFA	5
mRNA	PNRC1	3	mRNA	ADSS	3	mRNA	WASL	5
mRNA	PPAP2B	3	mRNA	AEN	3	mRNA	XPO7	5
mRNA	PPIH	3	mRNA	AGFG2	3	mRNA	ZKSCAN1	5
mRNA	PREPL	3	mRNA	AMOT	3	mRNA	ZNF395	5
mRNA	PRKACB	3	mRNA	ANGPT1	3	mRNA	ZSWIM6	5
mRNA	PRRG1	3	mRNA	ANKRD46	3	mRNA	ZXDB	5
mRNA	PSD3	3	mRNA	AP1S3	3	mRNA	ABCD3	4
mRNA	PTPDC1	3	mRNA	AP3D1	3	mRNA	ABL1	4
mRNA	RBM47	3	mRNA	APP	3	mRNA	ACADM	4
mRNA	RBPMS	3	mRNA	ARID5B	3	mRNA	ACER3	4
mRNA	RCOR1	3	mRNA	ARL2	3	mRNA	ACSL1	4
mRNA	RIOK3	3	mRNA	ARL5B	3	mRNA	ACTN1	4
mRNA	RNF150	3	mRNA	ASAH1	3	mRNA	ADD3	4
mRNA	RTKN2	3	mRNA	ASB9	3	mRNA	ADK	4
mRNA	SCAI	3	mRNA	ATAD5	3	mRNA	ADSS	4
mRNA	SCAPER	3	mRNA	ATP8A1	3	mRNA	AKAP1	4
mRNA	SEC61A2	3	mRNA	ATXN7L1	3	mRNA	AKT3	4
mRNA	SEMA6A	3	mRNA	B4GALT6	3	mRNA	ALCAM	4
mRNA	SERP1	3	mRNA	BCL9L	3	mRNA	ALG2	4
mRNA	SESN1	3	mRNA	BTBD8	3	mRNA	AMOT	4
mRNA	SGMS1	3	mRNA	C12orf5	3	mRNA	ANKRD26	4

mRNA	SHISA5	3	mRNA	C16orf87	3	mRNA	ANKRD28	4
mRNA	SLC16A9	3	mRNA	C18orf54	3	mRNA	AP3D1	4
mRNA	SLC35D2	3	mRNA	C1orf74	3	mRNA	APC	4
mRNA	SLC39A14	3	mRNA	CCM2	3	mRNA	APP	4
mRNA	SLC4A7	3	mRNA	CCP110	3	mRNA	APPL1	4
mRNA	SLC5A3	3	mRNA	CDADC1	3	mRNA	ARFGAP3	4
mRNA	SLTM	3	mRNA	CDCA7	3	mRNA	ARHGAP32	4
mRNA	SMAD5	3	mRNA	CDK14	3	mRNA	ARL6IP5	4
mRNA	SORL1	3	mRNA	CDK18	3	mRNA	ARL6IP6	4
mRNA	SPRED1	3	mRNA	CDKN1B	3	mRNA	ARRDC4	4
mRNA	SRSF7	3	mRNA	CENPF	3	mRNA	ASCC3	4
mRNA	SSBP2	3	mRNA	CEP68	3	mRNA	ATAD2B	4
mRNA	SYDE2	3	mRNA	CERCAM	3	mRNA	ATG5	4
mRNA	SYPL1	3	mRNA	CHD2	3	mRNA	ATP1B1	4
mRNA	TAB2	3	mRNA	CHD5	3	mRNA	ATP2A2	4
mRNA	TAB3	3	mRNA	CLIC4	3	mRNA	ATXN1	4
mRNA	TANK	3	mRNA	CLSPN	3	mRNA	B4GALT5	4
mRNA	TBC1D4	3	mRNA	CNKSR3	3	mRNA	B4GALT6	4
mRNA	TCF7L2	3	mRNA	COBLL1	3	mRNA	BCOR	4
mRNA	TFPI	3	mRNA	CPA3	3	mRNA	BNIP3L	4
mRNA	TGFBR2	3	mRNA	CPEB2	3	mRNA	BSDC1	4
mRNA	THAP11	3	mRNA	CTDSPL	3	mRNA	C16orf72	4
mRNA	TLE4	3	mRNA	CTTNBP2NL	3	mRNA	C5orf15	4
mRNA	TMEM65	3	mRNA	CUL4B	3	mRNA	C6orf211	4
mRNA	TOMM34	3	mRNA	DACH1	3	mRNA	CAB39	4
mRNA	TSHZ1	3	mRNA	DCUN1D3	3	mRNA	CADM1	4
mRNA	UBN2	3	mRNA	DENND5A	3	mRNA	CALU	4
mRNA	USP16	3	mRNA	DEPDC1	3	mRNA	CAMK2D	4
mRNA	USP53	3	mRNA	DHX33	3	mRNA	CAST	4
mRNA	WASL	3	mRNA	DIRC2	3	mRNA	CBX5	4
mRNA	WDR47	3	mRNA	DNMT1	3	mRNA	CCDC82	4
mRNA	YEATS4	3	mRNA	DRAM1	3	mRNA	CCNA1	4
mRNA	ZBTB44	3	mRNA	DUSP16	3	mRNA	CD164	4
mRNA	ZFC3H1	3	mRNA	EEPD1	3	mRNA	CD80	4
mRNA	ZHX1	3	mRNA	EHD3	3	mRNA	CDH1	4
mRNA	ZHX3	3	mRNA	EML4	3	mRNA	CDH2	4
mRNA	ZMAT3	3	mRNA	ERI2	3	mRNA	CDK14	4
mRNA	ZNF326	3	mRNA	ERO1L	3	mRNA	CENPF	4
mRNA	ZXDB	3	mRNA	ESCO2	3	mRNA	CHD7	4
mRNA	AADAT	2	mRNA	EXOSC8	3	mRNA	CHUK	4
mRNA	ABCD3	2	mRNA	EXPH5	3	mRNA	COBLL1	4
mRNA	ABL1	2	mRNA	EZH1	3	mRNA	COL15A1	4
mRNA	ACVR1C	2	mRNA	FAM133A	3	mRNA	CPEB2	4
mRNA	ADAM19	2	mRNA	FAM136A	3	mRNA	CPNE3	4
mRNA	ADAT2	2	mRNA	FAM189B	3	mRNA	CREB5	4
mRNA	ADD3	2	mRNA	FAM46C	3	mRNA	CREG1	4
mRNA	ADK	2	mRNA	FAM53B	3	mRNA	CREM	4
mRNA	ADSS	2	mRNA	FAM65B	3	mRNA	CSF1	4
mRNA	AKT3	2	mRNA	FAM84B	3	mRNA	CSNK1G1	4
mRNA	AKTIP	2	mRNA	FIGN	3	mRNA	CTGF	4
mRNA	ALPK1	2	mRNA	FLOT2	3	mRNA	CUL4B	4
mRNA	AMPD2	2	mRNA	FOXM1	3	mRNA	CUTC	4
mRNA	ANGPT1	2	mRNA	FOXRED1	3	mRNA	CUX1	4

mRNA	ANKRD26	2
mRNA	ANKRD28	2
mRNA	ANXA6	2
mRNA	APC	2
mRNA	ARAP2	2
mRNA	ARFGEF2	2
mRNA	ARHGAP12	2
mRNA	ARID5B	2
mRNA	ATXN7L1	2
mRNA	B4GALT4	2
mRNA	BACE1	2
mRNA	BACH2	2
mRNA	BAG4	2
mRNA	BCCIP	2
mRNA	BCL2	2
mRNA	BCL6	2
mRNA	BEND7	2
mRNA	BNIP3L	2
mRNA	BRE	2
mRNA	BRIX1	2
mRNA	BSDC1	2
mRNA	BTAF1	2
mRNA	BTBD2	2
mRNA	C11orf49	2
mRNA	C12orf4	2
mRNA	C16orf87	2
mRNA	C20orf194	2
mRNA	C4orf19	2
mRNA	C5	2
mRNA	C5orf30	2
mRNA	C6orf211	2
mRNA	CAB39	2
mRNA	CAMK2G	2
mRNA	CAMLG	2
mRNA	CAMSAP2	2
mRNA	CARD16	2
mRNA	CARS	2
mRNA	CASP7	2
mRNA	CCPG1	2
mRNA	CD302	2
mRNA	CD44	2
mRNA	CDC7	2
mRNA	CDK14	2
mRNA	CDK6	2
mRNA	CDKN1B	2
mRNA	CDYL2	2
mRNA	CEBPG	2
mRNA	CECR1	2
mRNA	CENPA	2
mRNA	CENPF	2
mRNA	CHM	2
mRNA	CIT	2
mRNA	CLEC2D	2
mRNA	FOXRED2	3
mRNA	FYN	3
mRNA	FZD5	3
mRNA	GAPT	3
mRNA	GARS	3
mRNA	GCA	3
mRNA	GCNT1	3
mRNA	GLCE	3
mRNA	GLIPR1	3
mRNA	GORAB	3
mRNA	GPSM2	3
mRNA	GPT2	3
mRNA	GULP1	3
mRNA	H2AFJ	3
mRNA	HIVEP1	3
mRNA	HOXA5	3
mRNA	IGFBP7	3
mRNA	INTS6	3
mRNA	IQSEC1	3
mRNA	IRF8	3
mRNA	ITM2B	3
mRNA	ITPR1	3
mRNA	JAG2	3
mRNA	JUP	3
mRNA	KATNAL1	3
mRNA	KCTD12	3
mRNA	KDM2B	3
mRNA	KIAA0430	3
mRNA	KIAA1161	3
mRNA	KIAA1211	3
mRNA	KIAA1462	3
mRNA	KLF12	3
mRNA	KLF7	3
mRNA	LHFPL2	3
mRNA	LIPT2	3
mRNA	LRP5	3
mRNA	LRRK2	3
mRNA	LYRM1	3
mRNA	MAP4K4	3
mRNA	MAP7D2	3
mRNA	MAST3	3
mRNA	MAST4	3
mRNA	MCM4	3
mRNA	MDM2	3
mRNA	MMD	3
mRNA	MYLIP	3
mRNA	NAV1	3
mRNA	NCOR2	3
mRNA	NDFIP1	3
mRNA	NEK9	3
mRNA	NFE2L1	3
mRNA	NFIA	3
mRNA	NIPAL2	3
mRNA	DAG1	4
mRNA	DCBLD2	4
mRNA	DCLRE1B	4
mRNA	DCTN4	4
mRNA	DHCR24	4
mRNA	DHX15	4
mRNA	DNAJA3	4
mRNA	DNAJB11	4
mRNA	DNM3	4
mRNA	DOCK4	4
mRNA	DOCK9	4
mRNA	DPYD	4
mRNA	DRAM1	4
mRNA	DST	4
mRNA	E2F5	4
mRNA	ECHDC1	4
mRNA	ECT2	4
mRNA	ELK3	4
mRNA	ELL2	4
mRNA	EPB41L2	4
mRNA	EPB41L5	4
mRNA	ERG	4
mRNA	ERI2	4
mRNA	ERICH1	4
mRNA	ERLIN2	4
mRNA	ETF1	4
mRNA	ETV5	4
mRNA	EZH2	4
mRNA	FAM117B	4
mRNA	FAM126A	4
mRNA	FAM133A	4
mRNA	FAM45A	4
mRNA	FAM63B	4
mRNA	FBXW7	4
mRNA	FIGN	4
mRNA	FRMD6	4
mRNA	FUBP3	4
mRNA	GALNT1	4
mRNA	GBP1	4
mRNA	GCNT2	4
mRNA	GLIPR1	4
mRNA	GOLT1B	4
mRNA	GORAB	4
mRNA	GPAM	4
mRNA	GPR126	4
mRNA	GRK5	4
mRNA	HBEGF	4
mRNA	HIVEP1	4
mRNA	HIVEP2	4
mRNA	HMBOX1	4
mRNA	HOMER1	4
mRNA	HOXA9	4
mRNA	HS2ST1	4

mRNA	CLGN	2	mRNA	NIPAL4	3	mRNA	HSPH1	4
mRNA	CLIP1	2	mRNA	NKIRAS1	3	mRNA	IGF1R	4
mRNA	CNOT6L	2	mRNA	NLGN1	3	mRNA	IGF2BP3	4
mRNA	CPEB2	2	mRNA	NLK	3	mRNA	IGFBP5	4
mRNA	CPEB4	2	mRNA	NME4	3	mRNA	IKZF2	4
mRNA	CREB5	2	mRNA	NMT2	3	mRNA	IL1RAP	4
mRNA	CREBBP	2	mRNA	NOTCH2	3	mRNA	IQGAP1	4
mRNA	CRTAP	2	mRNA	NR2C2AP	3	mRNA	ITGA2	4
mRNA	CSNK1G1	2	mRNA	NREP	3	mRNA	JAG2	4
mRNA	CTNNAL1	2	mRNA	ORAI3	3	mRNA	JMJD8	4
mRNA	CTNNBIP1	2	mRNA	PARP1	3	mRNA	KAL1	4
mRNA	CUX1	2	mRNA	PCMTD2	3	mRNA	KAT2B	4
mRNA	DACH1	2	mRNA	PCYOX1L	3	mRNA	KBTBD8	4
mRNA	DBP	2	mRNA	PDCD4	3	mRNA	KCTD12	4
mRNA	DEK	2	mRNA	PIK3IP1	3	mRNA	KDELC1	4
mRNA	DGKH	2	mRNA	PLCL2	3	mRNA	KDM6B	4
mRNA	DIRC2	2	mRNA	PLK4	3	mRNA	KIAA0922	4
mRNA	DIS3	2	mRNA	PLSCR4	3	mRNA	KIF23	4
mRNA	DLAT	2	mRNA	POLD3	3	mRNA	KIF3B	4
mRNA	DLEU1	2	mRNA	POLE3	3	mRNA	KLF11	4
mRNA	DNAJC18	2	mRNA	PPARA	3	mRNA	KLF12	4
mRNA	DPYD	2	mRNA	PPARD	3	mRNA	KLF5	4
mRNA	DPYSL3	2	mRNA	PPRC1	3	mRNA	LANCL1	4
mRNA	DSC2	2	mRNA	PRDM2	3	mRNA	LHFPL2	4
mRNA	DST	2	mRNA	PRKAR2B	3	mRNA	LMO4	4
mRNA	DUSP1	2	mRNA	PRR15	3	mRNA	LRIG1	4
mRNA	DUSP18	2	mRNA	PSAT1	3	mRNA	LRP5	4
mRNA	E2F5	2	mRNA	PTGER2	3	mRNA	LRRFIP1	4
mRNA	ECHDC2	2	mRNA	PTPN9	3	mRNA	LYST	4
mRNA	EDA2R	2	mRNA	RABGAP1	3	mRNA	MAD2L1	4
mRNA	EFNA3	2	mRNA	RAD18	3	mRNA	MAF	4
mRNA	EGLN3	2	mRNA	RASGEF1A	3	mRNA	MAP3K5	4
mRNA	EIF2AK4	2	mRNA	RBPMS	3	mRNA	MAP4K4	4
mRNA	EIF2B2	2	mRNA	RFK	3	mRNA	MAP7	4
mRNA	EIF2S2	2	mRNA	RGMA	3	mRNA	MAPKAP1	4
mRNA	ELL	2	mRNA	RHOU	3	mRNA	MARCKS	4
mRNA	ELOVL6	2	mRNA	RIN2	3	mRNA	MCF2L	4
mRNA	EMB	2	mRNA	RNF24	3	mRNA	MCM4	4
mRNA	ENOX1	2	mRNA	ROBO1	3	mRNA	MED12L	4
mRNA	EPB41L2	2	mRNA	RPL22	3	mRNA	MED15	4
mRNA	ERG	2	mRNA	RTKN2	3	mRNA	MEF2A	4
mRNA	ERLIN2	2	mRNA	SAMD10	3	mRNA	MEIS1	4
mRNA	EXPH5	2	mRNA	SAMD4A	3	mRNA	METAP1	4
mRNA	FADD	2	mRNA	SEC24A	3	mRNA	MGST1	4
mRNA	FAIM3	2	mRNA	SEC61A2	3	mRNA	MSH2	4
mRNA	FAM134B	2	mRNA	SEMA6D	3	mRNA	MTMR9	4
mRNA	FAM171B	2	mRNA	SEPN1	3	mRNA	MYO5A	4
mRNA	FAM188A	2	mRNA	SESN3	3	mRNA	NAGPA	4
mRNA	FAM45A	2	mRNA	SKA2	3	mRNA	NBEA	4
mRNA	FAM46C	2	mRNA	SLC31A1	3	mRNA	NEDD9	4
mRNA	FBXW7	2	mRNA	SLC35F1	3	mRNA	NEGR1	4
mRNA	FCHO1	2	mRNA	SLC46A1	3	mRNA	NKIRAS1	4
mRNA	FER	2	mRNA	SMARCA2	3	mRNA	NR4A2	4

mRNA	FERMT2	2	mRNA	SOCS5	3	mRNA	NUDT8	4
mRNA	FHL1	2	mRNA	SRSF12	3	mRNA	NUPL1	4
mRNA	FIGN	2	mRNA	STC2	3	mRNA	OTUD1	4
mRNA	FKBP9	2	mRNA	STX3	3	mRNA	PAG1	4
mRNA	FMNL2	2	mRNA	SULF2	3	mRNA	PALLD	4
mRNA	FOSB	2	mRNA	SUN2	3	mRNA	PAPSS2	4
mRNA	FOXN2	2	mRNA	SUV39H2	3	mRNA	PATZ1	4
mRNA	FRAT1	2	mRNA	SYPL1	3	mRNA	PBLD	4
mRNA	FRY	2	mRNA	TAB2	3	mRNA	PBX3	4
mRNA	GATA3	2	mRNA	TBC1D14	3	mRNA	PCMTD1	4
mRNA	GATM	2	mRNA	TBC1D4	3	mRNA	PDE4B	4
mRNA	GDE1	2	mRNA	TBX19	3	mRNA	PDGFRB	4
mRNA	GGCT	2	mRNA	TCEA2	3	mRNA	PGBD2	4
mRNA	GLIPR1	2	mRNA	TET1	3	mRNA	PHF11	4
mRNA	GLTP	2	mRNA	THRA	3	mRNA	PI4K2B	4
mRNA	GM2A	2	mRNA	TIMM50	3	mRNA	PIGA	4
mRNA	GPD1L	2	mRNA	TMEM2	3	mRNA	PKP4	4
mRNA	GPR137C	2	mRNA	TMEM242	3	mRNA	PLAG1	4
mRNA	GPR63	2	mRNA	TMEM38B	3	mRNA	PLEKHA8	4
mRNA	HECA	2	mRNA	TMEM57	3	mRNA	PLEKHM1	4
mRNA	HLA-DQB1	2	mRNA	TMTC4	3	mRNA	PLK4	4
mRNA	HMGA1	2	mRNA	TNFRSF10B	3	mRNA	PLSCR4	4
mRNA	HNRNPA3	2	mRNA	TOMM34	3	mRNA	PMEPA1	4
mRNA	HOMER1	2	mRNA	TRIM14	3	mRNA	PNKD	4
mRNA	HOXA3	2	mRNA	TRIM4	3	mRNA	PPAT	4
mRNA	HOXA7	2	mRNA	TRUB1	3	mRNA	PRC1	4
mRNA	HPS3	2	mRNA	TTC30B	3	mRNA	PRCP	4
mRNA	IARS	2	mRNA	TUBGCP4	3	mRNA	PRDM1	4
mRNA	IFI16	2	mRNA	USP2	3	mRNA	PRKACB	4
mRNA	IFNAR2	2	mRNA	UTRN	3	mRNA	PRKCB	4
mRNA	IGFBP7	2	mRNA	VAMP8	3	mRNA	PRRG1	4
mRNA	IL15	2	mRNA	WASL	3	mRNA	PSIP1	4
mRNA	IMPA2	2	mRNA	WDR43	3	mRNA	PTEN	4
mRNA	INPP1	2	mRNA	WDTC1	3	mRNA	PTPRK	4
mRNA	INTS6	2	mRNA	XPO7	3	mRNA	PXK	4
mRNA	IQGAP2	2	mRNA	YPEL2	3	mRNA	RAB11FIP1	4
mRNA	IQSEC1	2	mRNA	ZBTB10	3	mRNA	RAB5B	4
mRNA	ITGA6	2	mRNA	ZC3H6	3	mRNA	RABGAP1	4
mRNA	JUP	2	mRNA	ZDHHC17	3	mRNA	RALA	4
mRNA	KANK1	2	mRNA	ZFP90	3	mRNA	RASGRP3	4
mRNA	KBTBD6	2	mRNA	ZKSCAN1	3	mRNA	RGS2	4
mRNA	KCTD15	2	mRNA	ZNF583	3	mRNA	RHOB	4
mRNA	KCTD3	2	mRNA	ZNF624	3	mRNA	RIN2	4
mRNA	KCTD9	2	mRNA	ZXDB	3	mRNA	RPGRIP1L	4
mRNA	KDM2B	2	mRNA	AMER1	3	mRNA	RPS6KA5	4
mRNA	KIAA0101	2	mRNA	HIST2H4B	3	mRNA	RRAGD	4
mRNA	KIAA0430	2	mRNA	IDNK	3	mRNA	RUNX2	4
mRNA	KIAA0922	2	mRNA	SMDT1	3	mRNA	SAMD4A	4
mRNA	KIAA1161	2	mRNA	ZBTB18	3	mRNA	SAP30BP	4
mRNA	KIAA1211	2	mRNA	AADAT	2	mRNA	SBF2	4
mRNA	KIF11	2	mRNA	ABHD14B	2	mRNA	SCAPER	4
mRNA	KIF13A	2	mRNA	ABHD4	2	mRNA	SCARB2	4
mRNA	KIF13B	2	mRNA	ABL1	2	mRNA	SDCCAG8	4

mRNA	KIF20B	2	mRNA	ACADM	2	mRNA	SEC24A	4
mRNA	KLF6	2	mRNA	ACSL1	2	mRNA	SEH1L	4
mRNA	L2HGDH	2	mRNA	ACSL4	2	mRNA	SEMA3C	4
mRNA	LANCL1	2	mRNA	ADA	2	mRNA	SEMA6D	4
mRNA	LCLAT1	2	mRNA	ADAM10	2	mRNA	SERP1	4
mRNA	LIN9	2	mRNA	ADRBK1	2	mRNA	SGK3	4
mRNA	LIPT2	2	mRNA	ADRBK2	2	mRNA	SGMS2	4
mRNA	LIX1L	2	mRNA	AHDC1	2	mRNA	SH2B3	4
mRNA	LMBR1L	2	mRNA	AKAP12	2	mRNA	SH3BP5	4
mRNA	LRBA	2	mRNA	AKR7A2	2	mRNA	SLAIN1	4
mRNA	LRP1	2	mRNA	ALDH3B1	2	mRNA	SLC16A1	4
mRNA	LRRFIP1	2	mRNA	ALPK1	2	mRNA	SLC16A9	4
mRNA	LRRK2	2	mRNA	AMPD2	2	mRNA	SLC30A9	4
mRNA	LSM5	2	mRNA	ANKRA2	2	mRNA	SLC5A3	4
mRNA	LTV1	2	mRNA	ANKRD28	2	mRNA	SMAD5	4
mRNA	MAF	2	mRNA	ANKRD6	2	mRNA	SMAD6	4
mRNA	MAP3K3	2	mRNA	ANPEP	2	mRNA	SNX25	4
mRNA	MAPKAP1	2	mRNA	ANTXR1	2	mRNA	SOCS3	4
mRNA	MED10	2	mRNA	AP1G2	2	mRNA	SOCS5	4
mRNA	MEF2A	2	mRNA	AP3S1	2	mRNA	SP1	4
mRNA	MEIS1	2	mRNA	APBA2	2	mRNA	SPAG9	4
mRNA	MEST	2	mRNA	APC	2	mRNA	SPIRE1	4
mRNA	MIF4GD	2	mRNA	ARF5	2	mRNA	SPRED1	4
mRNA	MIS18BP1	2	mRNA	ARHGAP10	2	mRNA	SRGAP2	4
mRNA	MLLT3	2	mRNA	ARHGAP32	2	mRNA	SSBP2	4
mRNA	MMD	2	mRNA	ARHGDIB	2	mRNA	SSH1	4
mRNA	MPP6	2	mRNA	ARL6IP5	2	mRNA	SSTR2	4
mRNA	MPPED2	2	mRNA	ARPC5L	2	mRNA	STEAP2	4
mRNA	MREG	2	mRNA	ASIC1	2	mRNA	STK17B	4
mRNA	MRPL35	2	mRNA	ATAD2	2	mRNA	STK24	4
mRNA	MRPS23	2	mRNA	ATAD2B	2	mRNA	SULF2	4
mRNA	MRPS35	2	mRNA	ATL2	2	mRNA	SYPL1	4
mRNA	MRS2	2	mRNA	ATP1B1	2	mRNA	TAB3	4
mRNA	MSRB2	2	mRNA	ATP2A2	2	mRNA	TBC1D12	4
mRNA	MTSS1L	2	mRNA	ATP6V0E2	2	mRNA	TBC1D4	4
mRNA	MYBL1	2	mRNA	ATPAF1	2	mRNA	TCEB3	4
mRNA	MYH9	2	mRNA	AURKB	2	mRNA	TFPI	4
mRNA	MYO1E	2	mRNA	B4GALT5	2	mRNA	TFRC	4
mRNA	MYO5A	2	mRNA	BACE1	2	mRNA	TGFBR2	4
mRNA	NAA50	2	mRNA	BAIAP2	2	mRNA	TMEFF1	4
mRNA	NAGPA	2	mRNA	BAX	2	mRNA	TMEM194A	4
mRNA	NAV1	2	mRNA	BAZ1A	2	mRNA	TMEM2	4
mRNA	NBEAL1	2	mRNA	BCOR	2	mRNA	TMEM87B	4
mRNA	NDUFA5	2	mRNA	BEND7	2	mRNA	TMPO	4
mRNA	NEFH	2	mRNA	BLM	2	mRNA	TNFAIP3	4
mRNA	NEGR1	2	mRNA	BRCA1	2	mRNA	TOP2A	4
mRNA	NEK9	2	mRNA	BRCA2	2	mRNA	TOPBP1	4
mRNA	NFAT5	2	mRNA	BRD2	2	mRNA	TOR1A	4
mRNA	NIPAL4	2	mRNA	BRPF3	2	mRNA	TRIB1	4
mRNA	NKIRAS1	2	mRNA	BUB1	2	mRNA	TRIB2	4
mRNA	NME4	2	mRNA	BUB3	2	mRNA	TRIM36	4
mRNA	NRP2	2	mRNA	C20orf194	2	mRNA	TRUB1	4
mRNA	NUDCD3	2	mRNA	C9orf40	2	mRNA	TSHZ1	4

mRNA	NUMB	2
mRNA	NUP205	2
mRNA	NUP88	2
mRNA	NVL	2
mRNA	ORAI3	2
mRNA	P4HA1	2
mRNA	PAK1IP1	2
mRNA	PARP16	2
mRNA	PATZ1	2
mRNA	PBX3	2
mRNA	PCMTD1	2
mRNA	PCMTD2	2
mRNA	PEBP1	2
mRNA	PGAP2	2
mRNA	PHF6	2
mRNA	PHYH	2
mRNA	PIK3C2B	2
mRNA	PIK3CA	2
mRNA	PIK3R1	2
mRNA	PINK1	2
mRNA	PKP4	2
mRNA	PLAA	2
mRNA	PLEKHA6	2
mRNA	PLEKHB1	2
mRNA	POLQ	2
mRNA	POLR3G	2
mRNA	PPARD	2
mRNA	PPAT	2
mRNA	PRDM5	2
mRNA	PRMT2	2
mRNA	PRMT5	2
mRNA	PRR15	2
mRNA	PSAT1	2
mRNA	PSIP1	2
mRNA	PTEN	2
mRNA	PTGER4	2
mRNA	PTPN1	2
mRNA	PTPN12	2
mRNA	PTPN13	2
mRNA	QDPR	2
mRNA	RAB12	2
mRNA	RABGAP1	2
mRNA	RABGAP1L	2
mRNA	RALB	2
mRNA	RALGPS2	2
mRNA	RANBP17	2
mRNA	RAPGEF1	2
mRNA	RASGEF1B	2
mRNA	RAVER2	2
mRNA	RBM34	2
mRNA	RBM8A	2
mRNA	RELL1	2
mRNA	RGMA	2
mRNA	CA2	2
mRNA	CADM1	2
mRNA	CALD1	2
mRNA	CALU	2
mRNA	CAMK1D	2
mRNA	CAMK2D	2
mRNA	CASK	2
mRNA	CCDC138	2
mRNA	CCDC34	2
mRNA	CCDC43	2
mRNA	CCDC71L	2
mRNA	CCDC8	2
mRNA	CCDC85C	2
mRNA	CCNA2	2
mRNA	CCNJL	2
mRNA	CCT2	2
mRNA	CD164	2
mRNA	CD276	2
mRNA	CD83	2
mRNA	CDCA5	2
mRNA	CDH1	2
mRNA	CEBPG	2
mRNA	CHM	2
mRNA	CHMP4C	2
mRNA	CHUK	2
mRNA	CIDEB	2
mRNA	CKAP2	2
mRNA	CKB	2
mRNA	CLDND1	2
mRNA	CLEC2D	2
mRNA	CLIP1	2
mRNA	CLIP4	2
mRNA	CLN6	2
mRNA	CNNM3	2
mRNA	CPS1	2
mRNA	CRBN	2
mRNA	CREBBP	2
mRNA	CREBRF	2
mRNA	CREM	2
mRNA	CRIM1	2
mRNA	CROT	2
mRNA	CRTAP	2
mRNA	CSTF3	2
mRNA	CTPS2	2
mRNA	CTSD	2
mRNA	CUX1	2
mRNA	CYP2S1	2
mRNA	CYP2U1	2
mRNA	DCBLD2	2
mRNA	DCLRE1B	2
mRNA	DDB2	2
mRNA	DDX52	2
mRNA	DHRS4L2	2
mRNA	UBE2H	4
mRNA	UBR1	4
mRNA	UGCG	4
mRNA	USP16	4
mRNA	UTP14A	4
mRNA	VCAN	4
mRNA	VKORC1L1	4
mRNA	WASF1	4
mRNA	WDFY3	4
mRNA	WDR47	4
mRNA	YPEL2	4
mRNA	YPEL5	4
mRNA	ZBTB43	4
mRNA	ZC3H6	4
mRNA	ZDBF2	4
mRNA	ZER1	4
mRNA	ZHX1	4
mRNA	ZNF217	4
mRNA	ZNF362	4
mRNA	ZNF652	4
mRNA	ZNRF2	4
mRNA	AAGAB	3
mRNA	AAK1	3
mRNA	ABCB10	3
mRNA	ADAM10	3
mRNA	ADAM12	3
mRNA	AFF3	3
mRNA	ALAS1	3
mRNA	ALDH1A2	3
mRNA	AMPD3	3
mRNA	ANTXR1	3
mRNA	AP3S1	3
mRNA	APOO	3
mRNA	ARHGAP12	3
mRNA	ARHGEF3	3
mRNA	ARPC5L	3
mRNA	ATAD2	3
mRNA	ATG14	3
mRNA	ATP2B4	3
mRNA	ATP6V0E2	3
mRNA	ATPAF1	3
mRNA	B4GALT4	3
mRNA	BACE1	3
mRNA	BACH2	3
mRNA	BARD1	3
mRNA	BEND7	3
mRNA	BMF	3
mRNA	BMP6	3
mRNA	BRD2	3
mRNA	BRE	3
mRNA	BRPF3	3
mRNA	BTAF1	3
mRNA	BTG1	3

mRNA	RHOB	2
mRNA	RHOBTB1	2
mRNA	RNF19B	2
mRNA	RPS6KA2	2
mRNA	RSBN1	2
mRNA	RUNX2	2
mRNA	SAP30BP	2
mRNA	SASS6	2
mRNA	SCPEP1	2
mRNA	SDCCAG8	2
mRNA	SEMA3C	2
mRNA	SENP5	2
mRNA	SEPN1	2
mRNA	8-Sep	2
mRNA	SESN3	2
mRNA	SFMBT1	2
mRNA	SFXN2	2
mRNA	SGK1	2
mRNA	SH2D4A	2
mRNA	SHB	2
mRNA	SIDT1	2
mRNA	SLC20A2	2
mRNA	SLC2A13	2
mRNA	SLC35F1	2
mRNA	SMAD6	2
mRNA	SMARCA1	2
mRNA	SMYD2	2
mRNA	SPAG9	2
mRNA	SPIN4	2
mRNA	SPIRE1	2
mRNA	SSBP3	2
mRNA	ST8SIA4	2
mRNA	STK24	2
mRNA	STK32B	2
mRNA	STOM	2
mRNA	STRADB	2
mRNA	SUFU	2
mRNA	SYAP1	2
mRNA	TBC1D9	2
mRNA	TET3	2
mRNA	TFPI2	2
mRNA	TIMP2	2
mRNA	TMC7	2
mRNA	TMEFF1	2
mRNA	TMEM109	2
mRNA	TMEM19	2
mRNA	TMEM25	2
mRNA	TMEM38B	2
mRNA	TMEM39B	2
mRNA	TMEM57	2
mRNA	TNFRSF10B	2
mRNA	TOB1	2
mRNA	TOMM22	2
mRNA	DHX15	2
mRNA	DIS3L	2
mRNA	DOCK4	2
mRNA	DPY19L3	2
mRNA	DPYSL2	2
mRNA	DUSP7	2
mRNA	DYRK1B	2
mRNA	E2F2	2
mRNA	E2F7	2
mRNA	ECT2	2
mRNA	EHD1	2
mRNA	EIF2AK4	2
mRNA	EIF2S2	2
mRNA	EIF3J	2
mRNA	ELOVL6	2
mRNA	ENC1	2
mRNA	ENPP2	2
mRNA	ENPP4	2
mRNA	EPHB4	2
mRNA	EPT1	2
mRNA	ERG	2
mRNA	ERI1	2
mRNA	ERICH1	2
mRNA	ERLIN2	2
mRNA	ETAA1	2
mRNA	ETF1	2
mRNA	ETV5	2
mRNA	EXOSC5	2
mRNA	FAM188A	2
mRNA	FAM50B	2
mRNA	FAM69B	2
mRNA	FAM78A	2
mRNA	FAM83D	2
mRNA	FANCA	2
mRNA	FANCD2	2
mRNA	FANCM	2
mRNA	FASTKD5	2
mRNA	FBXO4	2
mRNA	FCHO1	2
mRNA	FERMT1	2
mRNA	FERMT2	2
mRNA	FGD4	2
mRNA	FKBP9	2
mRNA	FKTN	2
mRNA	FLI1	2
mRNA	FN3KRP	2
mRNA	FNDC3B	2
mRNA	FSD1	2
mRNA	GALK1	2
mRNA	GATA6	2
mRNA	GCOM1	2
mRNA	GGCT	2
mRNA	GINS1	2
mRNA	BTN3A1	3
mRNA	BTN3A2	3
mRNA	C16orf70	3
mRNA	C16orf87	3
mRNA	C1orf21	3
mRNA	C20orf194	3
mRNA	C2CD2	3
mRNA	CA2	3
mRNA	CALD1	3
mRNA	CAMLG	3
mRNA	CASK	3
mRNA	CASP4	3
mRNA	CASZ1	3
mRNA	CCDC28A	3
mRNA	CCDC50	3
mRNA	CCDC85C	3
mRNA	CCM2	3
mRNA	CCNE2	3
mRNA	CCT2	3
mRNA	CD47	3
mRNA	CD69	3
mRNA	CDADC1	3
mRNA	CDC42EP2	3
mRNA	CDK1	3
mRNA	CDK5	3
mRNA	CDKN1B	3
mRNA	CENPQ	3
mRNA	CHEK1	3
mRNA	CHM	3
mRNA	CHRNA5	3
mRNA	CIT	3
mRNA	CKAP2	3
mRNA	CLIC4	3
mRNA	CLIP4	3
mRNA	CNKSR3	3
mRNA	CNOT6L	3
mRNA	CRAMP1L	3
mRNA	CRBN	3
mRNA	CREBBP	3
mRNA	CREBRF	3
mRNA	CRTAP	3
mRNA	CTNNBIP1	3
mRNA	CTTNBP2NL	3
mRNA	CYB5D1	3
mRNA	DAB2	3
mRNA	DCUN1D5	3
mRNA	DDX52	3
mRNA	DEK	3
mRNA	DENND5A	3
mRNA	DEPDC1	3
mRNA	DGKD	3
mRNA	DIP2C	3
mRNA	DIRC2	3

mRNA	TPP1	2
mRNA	TRAPPC8	2
mRNA	TRERF1	2
mRNA	TRIAP1	2
mRNA	TRIM59	2
mRNA	TRIO	2
mRNA	TRIP10	2
mRNA	TSR1	2
mRNA	TUBE1	2
mRNA	TXNDC9	2
mRNA	UBE2E2	2
mRNA	UBE2G2	2
mRNA	UBE2H	2
mRNA	UBE2K	2
mRNA	UHRF1BP1	2
mRNA	UQCRB	2
mRNA	USP46	2
mRNA	UTRN	2
mRNA	VEGFA	2
mRNA	VGLL4	2
mRNA	VKORC1L1	2
mRNA	VPS26B	2
mRNA	WDR43	2
mRNA	WEE1	2
mRNA	XPO7	2
mRNA	YTHDF2	2
mRNA	ZBTB38	2
mRNA	ZDHHC1	2
mRNA	ZHX2	2
mRNA	ZNF397	2
mRNA	ZNF607	2
mRNA	ZNF624	2
mRNA	ZSWIM6	2
mRNA	ZW10	2
mRNA	ZXDA	2
mRNA	ADAM28	2
mRNA	BEND5	2
mRNA	CHEK2	2
mRNA	EXO5	2
mRNA	FAM229B	2
mRNA	RPF2	2
mRNA	RSRP1	2
mRNA	TPTE2	2
mRNA	XIRP2	2
mRNA	AAK1	1
mRNA	AATF	1
mRNA	ABCA5	1
mRNA	ABCB6	1
mRNA	ABHD14A	1
mRNA	ABHD4	1
mRNA	ABHD5	1
mRNA	ABHD6	1
mRNA	ABTB2	1
mRNA	GLIS3	2
mRNA	GLRX	2
mRNA	GLTP	2
mRNA	GMCL1	2
mRNA	GMFB	2
mRNA	GPR137C	2
mRNA	GTSE1	2
mRNA	GUCY1A3	2
mRNA	HBEGF	2
mRNA	HECA	2
mRNA	HECTD3	2
mRNA	HGF	2
mRNA	HMGB1	2
mRNA	HMGXB4	2
mRNA	HOMER1	2
mRNA	HOXA9	2
mRNA	HSPB11	2
mRNA	HTRA3	2
mRNA	ICMT	2
mRNA	IFI16	2
mRNA	IGF1R	2
mRNA	IL12A	2
mRNA	IL15	2
mRNA	INPP5K	2
mRNA	INSIG1	2
mRNA	INSR	2
mRNA	IQGAP3	2
mRNA	IRF4	2
mRNA	ITGA4	2
mRNA	ITGAV	2
mRNA	ITPRIP	2
mRNA	JMJD8	2
mRNA	KAL1	2
mRNA	KBTBD6	2
mRNA	KBTBD8	2
mRNA	KIAA1147	2
mRNA	KIF13A	2
mRNA	KIF24	2
mRNA	KIF2C	2
mRNA	KLF11	2
mRNA	KLF5	2
mRNA	KLHL2	2
mRNA	KLHL3	2
mRNA	LAMC1	2
mRNA	LARP6	2
mRNA	LCLAT1	2
mRNA	LIMCH1	2
mRNA	LIN9	2
mRNA	LMBR1L	2
mRNA	LMNB1	2
mRNA	LPP	2
mRNA	LRCH2	2
mRNA	LRIG1	2
mRNA	DNAJC12	3
mRNA	DNAJC18	3
mRNA	DNAJC7	3
mRNA	DOC2A	3
mRNA	DSC2	3
mRNA	DTL	3
mRNA	DUSP22	3
mRNA	DUSP8	3
mRNA	DYNLT1	3
mRNA	E2F3	3
mRNA	E2F7	3
mRNA	EDA2R	3
mRNA	EDARADD	3
mRNA	EDEM1	3
mRNA	EFNA3	3
mRNA	EGLN3	3
mRNA	EGR2	3
mRNA	EIF2B2	3
mRNA	EIF3J	3
mRNA	ELOVL6	3
mRNA	EML4	3
mRNA	ENAH	3
mRNA	ENPP2	3
mRNA	EPN2	3
mRNA	EPS8	3
mRNA	EPSTI1	3
mRNA	ERMP1	3
mRNA	ERO1L	3
mRNA	ETS1	3
mRNA	EXOC4	3
mRNA	FADD	3
mRNA	FAM102A	3
mRNA	FAM102B	3
mRNA	FAM107B	3
mRNA	FAM129A	3
mRNA	FAM134A	3
mRNA	FAM50B	3
mRNA	FAM81A	3
mRNA	FANCB	3
mRNA	FANCI	3
mRNA	FASTKD5	3
mRNA	FAT4	3
mRNA	FER	3
mRNA	FHL1	3
mRNA	FKTN	3
mRNA	FLI1	3
mRNA	FLNB	3
mRNA	FNBP1	3
mRNA	FOSB	3
mRNA	FSD1L	3
mRNA	FZD3	3
mRNA	FZD5	3
mRNA	GALNT10	3

mRNA	ACAA2	1	mRNA	LRP1	2	mRNA	GATA3	3
mRNA	ACOT9	1	mRNA	LSM5	2	mRNA	GBE1	3
mRNA	ACSS1	1	mRNA	LYRM5	2	mRNA	GCOM1	3
mRNA	ACTA2	1	mRNA	MAMLD1	2	mRNA	GFPT2	3
mRNA	ACTN1	1	mRNA	MAP3K8	2	mRNA	GGCT	3
mRNA	ADAM10	1	mRNA	MAP4	2	mRNA	GGH	3
mRNA	ADAM15	1	mRNA	MARCKS	2	mRNA	GLCE	3
mRNA	ADCY1	1	mRNA	MBOAT1	2	mRNA	GLRX3	3
mRNA	ADORA2B	1	mRNA	MCM3	2	mRNA	GMFB	3
mRNA	ADRA2A	1	mRNA	MED12L	2	mRNA	GOLPH3	3
mRNA	AEN	1	mRNA	MED15	2	mRNA	GPR124	3
mRNA	AGFG2	1	mRNA	MED16	2	mRNA	GPR137B	3
mRNA	AGMAT	1	mRNA	MEIS1	2	mRNA	GPR137C	3
mRNA	AGTRAP	1	mRNA	MEX3D	2	mRNA	GPRIN3	3
mRNA	AHDC1	1	mRNA	MIF4GD	2	mRNA	GPSM2	3
mRNA	AHI1	1	mRNA	MIS18BP1	2	mRNA	GSTCD	3
mRNA	AHNAK	1	mRNA	MLEC	2	mRNA	GTF2F2	3
mRNA	AIF1L	1	mRNA	MN1	2	mRNA	GTPBP8	3
mRNA	AIFM2	1	mRNA	MORN2	2	mRNA	HAS2	3
mRNA	AIM1	1	mRNA	MREG	2	mRNA	HAT1	3
mRNA	AKAP1	1	mRNA	MRPL37	2	mRNA	HCFC2	3
mRNA	AKIRIN1	1	mRNA	MRS2	2	mRNA	HECA	3
mRNA	ALAS1	1	mRNA	MSH2	2	mRNA	HERPUD1	3
mRNA	ALDH3B1	1	mRNA	MTHFD2	2	mRNA	HMGB3	3
mRNA	ALDH6A1	1	mRNA	MTL5	2	mRNA	HMGXB4	3
mRNA	ALG1	1	mRNA	MTMR9	2	mRNA	HN1L	3
mRNA	ALG2	1	mRNA	MXI1	2	mRNA	HNRNPAB	3
mRNA	AMACR	1	mRNA	MYBL2	2	mRNA	HOXA10	3
mRNA	AMIGO2	1	mRNA	MYCN	2	mRNA	HS3ST3B1	3
mRNA	ANAPC10	1	mRNA	MYH9	2	mRNA	HS6ST2	3
mRNA	ANKAR	1	mRNA	MYO1G	2	mRNA	HSPA13	3
mRNA	ANKRD46	1	mRNA	NAA15	2	mRNA	HSPD1	3
mRNA	ANXA1	1	mRNA	NAA16	2	mRNA	HTRA3	3
mRNA	ANXA2	1	mRNA	NASP	2	mRNA	ICMT	3
mRNA	AP1S3	1	mRNA	NBEA	2	mRNA	IER3	3
mRNA	AP3D1	1	mRNA	NCAPD2	2	mRNA	IGFBP7	3
mRNA	AP4E1	1	mRNA	NCAPG	2	mRNA	IL6	3
mRNA	APIP	1	mRNA	NCKAP1	2	mRNA	INADL	3
mRNA	APOL2	1	mRNA	NEFH	2	mRNA	INPP1	3
mRNA	ARHGAP10	1	mRNA	NET1	2	mRNA	INSIG1	3
mRNA	ARHGAP20	1	mRNA	NID1	2	mRNA	INSM1	3
mRNA	ARHGAP26	1	mRNA	NIPAL3	2	mRNA	IQGAP2	3
mRNA	ARHGDIB	1	mRNA	NIPSNAP1	2	mRNA	IQSEC1	3
mRNA	ARHGEF10	1	mRNA	NOP2	2	mRNA	IRF1	3
mRNA	ARHGEF2	1	mRNA	NPEPL1	2	mRNA	ISCU	3
mRNA	ARL2	1	mRNA	NPLOC4	2	mRNA	ITGA6	3
mRNA	ARL5A	1	mRNA	NR4A1	2	mRNA	ITGAV	3
mRNA	ARMCX6	1	mRNA	NRIP1	2	mRNA	ITPRIP	3
mRNA	ARPC5L	1	mRNA	NSUN7	2	mRNA	IVNS1ABP	3
mRNA	ARRDC4	1	mRNA	NUCB2	2	mRNA	JUP	3
mRNA	ASAH1	1	mRNA	NUDT8	2	mRNA	KBTBD6	3
mRNA	ASAP2	1	mRNA	NUP62CL	2	mRNA	KCNK5	3
mRNA	ASB13	1	mRNA	NUS1	2	mRNA	KCTD20	3

mRNA	ASPHD1	1	mRNA	NYNRIN	2	mRNA	KCTD3	3
mRNA	ATAD2	1	mRNA	OGG1	2	mRNA	KCTD9	3
mRNA	ATG4C	1	mRNA	OSBPL1A	2	mRNA	KDELC2	3
mRNA	ATL2	1	mRNA	OTUD1	2	mRNA	KDM2B	3
mRNA	ATM	1	mRNA	OTUD6B	2	mRNA	KIAA0895	3
mRNA	ATP13A3	1	mRNA	OXA1L	2	mRNA	KIAA1161	3
mRNA	ATP2A2	1	mRNA	P4HA1	2	mRNA	KIAA1462	3
mRNA	ATP6V0E2	1	mRNA	PALLD	2	mRNA	KIAA1524	3
mRNA	ATP7B	1	mRNA	PAM	2	mRNA	KIF21A	3
mRNA	ATP8A1	1	mRNA	PAPD5	2	mRNA	KIF5C	3
mRNA	ATP9A	1	mRNA	PARK2	2	mRNA	KPNA2	3
mRNA	B3GNT5	1	mRNA	PAXIP1	2	mRNA	LAMP3	3
mRNA	BAG2	1	mRNA	PCGF2	2	mRNA	LIMA1	3
mRNA	BAX	1	mRNA	PCMTD1	2	mRNA	LIMCH1	3
mRNA	BBS2	1	mRNA	PCTP	2	mRNA	LMBR1L	3
mRNA	BCL9L	1	mRNA	PDE4B	2	mRNA	LMNB1	3
mRNA	BCOR	1	mRNA	PDGFD	2	mRNA	LOX	3
mRNA	BDH1	1	mRNA	PDK1	2	mRNA	LRRC16A	3
mRNA	BIN1	1	mRNA	PER3	2	mRNA	LYRM1	3
mRNA	BIRC2	1	mRNA	PGAP2	2	mRNA	MAGT1	3
mRNA	BLOC1S1	1	mRNA	PGBD2	2	mRNA	MAN1A1	3
mRNA	BMP4	1	mRNA	PHF5A	2	mRNA	MAP3K13	3
mRNA	BMP8B	1	mRNA	PHF6	2	mRNA	MAP3K8	3
mRNA	BMPR1B	1	mRNA	PIBF1	2	mRNA	MASTL	3
mRNA	BRCA2	1	mRNA	PICALM	2	mRNA	ME1	3
mRNA	BRD2	1	mRNA	PINK1	2	mRNA	MECOM	3
mRNA	BRI3BP	1	mRNA	PKP4	2	mRNA	MELK	3
mRNA	BTG1	1	mRNA	PLK2	2	mRNA	MEST	3
mRNA	BTN3A2	1	mRNA	POLQ	2	mRNA	METTL2A	3
mRNA	BUB1	1	mRNA	PPAP2B	2	mRNA	MFSD2A	3
mRNA	BUB1B	1	mRNA	PPM1G	2	mRNA	MGLL	3
mRNA	BUB3	1	mRNA	PPM1H	2	mRNA	MKNK1	3
mRNA	C11orf74	1	mRNA	PPP2R5C	2	mRNA	MLLT3	3
mRNA	C12orf5	1	mRNA	PREPL	2	mRNA	MMD	3
mRNA	C16orf72	1	mRNA	PRICKLE1	2	mRNA	MORN2	3
mRNA	C17orf75	1	mRNA	PRIM1	2	mRNA	MPDZ	3
mRNA	C18orf54	1	mRNA	PRR11	2	mRNA	MPP6	3
mRNA	C1orf112	1	mRNA	PRR16	2	mRNA	MREG	3
mRNA	C1orf74	1	mRNA	PRRG1	2	mRNA	MRPL37	3
mRNA	C2CD2	1	mRNA	PSD3	2	mRNA	MRPS35	3
mRNA	C3orf58	1	mRNA	PTPDC1	2	mRNA	MRRF	3
mRNA	C9orf40	1	mRNA	PTPRJ	2	mRNA	MT1X	3
mRNA	CA8	1	mRNA	PUS7	2	mRNA	MTHFD2	3
mRNA	CAB39L	1	mRNA	QDPR	2	mRNA	MXD1	3
mRNA	CADM1	1	mRNA	RAB11FIP1	2	mRNA	MXI1	3
mRNA	CAMK2D	1	mRNA	RAB11FIP4	2	mRNA	MYBL1	3
mRNA	CARD6	1	mRNA	RAB12	2	mRNA	MYCN	3
mRNA	CASK	1	mRNA	RAB5B	2	mRNA	MYO18A	3
mRNA	CASP4	1	mRNA	RACGAP1	2	mRNA	MYO19	3
mRNA	CAST	1	mRNA	RAD51AP1	2	mRNA	NAA16	3
mRNA	CAT	1	mRNA	RAD51C	2	mRNA	NAA50	3
mRNA	CBLB	1	mRNA	RALGPS2	2	mRNA	NAE1	3
mRNA	CBS	1	mRNA	RAPGEFL1	2	mRNA	NAPG	3

mRNA	CBX2	1	mRNA	RAPH1	2	mRNA	NCKAP1	3
mRNA	CCDC126	1	mRNA	RBM47	2	mRNA	NDFIP1	3
mRNA	CCDC28A	1	mRNA	RBM8A	2	mRNA	NDUFA5	3
mRNA	CCDC43	1	mRNA	RCAN3	2	mRNA	NETO2	3
mRNA	CCDC50	1	mRNA	RCOR1	2	mRNA	NFAT5	3
mRNA	CCDC53	1	mRNA	RELL1	2	mRNA	NFE2L1	3
mRNA	CCDC8	1	mRNA	REPS2	2	mRNA	NFKBIA	3
mRNA	CCDC85C	1	mRNA	RERE	2	mRNA	NLGN1	3
mRNA	CCM2	1	mRNA	RFC3	2	mRNA	NOG	3
mRNA	CCNE2	1	mRNA	RFC4	2	mRNA	NPC2	3
mRNA	CCNF	1	mRNA	RNF150	2	mRNA	NPR3	3
mRNA	CCNJL	1	mRNA	RNF187	2	mRNA	NRIP1	3
mRNA	CCNYL1	1	mRNA	RNF44	2	mRNA	NRP2	3
mRNA	CCP110	1	mRNA	RPGRIP1L	2	mRNA	NSMCE2	3
mRNA	CCT2	1	mRNA	RPL13	2	mRNA	NUDCD2	3
mRNA	CCT5	1	mRNA	RPS6KA4	2	mRNA	NUP205	3
mRNA	CD37	1	mRNA	RRAGD	2	mRNA	NUP210	3
mRNA	CD47	1	mRNA	RSBN1	2	mRNA	NUP35	3
mRNA	CD79A	1	mRNA	RTKN	2	mRNA	PANK1	3
mRNA	CD83	1	mRNA	RUNX1T1	2	mRNA	PANX1	3
mRNA	CDADC1	1	mRNA	RUNX3	2	mRNA	PARP9	3
mRNA	CDCA5	1	mRNA	RWDD2B	2	mRNA	PAXIP1	3
mRNA	CDCA7	1	mRNA	S1PR1	2	mRNA	PBXIP1	3
mRNA	CDCA7L	1	mRNA	SAMD8	2	mRNA	PCGF2	3
mRNA	CDCA8	1	mRNA	SASS6	2	mRNA	PCTP	3
mRNA	CDK1	1	mRNA	SCARB1	2	mRNA	PDCD4	3
mRNA	CDK13	1	mRNA	SCARB2	2	mRNA	PDE4DIP	3
mRNA	CDK4	1	mRNA	SEH1L	2	mRNA	PDE8A	3
mRNA	CELSR3	1	mRNA	SEMA3A	2	mRNA	PDIK1L	3
mRNA	CENPK	1	mRNA	SEMA6A	2	mRNA	PDK4	3
mRNA	CENPO	1	mRNA	6-Sep	2	mRNA	PFKFB3	3
mRNA	CENPP	1	mRNA	8-Sep	2	mRNA	PGM3	3
mRNA	CEP128	1	mRNA	SERPINB8	2	mRNA	PHF5A	3
mRNA	CEP41	1	mRNA	SFXN1	2	mRNA	PITPNC1	3
mRNA	CEP68	1	mRNA	SFXN3	2	mRNA	PLAA	3
mRNA	CERCAM	1	mRNA	SGK3	2	mRNA	PLEKHA1	3
mRNA	CHAF1A	1	mRNA	SGMS1	2	mRNA	PLEKHA2	3
mRNA	CHD7	1	mRNA	SHB	2	mRNA	PLEKHA6	3
mRNA	CHMP4C	1	mRNA	SHCBP1	2	mRNA	PNRC1	3
mRNA	CHN1	1	mRNA	SHISA5	2	mRNA	PPARD	3
mRNA	CHUK	1	mRNA	SKAP2	2	mRNA	PPIF	3
mRNA	CIDEB	1	mRNA	SLAMF7	2	mRNA	PPIH	3
mRNA	CKAP2	1	mRNA	SLC16A9	2	mRNA	PRDM2	3
mRNA	CKS2	1	mRNA	SLC24A1	2	mRNA	PRKRA	3
mRNA	CLU	1	mRNA	SLC25A24	2	mRNA	PRR5L	3
mRNA	CMTM7	1	mRNA	SLC39A14	2	mRNA	PSAT1	3
mRNA	CNKSR3	1	mRNA	SLC4A7	2	mRNA	PSMB5	3
mRNA	CNPY4	1	mRNA	SLC6A9	2	mRNA	PSMD12	3
mRNA	COL15A1	1	mRNA	SLTM	2	mRNA	PSMD14	3
mRNA	COL24A1	1	mRNA	SMAD6	2	mRNA	PSME4	3
mRNA	COPB1	1	mRNA	SMAD7	2	mRNA	PTDSS1	3
mRNA	COPS3	1	mRNA	SMC2	2	mRNA	PTGER4	3
mRNA	COX11	1	mRNA	SNX25	2	mRNA	PTHLH	3

mRNA	CPA3	1	mRNA	SOBP	2	mRNA	PTPN14	3
mRNA	CPSF3	1	mRNA	SORL1	2	mRNA	PTPRA	3
mRNA	CPVL	1	mRNA	SPAG9	2	mRNA	PTPRJ	3
mRNA	CRAMP1L	1	mRNA	SPOCK3	2	mRNA	PVR	3
mRNA	CREB3L4	1	mRNA	SPRED1	2	mRNA	RAB11FIP5	3
mRNA	CREBRF	1	mRNA	SPTY2D1	2	mRNA	RAB4B	3
mRNA	CREG1	1	mRNA	SSBP2	2	mRNA	RACGAP1	3
mRNA	CROT	1	mRNA	ST8SIA4	2	mRNA	RAD18	3
mRNA	CSRNP1	1	mRNA	STEAP2	2	mRNA	RAD51AP1	3
mRNA	CSTF3	1	mRNA	STK39	2	mRNA	RANBP17	3
mRNA	CTSF	1	mRNA	STRADB	2	mRNA	RASSF5	3
mRNA	CTSS	1	mRNA	SUMO1	2	mRNA	RBM8A	3
mRNA	CTTNBP2NL	1	mRNA	SYDE2	2	mRNA	RBMS3	3
mRNA	CYB5D1	1	mRNA	TACC1	2	mRNA	RBPMS	3
mRNA	CYB5D2	1	mRNA	TAF13	2	mRNA	RCSD1	3
mRNA	CYP2S1	1	mRNA	TARBP1	2	mRNA	RDH10	3
mRNA	CYP2U1	1	mRNA	TBC1D12	2	mRNA	RERE	3
mRNA	CYP4V2	1	mRNA	TBC1D2	2	mRNA	REXO1	3
mRNA	DAB2	1	mRNA	TBCEL	2	mRNA	RFC3	3
mRNA	DAG1	1	mRNA	TCERG1	2	mRNA	RFK	3
mRNA	DAPP1	1	mRNA	TCP11L2	2	mRNA	RHOBTB3	3
mRNA	DCAF17	1	mRNA	TEX2	2	mRNA	RHOU	3
mRNA	DCBLD2	1	mRNA	TFPI2	2	mRNA	RIOK3	3
mRNA	DCK	1	mRNA	TFRC	2	mRNA	RNF115	3
mRNA	DCLK2	1	mRNA	THEM4	2	mRNA	RNF213	3
mRNA	DCLRE1B	1	mRNA	TIMP2	2	mRNA	RPAP3	3
mRNA	DCUN1D3	1	mRNA	TK1	2	mRNA	RPL13	3
mRNA	DDX21	1	mRNA	TMEM109	2	mRNA	RPL22	3
mRNA	DDX52	1	mRNA	TMEM134	2	mRNA	RPS6KA4	3
mRNA	DENND5A	1	mRNA	TMEM141	2	mRNA	RPSA	3
mRNA	DFFB	1	mRNA	TMEM19	2	mRNA	RRM1	3
mRNA	DGKD	1	mRNA	TMEM194A	2	mRNA	RUNDC3B	3
mRNA	DHRS4L2	1	mRNA	TMEM50B	2	mRNA	RUNX1T1	3
mRNA	DHX15	1	mRNA	TMEM63B	2	mRNA	RUNX3	3
mRNA	DIS3L	1	mRNA	TNFAIP8	2	mRNA	S1PR1	3
mRNA	DIXDC1	1	mRNA	TNFSF10	2	mRNA	SDC4	3
mRNA	DKC1	1	mRNA	TNFSF12	2	mRNA	SEC14L1	3
mRNA	DNAJA4	1	mRNA	TOP2A	2	mRNA	SEMA6A	3
mRNA	DNAJB12	1	mRNA	TOR1A	2	mRNA	SESN2	3
mRNA	DNAJC12	1	mRNA	TPX2	2	mRNA	SESN3	3
mRNA	DNAJC7	1	mRNA	TRAF3IP2	2	mRNA	SFXN1	3
mRNA	DNMT1	1	mRNA	TRIAP1	2	mRNA	SFXN2	3
mRNA	DOC2A	1	mRNA	TRIB2	2	mRNA	SGMS1	3
mRNA	DOCK4	1	mRNA	TSEN15	2	mRNA	SHISA5	3
mRNA	DOCK9	1	mRNA	TSPO	2	mRNA	SIK1	3
mRNA	DPP9	1	mRNA	TTYH3	2	mRNA	SKIL	3
mRNA	DPY19L3	1	mRNA	TYSND1	2	mRNA	SLC11A2	3
mRNA	DSCC1	1	mRNA	UBA2	2	mRNA	SLC20A2	3
mRNA	DTL	1	mRNA	UBE2C	2	mRNA	SLC30A7	3
mRNA	DTNA	1	mRNA	UBE2G2	2	mRNA	SLC31A1	3
mRNA	DUSP22	1	mRNA	UBE2V2	2	mRNA	SLC35E1	3
mRNA	DUT	1	mRNA	UBR1	2	mRNA	SLC41A2	3
mRNA	DZIP1	1	mRNA	UHRF1	2	mRNA	SLC44A5	3

mRNA	E2F7	1	mRNA	UNG	2	mRNA	SLC46A3	3
mRNA	E2F8	1	mRNA	VAMP4	2	mRNA	SLC9A8	3
mRNA	EDARADD	1	mRNA	VCAN	2	mRNA	SMAD3	3
mRNA	EEPD1	1	mRNA	VGLL4	2	mRNA	SMAD7	3
mRNA	EFNA4	1	mRNA	VRK1	2	mRNA	SNRNP40	3
mRNA	EGLN1	1	mRNA	WDR5	2	mRNA	SNX11	3
mRNA	EGR2	1	mRNA	WIBG	2	mRNA	SPATS2	3
mRNA	EHD1	1	mRNA	WNK3	2	mRNA	SPIN4	3
mRNA	EHD2	1	mRNA	XPOT	2	mRNA	SPRYD3	3
mRNA	EIF5	1	mRNA	XYLT1	2	mRNA	SPTBN1	3
mRNA	EIF5B	1	mRNA	YEATS4	2	mRNA	SPTLC2	3
mRNA	ELL2	1	mRNA	YPEL5	2	mRNA	SRGAP1	3
mRNA	EML4	1	mRNA	ZBTB20	2	mRNA	SRRT	3
mRNA	ENAH	1	mRNA	ZBTB38	2	mRNA	STK39	3
mRNA	ENC1	1	mRNA	ZC3HAV1L	2	mRNA	STOM	3
mRNA	ENPP2	1	mRNA	ZDHHC1	2	mRNA	STRADB	3
mRNA	ENPP4	1	mRNA	ZHX2	2	mRNA	SVIL	3
mRNA	EPB41L3	1	mRNA	ZHX3	2	mRNA	SYBU	3
mRNA	EPCAM	1	mRNA	ZKSCAN4	2	mRNA	SYDE2	3
mRNA	EPHA2	1	mRNA	ZNF138	2	mRNA	SYT1	3
mRNA	EPHB4	1	mRNA	ZNF2	2	mRNA	TAF13	3
mRNA	EPN2	1	mRNA	ZNF397	2	mRNA	TARS	3
mRNA	EPS8	1	mRNA	ZNF485	2	mRNA	TBC1D8	3
mRNA	EPT1	1	mRNA	ZNF516	2	mRNA	TBC1D9	3
mRNA	ERI1	1	mRNA	ZNF566	2	mRNA	TBPL1	3
mRNA	ERI2	1	mRNA	ZNF609	2	mRNA	TCERG1	3
mRNA	ERI3	1	mRNA	ZNF681	2	mRNA	TDG	3
mRNA	ERMP1	1	mRNA	ZNF695	2	mRNA	TDRD7	3
mRNA	ERO1L	1	mRNA	ZNF738	2	mRNA	TEX2	3
mRNA	ERP29	1	mRNA	ZNF75A	2	mRNA	TFB2M	3
mRNA	ESYT2	1	mRNA	ZNF77	2	mRNA	TFPI2	3
mRNA	ETF1	1	mRNA	ZNRF1	2	mRNA	THAP11	3
mRNA	ETFB	1	mRNA	ZXDA	2	mRNA	THBS1	3
mRNA	EXO1	1	mRNA	ADAM28	2	mRNA	THEM4	3
mRNA	EXOC4	1	mRNA	AGER	2	mRNA	TIAM1	3
mRNA	EXTL2	1	mRNA	BSPRY	2	mRNA	TJP2	3
mRNA	EZH1	1	mRNA	CLUH	2	mRNA	TMC7	3
mRNA	FAM114A1	1	mRNA	CRHBP	2	mRNA	TMCC3	3
mRNA	FAM124A	1	mRNA	GLI1	2	mRNA	TMEM161B	3
mRNA	FAM127C	1	mRNA	LILRA2	2	mRNA	TMEM165	3
mRNA	FAM133A	1	mRNA	PRIMPOL	2	mRNA	TMEM19	3
mRNA	FAM160B1	1	mRNA	S100B	2	mRNA	TMEM25	3
mRNA	FAM161A	1	mRNA	TCAF2	2	mRNA	TMEM39B	3
mRNA	FAM184A	1	mRNA	TLR6	2	mRNA	TMEM45A	3
mRNA	FAM195A	1	mRNA	XIRP2	2	mRNA	TMEM50B	3
mRNA	FAM198B	1	mRNA	ZNF391	2	mRNA	TMEM57	3
mRNA	FAM200A	1	mRNA	AAGAB	1	mRNA	TMEM63B	3
mRNA	FAM43A	1	mRNA	AATF	1	mRNA	TMEM97	3
mRNA	FAM49A	1	mRNA	ABCB10	1	mRNA	TMTC4	3
mRNA	FAM50B	1	mRNA	ABCB6	1	mRNA	TNFRSF10D	3
mRNA	FAM58A	1	mRNA	ABCD1	1	mRNA	TNFSF10	3
mRNA	FAM63B	1	mRNA	ABHD15	1	mRNA	TNFSF9	3
mRNA	FAM65B	1	mRNA	ABHD5	1	mRNA	TNIP1	3

mRNA	FAM83D	1	mRNA	ABHD6	1	mRNA	TRAF3IP2	3
mRNA	FAM84B	1	mRNA	ABTB1	1	mRNA	TRAPPC8	3
mRNA	FANCA	1	mRNA	ACACB	1	mRNA	TRERF1	3
mRNA	FANCC	1	mRNA	ACER3	1	mRNA	TRIAP1	3
mRNA	FANCD2	1	mRNA	ACOT7	1	mRNA	TRIM59	3
mRNA	FANCF	1	mRNA	ACSS1	1	mRNA	TRIO	3
mRNA	FANCM	1	mRNA	ACVR1B	1	mRNA	TRIP10	3
mRNA	FASTKD3	1	mRNA	ACVR1C	1	mRNA	TRIP13	3
mRNA	FASTKD5	1	mRNA	ADAM19	1	mRNA	TSHZ3	3
mRNA	FERMT1	1	mRNA	ADAT2	1	mRNA	TSPAN14	3
mRNA	FGD4	1	mRNA	ADCY9	1	mRNA	TSR1	3
mRNA	FGFR2	1	mRNA	ADPRH	1	mRNA	TTC9	3
mRNA	FHIT	1	mRNA	AFF3	1	mRNA	TUBGCP4	3
mRNA	FIGNL1	1	mRNA	AGAP4	1	mRNA	TUBGCP5	3
mRNA	FKBP11	1	mRNA	AGAP6	1	mRNA	TXNDC9	3
mRNA	FKTN	1	mRNA	AGMAT	1	mRNA	TYMS	3
mRNA	FLNB	1	mRNA	AGTRAP	1	mRNA	UBA2	3
mRNA	FLVCR2	1	mRNA	AHI1	1	mRNA	UBE2G2	3
mRNA	FOS	1	mRNA	AIFM2	1	mRNA	UBE2K	3
mRNA	FOSL2	1	mRNA	AIM1	1	mRNA	UBR3	3
mRNA	FOXM1	1	mRNA	AKIRIN1	1	mRNA	ULK1	3
mRNA	FOXRED1	1	mRNA	AKTIP	1	mRNA	URB2	3
mRNA	FPGT	1	mRNA	ALCAM	1	mRNA	VAT1	3
mRNA	FSD1	1	mRNA	ALDH1A1	1	mRNA	VRK1	3
mRNA	FUCA1	1	mRNA	ALDH1A2	1	mRNA	WASF2	3
mRNA	FYN	1	mRNA	ALDH7A1	1	mRNA	WDR5	3
mRNA	FZD3	1	mRNA	AMACR	1	mRNA	WEE1	3
mRNA	GABARAP	1	mRNA	ANKAR	1	mRNA	YEATS4	3
mRNA	GABARAPL2	1	mRNA	ANLN	1	mRNA	YIPF6	3
mRNA	GALK1	1	mRNA	ANXA1	1	mRNA	YTHDF2	3
mRNA	GALNT10	1	mRNA	APOO	1	mRNA	ZBTB10	3
mRNA	GAPT	1	mRNA	ARAP2	1	mRNA	ZBTB4	3
mRNA	GARS	1	mRNA	ARFGAP3	1	mRNA	ZBTB44	3
mRNA	GCA	1	mRNA	ARHGAP12	1	mRNA	ZDHHC2	3
mRNA	GCNT1	1	mRNA	ARHGAP18	1	mRNA	ZEB1	3
mRNA	GFI1	1	mRNA	ARHGAP20	1	mRNA	ZFP36L2	3
mRNA	GINS4	1	mRNA	ARHGEF10	1	mRNA	ZFPM2	3
mRNA	GLA	1	mRNA	ARHGEF2	1	mRNA	ZMAT3	3
mRNA	GLB1L2	1	mRNA	ARID4B	1	mRNA	ZNF230	3
mRNA	GLI2	1	mRNA	ARL5A	1	mRNA	ZNF248	3
mRNA	GLMN	1	mRNA	ARL6IP6	1	mRNA	ZNF326	3
mRNA	GMCL1	1	mRNA	ARMCX2	1	mRNA	ZNF367	3
mRNA	GMDS	1	mRNA	ARSK	1	mRNA	ZNF521	3
mRNA	GMFB	1	mRNA	ASB13	1	mRNA	ZNF532	3
mRNA	GNA12	1	mRNA	ASCC3	1	mRNA	ZNF609	3
mRNA	GNAI1	1	mRNA	ASF1B	1	mRNA	ZNF624	3
mRNA	GPR124	1	mRNA	ASPHD1	1	mRNA	ZNF695	3
mRNA	GPR137B	1	mRNA	ATF3	1	mRNA	ZNF75A	3
mRNA	GPR146	1	mRNA	ATG4D	1	mRNA	ZNFX1	3
mRNA	GPRIN3	1	mRNA	ATG5	1	mRNA	ZNRF1	3
mRNA	GPSM2	1	mRNA	ATP13A3	1	mRNA	NABP1	3
mRNA	GSG2	1	mRNA	ATP7B	1	mRNA	AATF	2
mRNA	GSTCD	1	mRNA	ATP9A	1	mRNA	ABHD5	2

mRNA	GSTK1	1
mRNA	GTF2F2	1
mRNA	GTF2I	1
mRNA	GTF2IRD2	1
mRNA	GTPBP8	1
mRNA	GUCY1A3	1
mRNA	GULP1	1
mRNA	H2AFJ	1
mRNA	H2AFY	1
mRNA	HADH	1
mRNA	HAT1	1
mRNA	HAUS1	1
mRNA	HAUS8	1
mRNA	HEBP1	1
mRNA	HECTD3	1
mRNA	HELLS	1
mRNA	HGF	1
mRNA	HGSNAT	1
mRNA	HHLA3	1
mRNA	HIST2H2BF	1
mRNA	HLA-DPB1	1
mRNA	HMBOX1	1
mRNA	HMMR	1
mRNA	HNRNPAB	1
mRNA	HOPX	1
mRNA	HOXA10	1
mRNA	HOXA4	1
mRNA	HOXA5	1
mRNA	HOXA9	1
mRNA	HOXB3	1
mRNA	HRSP12	1
mRNA	HSPA4L	1
mRNA	HSPA9	1
mRNA	HSPH1	1
mRNA	ICA1L	1
mRNA	ID1	1
mRNA	IGF1R	1
mRNA	IL12A	1
mRNA	IL12RB2	1
mRNA	IL13RA1	1
mRNA	IL1RAP	1
mRNA	IL21R	1
mRNA	IMMP2L	1
mRNA	INA	1
mRNA	INF2	1
mRNA	IPO4	1
mRNA	IPPK	1
mRNA	IQCA1	1
mRNA	IRF8	1
mRNA	ISCA2	1
mRNA	JARID2	1
mRNA	JUNB	1
mRNA	KBTBD8	1
mRNA	B3GNT5	1
mRNA	BARD1	1
mRNA	BCCIP	1
mRNA	BCL2	1
mRNA	BCL2L1	1
mRNA	BCL2L11	1
mRNA	BDH1	1
mRNA	BECN1	1
mRNA	BEND6	1
mRNA	BIN1	1
mRNA	BLOC1S1	1
mRNA	BMP4	1
mRNA	BMP6	1
mRNA	BMP8B	1
mRNA	BMPR1B	1
mRNA	BNIP1	1
mRNA	BRE	1
mRNA	BRI3BP	1
mRNA	BRIP1	1
mRNA	BSDC1	1
mRNA	BTAF1	1
mRNA	BTBD2	1
mRNA	BTG2	1
mRNA	BTG3	1
mRNA	BUB1B	1
mRNA	C10orf10	1
mRNA	C11orf49	1
mRNA	C14orf159	1
mRNA	C16orf45	1
mRNA	C1S	1
mRNA	C1orf112	1
mRNA	C2CD2	1
mRNA	C5	1
mRNA	C5orf15	1
mRNA	C6orf211	1
mRNA	CABLES2	1
mRNA	CAD	1
mRNA	CAMK2G	1
mRNA	CAMSAP2	1
mRNA	CARHSP1	1
mRNA	CARS	1
mRNA	CASZ1	1
mRNA	CAT	1
mRNA	CBX7	1
mRNA	CCDC15	1
mRNA	CCDC28A	1
mRNA	CCDC53	1
mRNA	CCPG1	1
mRNA	CCT6A	1
mRNA	CCT8	1
mRNA	CD24	1
mRNA	CD44	1
mRNA	CD47	1
mRNA	ABHD6	2
mRNA	ABTB2	2
mRNA	ACAT2	2
mRNA	ACOT7	2
mRNA	ACP2	2
mRNA	ACTL6A	2
mRNA	ACVR1B	2
mRNA	ADAM17	2
mRNA	ADCY1	2
mRNA	ADORA2B	2
mRNA	ADPRH	2
mRNA	AGFG2	2
mRNA	AHDC1	2
mRNA	AHI1	2
mRNA	AKAP13	2
mRNA	AKIRIN1	2
mRNA	AKTIP	2
mRNA	ALDH2	2
mRNA	ALG13	2
mRNA	ALPK1	2
mRNA	AMIGO2	2
mRNA	AMPD2	2
mRNA	ANGPT1	2
mRNA	ANKH	2
mRNA	ANKRD12	2
mRNA	ANKRD46	2
mRNA	ANKRD6	2
mRNA	ANLN	2
mRNA	ANXA1	2
mRNA	AP1S3	2
mRNA	AP4E1	2
mRNA	APBA2	2
mRNA	AQP3	2
mRNA	ARAP2	2
mRNA	ARF5	2
mRNA	ARG2	2
mRNA	ARHGAP11A	2
mRNA	ARHGAP18	2
mRNA	ARHGEF10	2
mRNA	ARHGEF2	2
mRNA	ARL2	2
mRNA	ASAH1	2
mRNA	ASB1	2
mRNA	ASB9	2
mRNA	ATP13A3	2
mRNA	ATP8A1	2
mRNA	BAG2	2
mRNA	BAG4	2
mRNA	BAIAP2	2
mRNA	BBS2	2
mRNA	BCL2L1	2
mRNA	BCL2L11	2
mRNA	BIRC5	2

mRNA	KCNK2	1	mRNA	CD59	1	mRNA	BLM	2
mRNA	KCNN4	1	mRNA	CD69	1	mRNA	BMP1	2
mRNA	KCTD14	1	mRNA	CD80	1	mRNA	BORA	2
mRNA	KCTD20	1	mRNA	CD99L2	1	mRNA	BRI3BP	2
mRNA	KDELC2	1	mRNA	CDC20	1	mRNA	BTBD2	2
mRNA	KIAA0040	1	mRNA	CDC23	1	mRNA	BTN3A3	2
mRNA	KIAA0226	1	mRNA	CDC42EP2	1	mRNA	BUB1	2
mRNA	KIAA1147	1	mRNA	CDC7	1	mRNA	C11orf73	2
mRNA	KIAA1462	1	mRNA	CDCA2	1	mRNA	C11orf96	2
mRNA	KIAA1598	1	mRNA	CDCA3	1	mRNA	C12orf4	2
mRNA	KIF18A	1	mRNA	CDH2	1	mRNA	C12orf5	2
mRNA	KIF20A	1	mRNA	CDK1	1	mRNA	C14orf2	2
mRNA	KIF23	1	mRNA	CDK4	1	mRNA	C18orf54	2
mRNA	KIF24	1	mRNA	CDKN3	1	mRNA	C1orf112	2
mRNA	KIF2A	1	mRNA	CDR2	1	mRNA	C1orf74	2
mRNA	KIF2C	1	mRNA	CENPK	1	mRNA	C3orf58	2
mRNA	KIRREL	1	mRNA	CENPM	1	mRNA	C6orf120	2
mRNA	KLF5	1	mRNA	CENPO	1	mRNA	C9orf40	2
mRNA	KLF7	1	mRNA	CEP128	1	mRNA	CAMK1	2
mRNA	KLHDC8B	1	mRNA	CEP135	1	mRNA	CAMK2G	2
mRNA	KLHL2	1	mRNA	CEP55	1	mRNA	CASP2	2
mRNA	KLHL23	1	mRNA	CERK	1	mRNA	CASP8AP2	2
mRNA	KLHL24	1	mRNA	CHAF1A	1	mRNA	CAT	2
mRNA	KLHL3	1	mRNA	CHCHD7	1	mRNA	CBLB	2
mRNA	KLHL36	1	mRNA	CHD7	1	mRNA	CCBL1	2
mRNA	KSR1	1	mRNA	CHMP4A	1	mRNA	CCDC126	2
mRNA	L3MBTL4	1	mRNA	CHRNA5	1	mRNA	CCDC34	2
mRNA	LACTB	1	mRNA	CISH	1	mRNA	CCDC43	2
mRNA	LAMA2	1	mRNA	CKS2	1	mRNA	CCDC53	2
mRNA	LAMA3	1	mRNA	CLGN	1	mRNA	CCDC68	2
mRNA	LAMA5	1	mRNA	CLU	1	mRNA	CCDC71L	2
mRNA	LAMC1	1	mRNA	CNKSR1	1	mRNA	CCNA2	2
mRNA	LAMP3	1	mRNA	CNOT6L	1	mRNA	CCNE1	2
mRNA	LDB1	1	mRNA	COL15A1	1	mRNA	CCNYL1	2
mRNA	LHX6	1	mRNA	COL24A1	1	mRNA	CCRN4L	2
mRNA	LIMA1	1	mRNA	COTL1	1	mRNA	CD24	2
mRNA	LLGL1	1	mRNA	CPEB4	1	mRNA	CD274	2
mRNA	LMNB1	1	mRNA	CPVL	1	mRNA	CD276	2
mRNA	LPAR1	1	mRNA	CREB5	1	mRNA	CD302	2
mRNA	LPCAT4	1	mRNA	CRELD1	1	mRNA	CD37	2
mRNA	LRMP	1	mRNA	CSF1	1	mRNA	CD44	2
mRNA	LRP12	1	mRNA	CSNK1G1	1	mRNA	CD48	2
mRNA	LRRC28	1	mRNA	CSRP2BP	1	mRNA	CDC25A	2
mRNA	LRRC61	1	mRNA	CTNNAL1	1	mRNA	CDC42BPB	2
mRNA	LSM11	1	mRNA	CTSF	1	mRNA	CDC7	2
mRNA	LYAR	1	mRNA	CTSO	1	mRNA	CDCA4	2
mRNA	MACC1	1	mRNA	CUEDC2	1	mRNA	CDCA5	2
mRNA	MAML3	1	mRNA	CXCL12	1	mRNA	CDCA7	2
mRNA	MAMLD1	1	mRNA	CYB5D1	1	mRNA	CDCA8	2
mRNA	MAN1A1	1	mRNA	CYP4V2	1	mRNA	CDIPT	2
mRNA	MANBA	1	mRNA	CYSLTR2	1	mRNA	CDK18	2
mRNA	MAP2K3	1	mRNA	CYTH3	1	mRNA	CDK4	2
mRNA	MAP3K13	1	mRNA	CYTH4	1	mRNA	CDKN1A	2

mRNA	MAP3K5	1	mRNA	DAB2	1	mRNA	CEBPG	2
mRNA	MAP4	1	mRNA	DACT1	1	mRNA	CELSR3	2
mRNA	MAP4K4	1	mRNA	DAG1	1	mRNA	CENPK	2
mRNA	MAP6D1	1	mRNA	DAGLA	1	mRNA	CENPO	2
mRNA	MAPKAPK3	1	mRNA	DAGLB	1	mRNA	CENPW	2
mRNA	MARS	1	mRNA	DAP3	1	mRNA	CEP128	2
mRNA	MASTL	1	mRNA	DBI	1	mRNA	CEP135	2
mRNA	MBOAT1	1	mRNA	DBP	1	mRNA	CEP55	2
mRNA	MCAM	1	mRNA	DCHS1	1	mRNA	CEP97	2
mRNA	MCF2L	1	mRNA	DCK	1	mRNA	CHCHD7	2
mRNA	MCM7	1	mRNA	DCLK2	1	mRNA	CHD5	2
mRNA	MDH1	1	mRNA	DCTN4	1	mRNA	CHMP4C	2
mRNA	MDK	1	mRNA	DCTPP1	1	mRNA	CLN6	2
mRNA	ME1	1	mRNA	DCUN1D5	1	mRNA	CLSPN	2
mRNA	MED11	1	mRNA	DDO	1	mRNA	CNKSR1	2
mRNA	MED12L	1	mRNA	DDX11	1	mRNA	COL7A1	2
mRNA	MED15	1	mRNA	DDX21	1	mRNA	CPA3	2
mRNA	MED16	1	mRNA	DDX60	1	mRNA	CPSF4	2
mRNA	MELK	1	mRNA	DECR2	1	mRNA	CROT	2
mRNA	METAP1	1	mRNA	DENND3	1	mRNA	CRYBG3	2
mRNA	METTL2A	1	mRNA	DEPDC1B	1	mRNA	CSRNP1	2
mRNA	MFN2	1	mRNA	DGKD	1	mRNA	CSTF3	2
mRNA	MFSD2A	1	mRNA	DHCR24	1	mRNA	CTSS	2
mRNA	MGLL	1	mRNA	DHFR	1	mRNA	CXCL12	2
mRNA	MICAL2	1	mRNA	DHX38	1	mRNA	CXCL6	2
mRNA	MKI67	1	mRNA	DIAPH3	1	mRNA	CXorf23	2
mRNA	MKNK1	1	mRNA	DIXDC1	1	mRNA	CYP2S1	2
mRNA	MLEC	1	mRNA	DKC1	1	mRNA	CYP2U1	2
mRNA	MLF1	1	mRNA	DLEU1	1	mRNA	CYTH3	2
mRNA	MLKL	1	mRNA	DLGAP5	1	mRNA	DACH1	2
mRNA	MORN2	1	mRNA	DMRTA2	1	mRNA	DAPP1	2
mRNA	MPDZ	1	mRNA	DNA2	1	mRNA	DBI	2
mRNA	MPP2	1	mRNA	DNAJA3	1	mRNA	DCAF17	2
mRNA	MRPL13	1	mRNA	DNAJA4	1	mRNA	DCLK2	2
mRNA	MRPL16	1	mRNA	DNAJB11	1	mRNA	DCLRE1A	2
mRNA	MRPL39	1	mRNA	DNAJC18	1	mRNA	DDX21	2
mRNA	MRPL40	1	mRNA	DNAJC3	1	mRNA	DDX56	2
mRNA	MRPL46	1	mRNA	DNAJC7	1	mRNA	DECR2	2
mRNA	MRRF	1	mRNA	DNM1	1	mRNA	DENND3	2
mRNA	MRTO4	1	mRNA	DOCK9	1	mRNA	DEPDC1B	2
mRNA	MSRB3	1	mRNA	DPF1	1	mRNA	DGKH	2
mRNA	MTL5	1	mRNA	DPM2	1	mRNA	DHODH	2
mRNA	MXD1	1	mRNA	DPPA4	1	mRNA	DHX33	2
mRNA	MYBL2	1	mRNA	DRG1	1	mRNA	DIAPH3	2
mRNA	MYCBP2	1	mRNA	DSC2	1	mRNA	DIS3	2
mRNA	MYCN	1	mRNA	DSCC1	1	mRNA	DLAT	2
mRNA	MYD88	1	mRNA	DTL	1	mRNA	DLEU1	2
mRNA	MYO18A	1	mRNA	DTWD1	1	mRNA	DLGAP5	2
mRNA	MYO19	1	mRNA	DTYMK	1	mRNA	DNA2	2
mRNA	MYO5C	1	mRNA	DUSP1	1	mRNA	DNAJA4	2
mRNA	MYOZ3	1	mRNA	DUSP19	1	mRNA	DNAJC6	2
mRNA	NAA16	1	mRNA	DUSP22	1	mRNA	DPP9	2
mRNA	NAA38	1	mRNA	DUSP5	1	mRNA	DPPA4	2

mRNA	NAP1L2	1
mRNA	NAPB	1
mRNA	NAPEPLD	1
mRNA	NASP	1
mRNA	NBR1	1
mRNA	NCAPD2	1
mRNA	NCKAP1	1
mRNA	NDUFB10	1
mRNA	NDUFB6	1
mRNA	NFIL3	1
mRNA	NFKBIA	1
mRNA	NFYB	1
mRNA	NIPSNAP1	1
mRNA	NLGN1	1
mRNA	NLN	1
mRNA	NOP2	1
mRNA	NPEPL1	1
mRNA	NR4A1	1
mRNA	NR4A2	1
mRNA	NRIP1	1
mRNA	NRM	1
mRNA	NRP1	1
mRNA	NSMCE2	1
mRNA	NSUN7	1
mRNA	NT5DC3	1
mRNA	NUP35	1
mRNA	NUPL1	1
mRNA	OCLN	1
mRNA	OPRL1	1
mRNA	OSBPL10	1
mRNA	OSBPL1A	1
mRNA	OSGEPL1	1
mRNA	OTUD5	1
mRNA	OTUD6B	1
mRNA	OXNAD1	1
mRNA	PA2G4	1
mRNA	PAFAH1B3	1
mRNA	PAG1	1
mRNA	PAIP2B	1
mRNA	PALLD	1
mRNA	PAPD5	1
mRNA	PAPSS2	1
mRNA	PARD3B	1
mRNA	PARP3	1
mRNA	PAXIP1	1
mRNA	PBK	1
mRNA	PCSK5	1
mRNA	PCYOX1L	1
mRNA	PDAP1	1
mRNA	PDCD1LG2	1
mRNA	PDCD4	1
mRNA	PDE4B	1
mRNA	PDE4DIP	1
mRNA	DUSP8	1
mRNA	DUT	1
mRNA	DYNLT3	1
mRNA	DZIP1	1
mRNA	E2F1	1
mRNA	E2F3	1
mRNA	E2F8	1
mRNA	EBF1	1
mRNA	ECHDC1	1
mRNA	EDARADD	1
mRNA	EDEM1	1
mRNA	EFNA4	1
mRNA	EGR2	1
mRNA	EHD2	1
mRNA	EHD4	1
mRNA	EHMT2	1
mRNA	EIF4E3	1
mRNA	EIF5	1
mRNA	ELL	1
mRNA	EMB	1
mRNA	EMG1	1
mRNA	EMP1	1
mRNA	ENAH	1
mRNA	ENOX1	1
mRNA	EPB41L2	1
mRNA	EPB41L3	1
mRNA	EPB41L5	1
mRNA	EPCAM	1
mRNA	EPN2	1
mRNA	EPS8	1
mRNA	EPSTI1	1
mRNA	ERP29	1
mRNA	ESYT2	1
mRNA	ETFB	1
mRNA	ETS1	1
mRNA	ETV3	1
mRNA	ETV4	1
mRNA	EWSR1	1
mRNA	EXO1	1
mRNA	EYA3	1
mRNA	FADD	1
mRNA	FAM102B	1
mRNA	FAM107B	1
mRNA	FAM117A	1
mRNA	FAM120C	1
mRNA	FAM124A	1
mRNA	FAM126A	1
mRNA	FAM127C	1
mRNA	FAM129A	1
mRNA	FAM161A	1
mRNA	FAM171B	1
mRNA	FAM173B	1
mRNA	FAM175A	1
mRNA	DPYSL3	2
mRNA	DSCC1	2
mRNA	DTNA	2
mRNA	DUSP18	2
mRNA	DUT	2
mRNA	E2F8	2
mRNA	EBF4	2
mRNA	ECE1	2
mRNA	EGLN1	2
mRNA	EHD1	2
mRNA	EIF2S2	2
mRNA	EIF4E3	2
mRNA	EIF5	2
mRNA	EIF5B	2
mRNA	ELP4	2
mRNA	EMG1	2
mRNA	ENOX1	2
mRNA	ENTPD1	2
mRNA	EPCAM	2
mRNA	EPHB4	2
mRNA	EPT1	2
mRNA	ERP29	2
mRNA	ESYT2	2
mRNA	ETAA1	2
mRNA	EWSR1	2
mRNA	EXOSC8	2
mRNA	EXPH5	2
mRNA	EZH1	2
mRNA	FAM111B	2
mRNA	FAM114A1	2
mRNA	FAM136A	2
mRNA	FAM184A	2
mRNA	FAM188A	2
mRNA	FAM189B	2
mRNA	FAM195A	2
mRNA	FAM53B	2
mRNA	FAM78A	2
mRNA	FAM84B	2
mRNA	FANCD2	2
mRNA	FANCM	2
mRNA	FASTKD3	2
mRNA	FBXO4	2
mRNA	FGD4	2
mRNA	FGF2	2
mRNA	FGF9	2
mRNA	FGFR2	2
mRNA	FICD	2
mRNA	FKBP11	2
mRNA	FKBP9	2
mRNA	FLT1	2
mRNA	FLVCR2	2
mRNA	FMNL2	2
mRNA	FMNL3	2

mRNA	PDE5A	1	mRNA	FAM177A1	1	mRNA	FN1	2
mRNA	PDE8A	1	mRNA	FAM184A	1	mRNA	FOXM1	2
mRNA	PDGFD	1	mRNA	FAM198B	1	mRNA	FOXN2	2
mRNA	PDIK1L	1	mRNA	FAM214B	1	mRNA	FOXO3	2
mRNA	PDK2	1	mRNA	FAM217B	1	mRNA	FOXRED1	2
mRNA	PDLIM1	1	mRNA	FAM43A	1	mRNA	FRAT1	2
mRNA	PDRG1	1	mRNA	FAM63B	1	mRNA	FRY	2
mRNA	PEAK1	1	mRNA	FAM92A1	1	mRNA	FUCA2	2
mRNA	PER3	1	mRNA	FASTKD3	1	mRNA	FYN	2
mRNA	PEX6	1	mRNA	FAT4	1	mRNA	GABARAP	2
mRNA	PGP	1	mRNA	FBXL6	1	mRNA	GABARAPL2	2
mRNA	PHF5A	1	mRNA	FBXW7	1	mRNA	GAK	2
mRNA	PIBF1	1	mRNA	FCHO2	1	mRNA	GALK1	2
mRNA	PIGM	1	mRNA	FGF2	1	mRNA	GATA2	2
mRNA	PIK3CD	1	mRNA	FGF9	1	mRNA	GATM	2
mRNA	PIK3CG	1	mRNA	FGL2	1	mRNA	GBF1	2
mRNA	PIK3IP1	1	mRNA	FHIT	1	mRNA	GCH1	2
mRNA	PIP5K1B	1	mRNA	FHL1	1	mRNA	GCNT1	2
mRNA	PITPNC1	1	mRNA	FHOD1	1	mRNA	GDNF	2
mRNA	PKMYT1	1	mRNA	FICD	1	mRNA	GLA	2
mRNA	PLAG1	1	mRNA	FIGNL1	1	mRNA	GLG1	2
mRNA	PLD2	1	mRNA	FLNA	1	mRNA	GLIS3	2
mRNA	PLEKHA1	1	mRNA	FN1	1	mRNA	GLTP	2
mRNA	PLEKHA2	1	mRNA	FNBP1	1	mRNA	GLTSCR1	2
mRNA	PLEKHA7	1	mRNA	FOSB	1	mRNA	GMCL1	2
mRNA	PLEKHA8	1	mRNA	FOXN2	1	mRNA	GMNN	2
mRNA	PLEKHM1	1	mRNA	FOXO3	1	mRNA	GNA12	2
mRNA	PLK2	1	mRNA	FPGT	1	mRNA	GPD1L	2
mRNA	PLK3	1	mRNA	FRY	1	mRNA	GPR107	2
mRNA	PLK4	1	mRNA	FUBP3	1	mRNA	GPR183	2
mRNA	PLS1	1	mRNA	FXYD5	1	mRNA	GPR56	2
mRNA	PLSCR4	1	mRNA	FZD3	1	mRNA	GPR63	2
mRNA	PLXNC1	1	mRNA	GABARAP	1	mRNA	GPT2	2
mRNA	PMEPA1	1	mRNA	GADD45B	1	mRNA	GRN	2
mRNA	PMM2	1	mRNA	GALNT1	1	mRNA	GRPEL1	2
mRNA	PNP	1	mRNA	GALNT10	1	mRNA	GRPEL2	2
mRNA	PNPLA4	1	mRNA	GART	1	mRNA	GSG2	2
mRNA	POC1A	1	mRNA	GAS2L3	1	mRNA	GTF2I	2
mRNA	POLA1	1	mRNA	GAS7	1	mRNA	GTPBP4	2
mRNA	POLD3	1	mRNA	GATA2	1	mRNA	GTSE1	2
mRNA	PPA1	1	mRNA	GATM	1	mRNA	GULP1	2
mRNA	PPAP2A	1	mRNA	GBE1	1	mRNA	HADH	2
mRNA	PPAPDC2	1	mRNA	GBP1	1	mRNA	HAUS8	2
mRNA	PPARA	1	mRNA	GFI1	1	mRNA	HCCS	2
mRNA	PPM1G	1	mRNA	GFPT2	1	mRNA	HEBP1	2
mRNA	PPOX	1	mRNA	GGH	1	mRNA	HECW2	2
mRNA	PRAME	1	mRNA	GINS4	1	mRNA	HEG1	2
mRNA	PREX1	1	mRNA	GLG1	1	mRNA	HELLS	2
mRNA	PRIM2	1	mRNA	GLI2	1	mRNA	HEY1	2
mRNA	PRKCA	1	mRNA	GLI3	1	mRNA	HGF	2
mRNA	PRKCB	1	mRNA	GLMN	1	mRNA	HLA-G	2
mRNA	PRKCH	1	mRNA	GLRX3	1	mRNA	HLTF	2
mRNA	PRR11	1	mRNA	GLTSCR1	1	mRNA	HMGA1	2

mRNA	PRUNE	1	mRNA	GM2A	1	mRNA	HNRNPA3	2
mRNA	PSMB5	1	mRNA	GMPR	1	mRNA	HOXA3	2
mRNA	PSMC3IP	1	mRNA	GMPS	1	mRNA	HOXA5	2
mRNA	PSMD11	1	mRNA	GNA12	1	mRNA	HOXA7	2
mRNA	PSMD12	1	mRNA	GOLPH3	1	mRNA	HOXB3	2
mRNA	PSME4	1	mRNA	GOLT1B	1	mRNA	HOXB4	2
mRNA	PSTPIP2	1	mRNA	GPD1L	1	mRNA	HPS3	2
mRNA	PTDSS1	1	mRNA	GPR137B	1	mRNA	HSD17B11	2
mRNA	PTK2B	1	mRNA	GPR180	1	mRNA	HSP90B1	2
mRNA	PTPLAD2	1	mRNA	GPR56	1	mRNA	HSPA14	2
mRNA	PTPLB	1	mRNA	GPR63	1	mRNA	HSPA4L	2
mRNA	PTPN22	1	mRNA	GPRIN3	1	mRNA	HSPA9	2
mRNA	PTPN4	1	mRNA	GRB10	1	mRNA	HSPB11	2
mRNA	PTPN9	1	mRNA	GRPEL1	1	mRNA	IARS	2
mRNA	PTPRC	1	mRNA	GRPEL2	1	mRNA	ICA1L	2
mRNA	PTPRF	1	mRNA	GSG2	1	mRNA	ICAM1	2
mRNA	PUS7	1	mRNA	GSPT2	1	mRNA	IFRD2	2
mRNA	PXK	1	mRNA	GSR	1	mRNA	IL11	2
mRNA	PYCRL	1	mRNA	GSTCD	1	mRNA	IL21R	2
mRNA	PYGL	1	mRNA	GTF2I	1	mRNA	IL7	2
mRNA	RAB11FIP4	1	mRNA	GTF2IRD2	1	mRNA	ILDR1	2
mRNA	RAB13	1	mRNA	GTPBP8	1	mRNA	INCENP	2
mRNA	RAB2B	1	mRNA	GUCY1B3	1	mRNA	INSR	2
mRNA	RAB31	1	mRNA	GUF1	1	mRNA	INTS6	2
mRNA	RAB3D	1	mRNA	H2AFY	1	mRNA	IPPK	2
mRNA	RAB5B	1	mRNA	H6PD	1	mRNA	IQCA1	2
mRNA	RABEPK	1	mRNA	HAS2	1	mRNA	IRF8	2
mRNA	RACGAP1	1	mRNA	HAT1	1	mRNA	ISCA2	2
mRNA	RAD18	1	mRNA	HAUS2	1	mRNA	ITGA4	2
mRNA	RAD51AP1	1	mRNA	HAUS4	1	mRNA	ITPR3	2
mRNA	RAD51C	1	mRNA	HAUS6	1	mRNA	KANK1	2
mRNA	RAI14	1	mRNA	HAUS8	1	mRNA	KATNAL1	2
mRNA	RAN	1	mRNA	HBS1L	1	mRNA	KCNA3	2
mRNA	RAPGEFL1	1	mRNA	HEBP1	1	mRNA	KCNN4	2
mRNA	RASGEF1A	1	mRNA	HEG1	1	mRNA	KCTD10	2
mRNA	RASSF6	1	mRNA	HELLS	1	mRNA	KCTD14	2
mRNA	RBL1	1	mRNA	HERPUD1	1	mRNA	KCTD15	2
mRNA	RBM10	1	mRNA	HEY1	1	mRNA	KIAA1109	2
mRNA	RBM38	1	mRNA	HHLA3	1	mRNA	KIAA1147	2
mRNA	RCAN3	1	mRNA	HIST1H2AB	1	mRNA	KIAA1244	2
mRNA	RCC1	1	mRNA	HIST1H3B	1	mRNA	KIAA1598	2
mRNA	RCSD1	1	mRNA	HIVEP2	1	mRNA	KIF11	2
mRNA	REPS2	1	mRNA	HLA-C	1	mRNA	KIF13A	2
mRNA	REXO1	1	mRNA	HLA-DQB1	1	mRNA	KIF13B	2
mRNA	RFC2	1	mRNA	HLTF	1	mRNA	KIF15	2
mRNA	RFC4	1	mRNA	HMBOX1	1	mRNA	KIF20A	2
mRNA	RFC5	1	mRNA	HMGXB3	1	mRNA	KIF24	2
mRNA	RFK	1	mRNA	HNRNPA3	1	mRNA	KIF3A	2
mRNA	RFX2	1	mRNA	HOXA10	1	mRNA	KLF7	2
mRNA	RFXAP	1	mRNA	HOXA4	1	mRNA	KLHL23	2
mRNA	RGS16	1	mRNA	HOXB2	1	mRNA	KSR1	2
mRNA	RIN2	1	mRNA	HS3ST3B1	1	mRNA	LACTB	2
mRNA	RNF24	1	mRNA	HS6ST2	1	mRNA	LCLAT1	2

mRNA	RNF44	1
mRNA	ROBO1	1
mRNA	RP9	1
mRNA	RPAP3	1
mRNA	RPL13	1
mRNA	RPL23	1
mRNA	RPL32	1
mRNA	RPL34	1
mRNA	RPS14	1
mRNA	RPS6KA4	1
mRNA	RPS6KA5	1
mRNA	RPS6KA6	1
mRNA	RPS6KC1	1
mRNA	RPSA	1
mRNA	RUNDC3B	1
mRNA	RWDD2B	1
mRNA	S1PR1	1
mRNA	SALL2	1
mRNA	SAMD12	1
mRNA	SAMD9L	1
mRNA	SAP30L	1
mRNA	SCAMP5	1
mRNA	SCFD1	1
mRNA	SDC1	1
mRNA	SEC24A	1
mRNA	SEPP1	1
mRNA	3-Sep	1
mRNA	6-Sep	1
mRNA	SESN2	1
mRNA	SFMBT2	1
mRNA	SFXN3	1
mRNA	SGCB	1
mRNA	SGK3	1
mRNA	SGMS2	1
mRNA	SGPL1	1
mRNA	SH2B3	1
mRNA	SHCBP1	1
mRNA	SHMT1	1
mRNA	SKA2	1
mRNA	SKIL	1
mRNA	SLAIN1	1
mRNA	SLAMF7	1
mRNA	SLC11A2	1
mRNA	SLC12A9	1
mRNA	SLC16A1	1
mRNA	SLC24A1	1
mRNA	SLC25A15	1
mRNA	SLC25A25	1
mRNA	SLC30A9	1
mRNA	SLC31A1	1
mRNA	SLC38A1	1
mRNA	SLC41A2	1
mRNA	SLC45A4	1
mRNA	HSPA13	1
mRNA	HSPA14	1
mRNA	HSPD1	1
mRNA	HSPH1	1
mRNA	IARS	1
mRNA	ICA1L	1
mRNA	ICAM5	1
mRNA	IFI30	1
mRNA	IFI44L	1
mRNA	IFIT2	1
mRNA	IFIT5	1
mRNA	IFITM1	1
mRNA	IFNAR2	1
mRNA	IFRD2	1
mRNA	IGF1	1
mRNA	IGFBP5	1
mRNA	IL1RAP	1
mRNA	IL21R	1
mRNA	IL6	1
mRNA	IMPA2	1
mRNA	INF2	1
mRNA	ING2	1
mRNA	INSM1	1
mRNA	IPO4	1
mRNA	IPPK	1
mRNA	IQCA1	1
mRNA	IQGAP1	1
mRNA	ISCA2	1
mRNA	ITGA2	1
mRNA	ITGA6	1
mRNA	ITGB3	1
mRNA	ITGB8	1
mRNA	ITPK1	1
mRNA	ITPR3	1
mRNA	IVNS1ABP	1
mRNA	JMJD6	1
mRNA	KANK1	1
mRNA	KCNA3	1
mRNA	KCNK5	1
mRNA	KCTD14	1
mRNA	KCTD15	1
mRNA	KCTD20	1
mRNA	KCTD9	1
mRNA	KDELC1	1
mRNA	KDELC2	1
mRNA	KDM6B	1
mRNA	KIAA0020	1
mRNA	KIAA0226	1
mRNA	KIAA0895	1
mRNA	KIAA0922	1
mRNA	KIAA1109	1
mRNA	KIAA1244	1
mRNA	KIAA1524	1
mRNA	LDLRAD3	2
mRNA	LHX6	2
mRNA	LIPT2	2
mRNA	LIX1L	2
mRNA	LMLN	2
mRNA	LPAR1	2
mRNA	LPCAT4	2
mRNA	LRP1	2
mRNA	LRP12	2
mRNA	LRRC61	2
mRNA	LSM5	2
mRNA	LTV1	2
mRNA	LY75	2
mRNA	LYAR	2
mRNA	MAK16	2
mRNA	MANF	2
mRNA	MAP2K3	2
mRNA	MAP3K14	2
mRNA	MAP4	2
mRNA	MAP7D2	2
mRNA	MAPKAPK3	2
mRNA	MAPKBP1	2
mRNA	MCM2	2
mRNA	MCM5	2
mRNA	MCM6	2
mRNA	MDFIC	2
mRNA	MED11	2
mRNA	MED16	2
mRNA	MESDC1	2
mRNA	METTL9	2
mRNA	MFN2	2
mRNA	MIF4GD	2
mRNA	MLEC	2
mRNA	MLF1	2
mRNA	MMAA	2
mRNA	MN1	2
mRNA	MNAT1	2
mRNA	MPPED2	2
mRNA	MRC2	2
mRNA	MRPL30	2
mRNA	MRPL35	2
mRNA	MRPL40	2
mRNA	MRPS23	2
mRNA	MRS2	2
mRNA	MT1E	2
mRNA	MTBP	2
mRNA	MTMR10	2
mRNA	MTMR14	2
mRNA	MTSS1L	2
mRNA	MTUS1	2
mRNA	MTX2	2
mRNA	MYO1E	2
mRNA	MYO5C	2

mRNA	SLC46A3	1	mRNA	KIAA1598	1	mRNA	NAP1L5	2
mRNA	SLC48A1	1	mRNA	KIF18B	1	mRNA	NAPEPLD	2
mRNA	SLC4A4	1	mRNA	KIF20A	1	mRNA	NBEAL1	2
mRNA	SLC6A9	1	mRNA	KIF21A	1	mRNA	NBR1	2
mRNA	SLC9A8	1	mRNA	KIF22	1	mRNA	NCAPG	2
mRNA	SMAD1	1	mRNA	KIF3A	1	mRNA	NDUFAB1	2
mRNA	SMAD7	1	mRNA	KIF5C	1	mRNA	NEIL3	2
mRNA	SMARCA2	1	mRNA	KIFAP3	1	mRNA	NEK9	2
mRNA	SMG5	1	mRNA	KIRREL	1	mRNA	NET1	2
mRNA	SMO	1	mRNA	KLC2	1	mRNA	NIF3L1	2
mRNA	SNAI1	1	mRNA	KLF6	1	mRNA	NIP7	2
mRNA	SNRNP40	1	mRNA	KLHDC8B	1	mRNA	NIPAL4	2
mRNA	SNRPF	1	mRNA	KLHL24	1	mRNA	NIPSNAP1	2
mRNA	SNTB1	1	mRNA	KPNA2	1	mRNA	NME4	2
mRNA	SNX7	1	mRNA	KSR1	1	mRNA	NMT2	2
mRNA	SNX9	1	mRNA	LACTB	1	mRNA	NOL12	2
mRNA	SOBP	1	mRNA	LAMA2	1	mRNA	NOLC1	2
mRNA	SPAG7	1	mRNA	LAMA5	1	mRNA	NQO1	2
mRNA	SPEF2	1	mRNA	LAMP3	1	mRNA	NR2C2AP	2
mRNA	SPG21	1	mRNA	LDB1	1	mRNA	NR4A1	2
mRNA	SPTBN1	1	mRNA	LETMD1	1	mRNA	NRIP3	2
mRNA	SRRT	1	mRNA	LFNG	1	mRNA	NT5DC3	2
mRNA	SRSF1	1	mRNA	LGALSL	1	mRNA	NUAK2	2
mRNA	SSTR2	1	mRNA	LGMN	1	mRNA	NUCB2	2
mRNA	SSU72	1	mRNA	LHX6	1	mRNA	NUDCD3	2
mRNA	ST3GAL6	1	mRNA	LIMK2	1	mRNA	NUDT15	2
mRNA	STEAP2	1	mRNA	LIPT1	1	mRNA	NUMB	2
mRNA	STEAP3	1	mRNA	LIX1L	1	mRNA	NUP62CL	2
mRNA	STUB1	1	mRNA	LLGL1	1	mRNA	NUSAP1	2
mRNA	STX3	1	mRNA	LMO4	1	mRNA	OAS3	2
mRNA	STX7	1	mRNA	LONRF2	1	mRNA	OCLN	2
mRNA	SUB1	1	mRNA	LOX	1	mRNA	OIP5	2
mRNA	SULF2	1	mRNA	LOXL1	1	mRNA	OSBPL1A	2
mRNA	SUSD3	1	mRNA	LPAR6	1	mRNA	OSBPL6	2
mRNA	SUV39H2	1	mRNA	LPCAT4	1	mRNA	OSGEP	2
mRNA	SVIL	1	mRNA	LRMP	1	mRNA	OXA1L	2
mRNA	SYT1	1	mRNA	LRP12	1	mRNA	OXNAD1	2
mRNA	TACC1	1	mRNA	LRP2BP	1	mRNA	P4HA1	2
mRNA	TAF5	1	mRNA	LRRC61	1	mRNA	P4HA2	2
mRNA	TAGAP	1	mRNA	LTBP3	1	mRNA	PAICS	2
mRNA	TARBP1	1	mRNA	LTV1	1	mRNA	PARP1	2
mRNA	TARBP2	1	mRNA	LURAP1L	1	mRNA	PARP3	2
mRNA	TBC1D14	1	mRNA	LY75	1	mRNA	PCDH9	2
mRNA	TBC1D17	1	mRNA	LYST	1	mRNA	PCMTD2	2
mRNA	TBC1D2	1	mRNA	MAF	1	mRNA	PCYOX1L	2
mRNA	TBCEL	1	mRNA	MAGT1	1	mRNA	PDCD1LG2	2
mRNA	TBX19	1	mRNA	MAML3	1	mRNA	PDCD5	2
mRNA	TCEA2	1	mRNA	MAN1C1	1	mRNA	PDE7B	2
mRNA	TCEAL1	1	mRNA	MANBA	1	mRNA	PDGFA	2
mRNA	TCERG1	1	mRNA	MANF	1	mRNA	PDRG1	2
mRNA	TCP11L2	1	mRNA	MAP3K13	1	mRNA	PDXK	2
mRNA	TCTEX1D2	1	mRNA	MAP4K1	1	mRNA	PEBP1	2
mRNA	TDG	1	mRNA	MAPKAP1	1	mRNA	PGAP2	2

mRNA	TET1	1	mRNA	MARVELD1	1	mRNA	PGP	2
mRNA	TFDP1	1	mRNA	MASTL	1	mRNA	PHACTR1	2
mRNA	THEM4	1	mRNA	MBNL3	1	mRNA	PHF1	2
mRNA	THOP1	1	mRNA	MBOAT2	1	mRNA	PHF19	2
mRNA	THRA	1	mRNA	MCM2	1	mRNA	PHKA1	2
mRNA	TIAM1	1	mRNA	MCM6	1	mRNA	PHYH	2
mRNA	TIMM10	1	mRNA	MCM7	1	mRNA	PIBF1	2
mRNA	TIMM50	1	mRNA	MDH1	1	mRNA	PIK3CA	2
mRNA	TIMM8B	1	mRNA	ME1	1	mRNA	PIP5K1B	2
mRNA	TIMP3	1	mRNA	MED10	1	mRNA	PKP2	2
mRNA	TIPIN	1	mRNA	MEF2A	1	mRNA	PLA2G4A	2
mRNA	TJP2	1	mRNA	MELK	1	mRNA	PLEKHG3	2
mRNA	TM4SF1	1	mRNA	MEST	1	mRNA	PLK1	2
mRNA	TMEM106B	1	mRNA	METAP1	1	mRNA	PLS1	2
mRNA	TMEM134	1	mRNA	MFN2	1	mRNA	PMM2	2
mRNA	TMEM161B	1	mRNA	MGLL	1	mRNA	PMP22	2
mRNA	TMEM187	1	mRNA	MGST1	1	mRNA	PNP	2
mRNA	TMEM194A	1	mRNA	MKNK1	1	mRNA	POLD3	2
mRNA	TMEM201	1	mRNA	MMAA	1	mRNA	PPA1	2
mRNA	TMEM242	1	mRNA	MNAT1	1	mRNA	PPAP2A	2
mRNA	TMEM45A	1	mRNA	MOSPD3	1	mRNA	PPAPDC2	2
mRNA	TMEM50B	1	mRNA	MPDZ	1	mRNA	PPARA	2
mRNA	TMEM80	1	mRNA	MPP7	1	mRNA	PPM1H	2
mRNA	TMPO	1	mRNA	MRPL2	1	mRNA	PPP1R14A	2
mRNA	TMTC4	1	mRNA	MRPL3	1	mRNA	PPP2R3A	2
mRNA	TNFAIP2	1	mRNA	MRPL30	1	mRNA	PPP2R5C	2
mRNA	TNFRSF10D	1	mRNA	MRPL40	1	mRNA	PPRC1	2
mRNA	TNFSF9	1	mRNA	MRPS35	1	mRNA	PRAME	2
mRNA	TOM1L2	1	mRNA	MSRB2	1	mRNA	PRDM5	2
mRNA	TOX	1	mRNA	MTMR10	1	mRNA	PRDX3	2
mRNA	TP53	1	mRNA	MTMR14	1	mRNA	PREPL	2
mRNA	TP53BP2	1	mRNA	MTSS1L	1	mRNA	PREX1	2
mRNA	TP53INP1	1	mRNA	MTX2	1	mRNA	PRIM1	2
mRNA	TRIM36	1	mRNA	MYBL1	1	mRNA	PRKAR2B	2
mRNA	TRIT1	1	mRNA	MYCBP2	1	mRNA	PRKCH	2
mRNA	TRMT6	1	mRNA	MYD88	1	mRNA	PROS1	2
mRNA	TSEN15	1	mRNA	MYO18A	1	mRNA	PRR16	2
mRNA	TSKU	1	mRNA	MYO19	1	mRNA	PSMB1	2
mRNA	TSPYL1	1	mRNA	MYO1D	1	mRNA	PSMB2	2
mRNA	TTC30B	1	mRNA	MYO1E	1	mRNA	PSMD1	2
mRNA	TTLL7	1	mRNA	N6AMT2	1	mRNA	PSMD2	2
mRNA	TTYH3	1	mRNA	NAGPA	1	mRNA	PSRC1	2
mRNA	TUBGCP5	1	mRNA	NAP1L2	1	mRNA	PTGS2	2
mRNA	TUFT1	1	mRNA	NAP1L5	1	mRNA	PTPLB	2
mRNA	TXNL4A	1	mRNA	NAPB	1	mRNA	PTPN1	2
mRNA	TYMS	1	mRNA	NCF2	1	mRNA	PTPN4	2
mRNA	TYSND1	1	mRNA	NDUFA5	1	mRNA	PTPN9	2
mRNA	UBE2C	1	mRNA	NDUFB6	1	mRNA	PTPRD	2
mRNA	UBR1	1	mRNA	NECAB3	1	mRNA	PTTG1	2
mRNA	UBXN2B	1	mRNA	NEK6	1	mRNA	PYROXD1	2
mRNA	UFD1L	1	mRNA	NETO2	1	mRNA	QDPR	2
mRNA	UGCG	1	mRNA	NFAT5	1	mRNA	RAB2B	2
mRNA	UHRF1	1	mRNA	NFKBIA	1	mRNA	RAB30	2

mRNA	UHRF1BP1L	1
mRNA	UQCR10	1
mRNA	URB2	1
mRNA	USP51	1
mRNA	VAMP4	1
mRNA	VAMP8	1
mRNA	VANGL1	1
mRNA	VAV3	1
mRNA	VRK1	1
mRNA	WBP4	1
mRNA	WDR35	1
mRNA	WDR54	1
mRNA	WDR81	1
mRNA	WIBG	1
mRNA	WWTR1	1
mRNA	XKR8	1
mRNA	XPO4	1
mRNA	XPOT	1
mRNA	XRRA1	1
mRNA	YPEL2	1
mRNA	YPEL5	1
mRNA	YTHDC2	1
mRNA	ZBTB1	1
mRNA	ZC3H6	1
mRNA	ZC3HAV1L	1
mRNA	ZDBF2	1
mRNA	ZDHHC11	1
mRNA	ZDHHC2	1
mRNA	ZFAND2A	1
mRNA	ZFP14	1
mRNA	ZFP36	1
mRNA	ZFP90	1
mRNA	ZKSCAN1	1
mRNA	ZMYM3	1
mRNA	ZNF138	1
mRNA	ZNF155	1
mRNA	ZNF2	1
mRNA	ZNF22	1
mRNA	ZNF230	1
mRNA	ZNF248	1
mRNA	ZNF32	1
mRNA	ZNF367	1
mRNA	ZNF485	1
mRNA	ZNF492	1
mRNA	ZNF493	1
mRNA	ZNF516	1
mRNA	ZNF521	1
mRNA	ZNF532	1
mRNA	ZNF583	1
mRNA	ZNF615	1
mRNA	ZNF681	1
mRNA	ZNF695	1
mRNA	ZNF75A	1
mRNA	NFYB	1
mRNA	NHEJ1	1
mRNA	NIP7	1
mRNA	NLE1	1
mRNA	NLN	1
mRNA	NOG	1
mRNA	NOLC1	1
mRNA	NR4A2	1
mRNA	NRCAM	1
mRNA	NRP1	1
mRNA	NRP2	1
mRNA	NSMCE2	1
mRNA	NT5DC2	1
mRNA	NUAK2	1
mRNA	NUDCD3	1
mRNA	NUDT6	1
mRNA	NUMB	1
mRNA	NUP205	1
mRNA	NUP35	1
mRNA	NUP88	1
mRNA	NUPL1	1
mRNA	NVL	1
mRNA	OAS1	1
mRNA	OAS3	1
mRNA	OIP5	1
mRNA	OMA1	1
mRNA	ORAI2	1
mRNA	PAFAH1B3	1
mRNA	PAICS	1
mRNA	PAIP2B	1
mRNA	PALM2-AKAP2	1
mRNA	PAPSS2	1
mRNA	PAQR4	1
mRNA	PARP12	1
mRNA	PARP16	1
mRNA	PARP3	1
mRNA	PARP9	1
mRNA	PBLD	1
mRNA	PC	1
mRNA	PCDH9	1
mRNA	PCSK5	1
mRNA	PDCD5	1
mRNA	PDGFRB	1
mRNA	PDK4	1
mRNA	PDLIM1	1
mRNA	PDRG1	1
mRNA	PEAK1	1
mRNA	PECR	1
mRNA	PERP	1
mRNA	PEX6	1
mRNA	PFKFB3	1
mRNA	PGM3	1
mRNA	PGPEP1	1
mRNA	RABEPK	2
mRNA	RAD51	2
mRNA	RAD51C	2
mRNA	RALGPS2	2
mRNA	RAN	2
mRNA	RAPGEF1	2
mRNA	RAPGEFL1	2
mRNA	RASGEF1A	2
mRNA	RASGEF1B	2
mRNA	RAVER2	2
mRNA	RBL1	2
mRNA	RBM10	2
mRNA	RBM34	2
mRNA	RBM47	2
mRNA	RCAN1	2
mRNA	RELB	2
mRNA	RELL1	2
mRNA	REPS2	2
mRNA	RFWD3	2
mRNA	RFX2	2
mRNA	RGMA	2
mRNA	RNF145	2
mRNA	RNF150	2
mRNA	RNF24	2
mRNA	RP9	2
mRNA	RPS14	2
mRNA	RPS6KC1	2
mRNA	RRM2	2
mRNA	RRN3	2
mRNA	RRP15	2
mRNA	RSBN1	2
mRNA	RSRC2	2
mRNA	SAE1	2
mRNA	SAMD10	2
mRNA	SAMSN1	2
mRNA	SASS6	2
mRNA	SCAI	2
mRNA	SCAMP5	2
mRNA	SCFD1	2
mRNA	SDC1	2
mRNA	SDCCAG3	2
mRNA	SEC23B	2
mRNA	SEC24D	2
mRNA	SEMA3A	2
mRNA	SEMA7A	2
mRNA	SEPN1	2
mRNA	8-Sep	2
mRNA	SERPINB8	2
mRNA	SESN1	2
mRNA	SGPL1	2
mRNA	SH2D2A	2
mRNA	SH3GL1	2
mRNA	SKA1	2

mRNA	ZNF77	1
mRNA	ZNF823	1
mRNA	ACOT12	1
mRNA	AGER	1
mRNA	ALDH16A1	1
mRNA	ALDH3B2	1
mRNA	AMER1	1
mRNA	ARL9	1
mRNA	BLVRB	1
mRNA	CA11	1
mRNA	CCSER1	1
mRNA	CEP152	1
mRNA	CLUH	1
mRNA	DLGAP3	1
mRNA	DNPH1	1
mRNA	EVI2A	1
mRNA	FGGY	1
mRNA	GCHFR	1
mRNA	GCSAM	1
mRNA	GLI1	1
mRNA	GPR157	1
mRNA	GPR21	1
mRNA	GTF2IRD2B	1
mRNA	HEMGN	1
mRNA	HNRNPDL	1
mRNA	JADE3	1
mRNA	MLNR	1
mRNA	MRPL21	1
mRNA	MRPL52	1
mRNA	MTFR2	1
mRNA	MYO1H	1
mRNA	NABP1	1
mRNA	NPRL2	1
mRNA	ORC1	1
mRNA	R3HCC1L	1
mRNA	RIC1	1
mRNA	SLC35F6	1
mRNA	SMDT1	1
mRNA	SSPN	1
mRNA	TLR3	1
mRNA	TLR6	1
mRNA	TMEM254	1
mRNA	TSGA10	1
mRNA	UQCC1	1
mRNA	UQCRC1	1
mRNA	ZBTB18	1
mRNA	ZP3	1
mRNA	PHACTR1	1
mRNA	PHF1	1
mRNA	PHGDH	1
mRNA	PHKA1	1
mRNA	PHYH	1
mRNA	PI4K2B	1
mRNA	PIEZO2	1
mRNA	PIK3CD	1
mRNA	PIM2	1
mRNA	PIP5K1A	1
mRNA	PIP5K1B	1
mRNA	PITPNC1	1
mRNA	PKMYT1	1
mRNA	PLA2G4A	1
mRNA	PLAA	1
mRNA	PLAT	1
mRNA	PLD2	1
mRNA	PLEC	1
mRNA	PLEKHA1	1
mRNA	PLEKHB1	1
mRNA	PLK1	1
mRNA	PLLP	1
mRNA	PLS1	1
mRNA	PLXNA2	1
mRNA	PMEPA1	1
mRNA	PMM2	1
mRNA	PNP	1
mRNA	PNPLA4	1
mRNA	PNRC1	1
mRNA	POC1A	1
mRNA	POLA1	1
mRNA	POLE	1
mRNA	POLR3G	1
mRNA	PPAPDC2	1
mRNA	PPIF	1
mRNA	PPIH	1
mRNA	PPOX	1
mRNA	PPP1R3E	1
mRNA	PRC1	1
mRNA	PRDM1	1
mRNA	PRDM16	1
mRNA	PRDX3	1
mRNA	PREX1	1
mRNA	PRIM2	1
mRNA	PRKCA	1
mRNA	PRKDC	1
mRNA	PRKRA	1
mRNA	PROS1	1
mRNA	PRRT3	1
mRNA	PRUNE	1
mRNA	PSMB3	1
mRNA	PSMB7	1
mRNA	PSMC6	1
mRNA	SLA	2
mRNA	SLAMF7	2
mRNA	SLBP	2
mRNA	SLC16A10	2
mRNA	SLC25A15	2
mRNA	SLC25A25	2
mRNA	SLC26A11	2
mRNA	SLC2A13	2
mRNA	SLC2A4RG	2
mRNA	SLC35B4	2
mRNA	SLC35D2	2
mRNA	SLC35F1	2
mRNA	SLC37A1	2
mRNA	SLC46A1	2
mRNA	SLC7A1	2
mRNA	SLC7A2	2
mRNA	SLC7A5	2
mRNA	SLCO5A1	2
mRNA	SMARCA2	2
mRNA	SMC2	2
mRNA	SMC4	2
mRNA	SMURF1	2
mRNA	SMYD2	2
mRNA	SNAI1	2
mRNA	SNRPC	2
mRNA	SNX29	2
mRNA	SNX4	2
mRNA	SOBP	2
mRNA	SORL1	2
mRNA	SOX5	2
mRNA	SPAG17	2
mRNA	SPAG7	2
mRNA	SPEF2	2
mRNA	SPG21	2
mRNA	SPRY4	2
mRNA	SPSB4	2
mRNA	SRF	2
mRNA	SRP19	2
mRNA	SRSF1	2
mRNA	SRSF12	2
mRNA	SSU72	2
mRNA	ST3GAL4	2
mRNA	ST3GAL5	2
mRNA	STARD4	2
mRNA	STAT6	2
mRNA	STC2	2
mRNA	STEAP3	2
mRNA	STK17A	2
mRNA	STK32B	2
mRNA	STMN1	2
mRNA	STOX1	2
mRNA	STRAP	2
mRNA	SUB1	2

mRNA	PSMD1	1	mRNA	SUFU	2
mRNA	PSMD11	1	mRNA	SUMO1	2
mRNA	PSMD12	1	mRNA	SUN2	2
mRNA	PSMD14	1	mRNA	SUV39H2	2
mRNA	PSMD3	1	mRNA	SYAP1	2
mRNA	PSMD4	1	mRNA	TAF5	2
mRNA	PSME4	1	mRNA	TARBP2	2
mRNA	PSRC1	1	mRNA	TBC1D14	2
mRNA	PTDSS1	1	mRNA	TBC1D2	2
mRNA	PTGER4	1	mRNA	TBC1D2B	2
mRNA	PTGS2	1	mRNA	TBX19	2
mRNA	PTK2B	1	mRNA	TCEAL7	2
mRNA	PTPLB	1	mRNA	TCF19	2
mRNA	PTPN1	1	mRNA	TCTEX1D2	2
mRNA	PTPN14	1	mRNA	TFB1M	2
mRNA	PTPN18	1	mRNA	TFCP2L1	2
mRNA	PTPN4	1	mRNA	TGFB1I1	2
mRNA	PTPRA	1	mRNA	TGM2	2
mRNA	PTPRF	1	mRNA	TMED9	2
mRNA	PTPRK	1	mRNA	TMEM106B	2
mRNA	PTPRM	1	mRNA	TMEM109	2
mRNA	PVRL2	1	mRNA	TMEM167A	2
mRNA	PYGO2	1	mRNA	TMEM201	2
mRNA	QARS	1	mRNA	TMEM242	2
mRNA	RAB11FIP5	1	mRNA	TMEM65	2
mRNA	RAB3D	1	mRNA	TMEM9	2
mRNA	RABEPK	1	mRNA	TMTC1	2
mRNA	RABGAP1L	1	mRNA	TNFAIP1	2
mRNA	RAD51B	1	mRNA	TNFAIP8	2
mRNA	RALA	1	mRNA	TNFRSF10B	2
mRNA	RALGPS1	1	mRNA	TNFRSF1B	2
mRNA	RAPGEF5	1	mRNA	TNFSF11	2
mRNA	RASGEF1B	1	mRNA	TNFSF12	2
mRNA	RASSF5	1	mRNA	TNIP2	2
mRNA	RBL1	1	mRNA	TNS3	2
mRNA	RBM10	1	mRNA	TOB1	2
mRNA	RBMS3	1	mRNA	TOM1L2	2
mRNA	RCC1	1	mRNA	TOMM22	2
mRNA	RDH10	1	mRNA	TOMM34	2
mRNA	RDH13	1	mRNA	TP53BP2	2
mRNA	REXO1	1	mRNA	TPCN1	2
mRNA	RFC5	1	mRNA	TPP1	2
mRNA	RFWD3	1	mRNA	TRAF4	2
mRNA	RFX2	1	mRNA	TRIM14	2
mRNA	RFXAP	1	mRNA	TRIM16	2
mRNA	RGL1	1	mRNA	TRIM4	2
mRNA	RHOB	1	mRNA	TRIM65	2
mRNA	RHOBTB1	1	mRNA	TSEN15	2
mRNA	RIOK3	1	mRNA	TSKU	2
mRNA	RNASEL	1	mRNA	TTC28	2
mRNA	RNF115	1	mRNA	TTC30B	2
mRNA	RNF170	1	mRNA	TTYH3	2
mRNA	RNF19A	1	mRNA	TUBB6	2

mRNA	RNF213	1	mRNA	TXNL4A	2
mRNA	RP9	1	mRNA	UBASH3B	2
mRNA	RPAP3	1	mRNA	UBE2C	2
mRNA	RPL23	1	mRNA	UBE2E2	2
mRNA	RPL39L	1	mRNA	UBTF	2
mRNA	RPP25	1	mRNA	UBXN8	2
mRNA	RPS23	1	mRNA	UHRF1BP1L	2
mRNA	RPS6KA2	1	mRNA	UQCR10	2
mRNA	RPS6KC1	1	mRNA	USP12	2
mRNA	RPSA	1	mRNA	USP13	2
mRNA	RRN3	1	mRNA	USP46	2
mRNA	SAAL1	1	mRNA	VAMP8	2
mRNA	SAMD12	1	mRNA	VASH1	2
mRNA	SAMD4B	1	mRNA	VAV3	2
mRNA	SAMSN1	1	mRNA	VBP1	2
mRNA	SAP30BP	1	mRNA	VDR	2
mRNA	SAP30L	1	mRNA	VGLL4	2
mRNA	SCAI	1	mRNA	VMP1	2
mRNA	SCAMP5	1	mRNA	VPS26B	2
mRNA	SCG5	1	mRNA	WBP2	2
mRNA	SCN9A	1	mRNA	WBP4	2
mRNA	SDC4	1	mRNA	WDR43	2
mRNA	SDCCAG3	1	mRNA	WDR5B	2
mRNA	SEC24D	1	mRNA	WDTC1	2
mRNA	SEMA4D	1	mRNA	WFS1	2
mRNA	SENP5	1	mRNA	WIBG	2
mRNA	10-Sep	1	mRNA	WLS	2
mRNA	SERP1	1	mRNA	WNK3	2
mRNA	SFMBT1	1	mRNA	WWC1	2
mRNA	SFT2D1	1	mRNA	WWTR1	2
mRNA	SFXN4	1	mRNA	XK	2
mRNA	SGCB	1	mRNA	XPO4	2
mRNA	SGK1	1	mRNA	XYLT1	2
mRNA	SGMS2	1	mRNA	YRDC	2
mRNA	SGOL1	1	mRNA	ZBTB17	2
mRNA	SGPL1	1	mRNA	ZC3HAV1L	2
mRNA	SH3BP4	1	mRNA	ZFP90	2
mRNA	SH3D19	1	mRNA	ZHX2	2
mRNA	SH3RF3	1	mRNA	ZHX3	2
mRNA	SHMT2	1	mRNA	ZKSCAN4	2
mRNA	SIDT1	1	mRNA	ZNF100	2
mRNA	SIX4	1	mRNA	ZNF138	2
mRNA	SKA1	1	mRNA	ZNF155	2
mRNA	SKA3	1	mRNA	ZNF22	2
mRNA	SKIL	1	mRNA	ZNF223	2
mRNA	SLA	1	mRNA	ZNF397	2
mRNA	SLBP	1	mRNA	ZNF485	2
mRNA	SLC11A2	1	mRNA	ZNF492	2
mRNA	SLC16A10	1	mRNA	ZNF516	2
mRNA	SLC1A4	1	mRNA	ZNF607	2
mRNA	SLC20A2	1	mRNA	ZNF672	2
mRNA	SLC25A15	1	mRNA	ZNF738	2
mRNA	SLC25A25	1	mRNA	ZNF77	2

mRNA	SLC27A3	1	mRNA	ZNF85	2
mRNA	SLC29A1	1	mRNA	ZNF93	2
mRNA	SLC2A13	1	mRNA	ZW10	2
mRNA	SLC2A4RG	1	mRNA	ZXDA	2
mRNA	SLC30A7	1	mRNA	ACOT12	2
mRNA	SLC30A9	1	mRNA	ALDH16A1	2
mRNA	SLC35D2	1	mRNA	ARL9	2
mRNA	SLC35E1	1	mRNA	ARSA	2
mRNA	SLC38A1	1	mRNA	AZIN2	2
mRNA	SLC40A1	1	mRNA	BEND5	2
mRNA	SLC41A2	1	mRNA	CCL5	2
mRNA	SLC44A5	1	mRNA	CHEK2	2
mRNA	SLC45A4	1	mRNA	GCSAM	2
mRNA	SLC5A3	1	mRNA	GCSAML	2
mRNA	SLC9A8	1	mRNA	HIST1H1A	2
mRNA	SLCO5A1	1	mRNA	HIST2H4B	2
mRNA	SMAD1	1	mRNA	JADE3	2
mRNA	SMAD3	1	mRNA	KLHL42	2
mRNA	SMAD5	1	mRNA	LAMC2	2
mRNA	SMG5	1	mRNA	MMP8	2
mRNA	SMYD2	1	mRNA	MRPL52	2
mRNA	SNAPC1	1	mRNA	R3HCC1L	2
mRNA	SNAPC4	1	mRNA	RSRP1	2
mRNA	SNRNP40	1	mRNA	SKIDA1	2
mRNA	SNRPF	1	mRNA	SMDT1	2
mRNA	SNTB1	1	mRNA	SUSD6	2
mRNA	SNX29	1	mRNA	TLR6	2
mRNA	SNX4	1	mRNA	TNIP3	2
mRNA	SNX7	1	mRNA	TPTE2	2
mRNA	SNX9	1	mRNA	UQCRC1	2
mRNA	SOCS1	1	mRNA	XIRP2	2
mRNA	SORBS1	1	mRNA	ZNF454	2
mRNA	SORD	1	mRNA	AADAT	1
mRNA	SPAG7	1	mRNA	ABCA5	1
mRNA	SPATS2	1	mRNA	ABCB6	1
mRNA	SPG21	1	mRNA	ABCC4	1
mRNA	SPIN4	1	mRNA	ABCD1	1
mRNA	SPPL2B	1	mRNA	ABHD15	1
mRNA	SPRYD3	1	mRNA	ABTB1	1
mRNA	SPSB4	1	mRNA	ACAA2	1
mRNA	SRF	1	mRNA	ACACB	1
mRNA	SRGAP1	1	mRNA	ACAD9	1
mRNA	SRP19	1	mRNA	ACOT9	1
mRNA	SRRT	1	mRNA	ACTA2	1
mRNA	SRSF1	1	mRNA	ACVR1C	1
mRNA	SRSF5	1	mRNA	ADA	1
mRNA	SSU72	1	mRNA	ADAM15	1
mRNA	ST3GAL4	1	mRNA	ADAM8	1
mRNA	STK17B	1	mRNA	ADAMTS3	1
mRNA	STK24	1	mRNA	ADAMTS7	1
mRNA	STMN1	1	mRNA	ADAT2	1
mRNA	STOX1	1	mRNA	ADCY9	1
mRNA	STRAP	1	mRNA	ADRBK1	1

mRNA	STX11	1	mRNA	ADRBK2	1
mRNA	SUB1	1	mRNA	AEN	1
mRNA	SUSD1	1	mRNA	AGAP6	1
mRNA	SUV39H1	1	mRNA	AGMAT	1
mRNA	SVIL	1	mRNA	AGPAT6	1
mRNA	SVIP	1	mRNA	AGTRAP	1
mRNA	SYAP1	1	mRNA	AIF1L	1
mRNA	SYBU	1	mRNA	AIM1	1
mRNA	SYNGR1	1	mRNA	AKAP7	1
mRNA	SYT1	1	mRNA	AKR7A2	1
mRNA	TAB3	1	mRNA	ALDH1A1	1
mRNA	TAGAP	1	mRNA	ALDH3B1	1
mRNA	TANK	1	mRNA	ALDH5A1	1
mRNA	TARS	1	mRNA	ALDH6A1	1
mRNA	TBC1D17	1	mRNA	ALG1	1
mRNA	TBC1D2B	1	mRNA	AMACR	1
mRNA	TBC1D9	1	mRNA	ANAPC1	1
mRNA	TCTEX1D2	1	mRNA	ANKAR	1
mRNA	TCTN1	1	mRNA	ANKRA2	1
mRNA	TDG	1	mRNA	ANO3	1
mRNA	TDRD7	1	mRNA	ANPEP	1
mRNA	TEAD4	1	mRNA	ANXA6	1
mRNA	TEX30	1	mRNA	AP1G2	1
mRNA	TFB1M	1	mRNA	APIP	1
mRNA	TFB2M	1	mRNA	APOBEC3B	1
mRNA	TFDP1	1	mRNA	APOL2	1
mRNA	TFR2	1	mRNA	ARHGAP10	1
mRNA	TGFBR2	1	mRNA	ARHGAP20	1
mRNA	TGM2	1	mRNA	ARHGAP6	1
mRNA	THAP11	1	mRNA	ARHGDIB	1
mRNA	THAP2	1	mRNA	ARL13B	1
mRNA	THBS1	1	mRNA	ARL5A	1
mRNA	THOP1	1	mRNA	ARMCX2	1
mRNA	TIAM1	1	mRNA	ARMCX6	1
mRNA	TIMP3	1	mRNA	ARSK	1
mRNA	TJP2	1	mRNA	ASAP2	1
mRNA	TLE1	1	mRNA	ASB13	1
mRNA	TLE4	1	mRNA	ASF1B	1
mRNA	TLN1	1	mRNA	ASIC1	1
mRNA	TM7SF3	1	mRNA	ASPHD1	1
mRNA	TMC7	1	mRNA	ATAD5	1
mRNA	TMCO4	1	mRNA	ATF5	1
mRNA	TMEFF1	1	mRNA	ATP5A1	1
mRNA	TMEM110	1	mRNA	ATP7B	1
mRNA	TMEM136	1	mRNA	AURKA	1
mRNA	TMEM167A	1	mRNA	AURKB	1
mRNA	TMEM180	1	mRNA	AVL9	1
mRNA	TMEM187	1	mRNA	B3GNT5	1
mRNA	TMEM208	1	mRNA	BAX	1
mRNA	TMEM39A	1	mRNA	BBS9	1
mRNA	TMEM45A	1	mRNA	BCAT2	1
mRNA	TMEM80	1	mRNA	BCCIP	1
mRNA	TMEM87B	1	mRNA	BCL2L12	1

mRNA	TMEM97	1
mRNA	TMOD2	1
mRNA	TMPO	1
mRNA	TNFAIP2	1
mRNA	TNFAIP3	1
mRNA	TNFRSF10D	1
mRNA	TNIK	1
mRNA	TNIP1	1
mRNA	TOB1	1
mRNA	TOM1L2	1
mRNA	TOMM22	1
mRNA	TOPBP1	1
mRNA	TOX	1
mRNA	TPCN1	1
mRNA	TRAPPC8	1
mRNA	TRAPPC9	1
mRNA	TRIB1	1
mRNA	TRIM16	1
mRNA	TRIM36	1
mRNA	TRIM45	1
mRNA	TRIM65	1
mRNA	TRIO	1
mRNA	TRIP10	1
mRNA	TRIP13	1
mRNA	TRIT1	1
mRNA	TRMT6	1
mRNA	TSHZ3	1
mRNA	TSKU	1
mRNA	TSPAN14	1
mRNA	TSR1	1
mRNA	TTC28	1
mRNA	TTC7B	1
mRNA	TTK	1
mRNA	TXLNB	1
mRNA	TXN	1
mRNA	TXNDC9	1
mRNA	UBASH3B	1
mRNA	UBE2E2	1
mRNA	UBE2H	1
mRNA	UBE2L6	1
mRNA	UBE2T	1
mRNA	UBXN2B	1
mRNA	UBXN8	1
mRNA	UEVLD	1
mRNA	UGCG	1
mRNA	UHRF1BP1	1
mRNA	ULK1	1
mRNA	UNC5B	1
mRNA	UPF3B	1
mRNA	URB2	1
mRNA	USP11	1
mRNA	USP13	1
mRNA	UTP14A	1
mRNA	BCL9L	1
mRNA	BDH1	1
mRNA	BEND6	1
mRNA	BEST1	1
mRNA	BHLHE41	1
mRNA	BIN1	1
mRNA	BIRC2	1
mRNA	BLOC1S1	1
mRNA	BMP4	1
mRNA	BMP8B	1
mRNA	BMPR1B	1
mRNA	BNIP1	1
mRNA	BOLA3	1
mRNA	BRCA1	1
mRNA	BRIP1	1
mRNA	BSCL2	1
mRNA	BTBD8	1
mRNA	BTG2	1
mRNA	BUB1B	1
mRNA	C10orf10	1
mRNA	C11orf49	1
mRNA	C14orf159	1
mRNA	C15orf39	1
mRNA	C16orf59	1
mRNA	C17orf49	1
mRNA	C17orf53	1
mRNA	C17orf75	1
mRNA	C21orf33	1
mRNA	C3	1
mRNA	C4orf19	1
mRNA	C8orf33	1
mRNA	C9orf9	1
mRNA	CA13	1
mRNA	CA8	1
mRNA	CABLES2	1
mRNA	CACNB2	1
mRNA	CAMK1D	1
mRNA	CARD16	1
mRNA	CARD6	1
mRNA	CASP10	1
mRNA	CBLN3	1
mRNA	CBS	1
mRNA	CBX6	1
mRNA	CCDC109B	1
mRNA	CCDC138	1
mRNA	CCDC15	1
mRNA	CCDC58	1
mRNA	CCDC59	1
mRNA	CCDC8	1
mRNA	CCNB1	1
mRNA	CCND3	1
mRNA	CCP110	1
mRNA	CCT5	1

mRNA	VANGL1	1
mRNA	VBP1	1
mRNA	VEGFA	1
mRNA	VKORC1L1	1
mRNA	VMP1	1
mRNA	VPS26B	1
mRNA	VPS37C	1
mRNA	WASF2	1
mRNA	WBP1	1
mRNA	WDFY3	1
mRNA	WDR12	1
mRNA	WDR35	1
mRNA	WDR5B	1
mRNA	WDR81	1
mRNA	WFS1	1
mRNA	WHAMM	1
mRNA	WLS	1
mRNA	WNT1	1
mRNA	WWTR1	1
mRNA	YARS	1
mRNA	YIPF6	1
mRNA	YPEL1	1
mRNA	YRDC	1
mRNA	YTHDF2	1
mRNA	ZBTB1	1
mRNA	ZBTB17	1
mRNA	ZBTB43	1
mRNA	ZDBF2	1
mRNA	ZDHHC2	1
mRNA	ZKSCAN3	1
mRNA	ZMAT3	1
mRNA	ZMPSTE24	1
mRNA	ZMYM3	1
mRNA	ZNF100	1
mRNA	ZNF117	1
mRNA	ZNF155	1
mRNA	ZNF22	1
mRNA	ZNF223	1
mRNA	ZNF230	1
mRNA	ZNF239	1
mRNA	ZNF250	1
mRNA	ZNF286A	1
mRNA	ZNF367	1
mRNA	ZNF492	1
mRNA	ZNF493	1
mRNA	ZNF569	1
mRNA	ZNF607	1
mRNA	ZNF672	1
mRNA	ZNF730	1
mRNA	ZNF823	1
mRNA	ZSWIM6	1
mRNA	ZW10	1
mRNA	ZWILCH	1
mRNA	CCT6A	1
mRNA	CCT8	1
mRNA	CD36	1
mRNA	CD4	1
mRNA	CD59	1
mRNA	CD70	1
mRNA	CD79A	1
mRNA	CD99	1
mRNA	CDCA2	1
mRNA	CDCA3	1
mRNA	CDCP1	1
mRNA	CDK2	1
mRNA	CDKN2A	1
mRNA	CDKN3	1
mRNA	CDYL2	1
mRNA	CENPM	1
mRNA	CENPP	1
mRNA	CENPV	1
mRNA	CEP41	1
mRNA	CEP68	1
mRNA	CERCAM	1
mRNA	CERK	1
mRNA	CFLAR	1
mRNA	CHAF1A	1
mRNA	CHFR	1
mRNA	CHMP4A	1
mRNA	CHN1	1
mRNA	CIB2	1
mRNA	CIDEB	1
mRNA	CKAP2L	1
mRNA	CKB	1
mRNA	CKS2	1
mRNA	CLCN4	1
mRNA	CLEC2D	1
mRNA	CLEC4D	1
mRNA	CLGN	1
mRNA	CLIC5	1
mRNA	CLIP1	1
mRNA	CLIP3	1
mRNA	CLU	1
mRNA	CMTM7	1
mRNA	CNNM3	1
mRNA	CNPY4	1
mRNA	COL24A1	1
mRNA	COPB1	1
mRNA	COTL1	1
mRNA	COX11	1
mRNA	COX17	1
mRNA	COX7B	1
mRNA	CPSF3	1
mRNA	CPVL	1
mRNA	CREB3L4	1
mRNA	CRELD1	1

mRNA	AAAS	1
mRNA	ACP6	1
mRNA	ADAMTS9	1
mRNA	ARL9	1
mRNA	BCS1L	1
mRNA	BEND5	1
mRNA	C14orf93	1
mRNA	CCDC171	1
mRNA	CCSER1	1
mRNA	CD34	1
mRNA	CD8A	1
mRNA	CDH9	1
mRNA	CEP152	1
mRNA	CH25H	1
mRNA	CHEK2	1
mRNA	CXCL13	1
mRNA	DLGAP3	1
mRNA	ELMSAN1	1
mRNA	EXO5	1
mRNA	FAM213A	1
mRNA	GAS2	1
mRNA	GCSAM	1
mRNA	GCSAML	1
mRNA	GPLD1	1
mRNA	GPR155	1
mRNA	GSTM5	1
mRNA	GTF2IRD2B	1
mRNA	HGD	1
mRNA	HIST1H1A	1
mRNA	HIST1H2AM	1
mRNA	HIST2H3C	1
mRNA	IL1R2	1
mRNA	IL24	1
mRNA	KCNA4	1
mRNA	KLHL42	1
mRNA	LAMC2	1
mRNA	MMP10	1
mRNA	MMP8	1
mRNA	MTFR2	1
mRNA	NABP1	1
mRNA	NSMF	1
mRNA	PDE1B	1
mRNA	RMDN3	1
mRNA	ROPN1L	1
mRNA	RPL21	1
mRNA	RPS2	1
mRNA	RRP9	1
mRNA	RSG1	1
mRNA	SDE2	1
mRNA	SERPINB2	1
mRNA	SGIP1	1
mRNA	SKIDA1	1
mRNA	TLR3	1
mRNA	CSRP1	1
mRNA	CSRP2BP	1
mRNA	CTDSPL	1
mRNA	CTNNAL1	1
mRNA	CTSD	1
mRNA	CTSO	1
mRNA	CUEDC2	1
mRNA	CXCL10	1
mRNA	CXorf57	1
mRNA	CYC1	1
mRNA	CYP4V2	1
mRNA	CYTH2	1
mRNA	CYTIP	1
mRNA	DACT1	1
mRNA	DAGLA	1
mRNA	DAP3	1
mRNA	DBP	1
mRNA	DCHS1	1
mRNA	DCUN1D3	1
mRNA	DDB2	1
mRNA	DDX60	1
mRNA	DERA	1
mRNA	DHFR	1
mRNA	DHRS4L2	1
mRNA	DHX38	1
mRNA	DIS3L	1
mRNA	DIXDC1	1
mRNA	DKC1	1
mRNA	DMKN	1
mRNA	DMRTA2	1
mRNA	DNAH10	1
mRNA	DNAJB5	1
mRNA	DNMT1	1
mRNA	DONSON	1
mRNA	DPF1	1
mRNA	DPM2	1
mRNA	DPY19L3	1
mRNA	DRAP1	1
mRNA	DRG1	1
mRNA	DSEL	1
mRNA	DTWD1	1
mRNA	DUSP1	1
mRNA	DUSP12	1
mRNA	DUSP16	1
mRNA	DUSP19	1
mRNA	DYNC1I2	1
mRNA	DYRK1B	1
mRNA	DZIP1	1
mRNA	DZIP3	1
mRNA	E2F1	1
mRNA	E2F2	1
mRNA	ECH1	1
mRNA	ECHDC2	1

mRNA	TNIP3	1	mRNA	EEPD1	1
mRNA	TPK1	1	mRNA	EFNA4	1
mRNA	UBA7	1	mRNA	EHD3	1
mRNA	WDR45B	1	mRNA	EHD4	1
mRNA	ZNF454	1	mRNA	EHMT2	1
			mRNA	EIF2AK4	1
			mRNA	EIF2S1	1
			mRNA	ELL	1
			mRNA	EMB	1
			mRNA	EME1	1
			mRNA	ENTPD5	1
			mRNA	EPHA2	1
			mRNA	ERI3	1
			mRNA	ESCO2	1
			mRNA	ESYT1	1
			mRNA	ETFB	1
			mRNA	ETV4	1
			mRNA	EXOSC1	1
			mRNA	EXOSC5	1
			mRNA	EXOSC9	1
			mRNA	EXTL2	1
			mRNA	EXTL3	1
			mRNA	EYA3	1
			mRNA	F11R	1
			mRNA	FABP5	1
			mRNA	FAIM3	1
			mRNA	FAM110B	1
			mRNA	FAM117A	1
			mRNA	FAM124A	1
			mRNA	FAM127A	1
			mRNA	FAM127B	1
			mRNA	FAM127C	1
			mRNA	FAM171B	1
			mRNA	FAM173B	1
			mRNA	FAM175A	1
			mRNA	FAM198B	1
			mRNA	FAM200A	1
			mRNA	FAM20C	1
			mRNA	FAM217B	1
			mRNA	FAM219A	1
			mRNA	FAM49A	1
			mRNA	FAM58A	1
			mRNA	FAM64A	1
			mRNA	FAM65B	1
			mRNA	FAM83D	1
			mRNA	FAM92A1	1
			mRNA	FANCC	1
			mRNA	FANCE	1
			mRNA	FBXL5	1
			mRNA	FBXL6	1
			mRNA	FBXO22	1
			mRNA	FBXO5	1
			mRNA	FCHO1	1

		mRNA	FERMT1	1
		mRNA	FGL2	1
		mRNA	FIGNL1	1
		mRNA	FKBP10	1
		mRNA	FKBP1B	1
		mRNA	FLOT2	1
		mRNA	FN3KRP	1
		mRNA	FNTB	1
		mRNA	FOS	1
		mRNA	FOSL1	1
		mRNA	FOSL2	1
		mRNA	FOXRED2	1
		mRNA	FRMD4A	1
		mRNA	FSD1	1
		mRNA	FSIP1	1
		mRNA	FUCA1	1
		mRNA	FXN	1
		mRNA	FXYD5	1
		mRNA	GAB2	1
		mRNA	GADD45B	1
		mRNA	GALNT12	1
		mRNA	GAPT	1
		mRNA	GARS	1
		mRNA	GART	1
		mRNA	GAS2L3	1
		mRNA	GAS7	1
		mRNA	GCA	1
		mRNA	GDE1	1
		mRNA	GDF15	1
		mRNA	GFI1	1
		mRNA	GFPT1	1
		mRNA	GINS4	1
		mRNA	GIPC2	1
		mRNA	GLB1L2	1
		mRNA	GLI2	1
		mRNA	GLMN	1
		mRNA	GLRB	1
		mRNA	GLRX	1
		mRNA	GLUL	1
		mRNA	GM2A	1
		mRNA	GMDS	1
		mRNA	GMPPB	1
		mRNA	GMPR	1
		mRNA	GMPS	1
		mRNA	GPR180	1
		mRNA	GRB10	1
		mRNA	GSPT2	1
		mRNA	GSR	1
		mRNA	GSTK1	1
		mRNA	GTF2IRD2	1
		mRNA	GUCY1A3	1
		mRNA	GUF1	1
		mRNA	H2AFJ	1

		mRNA	H2AFX	1
		mRNA	H2AFY	1
		mRNA	HABP4	1
		mRNA	HAPLN3	1
		mRNA	HAUS1	1
		mRNA	HAUS2	1
		mRNA	HAUS4	1
		mRNA	HAUS6	1
		mRNA	HECTD3	1
		mRNA	HIST1H2BG	1
		mRNA	HIST1H3B	1
		mRNA	HIST2H2BE	1
		mRNA	HK2	1
		mRNA	HLA-C	1
		mRNA	HLA-DPB1	1
		mRNA	HLA-DQB1	1
		mRNA	HLA-E	1
		mRNA	HLA-F	1
		mRNA	HMGB1	1
		mRNA	HMMR	1
		mRNA	HN1	1
		mRNA	HOPX	1
		mRNA	HOXA4	1
		mRNA	HOXB2	1
		mRNA	HPDL	1
		mRNA	HPGD	1
		mRNA	HRSP12	1
		mRNA	HS3ST1	1
		mRNA	HSPA4	1
		mRNA	HSPE1	1
		mRNA	HYOU1	1
		mRNA	ID1	1
		mRNA	IFI16	1
		mRNA	IFI30	1
		mRNA	IFIT1	1
		mRNA	IFIT2	1
		mRNA	IGBP1	1
		mRNA	IGF2BP2	1
		mRNA	IL12A	1
		mRNA	IL13RA1	1
		mRNA	IL15RA	1
		mRNA	IL16	1
		mRNA	IL1R1	1
		mRNA	IL2RB	1
		mRNA	IL32	1
		mRNA	ILDR2	1
		mRNA	IMPA2	1
		mRNA	INA	1
		mRNA	INF2	1
		mRNA	ING2	1
		mRNA	INPP5K	1
		mRNA	IQGAP3	1
		mRNA	IRAK2	1

		mRNA	ITGA1	1
		mRNA	ITGB5	1
		mRNA	JUNB	1
		mRNA	KATNB1	1
		mRNA	KCNK2	1
		mRNA	KCNMB1	1
		mRNA	KIAA0101	1
		mRNA	KIAA1211	1
		mRNA	KIAA1377	1
		mRNA	KIAA2022	1
		mRNA	KIF18A	1
		mRNA	KIF18B	1
		mRNA	KIF20B	1
		mRNA	KIF22	1
		mRNA	KIF2A	1
		mRNA	KIF2C	1
		mRNA	KIRREL	1
		mRNA	KLC2	1
		mRNA	KLHDC8B	1
		mRNA	KLHL3	1
		mRNA	KLHL36	1
		mRNA	L2HGDH	1
		mRNA	LAMA2	1
		mRNA	LAMA3	1
		mRNA	LAMTOR1	1
		mRNA	LAT2	1
		mRNA	LETMD1	1
		mRNA	LFNG	1
		mRNA	LGMN	1
		mRNA	LIF	1
		mRNA	LILRB1	1
		mRNA	LIMK2	1
		mRNA	LIMS3	1
		mRNA	LIPT1	1
		mRNA	LLGL1	1
		mRNA	LONRF2	1
		mRNA	LOXL1	1
		mRNA	LPL	1
		mRNA	LRMP	1
		mRNA	LRP2BP	1
		mRNA	LRRK2	1
		mRNA	LSM11	1
		mRNA	LSM4	1
		mRNA	LTA	1
		mRNA	LTA4H	1
		mRNA	LTBP3	1
		mRNA	LURAP1L	1
		mRNA	LXN	1
		mRNA	LY6E	1
		mRNA	LYRM5	1
		mRNA	MACC1	1
		mRNA	MAMLD1	1
		mRNA	MAN1B1	1

		mRNA	MAN1C1	1
		mRNA	MANBA	1
		mRNA	MAP2K1	1
		mRNA	MAP4K1	1
		mRNA	MAP6D1	1
		mRNA	MARS	1
		mRNA	MARVELD1	1
		mRNA	MBOAT1	1
		mRNA	MBOAT2	1
		mRNA	MCAM	1
		mRNA	MCM3	1
		mRNA	MCM8	1
		mRNA	MDK	1
		mRNA	MDM2	1
		mRNA	METTL13	1
		mRNA	MEX3D	1
		mRNA	MFSD9	1
		mRNA	MGAT3	1
		mRNA	MGST3	1
		mRNA	MICA	1
		mRNA	MICAL2	1
		mRNA	MIPOL1	1
		mRNA	MKI67	1
		mRNA	MMP14	1
		mRNA	MMS22L	1
		mRNA	MOB3B	1
		mRNA	MOB3C	1
		mRNA	MOSPD3	1
		mRNA	MPI	1
		mRNA	MPP2	1
		mRNA	MPV17L	1
		mRNA	MPZL2	1
		mRNA	MRE11A	1
		mRNA	MRPL13	1
		mRNA	MRPL16	1
		mRNA	MRPL3	1
		mRNA	MRPL39	1
		mRNA	MRPS34	1
		mRNA	MRTO4	1
		mRNA	MSH6	1
		mRNA	MSRB2	1
		mRNA	MSRB3	1
		mRNA	MTG1	1
		mRNA	MTRF1L	1
		mRNA	MUC1	1
		mRNA	MXD4	1
		mRNA	MYBL2	1
		mRNA	MYD88	1
		mRNA	MYO1D	1
		mRNA	MYO1G	1
		mRNA	MYOF	1
		mRNA	MYOZ3	1
		mRNA	N6AMT2	1

mRNA	NAA38	1
mRNA	NAGLU	1
mRNA	NAP1L2	1
mRNA	NAPB	1
mRNA	NASP	1
mRNA	NCAPD2	1
mRNA	NCAPD3	1
mRNA	NCOR2	1
mRNA	NDUFB6	1
mRNA	NDUFS6	1
mRNA	NECAB3	1
mRNA	NEFH	1
mRNA	NEK2	1
mRNA	NEK6	1
mRNA	NFIL3	1
mRNA	NFKB2	1
mRNA	NFYB	1
mRNA	NHEJ1	1
mRNA	NIPAL2	1
mRNA	NIPAL3	1
mRNA	NLN	1
mRNA	NOP2	1
mRNA	NRCAM	1
mRNA	NREP	1
mRNA	NSUN7	1
mRNA	NTN1	1
mRNA	NUDT6	1
mRNA	NUF2	1
mRNA	NUP107	1
mRNA	NUP155	1
mRNA	NUP88	1
mRNA	NXPH4	1
mRNA	NYNRIN	1
mRNA	OAS1	1
mRNA	OGG1	1
mRNA	OPRL1	1
mRNA	OPTN	1
mRNA	ORAI2	1
mRNA	ORAI3	1
mRNA	OSBPL10	1
mRNA	OSGEPL1	1
mRNA	OTUD5	1
mRNA	P2RY10	1
mRNA	PA2G4	1
mRNA	PAFAH1B3	1
mRNA	PAIP2B	1
mRNA	PAK1IP1	1
mRNA	PALM2-AKAP[illegible]	1
mRNA	PAQR4	1
mRNA	PAQR5	1
mRNA	PARD3B	1
mRNA	PARK2	1
mRNA	PARP12	1

		mRNA	PARP16	1
		mRNA	PBK	1
		mRNA	PCDH17	1
		mRNA	PCNA	1
		mRNA	PCNXL2	1
		mRNA	PCSK5	1
		mRNA	PCYT1A	1
		mRNA	PDAP1	1
		mRNA	PDE4A	1
		mRNA	PDGFD	1
		mRNA	PDLIM1	1
		mRNA	PDXP	1
		mRNA	PECR	1
		mRNA	PERP	1
		mRNA	PEX6	1
		mRNA	PHGDH	1
		mRNA	PIGM	1
		mRNA	PIK3CD	1
		mRNA	PIK3CG	1
		mRNA	PIK3IP1	1
		mRNA	PIK3R5	1
		mRNA	PIM2	1
		mRNA	PKMYT1	1
		mRNA	PLAT	1
		mRNA	PLAUR	1
		mRNA	PLCL2	1
		mRNA	PLD2	1
		mRNA	PLEC	1
		mRNA	PLEK2	1
		mRNA	PLEKHA7	1
		mRNA	PLEKHB1	1
		mRNA	PLK2	1
		mRNA	PLK3	1
		mRNA	PLXNA1	1
		mRNA	PLXNA2	1
		mRNA	PLXNA3	1
		mRNA	PLXNC1	1
		mRNA	PNPLA4	1
		mRNA	PNPLA6	1
		mRNA	PNPO	1
		mRNA	PNPT1	1
		mRNA	POFUT2	1
		mRNA	POLE2	1
		mRNA	POLE3	1
		mRNA	POLQ	1
		mRNA	POLR2E	1
		mRNA	POLR3B	1
		mRNA	POLR3G	1
		mRNA	PPM1G	1
		mRNA	PPP1R3E	1
		mRNA	PQLC3	1
		mRNA	PRICKLE1	1
		mRNA	PRKCA	1

		mRNA	PRKDC	1
		mRNA	PRMT2	1
		mRNA	PRMT5	1
		mRNA	PRPF18	1
		mRNA	PRR15	1
		mRNA	PRUNE	1
		mRNA	PSEN2	1
		mRNA	PSMA5	1
		mRNA	PSMB3	1
		mRNA	PSMD11	1
		mRNA	PSMD3	1
		mRNA	PSMD4	1
		mRNA	PSMD9	1
		mRNA	PTPLAD2	1
		mRNA	PTPN18	1
		mRNA	PUS7	1
		mRNA	PVRL2	1
		mRNA	PYCRL	1
		mRNA	PYGL	1
		mRNA	PYGO2	1
		mRNA	QARS	1
		mRNA	QSOX2	1
		mRNA	R3HDM4	1
		mRNA	RAB11FIP4	1
		mRNA	RAB13	1
		mRNA	RAB27B	1
		mRNA	RAB31	1
		mRNA	RAB3D	1
		mRNA	RAE1	1
		mRNA	RALB	1
		mRNA	RALGPS1	1
		mRNA	RANBP1	1
		mRNA	RANBP10	1
		mRNA	RAP1GAP2	1
		mRNA	RAPGEF5	1
		mRNA	RBM38	1
		mRNA	RCAN3	1
		mRNA	RCL1	1
		mRNA	RDH13	1
		mRNA	REL	1
		mRNA	RFC5	1
		mRNA	RFXAP	1
		mRNA	RHOBTB1	1
		mRNA	RNASEL	1
		mRNA	RNF121	1
		mRNA	RNF187	1
		mRNA	RNF19B	1
		mRNA	RPA1	1
		mRNA	RPL17	1
		mRNA	RPL32	1
		mRNA	RPL34	1
		mRNA	RPP25	1
		mRNA	RPS23	1

		mRNA	RPS6KA2	1
		mRNA	RPS6KA6	1
		mRNA	RUVBL1	1
		mRNA	RUVBL2	1
		mRNA	RWDD2B	1
		mRNA	SAAL1	1
		mRNA	SAMD12	1
		mRNA	SAMD4B	1
		mRNA	SAMD9L	1
		mRNA	SAP30L	1
		mRNA	SARS2	1
		mRNA	SCARB1	1
		mRNA	SCG5	1
		mRNA	SCN4B	1
		mRNA	SCP2	1
		mRNA	SDF2L1	1
		mRNA	SEC61A2	1
		mRNA	10-Sep	1
		mRNA	6-Sep	1
		mRNA	SERPINE2	1
		mRNA	SFMBT1	1
		mRNA	SFMBT2	1
		mRNA	SFT2D1	1
		mRNA	SFXN3	1
		mRNA	SGCB	1
		mRNA	SGOL2	1
		mRNA	SGSH	1
		mRNA	SH2D4A	1
		mRNA	SH3D19	1
		mRNA	SH3GL3	1
		mRNA	SH3RF1	1
		mRNA	SH3RF3	1
		mRNA	SHPK	1
		mRNA	SIDT1	1
		mRNA	SIPA1L1	1
		mRNA	SIVA1	1
		mRNA	SKA2	1
		mRNA	SKA3	1
		mRNA	SKAP2	1
		mRNA	SLAMF1	1
		mRNA	SLC12A4	1
		mRNA	SLC12A9	1
		mRNA	SLC15A3	1
		mRNA	SLC1A5	1
		mRNA	SLC24A1	1
		mRNA	SLC25A33	1
		mRNA	SLC25A45	1
		mRNA	SLC29A1	1
		mRNA	SLC30A4	1
		mRNA	SLC37A4	1
		mRNA	SLC39A11	1
		mRNA	SLC39A13	1
		mRNA	SLC39A7	1

		mRNA	SLC40A1	1
		mRNA	SLC45A4	1
		mRNA	SLC48A1	1
		mRNA	SLC5A6	1
		mRNA	SLC6A9	1
		mRNA	SLFN12	1
		mRNA	SMC1A	1
		mRNA	SMG5	1
		mRNA	SMO	1
		mRNA	SNAPC1	1
		mRNA	SNRPF	1
		mRNA	SNX7	1
		mRNA	SOCS1	1
		mRNA	SPAG5	1
		mRNA	SPOCK3	1
		mRNA	SPPL2B	1
		mRNA	SRPRB	1
		mRNA	SRSF10	1
		mRNA	ST8SIA4	1
		mRNA	STARD8	1
		mRNA	STIL	1
		mRNA	STX11	1
		mRNA	STYK1	1
		mRNA	SUOX	1
		mRNA	SUSD1	1
		mRNA	SUSD3	1
		mRNA	SUV39H1	1
		mRNA	SVIP	1
		mRNA	SYNGR1	1
		mRNA	SYP	1
		mRNA	SYT11	1
		mRNA	TACC3	1
		mRNA	TAGAP	1
		mRNA	TARBP1	1
		mRNA	TBC1D16	1
		mRNA	TBC1D17	1
		mRNA	TCEAL1	1
		mRNA	TCF7	1
		mRNA	TCP11L2	1
		mRNA	TCTN1	1
		mRNA	TET1	1
		mRNA	TEX30	1
		mRNA	TFDP1	1
		mRNA	TFEC	1
		mRNA	TFR2	1
		mRNA	THAP2	1
		mRNA	THOP1	1
		mRNA	THRA	1
		mRNA	THY1	1
		mRNA	TIMM10	1
		mRNA	TIMM17A	1
		mRNA	TIMM22	1
		mRNA	TIMM8B	1

		mRNA	TIMP3	1
		mRNA	TIPIN	1
		mRNA	TLN1	1
		mRNA	TM4SF1	1
		mRNA	TM7SF3	1
		mRNA	TMED4	1
		mRNA	TMED8	1
		mRNA	TMEM110	1
		mRNA	TMEM134	1
		mRNA	TMEM136	1
		mRNA	TMEM141	1
		mRNA	TMEM187	1
		mRNA	TMEM194B	1
		mRNA	TMEM39A	1
		mRNA	TMEM80	1
		mRNA	TNFAIP2	1
		mRNA	TNFRSF8	1
		mRNA	TNFSF15	1
		mRNA	TNIK	1
		mRNA	TP53	1
		mRNA	TPX2	1
		mRNA	TRIT1	1
		mRNA	TRMT6	1
		mRNA	TROAP	1
		mRNA	TSPAN2	1
		mRNA	TSPAN7	1
		mRNA	TSPO	1
		mRNA	TSPYL1	1
		mRNA	TTK	1
		mRNA	TUBD1	1
		mRNA	TUBE1	1
		mRNA	TUBGCP3	1
		mRNA	TUFM	1
		mRNA	TXLNB	1
		mRNA	TXN	1
		mRNA	TYSND1	1
		mRNA	UBE2L6	1
		mRNA	UBE2T	1
		mRNA	UBL4A	1
		mRNA	UBR7	1
		mRNA	UBXN2B	1
		mRNA	UCHL5	1
		mRNA	UEVLD	1
		mRNA	UFD1L	1
		mRNA	UHRF1	1
		mRNA	UHRF1BP1	1
		mRNA	UNG	1
		mRNA	UPF3B	1
		mRNA	UQCRH	1
		mRNA	USP2	1
		mRNA	USP51	1
		mRNA	VAMP4	1
		mRNA	VANGL1	1

		mRNA	VKORC1	1
		mRNA	VPS37C	1
		mRNA	WARS	1
		mRNA	WDR12	1
		mRNA	WDR35	1
		mRNA	WDR54	1
		mRNA	WDR77	1
		mRNA	WDR81	1
		mRNA	WIPI1	1
		mRNA	XKR8	1
		mRNA	XPOT	1
		mRNA	XRCC4	1
		mRNA	XRRA1	1
		mRNA	YARS	1
		mRNA	YIF1B	1
		mRNA	YTHDC2	1
		mRNA	ZBTB1	1
		mRNA	ZBTB20	1
		mRNA	ZBTB38	1
		mRNA	ZC3H12A	1
		mRNA	ZDHHC1	1
		mRNA	ZFAND2A	1
		mRNA	ZFP36	1
		mRNA	ZKSCAN3	1
		mRNA	ZMPSTE24	1
		mRNA	ZMYM3	1
		mRNA	ZNF2	1
		mRNA	ZNF239	1
		mRNA	ZNF286A	1
		mRNA	ZNF493	1
		mRNA	ZNF513	1
		mRNA	ZNF528	1
		mRNA	ZNF566	1
		mRNA	ZNF569	1
		mRNA	ZNF583	1
		mRNA	ZNF615	1
		mRNA	ZNF622	1
		mRNA	ZNF681	1
		mRNA	ZNF724P	1
		mRNA	ZNF730	1
		mRNA	ZNF823	1
		mRNA	ZNF844	1
		mRNA	ZWILCH	1
		mRNA	ZWINT	1
		mRNA	AAAS	1
		mRNA	ADAMTS9	1
		mRNA	ADRM1	1
		mRNA	AGER	1
		mRNA	AGO2	1
		mRNA	ALDH3B2	1
		mRNA	AMER1	1
		mRNA	ARID5A	1
		mRNA	ATP1A3	1

		mRNA	BCAS3	1
		mRNA	BCS1L	1
		mRNA	BLOC1S4	1
		mRNA	BSPRY	1
		mRNA	C14orf93	1
		mRNA	CA11	1
		mRNA	CCDC169	1
		mRNA	CCDC171	1
		mRNA	CD244	1
		mRNA	CD8A	1
		mRNA	CHRFAM7A	1
		mRNA	CLUH	1
		mRNA	CRHBP	1
		mRNA	CTPS1	1
		mRNA	CXCL13	1
		mRNA	DLGAP3	1
		mRNA	DNAAF5	1
		mRNA	ELMSAN1	1
		mRNA	ERP27	1
		mRNA	EXO5	1
		mRNA	FAM213A	1
		mRNA	FAM229B	1
		mRNA	FANCG	1
		mRNA	FGGY	1
		mRNA	FMN1	1
		mRNA	GCHFR	1
		mRNA	GIMAP4	1
		mRNA	GPLD1	1
		mRNA	GPR155	1
		mRNA	GPR157	1
		mRNA	GPR19	1
		mRNA	GSTM5	1
		mRNA	GTF2IRD2B	1
		mRNA	HEMGN	1
		mRNA	HGD	1
		mRNA	HIST2H3C	1
		mRNA	HLF	1
		mRNA	HNRNPDL	1
		mRNA	HSD17B8	1
		mRNA	IDNK	1
		mRNA	IL24	1
		mRNA	KIAA1958	1
		mRNA	KRTAP5-3	1
		mRNA	KRTAP5-7	1
		mRNA	LAMTOR2	1
		mRNA	LANCL3	1
		mRNA	LRRC63	1
		mRNA	MMP10	1
		mRNA	MMP9	1
		mRNA	MRPL21	1
		mRNA	MUC4	1
		mRNA	MYO1H	1
		mRNA	NDC1	1

		mRNA	NSMF	1
		mRNA	PDE1B	1
		mRNA	PRIMPOL	1
		mRNA	PTGIS	1
		mRNA	RASEF	1
		mRNA	RPF2	1
		mRNA	RPL21	1
		mRNA	RRP9	1
		mRNA	S100B	1
		mRNA	SGIP1	1
		mRNA	SLC35F6	1
		mRNA	SLIRP	1
		mRNA	TCAF2	1
		mRNA	TMEM239	1
		mRNA	TPK1	1
		mRNA	UBA7	1
		mRNA	WDR45B	1
		mRNA	WDR66	1
		mRNA	ZBTB16	1
		mRNA	ZBTB18	1
		mRNA	ZNF391	1
		mRNA	ZP3	1
