# Supplementary material for: Identifying Dysregulated lncRNA-Associated ceRNA Network Biomarkers in CML Based on Dynamical Network Biomarkers
Source: Biomed Res Int. 2020 Feb 18;2020:5189549. doi: 10.1155/2020/5189549 (PMC7049421; doi:10.1155/2020/5189549)
Supplement: Supplementary Materials — Supplementary Table 1: dysregulated lncRNA-mRNA competing interactions of CP, AP, BC for CML. Supplementary Table 2: dysregulated lncRNA-associated ceRNA networks of CP, AP, and BC for CML (DLCN_CP, DLCN_AP, and DLCN_BC). Supplementary Table 3: CeRNA network biomarkers in DLCN_CP, DLCN_AP, and DLCN_BC. Supplementary Table 4: significantly enriched pathways in DLCN_CP, DLCN_AP, and DLCN_BC. . [file 5189549.f1.zip › 5189549.f1/Supplementary Table S3.pdf]

Supplementary Table 3: CeRNA network biomarkers in DLCN_CP, DLCN_AP, DLCN BC.

CeRNA network biomarkers in DLCN_CP		CeRNA network biomarkers in DLCN_AP		CeRNA network biomarkers in DLCN_BC	
Node type	Node name	Node type	Node name	Node type	Node name
lncRNA	SNHG5	lncRNA	DLEU2	lncRNA	SNHG5
mRNA	ACVR1C	mRNA	BCOR	lncRNA	SNHG3
mRNA	ECHDC2	mRNA	MMD	mRNA	ARHGAP32
mRNA	ITM2B	mRNA	SLC35F1	mRNA	CDH1
mRNA	KIAA1598			mRNA	EPSTI1
mRNA	SGMS1			mRNA	ITM2B
				mRNA	NUP62CL
				mRNA	PARP9
				mRNA	RAB3D
				mRNA	RPL13
				mRNA	RPS14
				mRNA	RPSA
				mRNA	SGMS1
				mRNA	SH3BP4
				mRNA	SYDE2
				mRNA	TMEM187
				mRNA	TTC28
