# Supplementary material for: Identifying Dysregulated lncRNA-Associated ceRNA Network Biomarkers in CML Based on Dynamical Network Biomarkers
Source: Biomed Res Int. 2020 Feb 18;2020:5189549. doi: 10.1155/2020/5189549 (PMC7049421; doi:10.1155/2020/5189549)
Supplement: Supplementary Materials — Supplementary Table 1: dysregulated lncRNA-mRNA competing interactions of CP, AP, BC for CML. Supplementary Table 2: dysregulated lncRNA-associated ceRNA networks of CP, AP, and BC for CML (DLCN_CP, DLCN_AP, and DLCN_BC). Supplementary Table 3: CeRNA network biomarkers in DLCN_CP, DLCN_AP, and DLCN_BC. Supplementary Table 4: significantly enriched pathways in DLCN_CP, DLCN_AP, and DLCN_BC. . [file 5189549.f1.zip › 5189549.f1/Supplementary Table S4.pdf]

Supplementary Table 4: Significantly enriched pathways in DLCN_CP, DLCN_AP, DLCN_BC.

Significantly enriched pathways in DLCN_CP		
Category	Term	P-Value
KEGG_PATHWAY	hsa04720:Long-term potentiation	0.00091096
KEGG_PATHWAY	hsa04916:Melanogenesis	0.00203195
KEGG_PATHWAY	hsa04722:Neurotrophin signaling pathway	0.002888384
KEGG_PATHWAY	hsa04114:Oocyte meiosis	0.003029141
KEGG_PATHWAY	hsa04550:Signaling pathways regulating pluripotency of stem cells	0.003993752
KEGG_PATHWAY	hsa04310:Wnt signaling pathway	0.004047987
KEGG_PATHWAY	hsa04390:Hippo signaling pathway	0.004667156
KEGG_PATHWAY	hsa04150:mTOR signaling pathway	0.004667156
KEGG_PATHWAY	hsa04360:Axon guidance	0.006027904
KEGG_PATHWAY	hsa05202:Transcriptional misregulation in cancer	0.006292572
KEGG_PATHWAY	hsa05205:Proteoglycans in cancer	0.008064462
KEGG_PATHWAY	hsa05166:HTLV-I infection	0.012559975
KEGG_PATHWAY	hsa05206:MicroRNAs in cancer	0.016448949
KEGG_PATHWAY	hsa00601:Glycosphingolipid biosynthesis - lacto and neolacto series	0.017500984
KEGG_PATHWAY	hsa04392:Hippo signaling pathway -multiple species	0.019427259
KEGG_PATHWAY	hsa00600:Sphingolipid metabolism	0.03090887
KEGG_PATHWAY	hsa04978:Mineral absorption	0.034075195
KEGG_PATHWAY	hsa05217:Basal cell carcinoma	0.035970213
KEGG_PATHWAY	hsa05214:Glioma	0.042261155
KEGG_PATHWAY	hsa05031:Amphetamine addiction	0.043514598
KEGG_PATHWAY	hsa04115:p53 signaling pathway	0.044766463
KEGG_PATHWAY	hsa04971:Gastric acid secretion	0.047889238
KEGG_PATHWAY	hsa01524:Platinum drug resistance	0.048512614

Significantly enriched pathways in DLCN_AP		
Category	Term	P-Value
KEGG_PATHWAY	hsa04115:p53 signaling pathway	0.000180121
KEGG_PATHWAY	hsa05200:Pathways in cancer	0.003168035
KEGG_PATHWAY	hsa05217:Basal cell carcinoma	0.003378333
KEGG_PATHWAY	hsa03015:mRNA surveillance pathway	0.008928059
KEGG_PATHWAY	hsa04211:Longevity regulating pathway	0.009296197
KEGG_PATHWAY	hsa04150:mTOR signaling pathway	0.023269017
KEGG_PATHWAY	hsa03013:RNA transport	0.028474248
KEGG_PATHWAY	hsa05164:Influenza A	0.029688848
KEGG_PATHWAY	hsa03060:Protein export	0.035553067
KEGG_PATHWAY	hsa04024:cAMP signaling pathway	0.037060694
KEGG_PATHWAY	hsa00051:Fructose and mannose metabolism	0.049997293

Significantly enriched pathways in DLCN_BC		
Category	Term	P-Value
KEGG_PATHWAY	hsa05160:Hepatitis C	0.0000974
KEGG_PATHWAY	hsa04071:Sphingolipid signaling pathway	0.001328309
KEGG_PATHWAY	hsa05162:Measles	0.001841588
KEGG_PATHWAY	hsa03010:Ribosome	0.001918092
KEGG_PATHWAY	hsa04550:Signaling pathways regulating pluripotency of stem cells	0.002076984
KEGG_PATHWAY	hsa05164:Influenza A	0.003762174
KEGG_PATHWAY	hsa05213:Endometrial cancer	0.003993438
KEGG_PATHWAY	hsa05168:Herpes simplex infection	0.004378412
KEGG_PATHWAY	hsa05200:Pathways in cancer	0.005229284
KEGG_PATHWAY	hsa05203:Viral carcinogenesis	0.005709864
KEGG_PATHWAY	hsa04015:Rap1 signaling pathway	0.006175297
KEGG_PATHWAY	hsa05230:Central carbon metabolism in cancer	0.006438404
KEGG_PATHWAY	hsa04810:Regulation of actin cytoskeleton	0.006497832
KEGG_PATHWAY	hsa00562:Inositol phosphate metabolism	0.007180301
KEGG_PATHWAY	hsa05218:Melanoma	0.007180301
KEGG_PATHWAY	hsa01524:Platinum drug resistance	0.007959021
KEGG_PATHWAY	hsa05100:Bacterial invasion of epithelial cells	0.008566907
KEGG_PATHWAY	hsa01521:EGFR tyrosine kinase inhibitor resistance	0.009194987
KEGG_PATHWAY	hsa05215:Prostate cancer	0.010966928
KEGG_PATHWAY	hsa03015:mRNA surveillance pathway	0.011667186
KEGG_PATHWAY	hsa04070:Phosphatidylinositol signaling system	0.01312504
KEGG_PATHWAY	hsa05146:Amoebiasis	0.013627751
KEGG_PATHWAY	hsa04152:AMPK signaling pathway	0.020590294
KEGG_PATHWAY	hsa04151:PI3K-Akt signaling pathway	0.022169577
KEGG_PATHWAY	hsa04650:Natural killer cell mediated cytotoxicity	0.023711161
KEGG_PATHWAY	hsa05322:Systemic lupus erythematosus	0.024033324
KEGG_PATHWAY	hsa04910:Insulin signaling pathway	0.025010608
KEGG_PATHWAY	hsa04261:Adrenergic signaling in cardiomyocytes	0.02838305
KEGG_PATHWAY	hsa04390:Hippo signaling pathway	0.030133971
KEGG_PATHWAY	hsa04150:mTOR signaling pathway	0.030133971
KEGG_PATHWAY	hsa00120:Primary bile acid biosynthesis	0.030734799
KEGG_PATHWAY	hsa00220:Arginine biosynthesis	0.037437282
KEGG_PATHWAY	hsa04360:Axon guidance	0.038325909
KEGG_PATHWAY	hsa05034:Alcoholism	0.039502146
